# People and research: improved health systems for West Africans, by West Africans - report on special supplement

**DOI:** 10.1186/s12919-019-0162-0

**Published:** 2019-02-07

**Authors:** Sue Godt, Sharmila Mhatre, Anne-Marie Schryer-Roy

**Affiliations:** 1 Maternal and Child Health Program, International Development Research Centre, Box 62084, Nairobi, PO 00200 Kenya; 2 Open Society Foundations, New York, NY 10019 USA; 3 Independent consultant, Po Box 91, Nairobi, 00606 Kenya

## Background

In 2011, the International Development Research Centre (IDRC) Governance for Equity in Health Systems programme, now named the Maternal and Child Health programme, embarked on a concerted effort to strengthen research in West Africa to improve equitable health systems. Systematic consultations with stakeholders in the region pointed to persistent challenges that made it difficult for health systems to promote good health and provide quality and sustainable services for the most vulnerable. The research environment did not enable the building of relevant skills or catalyse the needed resources to mount comprehensive research programmes that would strengthen health systems and address national priorities. Researchers were often fragmented by discipline, language and national boundaries. Even with relevant findings, there were weak research-to-policy-and-practice mechanisms and processes to apply the results.

To overcome this fragmentation, IDRC collaborated with the West African Health Organisation (WAHO), a regional body with the mandate to engage with the 15 member states of the Economic Community of West African States (ECOWAS), to strengthen policy and collective practice to improve health outcomes. Recognizing that change cannot be imposed from the outside but must be driven from within the region, the main objective was to strengthen a critical mass of researchers, research institutions, practitioners and decision-makers to undertake and apply relevant research to strengthen health systems and contribute to improving health outcomes. The journal supplement – and this translated proceedings report – are outputs of efforts to strengthen the research and evidence-based policy environment.

### Emerging themes

The articles present evidence around two consistent themes that emerged from the work.

#### Context matters

It is critical to understand the context in which interventions are developed and implemented. It is not enough to simply ‘adapt’ promising innovations developed elsewhere. The evidence shows how context can change the shape of externally imposed interventions or policies resulting in unintended outcomes.

#### Overcoming the fragmentation

There is need to overcome the existing fragmentation of expertise, knowledge and actors by strengthening collaboration across expert institutions and individuals. Evidence shows the extent of the geographical and linguistic divides in the production of research across the region. This fragmentation exacerbates efforts to undertake the most needed research to address health challenges. Authors point to the need to build strong working relationships amongst researchers, decision-makers and practitioners, so they can effectively work together to identify priority issues that can realistically be addressed given the available windows of opportunity.

### The collection

The collection includes a mix of 11 commentary, review and research articles which present evidence highlighting the barriers to sustainable innovation and change. They also demonstrate why health systems need to be holistically strengthened given how vertical interventions focusing on one specific outcome can have skewing effects on the delivery and impact of related healthcare services.

The articles in French are attached as additional files. Their abstracts are highlighted below.

We acknowledge with appreciation the support from the reviewers who provided very useful feedback on the originally submitted articles: Ties Boerma, University of Manitoba; Erica di Ruggiero, University of Toronto; Marc-Eric Gruenais, Université de Bordeaux; Fréderic Le Marcis, Ecole normale supérieure de Lyon; Sharmila Mhatre, Open Society Foundations; Alison Riddle, Health and gender equality advisor, Canada; Helen Schneider, University of Western Cape; Werner Soors, Institute of Tropical Medicine, Antwerp; Benjamin Uzochukwu, University of Nigeria Nsukka; Wim van Damme, Institute of Tropical Medicine, Antwerp.

Godt, S., Mhatre, S., Schryer-Roy, A-M. ***Les artisans du changement de l’Afrique de l’Ouest*** (Originally published as The change-makers of West Africa).

This introductory article provides overall background to IDRC’s approach in the region and to the implementation of the specific initiative. It draws out the key themes around context and fragmentation and reflects on the implications of the evidence for global health. There are issues around the governance of health research, around whose agendas matter and on the importance of starting implementation from the local level to inform global arenas. The impact of funder agendas, priorities and practices, in particular the skewing effects of vertical programmes are noted. The authors reflect on the importance of vibrant West African-led collaborations amongst researchers, decision-makers and civil society which are effectively supported by national, regional and global funding to foster, strengthen and use locally-generated evidence to ensure that efforts to strengthen health systems and improve regional health outcomes are successful. (Additional file 1).

Sombie, I., Bouwayé, A., Mongbo, Y., Keita, N., Lokossou, V., Johnson, E., Assogba, L., Crespin, X. ***Promouvoir la recherche pour améliorer la santé des mères, des nouveau-nés, des nourrissons et des adolescents en Afrique de l’Ouest***: ***le rôle de l’Organisation ouest- africaine de la santé*** (Originally published as Promoting research to improve maternal, neonatal, infant and adolescent health in West Africa: the role of the West African Health Organisation).

The West African Health Organisation is a specialised institution of the Economic Community of West African States (ECOWAS). This commentary examines the regional organisation’s role in promoting research as a tool for strengthening maternal and infant health in West Africa. WAHO’s experiences demonstrate how a regional health institution can integrate research promotion into the fight against maternal and infant mortality. At the same time, the challenges encountered also demonstrate the importance of cohesion, collaboration and coordination amongst country actors and all partners in promoting such initiatives. The critical need is underlined for leadership and commitment by country actors to steer these processes. (Additional file 2).

Koroma, M., Kamara, S., Bangura, E., Kamara, M., Lokossou, V., Keita, N. ***La qualité des services prénataux et d’accouchement gratuits au nord de la Sierra Leone*** (Originally published as The quality of free antenatal and delivery services in Northern Sierra Leone).

This research reports on a survey of health facilities undertaken in Bambali District, northern Sierra Leone in 2014. Sierra Leone had responded to having the highest maternal mortality ratio in sub-Saharan Africa by removing financial barriers in 2010 to encourage use of available antenatal, delivery and postnatal services. This study examined the quality of free antenatal services and access to emergency obstetric care. Measured against national standards, the quality of general maternal and emergency obstetric services was poor. The findings point to the need to monitor quality in addition to access as well as to investigate continuing inequities adversely influencing the uptake of available services. (Additional file 3).

Yaogo, M. ***Gratuité ou subvention***: ***options d’exemption de paiement des frais de santé, accès aux soins des groupes vulnérables et effets sur le système de santé au Burkina Faso*** (Originally published as Free versus subsidised healthcare: options for fee exemptions, access to care for vulnerable groups and effects on the health system in Burkina Faso).

This article examines the experience of both total exemption (free) and cost subsidisation options of healthcare that have been implemented in Burkina Faso over the past two decades. Contextual variations and the ways in which the options affect access to healthcare for vulnerable people as well as the operation of the health system were studied. Results were summarised in reference to a conceptual framework and a chronological review of policy interventions. Stakeholder perspectives pointed to the importance of more equitable governance of the many different approaches. (Additional file 4).

Duclos, V., Yé, M., Moubassira, K., Sanou, H., Sawadogo, H., Bibeau, G., Sié, A. ***État de la santé mobile***: ***étude qualitative sur les attentes relatives à la santé mobile dans le district sanitaire rural de Nouna, au Burkina Faso*** (Originally published as Situating mobile health: a qualitative study of mHealth expectations in the rural health district of Nouna, Burkina Faso).

This study investigates the expected benefits, challenges and limitations associated with mHealth interventions, particularly from the experiences and perspectives of direct and indirect intended users of the system including health workers, community members, and pregnant women. The evidence demonstrated that mHealth expectations are deeply linked to context and are inseparable from local health-related experiences, practices and constraints. It is important to move beyond universalistic approaches. (Additional file 5).

Agyepong, I., Kwamie, A., Frimpong, E., Defor, S., Ibrahim, A., Aryeetey, G., Lokossou, V., Sombie, I. ***Jeter un pont entre le programme sur la santé des mères, des nouveau-nés et des enfants (SMNE) et les recherches sur les systèmes de santé***: ***facteurs propices et défavorables liés aux systèmes de santé pour améliorer les incidences de la SMNE en Afrique de l’Ouest*** (Originally published as Spanning maternal, newborn and child health (MNCH) and health systems research boundaries: conducive and limiting health systems factors to improving MNCH outcomes in West Africa).

Beyond our knowledge of *what* interventions work, insights are needed on other factors that facilitate or inhibit improved maternal newborn and child health (MNCH) outcomes. This study aimed to explore both health system factors conducive or limiting to MNCH policy and programme implementation and outcomes in West Africa, and how and why they work in context. The evidence demonstrated that numerous MNCH policies and interventions were being piloted, researched or implemented at scale in the sub-region. Most faced multiple interacting conducive and limiting health system factors that impacted on effective implementation. Context acted through its effect on both health system factors and the social determinants of health. To accelerate and sustain improved MNCH outcomes in West Africa, an integrated approach to research and practice is needed that simultaneously addresses health systems and contextual factors alongside MNCH service delivery interventions. This requires multi-level, multi-sectoral and multi-stakeholder engagement approaches that span current geographical, language, research and practice community boundaries in West Africa, and effectively link the efforts of actors interested in health systems strengthening with those of actors interested in MNCH outcome improvement. (Additional file 6).

Olivier de Sardan, J-P., Diarra, A., Moha, M. ***Les modèles voyageurs à l’épreuve des contextes et des normes pratiques: le cas de la santé maternelle*** (Originally published as Travelling models and the challenge of pragmatic contexts and practical norms: the case of maternal health).

This review article examines the proliferation of ‘traveling models’ developed by international experts and introduced in an almost identical format across numerous low-and-middle-income countries to improve some aspect of maternal health systems. Specific examples are reviewed showing how standardised interventions often result in drifts and distortions once they engage with real contexts. The article concludes by suggesting that an alternative approach would be to start with the daily reality of social and practical norms instead of relying on models, and to promote innovations that emerge from within local health systems. (Additional file 7).

Sombie, I., Aidam, J., Montorzi, G. ***Évaluation d’un projet régional visant à renforcer les systèmes nationaux de recherche en santé dans quatre pays d’Afrique de l’Ouest: leçons apprises*** (Originally published as Evaluation of regional project to strengthen national health research systems in four countries in West Africa: lessons learned).

The article presents evidence of efforts to strengthen systems for health research in four post-conflict West African countries – Guinea-Bissau, Liberia, Sierra Leone and Mali. Four key issues were examined: governance and management of research, capacities, funding, and dissemination/use of research findings. The lessons demonstrated that the fragile context of these countries requires long-term engagement and that support from a regional institution is needed to address existing challenges and successfully strengthen the entire national health research system. (Additional file 8).

Defor, S., Kwamie, A., Agyepong, I. ***Comprendre l’état de la recherche sur les politiques et les systèmes de santé en Afrique occidentale et les besoins de renforcement des capacités***: ***portée des tendances et des caractéristiques des publications à comité de lecture de 1990 à 2015*** (Originally published as Understanding the state of health policy and systems research in West Africa and capacity strengthening needs: scoping of peer-reviewed publications trends and patterns 1990–2015).

Health policy and systems research (HPSR) is a field with great potential for addressing many of the sub-region’s intransigent health challenges. This paper presents an analysis of trends and patterns of peer-reviewed HPSR publications across the Economic Community of West African States (ECOWAS), particularly around the degree of involvement of West African researchers in HPSR evidence generation. The goal was to use the findings to inform the development of a sub-regional strategy to strengthen HPSR and its use to inform development and improvement of health outcomes. Despite progressive improvements over time, West Africa remains a weak sub-region in terms of peer-reviewed HPSR publications. Bridging the gap between lead institutions (universities and research centres) and the practice community (ministries, hospitals, non-governmental organisations) is indispensable for ensuring the practical application of HPSR evidence. There remains a major need for investments in HPSR capacity building in West Africa. (Additional file 9).

Keita, N., Lokossou, V., Berthe, A., Sombie, I., Johnson, E., Busia, K. ***L’expérience ouest-africaine de constitution de comités de pilotage pour améliorer la collaboration entre chercheurs et décideurs et accroître l’utilisation des résultats de la recherche en santé*** (Originally published as The West African experience in establishing steering committees for better collaboration between researchers and decision-makers to increase the use of health research findings).

Aware of the advantages of a project steering committee (SC) to influence the development of evidence-based health policies, the West African Health Organisation (WAHO) encouraged and supported the creation of such SCs around research projects in four countries (Burkina Faso, Nigeria, Senegal and Sierra Leone). This study was conducted to describe the process that was used to establish these committees and its findings aim to assist other stakeholders in initiating this type of process. The ‘learning-by-doing’ approach made it possible to develop strategies adapted to each context to create, facilitate and operate each SC and manage its difficulties. To reproduce such an experience, a strong understanding of the local context and the involvement of strong partners are required. (Additional file 10).

Uneke, C., Sombie, I., Keita, N., Lokossou, V., Johnson, E., Ongolo-Zogo, P. ***Amélioration des processus d’élaboration de politiques sur la santé des mères et des enfants au Nigéria***: ***une évaluation des besoins des décideurs, des obstacles et des éléments facilitateurs de l’élaboration de politiques fondées sur des données probantes*** (Originally published as Improving maternal and child health policymaking processes in Nigeria: an assessment of policymakers’ needs, barriers and facilitators of evidence-informed policymaking).

In Nigeria, interest in the evidence-to-policy process is gaining momentum among policymakers involved in maternal, newborn and child health (MNCH). However, numerous gaps exist amongst policymakers on the use of research evidence in policymaking. The objective of this study was to assess the perception of MNCH policymakers regarding their needs and the barriers and facilitators to use of research evidence in policymaking in Nigeria. The results underlined the need to establish mechanisms that will facilitate the movement from evidence to policy and address the needs identified by policymakers. It is also imperative to improve organisational initiatives that facilitate use of research evidence for policymaking. (Additional file 11).

## Additional files

Additional file 1: Les artisans du changement de l’Afrique de l’Ouest, Godt, S., Mhatre, S., Schryer-Roy, A-M.

Additional file 2: Promouvoir la recherche pour améliorer la santé des mères, des nouveau-nés, des nourrissons et des adolescents en Afrique de l’Ouest: le rôle de l’Organisation ouest-africaine de la santé, Sombie, I., Bouwayé, A., Mongbo, Y., Keita, N., Lokossou, V., Johnson, E., Assogba, L., Crespin, X.

Additional file 3: La qualité des services prénataux et d’accouchement gratuits au nord de la Sierra Leone, Koroma, M., Kamara, S., Bangura, E., Kamara, M., Lokossou, V., Keita, N.

Additional file 4: Gratuité ou subvention: options d’exemption de paiement des frais de santé, accès aux soins des groupes vulnérables et effets sur le système de santé au Burkina Faso, Yaogo, M.

Additional file 5: État de la santé mobile: étude qualitative sur les attentes relatives à la santé mobile dans le district sanitaire rural de Nouna, au Burkina Faso, Duclos, V., Yé, M., Moubassira, K., Sanou, H., Sawadogo, H., Bibeau, G., Sié, A.

Additional file 6: Jeter un pont entre le programme sur la santé des mères, des nouveau-nés et des enfants (SMNE) et les recherches sur les systèmes de santé: facteurs propices et défavorables liés aux systèmes de santé pour améliorer les incidences de la SMNE en Afrique de l’Ouest, Agyepong, I., Kwamie, A., Frimpong, E., Defor, S., Ibrahim, A., Aryeetey, G., Lokossou, V., Sombie, I.

Additional file 7: Les modèles voyageurs à l’épreuve des contextes et des normes pratiques: le cas de la santé maternelle, Olivier de Sardan, J-P., Diarra, A., Moha, M.

Additional file 8: Évaluation d’un projet régional visant à renforcer les systèmes nationaux de recherche en santé dans quatre pays d’Afrique de l’Ouest: leçons apprises, Sombie, I., Aidam, J., Montorzi, G.

Additional file 9: Comprendre l’état de la recherche sur les politiques et les systèmes de santé en Afrique occidentale et les besoins de renforcement des capacités: portée des tendances et des caractéristiques des publications à comité de lecture de 1990 à 2015, Defor, S., Kwamie, A., Agyepong, I.

Additional file 10: L’expérience ouest-africaine de constitution de comités de pilotage pour améliorer la collaboration entre chercheurs et décideurs et accroître l’utilisation des résultats de la recherche en santé, Keita, N., Lokossou, V., Berthe, A., Sombie, I., Johnson, E., Busia, K.

Additional file 11: Amélioration des processus d’élaboration de politiques sur la santé des mères et des enfants au Nigéria: une évaluation des besoins des décideurs, des obstacles et des éléments facilitateurs de l’élaboration de politiques fondées sur des données probantes, Uneke, C., Sombie, I., Keita, N., Lokossou, V., Johnson, E., Ongolo-Zogo, P.

