# Supplementary material for: People and research: improved health systems for West Africans, by West Africans - report on special supplement
Source: BMC Proc. 2019 Feb 7;13(Suppl 1):1. doi: 10.1186/s12919-019-0162-0 (PMC6366023; doi:10.1186/s12919-019-0162-0)
Supplement: Supplementary file 1 — Les artisans du changement de l’Afrique de l’Ouest, Godt, S., Mhatre, S., Schryer-Roy, A-M. [file 12919_2019_162_MOESM1_ESM.docx]

***Les artisans du changement de l’Afrique de l’Ouest***

**Sue Godt,^*1^ Sharmila Mhatre^2^, et Anne-Marie Schryer-Roy^3^**

* Correspondance : [smg](mailto:sgodt@crdi.ca)odt@gmail.com

^1^ Programme Santé des mères et des enfants, Centre de recherches pour le développement international, C.P. 62084, 00200, Nairobi, Kenya

La liste complète des auteurs est disponible à la fin de l’article.

**Résumé**

L’Afrique de l’Ouest a fait l’objet de l’attention du monde entier au cours de l’éclosion du virus Ebola, alors que les faiblesses des systèmes de santé ont aggravé une urgence grave et compliqué les efforts d’intervention. À la suite de la crise, des demandes ont été formulées pour renforcer les systèmes de santé, mais les investissements réalisés à ce jour n’ont pas permis de fournir le soutien nécessaire pour créer des systèmes de santé solide qui permettraient de prévenir et de gérer les éclosions futures. En partie, cette réalité sert à mettre en évidence les lacunes des solutions auxquelles les experts et les bailleurs de fonds externes accordent constamment la priorité. Souvent, ces solutions ne permettent pas de prendre en considération l’abondance de données probantes de l’Afrique de l’Ouest, pas plus que les acteurs qui travaillent d’arrache-pied à renforcer le leadership ainsi que les systèmes de santé dont on a besoin pour produire et améliorer de façon durable les résultats sur la santé à l’échelle nationale. Malheureusement, ces connaissances et cette expérience se font rarement entendre sur la scène mondiale. Le présent supplément de revue constitue une contribution, quoique minime, visant à modifier cette pratique en exposant les perspectives, les expériences et les connaissances des Africains de l’Ouest. Il présente les résultats découlant d’une série de projets de renforcement des capacités et de recherche en Afrique, lesquels ont été financés par le Centre de recherches pour le développement international (CRDI) et le Programme de soins de santé maternelle et infantile, anciennement désigné par Gouvernance pour l’équité dans les systèmes de santé.

Les données probantes présentées ici s’articulent autour de deux principaux thèmes. Le premier thème est que le contexte est important. Les données probantes indiquent en quoi le contexte peut changer la manière dont sont façonnées les politiques et les interventions extérieures qui se traduisent par des résultats inattendus. Parallèlement, elles mettent en lumière les données probantes montrant la façon dont les acteurs locaux novateurs élaborent leurs propres approches, qui sont habituellement économiques et intégrées au contexte, en vue de susciter un changement. Deuxièmement, les divers articles traitent du besoin crucial de surmonter la fragmentation existante de l’expertise, des connaissances et des acteurs, et d’établir de solides relations de travail entre tous les acteurs afin qu’ils puissent travailler efficacement ensemble pour cerner les questions prioritaires qui peuvent être abordées, de manière réaliste, compte tenu des occasions offertes.

Les collaborations dynamiques menées en Afrique de l’Ouest entre les chercheurs, les décideurs et la société civile, qui sont appuyées efficacement par le financement national, régional et mondial, doivent favoriser, renforcer et utiliser les données probantes à l’échelle locale pour assurer le succès des efforts visant à renforcer les systèmes de santé et à améliorer les résultats en matière de santé à l’échelle régionale. Il est clair que l’on ne trouvera pas les solutions dans les « modèles de déplacement » des interventions normalisées.

**Introduction**

L’Afrique de l’Ouest a fait l’objet de l’attention du monde entier au cours de l’éclosion du virus Ebola [1, 2]. L’épidémie a tragiquement illustré comment les lacunes du système de santé, y compris des systèmes nationaux de surveillance des maladies inadéquats ou inexistants, ont contribué à la dégradation d’une urgence grave et compliqué les efforts d’intervention [3-6]. Même si des demandes ont été émises pour renforcer les systèmes de santé dans le cadre des efforts de reconstruction à la suite de la crise du virus Ebola, les investissements réalisés à ce jour n’ont pas permis de fournir le soutien nécessaire pour créer des systèmes de santé solide capables de prévenir et de gérer les éclosions futures [7].

En partie, cette lacune est attribuable au défi que pose le renforcement des systèmes de santé. Toutefois, par la même occasion, elle met aussi en évidence les échecs des solutions auxquelles les experts et les bailleurs de fonds externes accordent constamment la priorité. La manchette manquante dans tout ce qu’on a pu dire ou écrire au sujet de l’épidémie du virus Ebola est que l’Afrique de l’Ouest regroupe, outre une abondance de données probantes, des chercheurs, des praticiens, des décideurs et des acteurs de la société civile, lesquels travaillent activement à renforcer le leadership et les systèmes de santé dont on a besoin pour produire et améliorer de façon durable les résultats sur la santé à l’échelle nationale. Malheureusement, ces connaissances et cette expérience se font rarement entendre sur la scène mondiale. En fait, les investissements, les discours et les programmes d’aide en matière de recherche et de développement ont tendance à être dictés par des fonds, des intérêts et des voix externes, ce qui laisse peu de marge de manoeuvre aux chercheurs et aux dirigeants de l’Afrique de l’Ouest pour partager leurs points de vue et leurs expériences.

Le présent supplément de revue constitue une contribution, quoique minime, visant à modifier cette pratique en exposant les perspectives, les expériences et les connaissances des Africains de l’Ouest. Il vise à apporter des pistes de réflexion et examine de quelle façon les intervenants – de l’échelle locale à l’échelle mondiale – pourraient jouer plus efficacement leur rôle pour soutenir les efforts de renforcement des systèmes de santé en Afrique de l’Ouest.

En 2011, le programme Gouvernance pour l’équité dans les systèmes de santé du CRDI, maintenant appelé le programme de soins de santé maternelle et infantile, s’est engagé dans un effort concerté visant à renforcer la recherche en Afrique de l’Ouest pour améliorer les systèmes de santé équitables. Les consultations systématiques avec les intervenants dans la région ont révélé des défis persistants qui ont généré des répercussions négatives sur la santé. Les systèmes de santé n’ont pas été en mesure de faire la promotion de la santé et de fournir des services durables de qualité aux personnes les plus vulnérables. De plus, le milieu de la recherche n’a pas permis de développer les compétences pertinentes ou de catalyser les ressources nécessaires pour lancer des programmes de recherche complets qui renforceraient les systèmes de santé et répondraient aux priorités nationales. Les chercheurs voyaient souvent leur travail fragmenté, en raison de la diversité des disciplines et des langues, de même que des frontières nationales. Malgré des résultats pertinents, on constatait une faiblesse dans les mécanismes et les processus servant à allier la recherche, les politiques et les pratiques pour appliquer les résultats.

En collaboration avec l’Organisation ouest-africaine de la santé (OOAS), un organisme régional ayant le mandat de collaborer avec les 15 États membres de la Communauté économique des États de l’Afrique de l’Ouest (CÉDÉAO) en vue de renforcer les politiques et les pratiques collectives en vue d’améliorer les résultats sur la santé, on a élaboré un plan dont la priorité était de surmonter cette fragmentation. Par la suite, ces problèmes ont été intégrés dans un appel à notes conceptuelles à l’échelle régionale. En se fondant sur le constat que le changement ne peut pas s’imposer de l’extérieur, mais qu’il doit plutôt provenir de l’intérieur de la région, le principal objectif était de consolider une masse critique de chercheurs, d’instituts de recherche, de praticiens et de décideurs afin qu’ils mènent et appliquent les recherches pertinentes pour renforcer les systèmes de santé et qu’ils contribuent à améliorer les résultats en matière de santé. Ainsi, le présent supplément de revue constitue l’un des extrants du programme de travail du CRDI visant à renforcer le milieu de la recherche.

**Thèmes émergents**

Les articles du présent supplément présentent des données probantes soulignant les obstacles à l’innovation et au changement durables. De plus, ils démontrent les motifs pour lesquels les systèmes de santé doivent être renforcés de manière globale, étant donné que les interventions verticales qui se concentrent sur un résultat précis peuvent avoir des effets biaisés sur la prestation et les répercussions des services de soins de santé connexes [8-11]. Par ailleurs, le contexte est important : il ne suffit pas « d’adapter » des innovations prometteuses qui ont été mises au point ailleurs. Les données probantes indiquent en quoi le contexte peut changer la manière dont sont façonnées les politiques et les interventions extérieures qui se traduisent par des résultats inattendus [12].

Ainsi, Defor et coll. [13] ont présenté des données probantes sur l’ampleur de la division géographique et linguistique dans la production de la recherche. Cette fragmentation intensifie les efforts visant à entreprendre les recherches les plus pressantes pour relever les défis liés à la santé.

Par la même occasion, les articles décrivent les efforts qui sont déployés actuellement afin de renforcer le milieu de la recherche pour la production et l’utilisation des données probantes qui permettront de soutenir un changement durable [14, 15]. Afin d’atténuer une partie de la fragmentation géographique, disciplinaire et linguistique, l’OOAS démontre de quelle façon la gouvernance de la recherche, combinée à de solides plateformes d’engagement envers les pratiques et les politiques, peut jouer un rôle de soutien [16, 17]. Cette fondation contribue à renforcer les collaborations en recherche de même que celles entre les intervenants aux échelles nationale et régionale.

**Le contexte compte**

L’un des principaux thèmes découlant des articles souligne l’importance de la portée des interventions ainsi que du contexte dans lequel ces dernières sont élaborées. En Sierra Leone, Koroma et coll. [8] examinent les efforts qui ont été déployés en vue de fournir des soins gratuits en matière de soins de santé maternels dans le district rural de Bombali, avant l’éclosion du virus Ebola, et ils constatent que, malgré de nombreuses demandes de la population en ce sens, la mauvaise qualité des services a compromis les résultats. Des infrastructures déficientes, un personnel disposant de compétences inadéquates et la disponibilité réduite des approvisionnements combinés à des inégalités structurelles et à un manque de mécanismes de financement durables, ont fait en sorte de compromettre la prestation des services. Au Burkina Faso, Yaogo [11] fait appel à des recherches antérieures sur les politiques en vue d’abolir – en partie ou entièrement – différents frais de soins de santé, et il cerne des obstacles semblables qui préviennent la prestation de services de qualité accessibles. Par ailleurs, les deux articles soulignent les effets biaisés des programmes financés par des sources externes. En Sierra Leone, le pourcentage de femmes enceintes qui ont été soumises à un test de dépistage du VIH s’est avéré beaucoup plus élevé que pour tout autre test prénatal [8], et ce, en raison de leur participation à un programme national dicté par les bailleurs de fonds. Au Burkina Faso, il y a eu des problèmes liés à la gestion de toutes les différentes contributions de bailleurs de fonds, souvent isolées, et servant à subventionner la prévention et le traitement des maladies [11].

Duclos et coll. [10] examinent les interventions en santé mobile dans le district de santé de Nouna, au Burkina Faso, selon le point de vue et les attentes des utilisateurs et des travailleurs en santé de première ligne. Même si les téléphones mobiles sont utiles pour créer un réseau de soutien, ils ne peuvent pas éliminer les importants obstacles sous-jacents qui empêchent l’accès aux soins prénataux, y compris la pauvreté, les questions sexospécifiques et les distances géographiques. Les auteurs mettent en garde contre le fait d’appliquer des approches universalistes à la santé mobile, et revendiquent une *« élaboration de politiques et une conception de projets soignées, qui prennent en compte les connaissances et les pratiques des communautés locales »*.

En recourant à la santé des mères, des nouveau-nés et des enfants à titre d’exemple, Agyepong et coll. [18] soulignent la manière dont le contexte ainsi que les facteurs liés aux systèmes de santé (les composantes de base fondamentales de l’Organisation mondiale de la Santé, combinées aux gens, au pouvoir, aux processus et aux valeurs) ont joué un rôle habilitant ou limitatif pour réduire ou accroître les répercussions des interventions en santé.

Comme les auteurs le soulignent, on a mis en oeuvre plusieurs interventions « éprouvées » ou « présumées efficaces » visant à s’attaquer aux problèmes tels que la mortalité maternelle et infantile, mais les résultats ne se sont pas toujours améliorés comme prévu.

Olivier de Sardan et coll. [12] élaborent une théorie ancrée afin de *« comprendre la relation entre les interventions normalisées et les contextes de mise en oeuvre, ainsi que les nombreux effets inattendus, invisibles ou pervers »* qui en découlent. Les auteurs étudient en détail la « vengeance du contexte » et, grâce à une analyse de l’élaboration et de la diffusion des « modèles de déplacement » des interventions normalisées, ils soulignent l’assise de données probantes au Niger et dans d’autres pays qui démontrent l’écart entre les conditions locales et interventions extérieures et les normes professionnelles et sociales. Souvent, les institutions internationales, les organisations non gouvernementales et d’autres acteurs jouent le rôle « d’agences de voyages » en vue de faciliter le transfert de tels modèles. Enfin, les auteurs concluent en soulignant les données probantes qui témoignent de la façon dont les acteurs locaux novateurs élaborent leurs propres approches, lesquelles sont habituellement économiques et intégrées au contexte, afin de susciter un changement.

**Surmonter la fragmentation**

En guise de deuxième thème, plusieurs auteurs cernent la nécessité absolue de surmonter la fragmentation actuelle au chapitre de l’expertise, des connaissances et des acteurs, en renforçant la collaboration entre les personnes et les institutions spécialisées. De plus, ils soulignent la nécessité d’établir de solides relations de travail entre les chercheurs, les décideurs et les praticiens pour qu’ensemble, ces gens puissent travailler efficacement en vue de cerner les questions prioritaires qui peuvent être abordées, de manière réaliste, compte tenu des occasions offertes.

Sombie, Aidam et Montorzi [16] discutent de la façon dont les systèmes nationaux de recherche en santé dans quatre États fragiles ont été financés afin d’établir les assises qui permettront d’entreprendre et d’utiliser les recherches pertinentes dans le domaine de la santé. Malgré les difficultés contextuelles, comme l’instabilité politique et l’éclosion du virus Ebola, certains progrès ont été réalisés pour améliorer la gouvernance nationale, notamment en élaborant des priorités et des politiques nationales en matière de recherche sur la santé, en renforçant l’examen de l’éthique et en mettant en place un système régional d’information sur la recherche. De plus, les auteurs ont réfléchi sur le rôle d’accompagnement stratégique que joue l’OOAS, en tant qu’organisation régionale, et sur la nécessité, pour les acteurs sur la scène internationale, d’appuyer les activités de renforcement des capacités, de mobilisation des ressources et de défense des intérêts qui répondent aux priorités nationales et régionales. Dans le cadre de leur examen de la littérature concernant la recherche sur les politiques et les systèmes de santé, Defor et coll. [13] décrivent les habitudes et les tendances des publications examinées par les pairs, aussi bien anglophones que francophones, qui ont été diffusées dans les pays de la CÉDÉAO, de 1990 à 2015.

Même si leurs conclusions indiquent une augmentation des taux de production de recherche depuis 2008, on constate que cette région est encore bien en retard sur les autres régions. Le Nigéria, le Burkina Faso et le Ghana produisent plus de 70 % des publications, dont la plupart sont rédigées en anglais.

Étant donné que la recherche sur les politiques et les systèmes de santé dépend du contexte, les auteurs soulignent qu’il est urgent que les *« acteurs locaux qui comprennent et connaissent les problèmes liés à leurs propres systèmes de santé dirigent les processus permettant de produire et d’appliquer les données probantes »* [13]. Par ailleurs, en guise de moyen pour surmonter la fragmentation actuelle, les auteurs sont favorables à une plus grande collaboration institutionnelle entre les pays, laquelle mettrait l’accent sur l’établissement d’un programme de recherche conjoint avec les utilisateurs de la recherche.

Puisque les mécanismes servant à influer sur les politiques et les pratiques varient et sont faibles dans bon nombre de pays, Keita et coll. [15] font le point sur une initiative régionale visant à stimuler, à promouvoir et à renforcer la collaboration entre les chercheurs, les acteurs et les décideurs afin qu’ils puissent mener des recherches et utiliser les résultats en vue d’améliorer la gouvernance et l’équité des systèmes de santé. À la lumière des données probantes figurant dans la littérature, l’OOAS a encouragé la mise sur pied de comités directeurs pour accompagner quatre projets de recherche sur les systèmes de santé au Burkina Faso, au Nigéria, au Sénégal et en Sierra Leone. Chaque comité a pris une forme différente selon le contexte local et national, et cette flexibilité a permis de renforcer la mise en oeuvre. Par exemple, dans les campagnes de la Sierre Leone, le comité directeur local comprenait des intervenants de la collectivité et des districts qui provenaient des secteurs des transports, de la défense et de la sécurité, et qui étaient tous engagés à faire en sorte que la politique relative aux soins de santé maternels gratuits soit fonctionnelle pour tous. Cette expérience a contribué à l’établissement de relations à plus long terme entre les équipes de recherche et les décideurs. Néanmoins, et compte tenu de la dépendance à l’égard des chercheurs subventionnés pour l’obtention des ressources, on a cerné le besoin d’autonomie de tous les comités et la nécessité de renforcer leur action.

Uneke et coll. [14] étudient l’augmentation de la demande en données probantes, en évaluant les perceptions des intervenants et des décideurs politiques nigériens au sujet de leurs besoins en ce qui a trait à l’utilisation des données probantes émanant de la recherche dans l’élaboration des politiques, ainsi que les obstacles et les éléments facilitateurs à une telle utilisation. Les obstacles cernés comprennent les suivants : « la capacité inadéquate des organisations à mener des recherches en lien avec les politiques; des affectations budgétaires tout aussi inadéquates pour les recherches en lien avec les politiques; l’indifférence des décideurs à l’égard des données probantes émanant de la recherche; une piètre diffusion des données probantes émanant de la recherche aux décideurs; et l’absence d’un forum d’interaction entre les chercheurs et les décideurs » [14]. De plus, l’étude met en évidence la nécessité de renforcer les capacités individuelles et institutionnelles, et d’améliorer le financement et l’infrastructure de recherche. Plus particulièrement, l’évaluation cerne aussi la nécessité de créer des plateformes durables permettant une interaction entre les chercheurs et les décideurs.

**Conclusion**

Les articles du présent supplément nous permettent de réfléchir sur la gouvernance de la recherche en santé, sur ceux pour qui les programmes comptent et, ultimement, sur l’importance d’amorcer la mise en oeuvre à l’échelle locale afin d’éclairer la scène mondiale. Aux échelons nationaux, les gouvernements prennent des engagements visant à renforcer le secteur de la santé [19] et la recherche en santé [20], mais dans bien des cas, ne remplissent pas leurs obligations à cet égard.

La situation est brouillée par les tensions entourant les pratiques, les priorités et les programmes des bailleurs de fonds, ainsi que leur incidence dans la région, surtout en ce qui a trait aux effets biaisés des programmes verticaux.

Malgré de bonnes intentions, il y a clairement un décalage au carrefour de la gouvernance régionale, nationale et mondiale en Afrique de l’Ouest. L’histoire complexe de la région, avec ses fossés linguistiques et coloniaux, continue d’influer sur les institutions, les normes et les pratiques. Les collaborations dynamiques menées en Afrique de l’Ouest entre les chercheurs, les décideurs et la société civile, qui sont appuyées efficacement par le financement national, régional et mondial, doivent favoriser, renforcer et utiliser les données probantes à l’échelle locale pour assurer le succès des efforts visant à renforcer les systèmes de santé et à améliorer les résultats en matière de santé à l’échelle régionale. Il est clair que l’on ne trouvera pas les solutions dans les « modèles de déplacement » des interventions normalisées.

Cette approche axée sur le développement s’est reflétée dans les engagements de l’ère des Objectifs du Millénaire pour le développement, qui ont été pris dans la Déclaration de Paris sur l’efficacité de l’aide au développement et le Programme d’action d’Accra, lesquels accordaient la priorité à l’harmonisation de l’aide au développement et à son adhésion aux priorités régionales et nationales. Même si cette vision ne s’est pas entièrement concrétisée [21], ces questions sont abordées, encore une fois, dans le contexte de la réalisation des objectifs de développement durable et les appels pour l’élaboration d’une Convention-cadre pour la santé mondiale [22].

Il n’y a pas de réponse facile, mais nous espérons que le présent supplément contribuera à susciter un dialogue continu au sujet de ces questions importantes.

**Abréviations**

CÉDÉAO : Communauté économique des États de l’Afrique de l’Ouest; CRDI : Centre de recherches pour le développement international; OOAS : Organisation ouest-africaine de la santé

**Remerciements**

Nous aimerions remercier spécialement les 11 examinateurs externes qui ont accepté de participer à cette importante initiative et sans qui il aurait été impossible d’élaborer le présent supplément de revue.

**Financement**

Les coûts de publication ont été financés par le Centre de recherches pour le développement international (CRDI). Toutefois, les points de vue exprimés dans la présente introduction n’engagent que les auteurs et ne sauraient être attribués au CRDI.

**Contribution des auteures**

Tous les auteurs approuvés ont contribué à l’élaboration et à la rédaction du présent article, et SG a dirigé la rédaction de ce dernier.

**Intérêts conflictuels**

Les auteurs déclarent qu’ils n’ont aucun intérêt conflictuel.

**Détails à propos des auteurs**

^1^ Programme Santé des mères et des enfants, Centre de recherches pour le développement international, C.P. 62084, 00200, Nairobi, Kenya ^2^ Open Society Foundation, New York, NY 10019, États-Unis. ^3^Consultante indépendante, C.P. 91, 00606, Nairobi, Kenya.

**Date de publication : 12 juillet 2017**

**Références**

1. Heymann, D.L. et coll., « Global health security: the wider lessons from the west, African Ebola virus disease epidemic », *Lancet*, vol. 385, 2015, p. 1884-909.

2. DuBois, M. et C. Wake, « The Ebola response in West Africa: Exposing the Politics and Culture of International Aid », *HPG Working Paper*, octobre 2015. Accès : [https://www.odi.org/sites/odi.org.uk/files/odi-assets/publications-opinion-files/9903.pdf.](https://www.odi.org/sites/odi.org.uk/files/odi-assets/publications-opinion-files/9903.pdf) Consulté le 5 juin 2017.

3. Kieny, M.-P. et coll., « Health-system resilience: reflections on the Ebola crisis in western Africa », *Bulletin of the World Health Organization*, vol. 92, n^o^ 12, 2014, p. 850. DOI : [10.2471/BLT.14.149278.](http://dx.doi.org/10.2471/BLT.14.149278)

4. O’Hare, B., « Weak health systems and Ebola », *Lancet Global Health*, vol. 3, n^o^ 2, 2015, e71-2.

5. Gates, B., « Perspective: The Next Epidemic — Lessons from Ebola », *New England Journal of Medicine*, vol. 372, 2015, p. 1381-1384. [DOI : 10.1056/NEJMp1502918.](http://dx.doi.org/10.1056/NEJMp1502918)

6. Save the Children, *A Wake-up Call: Lessons from Ebola for the World’s Health Systems*, 2015. Accès <https://www.savethechildren.net/sites/default/files/libraries/WAKE%20UP%20CALL%20REPORT%20PDF.pdf>. Consulté le 5 juin 2017.

7. Glassman, A., « After Ebola », *Finance and Development*, juin 2016. Accès : [http://www.imf.org/external/pubs/ft/fandd/2016/06/pdf/glassman.pdf.](http://www.imf.org/external/pubs/ft/fandd/2016/06/pdf/glassman.pdf) Consulté le 5 juin 2017.

8. Agyepong, I.A. et coll., « Spanning maternal, newborn and child health (MNCH) and health systems research boundaries: conducive and limiting health systems factors to improving MNCH outcomes in West Africa », *Health Research Policy and Systems*, vol. 15, suppl. 1, 2017. [DOI : 10.1186/s12961-017-0212-x.](http://dx.doi.org/10.1186/s12961-017-0212-x)

9. Koroma, M.M. et coll., « The quality of free antenatal and delivery services in Northern Sierra Leone », *Health Research Policy and Systems*, vol. 15, suppl. 1, 2017. DOI : [10.1186/s12961-017-0218-4.](http://dx.doi.org/10.1186/s12961-017-0218-4)

10. Duclos, V. et coll., « Situating mobile health: a qualitative study of mHealth expectations in the rural health district of Nouna, Burkina Faso », *Health Research Policy and Systems*, vol. 15, suppl. 1, 2017. [DOI : 10.1186/s12961-017-0211-y.](http://dx.doi.org/10.1186/s12961-017-0211-y)

11. Yaogo, M., « Gratuité vs subvention : options d’exemptions du paiement des frais de santé, accès aux soins des groupes vulnérables et effets sur le système de santé au Burkina Faso », *Health Research Policy and Systems*, vol. 15, suppl. 1, 2017. DOI : [10.1186/s12961-017-0210-z](http://dx.doi.org/10.1186/s12961-017-0210-z).

12. Olivier de Sardan, J.P. et coll., « Travelling models and the challenge of pragmatic contexts and practical norms: the case of maternal health », *Health Research Policy and Systems*, vol. 15, suppl. 1, 2017. DOI : [10.1186/s12961-017-0213-9](http://dx.doi.org/10.1186/s12961-017-0213-9).

13. Defor, S. et coll., « Towards a better understanding of the state of health policy and systems research in West Africa and the capacity strengthening needs: a review of peer-reviewed publication trends and patterns 1990-2015 », *Health Research Policy and Systems*, vol. 15, suppl. 1, 2017. [DOI : 10.1186/s12961-017-0215-7.](http://dx.doi.org/10.1186/s12961-017-0215-7)

14. Uneke, C.J. et coll., « Improving maternal and child health policymaking process in Nigeria: an assessment of policymakers’ needs, barriers and facilitators of evidence-informed policymaking », *Health Research Policy and Systems*, vol. 15, suppl. 1, 2017. DOI : [10.1186/s12961-017-0217-5.](http://dx.doi.org/10.1186/s12961-017-0217-5)

15. Keita, M. et coll., « The West African experience in establishing steering committees for better collaboration between researchers and decision-makers to increase the use of health research findings », *Health Research Policy and Systems*, vol. 15, suppl. 1, 2017. DOI : [10.1186/s12961-017-0216-6](http://dx.doi.org/10.1186/s12961-017-0216-6).

16. Sombie, I., J. Aidam et G. Montorzi, « Evaluation of regional project to strengthen national health research systems in four countries in West Africa: lessons learned », *Health Research Policy and Systems*, vol. 15, suppl. 1, 2017. DOI : [10.1186/s12961-017-0214-8.](http://dx.doi.org/10.1186/s12961-017-0214-8)

17. Sombie, I. et coll., « Promoting research to improve maternal, neonatal, infant and adolescent health in West Africa: the role of the West African Health Organisation », *Health Research Policy and Systems*, vol. 15, suppl. 1, 2017. DOI : [10.1186/s12961-017-0209-](http://dx.doi.org/10.1186/s12961-017-0209-5)5.

18. *The WHO Health Systems Framework*. Accès : [http://www.wpro.who.int/health_services/health systems_framework/en/.](http://www.wpro.who.int/health_services/health_systems_framework/en/) Consulté le 15 janvier 2017.

19. *Déclaration d’Abuja*, 2001. Accès : <http://www.un.org/ga/aids/pdf/abuja_declaration.pdf>. Consulté le 5 juin 2017.

20. *Bamako Call to Action on Research for Health*, 2008. Accès : [http://www.who.int/rpc/news/BAMAKOCALLTOACTIONFinalNov24.pdf.](http://www.who.int/rpc/news/BAMAKOCALLTOACTIONFinalNov24.pdf) Consulté le 15 janvier 2017.

*21. Évaluation de la mise en oeuvre de la Déclaration de Paris*. Accès : [http://www.oecd.org/fr/cad/evaluation/evaluationdelamiseenuvredeladeclarationdeparis.htm.](http://www.oecd.org/dac/evaluation/evaluationoftheimplementationoftheparisdeclaration.htm) Consulté le 15 janvier 2017.

22. Gostin, L.O. et coll., « The next WHO Director-General’s highest priority: A Global Treaty on the Human Right to Health », *Lancet Global Health*, vol. 4, n^o^ 12, 2016, e890-2.
