# Supplementary material for: People and research: improved health systems for West Africans, by West Africans - report on special supplement
Source: BMC Proc. 2019 Feb 7;13(Suppl 1):1. doi: 10.1186/s12919-019-0162-0 (PMC6366023; doi:10.1186/s12919-019-0162-0)
Supplement: Supplementary file 2 — Promouvoir la recherche pour améliorer la santé des mères, des nouveau-nés, des nourrissons et des adolescents en Afrique de l’Ouest: le rôle de l’Organisation ouest-africaine de la santé, Sombie, I., Bouwayé, A., Mongbo, Y., Keita, N., Lokossou, V., Johnson, E., Assogba, L., Crespin, X. [file 12919_2019_162_MOESM2_ESM.docx]

***Promouvoir la recherche pour améliorer la santé des mères, des nouveau-nés, des nourrissons et des adolescents en Afrique de l’Ouest : le rôle de l’Organisation ouest-africaine de la santé***

**Issiaka Sombie^1^*, Aicha Bouwayé^1^, Yves Mongbo^1^, Namoudou Keita^1^, Virgil Lokossou^1^, Ermel Johnson^1^, Laurent Assogba^1^, Xavier Crespin^1^**

^1^Organisation ouest-africaine de la santé, 175 Avenue Ouezzin Coulibaly, 01 BP 153 Bobo-Dioulasso 01, Burkina Faso

* *Auteur-ressource,* [isombie@wahooas.org](mailto:isombie@wahooas.org)

**Résumé**

L’Afrique de l’Ouest a adopté de nombreuses stratégies pour lutter contre la mortalité maternelle et infantile, offre des programmes nationaux de santé maternelle et infantile, et accueille de nombreux partenaires techniques et financiers actifs, ainsi que des organisations non gouvernementales. En dépit de ces mesures, les indicateurs de mortalité et de morbidité maternelles et infantiles restent très élevés. Dans cette analyse, des acteurs internes et des représentants de l’Organisation ouest-africaine de la santé (OOAS) examinent le rôle de l’organisation régionale lorsqu’il s’agit de promouvoir la recherche en tant qu’outil d’amélioration de la santé maternelle et infantile en Afrique de l’Ouest.

En tant qu’institution spécialisée de la Communauté économique des États de l’Afrique de l’Ouest (CÉDÉAO) responsable des questions de santé, l’OOAS a pour mission d’offrir le niveau le plus élevé en matière de prestations de soins de santé aux populations de la sous-région sur la base de l’harmonisation des politiques des États membres, de la mise en commun des ressources et de la coopération entre les États membres et les pays tiers en vue de trouver collectivement et stratégiquement des solutions aux problèmes de santé de la sous-région. Pour y parvenir, la principale stratégie d’intervention de l’OOAS est celle de la facilitation, étant donné que cela encourage la production et l’utilisation de données probantes pour éclairer la prise de décisions et renforcer les pratiques.

L’analyse des interventions de l’OOAS depuis 2000 a montré qu’elle avait entraîné des modifications de la gouvernance, de la gestion et du financement de la recherche, ainsi que du renforcement des capacités individuelles et institutionnelles, de la diffusion de la recherche, de la collaboration et des échanges entre les différents intervenants. Elle a également révélé de nombreux défis, comme l’appropriation des processus, l’engagement des pays membres, les faibles capacités institutionnelles et individuelles, la mobilisation et l’engagement des intervenants. Afin de renforcer la prise de décisions fondée sur des données probantes, en 2016, l’OOAS a créé un programme unique visant à améliorer la production, la diffusion et l’utilisation des données et des résultats de la recherche pour la planification des programmes sanitaires, l’objectif à terme étant d’améliorer la santé des populations.

Si les expériences de l’OOAS à ce jour montrent comment une institution de santé régionale peut intégrer la promotion de la recherche à la lutte contre la mortalité maternelle et infantile, les défis que l’organisation a relevés révèlent également l’importance d’une certaine cohésion entre les acteurs promouvant cette initiative, l’importance du leadership et de l’engagement des acteurs des pays membres pilotant ce processus, ainsi que la nécessité d’une collaboration et d’une coordination entre l’ensemble des partenaires des pays membres et de la région.

**Introduction**

Dans cette analyse, nous, acteurs internes et représentants de l’Organisation ouest-africaine de la santé (OOAS), examinons le rôle de notre institution régionale lorsqu’il s’agit de promouvoir la recherche en tant qu’outil de renforcement de la santé maternelle et infantile en Afrique de l’Ouest. Ces réflexions visent à décrire et analyser le processus mis en oeuvre par l’OOAS pour améliorer la recherche et l’environnement dans lequel elle est menée, en facilitant la collaboration de l’ensemble des intervenants nationaux et régionaux en vue d’améliorer la santé maternelle et infantile.

L’Afrique de l’Ouest dispose d’un certain nombre de stratégies pour lutter contre la mortalité maternelle et infantile, offre des programmes nationaux de santé maternelle et infantile, et accueille de nombreux partenaires techniques et financiers actifs, ainsi que des organisations non gouvernementales. En dépit de ces mesures, les indicateurs de mortalité et de morbidité maternelles et infantiles restent très élevés [1]. En 2015, un examen parrainé par l’OOAS a montré qu’en Afrique de l’Ouest, les obstacles freinant l’amélioration de ces indicateurs étaient liés au contexte et aux systèmes de santé. Les obstacles contextuels étaient notamment l’état des routes, la culture, la connaissance des risques et le statut de la femme, ceux liés au système de santé comprenaient entre autres la distance géographique des centres de santé, l’organisation de l’offre de services, la disponibilité et la capacité des services de santé, et enfin la qualité des soins. Cette analyse a montré que la combinaison de ces facteurs faisait augmenter la mortalité et la morbidité maternelles ou infantiles [2], de sorte que chaque pays est confronté à un défi unique : réduire l’ensemble de ces facteurs. Les problèmes autour de la gouvernance et la façon dont les décisions sont prises aggravent encore ces facteurs. En Afrique de l’Ouest, comme c’est le cas dans de nombreux pays en développement, les processus décisionnels ne se fondent pas toujours sur des données probantes et sont influencés par divers autres facteurs, ce qui donne souvent lieu à des politiques et des programmes déficients, en plus de limiter les efforts d’amélioration de la santé maternelle et infantile.

Par conséquent, le renforcement de la lutte contre la mortalité maternelle et infantile doit dépasser le cadre de la formation clinique et être adapté à cet éventail de facteurs plus large, dont le système national de recherche sur la santé. Cette démarche contribuera à améliorer la demande, l’accès et la qualité des soins, la satisfaction vis-à-vis de ceux-ci, ainsi que l’adaptation, l’exécution et la mise à l’échelle des interventions ou des stratégies efficaces [3]. L’un des points essentiels de cette démarche est que la recherche sera menée et utilisée pour orienter les politiques et les pratiques.

L’intégration de la recherche à ce processus, comme le préconisent l’Organisation mondiale de la Santé (OMS) et d’autres acteurs, nécessite de connaître sa valeur ajoutée et les compétences des divers acteurs en matière de recherche sur des systèmes de santé, définie comme de la recherche axée sur la gouvernance, les aspects financiers, les soins et les services offerts à la population, de même que sur le contexte dans lequel ils sont négociés, mis en oeuvre et refondés [4]. Cette recherche multidisciplinaire emploie des méthodes quantitatives et qualitatives, et nécessite un engagement des intervenants [5]. Elle peut être réalisée par des agents de santé, des gestionnaires de programme et des dirigeants nationaux du secteur des soins de santé [6]. Malheureusement, les lacunes techniques et organisationnelles limitent cette intégration dans les pays en développement, en particulier en Afrique de l’Ouest [7].

**Approche de l’OOAS pour améliorer la santé maternelle en Afrique de l’Ouest en promouvant la recherche**

L’OOAS est une institution spécialisée de la Communauté économique des États de l’Afrique de l’Ouest (CÉDÉAO) responsable des questions de santé. Elle exerce donc ses activités indépendamment de l’OMS, mais travaille en étroite collaboration avec le Bureau régional de l’Afrique de cette organisation. La mission de cette organisation régionale est d’offrir le niveau le plus élevé en matière de prestations de soins de santé aux populations de la sous-région sur la base de l’harmonisation des politiques des États membres, de la mise en commun des ressources et de la coopération entre les États membres et les pays tiers en vue de trouver collectivement et stratégiquement des solutions aux problèmes de santé de la sous-région. Pour accomplir sa mission et atteindre ses objectifs, la principale stratégie d’intervention de l’OOAS est celle de la facilitation, car ce faisant, elle encourage la production et l’utilisation de données probantes pour éclairer la prise de décisions et renforcer les pratiques. L’OOAS considère qu’il est particulièrement important d’encourager la contribution de partenaires solides qui peuvent cerner et aborder efficacement les priorités. Dans le cadre de cette stratégie, les fonctions essentielles de l’OOAS sont le leadership, le marketing, la communication stratégique, la préconisation de politiques, la coordination, le réseautage, le soutien à la mobilisation des ressources, le soutien à l’harmonisation et la création de partenariats. L’OOAS a délibérément adopté une approche fondée sur la réflexion et l’apprentissage par la pratique, ce qui lui permet d’examiner, d’évaluer et de tirer des enseignements de ses expériences afin d’améliorer constamment son action, ainsi que d’évoluer et de s’adapter à un contexte régional variable et exigeant.

Une recherche de qualité est un aspect fondamental de la prise de décisions fondée sur des données probantes. La promotion de la recherche comme outil pour résoudre les problèmes de santé dans les pays de la CÉDÉAO a joué un rôle de plus en plus important pour l’OOAS, comme le montrent ses trois plans stratégiques. Si le premier plan (en 2000) n’intégrait pas de programme fondé sur la recherche, au moment où le deuxième a été rédigé (2009-2013), la promotion de la recherche était devenue un programme axé sur le renforcement des systèmes nationaux de recherche en santé. Ce programme a été instauré en appliquant le cadre conceptuel établi par Pang et coll. [8], qui est basé sur le renforcement de quatre fonctions de recherche essentielles : la gestion collaborative, le financement de la recherche, la capacité de recherche individuelle et institutionnelle, et la promotion de la diffusion et de l’utilisation des résultats de recherche. À ces fonctions, l’OOAS a ajouté une fonction d’établissement de partenariats [9, 10]. L’analyse des interventions de l’OOAS pendant cette période a montré que l’action de cette organisation avait entraîné des modifications de la gouvernance, de la gestion et du financement de la recherche, ainsi que du renforcement des capacités individuelles et institutionnelles, de la diffusion de la recherche, de la collaboration et des échanges entre les différents intervenants. Elle a également révélé de nombreux défis, parmi lesquels l’appropriation des processus, l’engagement des pays membres, les faibles capacités institutionnelles et individuelles, la mobilisation et l’engagement des intervenants [10,11,12].

Afin de renforcer la prise de décisions fondée sur des données probantes, les données sur la santé et la recherche ont été intégrées au troisième plan stratégique (2016-2020), avec la création d’un programme unique visant à améliorer la production, la diffusion et l’utilisation des données et des résultats de la recherche pour la planification des programmes sanitaires, l’objectif à terme étant d’améliorer la santé des populations. À cette fin, il est devenu important d’élargir les partenariats à des intervenants très variés.

Si le point d’ancrage de l’OOAS à l’échelle du pays a toujours été le ministère de la Santé, en intégrant la recherche à son travail, l’organisation a également appris à jouer son rôle aux côtés d’autres partenaires et à travailler avec des réseaux de centres de recherche et d’universités.

Au niveau institutionnel, cette approche est mise en oeuvre conjointement par les unités responsables de la recherche, de la santé maternelle et infantile, du renforcement des systèmes de santé et de l’établissement de partenariats. En outre, elle est appliquée de concert avec les ministères de la Santé, les institutions de recherche, la société civile, les organisations non gouvernementales et les partenaires techniques, avec le soutien financier des partenaires internationaux et de la CÉDÉAO. Ces activités de promotion de la recherche sont également appuyées par de nombreux projets régionaux [13-16].

**Résultats préliminaires**

***Renforcement de l’engagement des intervenants***

Les initiatives de gestion collaborative de l’OOAS favorisent la détermination des besoins et priorités de recherche, et renforcent la collaboration entre les chercheurs, les décideurs politiques et les autres intervenants de la santé, ce qui favorise un climat de confiance et les partenariats. Grâce à ces activités, l’OOAS a aidé plusieurs pays à élaborer et adopter leurs documents stratégiques (politiques, plans et priorités), et a commencé à encourager un dialogue au sein des pays et des régions dans le cadre de six ateliers nationaux et d’un atelier régional en 2015. Parmi les participants, on comptait des décideurs, des chercheurs, des dirigeants d’organisations non gouvernementales, des acteurs de la société civile, des représentants des associations professionnelles de santé; des partenaires du développement et des gestionnaires de plateformes de transfert des connaissances. Durant ces ateliers, les discussions se sont concentrées sur les thèmes suivants : collaboration entre chercheurs et utilisateurs des résultats de recherche; inclusion des facteurs systémiques, d’équité et de sexospécificité; évaluation des compétences et des besoins des intervenants en matière de transfert des connaissances; et obstacles et éléments favorables à une prise de décisions fondée sur des données probantes [17,18]. L’enthousiasme des intervenants a révélé que ces plateformes collaboratives devaient être maintenues pour les raisons suivantes : cerner des priorités et besoins de recherche supplémentaires en matière de santé maternelle et infantile; mobiliser tous les acteurs et s’assurer de leur adhésion; déterminer collectivement des solutions validées par la recherche aux problèmes de santé maternelle et infantile. Cette collaboration devrait également persuader les décideurs politiques nationaux d’augmenter la demande de recherche et convaincre les chercheurs de prendre en compte les priorités et les besoins nationaux lorsqu’ils conçoivent leur projet de recherche.

***Renforcer la disponibilité des données probantes***

L’OOAS finance la recherche afin d’accroître la disponibilité des données probantes qui éclairent la prise de décisions dans le domaine de la santé maternelle et infantile. En 2014, l’organisation a financé un examen des indicateurs de santé maternelle et infantile et une analyse de l’exécution des programmes de santé reproductive de 15 pays de l’Afrique de l’Ouest [19]. Cet examen a montré entre autres que dans de nombreux pays, la mortalité maternelle et infantile n’avait pas suffisamment diminué pour que les objectifs du Millénaire pour le développement soient atteints d’ici 2015. En 2015, l’OOAS a financé trois autres examens portant respectivement sur les questions suivantes : élaboration de programmes de santé maternelle et infantile qui tiennent compte de la sexospécificité et de l’équité; facteurs systémiques qui facilitent ou limitent l’amélioration de la santé maternelle et infantile; et transfert des connaissances. Ces examens ont mis en lumière l’importance d’intégrer les actions suivantes aux programmes de santé maternelle et infantile : promotion de l’autonomisation des femmes; prise en compte des besoins de sous-groupes particuliers de femmes et de nourrissons; prise en compte des facteurs systémiques et contextuels; et renforcement du processus de transfert des connaissances afin d’appuyer l’utilisation des données probantes [20, 21, 22].

***Renforcement des capacités***

Afin de renforcer les capacités individuelles et institutionnelles, l’OOAS octroie des bourses de formation aux jeunes chercheurs et a mis en place un réseau d’établissements de recherche afin de soutenir le perfectionnement professionnel des chercheurs, ainsi que les échanges et la collaboration entre eux. L’OOAS participe également à un projet régional de renforcement des capacités sur la recherche sur les politiques et les systèmes de santé en Afrique de l’Ouest [15]. Un forum régional inclusif a été organisé en 2015 et en 2016 sous la direction de l’Université du Ghana : il a permis d’analyser les forces et les faiblesses de la recherche sur les politiques et de systèmes de santé en Afrique de l’Ouest, ainsi que de déterminer les mesures de renforcement des capacités pouvant être mises en place à l’échelle régionale [23]. À l’issue du forum, un projet régional quinquennal a été lancé en 2016 afin de renforcer la capacité de la région à diriger des recherches sur les politiques et les systèmes de santé, ainsi qu’à diffuser des résultats qui influenceront les politiques de santé maternelle et infantile [16]. L’OOAS contribuera au projet en offrant son leadership, ses moyens techniques et son réseau régional pour mobiliser et faire participer les différents intervenants nécessaires à la réussite du projet.

***Renforcement des plateformes de transfert des connaissances et de partenariat***

Afin de promouvoir la diffusion et l’utilisation des résultats de recherche, l’OOAS collabore avec des personnes et des établissements spécialisés dans la recherche sur la santé maternelle et infantile : l’École de santé publique de l’Université du Ghana, le Laboratoire d’Études et de Recherche sur les Dynamiques Sociales et le Développement Local (LASDEL), les institutions des Nations Unies (OMS, Fonds des Nations Unies pour la population, UNICEF) et l’Agence de Médecine Préventive (AMP), entre autres. Par exemple, l’AMP et l’OOAS ont créé des groupes consultatifs techniques sur l’immunisation au Sénégal, en Côte d’Ivoire, au Burkina Faso et au Nigéria [24, 25]. Les membres de ces groupes ont été formés à la recherche et à l’utilisation des données probantes, et sont donc compétents pour conseiller les autorités sanitaires chargées de la vaccination. En se fondant sur les données actuelles, ces groupes ont conseillé aux dirigeants des pays d’introduire de nouveaux vaccins et d’organiser des programmes de vaccination.

En 2015, l’OOAS a également lancé un forum régional annuel à Ouagadougou, au Burkina Faso, sur les bonnes pratiques au sein de la CÉDÉAO. L’objectif était d’appuyer la définition, la documentation, la mise en commun et la mise à l’échelle des bonnes pratiques en matière de santé avec le soutien de l’Agence internationale pour le développement des États-Unis (USAID), de l’Agence allemande de développement (GIZ) et de l’Agence canadienne de développement international (ACDI). Dans l’optique de déterminer et de documenter les bonnes pratiques, des ateliers nationaux de formation aux méthodes de documentation ont permis de former plus de 400 personnes, avec le soutien de partenaires techniques et financiers de 14 pays de la CÉDÉAO.

L’OOAS soutient également la mise en place d’une plateforme de transfert des connaissances permettant d’utiliser les connaissances pour mettre en oeuvre les politiques et les programmes de santé maternelle et individuelle.

**Relever les défis**

En commandant ces examens, l’OOAS espérait obtenir une base de données probantes qui lui permettrait de jouer son rôle de sensibilisation et de catalyseur, amenant ainsi efficacement l’ensemble des intervenants à adopter une approche des systèmes de santé tournée vers l’amélioration de la mortalité maternelle et infantile en Afrique de l’Ouest. À ce titre, au lieu d’adopter des modèles de santé importés (ou itinérants), les intervenants des pays devraient collaborer pour cerner les éléments systémiques et contextuels qui constituent des obstacles, puis déterminer des solutions qui soient adaptables et applicables localement. Cette vision nécessite la participation de l’ensemble des intervenants concernés, et plus important encore, leur collaboration aux échelles régionale et nationale. Pour concrétiser cette vision, il faudra relever plusieurs défis.

La réussite de la mise en oeuvre de cette approche est tout d’abord conditionnée par la coordination des acteurs au sein de l’OOAS elle-même. En effet, le fait que plusieurs unités puissent lancer leurs activités sans consulter les autres peut limiter l’engagement et la collaboration des acteurs institutionnels. Pour relever ce défi, la haute direction de l’OOAS a mis sur pied une unité de gestion de projet qui organise des séances de planification trimestrielles avec l’ensemble du personnel. Par conséquent, les programmes de l’institution ont créé une collaboration plus forte entre les unités, une culture institutionnelle de la surveillance et de l’évaluation, et la promotion de l’utilisation des données probantes pour les activités afin d’améliorer leur rendement.

Le deuxième défi est de persuader les pays de s’engager et de s’approprier cette approche étant donné les ressources humaines réduites, lesquelles possèdent une faible capacité et travaillent dans un environnement où le roulement de la direction est incessant. Lors d’une réunion régionale en 2011, les représentants responsables de la gestion de la recherche au sein des ministères de la santé ont indiqué qu’ils avaient besoin de renforcer leurs capacités en leadership, communication, en sensibilisation, en mobilisation des ressources, en réseautage, en gestion de recherche et en surveillance/évaluation [22]. L’OOAS et d’autres partenaires financiers ont soutenu la formation de ces gestionnaires, puis l’institution a poursuivi ce soutien en mettant en oeuvre leur nouveau plan stratégique et en menant plusieurs projets régionaux [13, 16]. Dans le domaine des services de recherche, les compétences en gestion de la recherche, en leadership, en sensibilisation, en communication et en réseautage devraient contribuer à améliorer la mobilisation et l’engagement de l’ensemble des intervenants. En outre, les plateformes encourageant la collaboration entre les chercheurs et les décideurs politiques garantiront que la recherche est créative, novatrice, multidisciplinaire, et guidée par des besoins et des possibilités actuels, l’objectif étant de produire des solutions se fondant sur le contexte, ainsi que des résultats qui sont utilisés et diffusés comme il convient [26]. Ici, les efforts déployés par l’OOAS pour mettre en place un comité consultatif régional composé de chercheurs expérimentés ont contribué à peaufiner les protocoles de contrôle de la qualité, à faire aboutir des projets de recherche et, plus important, à réunir les chercheurs et les utilisateurs potentiels de la recherche, notamment les décideurs. Ce type d’accompagnement devrait être reproduit.

L’importance de la recherche sur la lutte contre les maladies et du renforcement des systèmes de santé a été reconnue après l’épidémie récente de la maladie à virus Ebola [27], ce qui donne l’occasion à l’OOAS de persuader les décideurs politiques de s’engager davantage à renforcer la recherche, et plus particulièrement, de faire de la recherche un outil de soutien à la prise de décisions pour améliorer la santé en Afrique de l’Ouest. Pour relever ce défi, l’OOAS et l’ensemble de ses partenaires devront s’engager dans une collaboration efficace.

Malheureusement, la coordination et la collaboration des partenaires techniques et financiers dans les pays d’Afrique de l’Ouest constituent le troisième défi. En effet, la faible communication entre les différents acteurs limite leur connaissance des activités des autres et entraîne souvent des activités redondantes. L’OOAS s’emploie à faciliter cette coordination en invitant l’ensemble des autres intervenants à participer à la planification, à la mise en oeuvre et à l’évaluation de ses programmes. L’OOAS a créé le Forum des partenaires, espace de dialogue permanent entre les partenaires techniques et financiers, lors de l’Assemblée annuelle des ministres de la Santé. L’OOAS fait également la promotion d’un cadre de collaboration bilatérale thématique avec ses partenaires. Ce dialogue permanent entre l’OOAS et les autres partenaires permet également d’exercer une action de sensibilisation et de mobiliser des fonds pour les besoins et les priorités des pays, ce qui démontre que l’institution fait preuve de leadership.

**Conclusion**

Selon nous, les expériences de l’OOAS à ce jour montrent comment une institution de santé régionale peut intégrer la promotion de la recherche à la lutte contre la mortalité maternelle et infantile. Parallèlement, les défis que l’organisation a relevés révèlent également l’importance d’une certaine cohésion entre les acteurs promouvant cette initiative, l’importance du leadership et de l’engagement des acteurs des pays membres pilotant ce processus, ainsi que la nécessité d’une collaboration et d’une coordination entre l’ensemble des partenaires des pays membres et de la région. Pour atteindre les objectifs de développement durable en Afrique de l’Ouest, l’intégration nécessitera de la cohésion, une culture de la recherche et de la surveillance et de l’évaluation, ainsi que la participation de l’ensemble des partenaires techniques et financiers de la mise en oeuvre dont la mission est de promouvoir une recherche qui oeuvre pour l’amélioration de la santé. Ensuite, l’OOAS et l’ensemble des partenaires devraient s’employer à persuader les dirigeants des pays membres de piloter le processus de mobilisation de l’ensemble des intervenants : cette initiative créera un environnement favorable à la recherche, en bref, des systèmes nationaux de recherche en santé efficaces. Enfin, les expériences passées [10,11] montrent qu’il est nécessaire de mettre en place un système d’apprentissage et de surveillance et d’évaluation afin de tirer tous les enseignements nécessaires et de partager cette expérience avec l’ensemble de la communauté.

**Références**

1. Alkema L, Chou D, Hogan D, Zhang S, Moller AB, Gemmill A, Fat DM, Boerma T, Temmerman M, Mathers C, Say L, au nom du groupe consultatif technique et des collaborateurs du Groupe interorganismes pour l’estimation de la mortalité maternelle des Nations Unies. Global, regional, and national levels and trends in maternal mortality between 1990 and 2015, with scenario-based projections to 2030: a systematic analysis by the UN Maternal Mortality Estimation Inter-Agency Group. www.thelancet.com. Mis en ligne le 12 novembre 2015 http://dx.doi.org/10.1016/S0140-6736(15)00838-7
2. Agyepong IA, Kwamie A, Defor S, Frimpong E, Aryeetey GC, Ibrahim A. Health Systems and MNCH Outcomes in West Africa. A study of Conducive and limiting Health Systems factors to improving mother, new Born and child health in West Africa with a focus on Ghana, Benin, Burkina Faso, Mali, Nigeria and Senegal. Organisation ouest-africaine de la santé, Bobo-Dioulasso, 2015.
3. Ramaswamy R, Kallam B, Kopic D, Pujic B, Owen MD. Global health partnerships: building multinational collaborations to achieve lasting improvements in maternal and neonatal health. *Globalization and Health* 2016, 12:22.
4. Hoffman SJ, Røttingen JA, Bennett S, Lavis JN, Edge JS, Frenk J. A Review of Conceptual Barriers and Opportunities facing Health Systems Research to inform a Strategy from the World Health Organization. *Document d’information commandé par l’Alliance pour la recherche sur les politiques et les systèmes afin d’élaborer la Stratégie de recherche sur les systèmes de santé de l’OMS*. Alliance pour la recherche sur les politiques et systèmes de santé. OMS; 2012. Genève.
5. Lucy Gilson (dir.) (2012). Recherche sur les politiques et les systèmes de santé : Manuel de méthodologie. Version abrégée. Alliance pour la recherche sur les politiques et systèmes de santé. Organisation mondiale de la Santé. Genève.
6. Remme JHF, Adam T, Becerra-Posada F, D’Arcangues C, Devlin M, et coll. Defining Research to Improve Health Systems. PLoS Med 2010, 7(11): e1001000. doi:10.1371/journal.pmed.1001000
7. Implementation research for control of infectious diseases of poverty: strengthening the evidence base for the access and delivery of new and improved tools, strategies and interventions. Organisation mondiale de la Santé, Programme spécial de recherche et de formation concernant les maladies tropicales UNICEF/PNUD/Banque mondiale/OMS. 2002.
8. Pang T, Sadana R, Hanney S, Bhutta ZA, Hyder AA, Simon J. [Knowledge for better health: a conceptual framework and foundation for health research systems.](http://www.ncbi.nlm.nih.gov/pubmed/14758408) Bull World Health Organ. 2003;81(11):815-20.
9. Sombié I, Aidam J, Konaté B, Somé TD, Kambou SS. [The state of the research for health environment in the ministries of health of the Economic Community of the West African States (ECOWAS).](http://www.ncbi.nlm.nih.gov/pubmed/14758408) *Health Res Policy Syst.* 11 sept 2013; 11:35. doi: 10.1186/1478-4505-11-35.
10. Aidam J, Sombié I. [**The West African Health Organization’s experience in improving the health research environment in the ECOWAS region.**](http://www.ncbi.nlm.nih.gov/pubmed/27098359) *Health Res Policy Syst*. 20 avr. 2016; 14:30. doi: 10.1186/s12961-016-0102-7.
11. Sombie I, Aidam J, Montorzi G. Strengthening national health research system in the Post conflicts in West Africa.
12. Keita N, Lokossou V, Berthe A, Sombie I, Johnson E, Busia K. The West African experience to establish steering committees, improve collaboration between researchers and decision-makers and increase the use of health research.
13. Transformer en politiques les données probantes concernant la santé des mères, des nouveau-nés et des enfants en Afrique de l’Ouest. https://www.idrc.ca/fr/project/transformer-en-politiques-les-donnees-probantes-concernant-la-sante-des-meres-des-nouveau (consulté le 16/10/2016).
14. Strengthening National Health Research system in West Africa <http://www.cohred.org/westafrica/> (consulté le 16/10/2016).
15. Initiative ouest-africaine de renforcement des capacités au moyen de la recherche sur les systèmes de santé <https://www.idrc.ca/fr/project/initiative-ouest-africaine-de-renforcement-des-capacites-au-moyen-de-la-recherche-sur-les> (consulté le 16/10/2016).
16. Partenariat entre l’Afrique occidentale et l’Afrique centrale pour les politiques et les systèmes de santé et la santé de la mère, du nouveau-né, de l’enfant et de l’adolescent. Partenariat sud-sud de renforcement des capacités et de réseautage pour le leadership, la recherche et les pratiques appuyant les politiques et systèmes de santé visant à améliorer les résultats de santé de la mère, du nouveau-né, de l’enfant et de l’adolescent en Afrique occidentale et en Afrique centrale. 2016.
17. Uneke CJ, Sombie I, Keita K, Lokossou V, Johnson E, Ongolo-Zogo P. An Assessment of National Maternal and Child Health Policy-Makers’ Knowledge and Capacity for Evidence Informed Policy-Making in Nigeria. *International Journal of Health Policy and Management 2016,* 5(x), 1–8.
18. Uneke CJ, Sombie I, Keita N, Lokossou V, Johnson E, Ongolo-Zogo P. **Improving maternal and child health policymaking process in Nigeria: an assessment of policymakers’ needs, barriers and facilitators of evidence-informed policymaking.**
19. Organisation ouest-africaine de la santé. Analyse de la situation de la santé de la reproduction et la planification familiale dans l’espace CÉDÉAO. Mai 2014. Bobo-Dioulasso. 80 pages.
20. Larson EA. Inclusion of Gender and Equity in Maternal, Newborn and Child Health Services in West Africa: A Literature Review of Programming West African Health Organisation, Bobo-Dioulasso, 2015.
21. Agyepong IA, Kwamie A, Frimpong E, Defor S, Aryeetey GC, Abdallah I, Virgil Lokossou V, Sombie I. Conducive and limiting Health Systems factors to improving maternal, newborn and child health in West Africa. Bringing the worlds together: Health Systems, Context, Interventions and maternal, newborn and child health research and outcomes improvement in West Africa.
22. Ongolo-Zogo Pierre. Analyse de la situation sur le transfert de connaissance au profit de la santé maternelle et néonatale en Afrique de l’Ouest. OOAS, février 2016.
23. Defor S, Kwamie A, Sombie I. Renforcement de la capacité de recherche concernant les systèmes de politique en santé en Afrique de l’Ouest : leçons et expériences d’un effort de collaboration sous-régionale. Symposium #G1. 22^e^ Conférence canadienne sur la santé mondiale. Renforcement des capacités en santé mondiale : recherche et pratiques. Du 5 au 7 novembre 2015, Montréal, hôtel Bonaventure.
24. Adjagba A, Senouci K, Biellik R, Batmunkh N, Faye PC, Durupt A, Gessner BD, da Silva A. Aider les pays à créer et à renforcer leur GTCV : enseignements tirés de cinq années d’existence de SIVAC. Vaccine 2015; 33: 588–595
25. Les groupes techniques consultatifs de la vaccination. <http://www.nitag-resource.org/fr/qui-sommes-nous#nrc-map>. (Consulté le 03/09/2016.)
26. Sipido K, Degos L, Frackowiak R, Ganten D, Hofstraat H, Horvath I, Luyten F, Manns M, Oertel W, Zima T. Scientiﬁc Panel for Health: better research for better health. The Lancet 2016, 388: 865-866.
27. Massaquoi MBF, Kennedy SB, Tegli JK, Bolay FK, Kateh KN. Fostering collaboration on post-Ebola clinical research in Liberia. The Lancet 2016, 4: e239.
