# Supplementary material for: People and research: improved health systems for West Africans, by West Africans - report on special supplement
Source: BMC Proc. 2019 Feb 7;13(Suppl 1):1. doi: 10.1186/s12919-019-0162-0 (PMC6366023; doi:10.1186/s12919-019-0162-0)
Supplement: Supplementary file 3 — La qualité des services prénataux et d’accouchement gratuits au nord de la Sierra Leone, Koroma, M., Kamara, S., Bangura, E., Kamara, M., Lokossou, V., Keita, N. [file 12919_2019_162_MOESM3_ESM.docx]

***La qualité des services prénataux et d’accouchement gratuits au nord de la Sierra Leone***

**Manso M. Koroma^1*^, Samuel S. Kamara^1^, Evelyn A. Bangura^1^, Mohamed A. Kamara^1^, Virgil Lokossou^2^ et Namoudou Keita^2^**

^1^Département des sciences de l’environnement, Collège universitaire de Makeni, Université des sciences et de la technologie Ernest Bai Koroma, Makeni, Sierra Leone;

^2^Unité des soins de santé primaires et du renforcement des soins de santé, Organisation ouest-africaine de la santé, Bobo Dioulasso, Burkina Faso

**RÉSUMÉ**

**Contexte**

Le nombre de décès des mères en Afrique subsaharienne demeure extrêmement élevé. Parmi les pays d’Afrique de l’Ouest, la Sierra Leone est celui qui affiche le taux de mortalité maternelle le plus élevé. En 2010, les obstacles financiers ont été abolis afin d’amener plus de femmes à utiliser les services prénataux, d’accouchement et postnataux. Peu d’études ont été réalisées sur la qualité des services prénataux gratuits en Sierra Leone et sur l’accès à des soins obstétricaux d’urgence dans ce pays.

**Méthodologie**

En 2014, une enquête transversale a été menée dans 97 établissements de santé périphériques et trois hôpitaux du district de Bombali, au nord. Cent fournisseurs de soins prénataux et 486 femmes enceintes ont été interviewés, et 276 observations ont été faites. Nous avons évalué les services prénataux et d’accouchement fournis par rapport aux normes nationales. La distance entre chaque établissement donnant des services d’accouchement et le centre de soins obstétricaux d’urgence complets le plus proche a été calculée, et la proportion d’établissements d’une chefferie se trouvant dans un rayon de 15 km de chaque centre a aussi été établie. Enfin, une carte thématique a été élaborée pour illustrer les inégalités.

**Résultats**

Les services étaient de mauvaise qualité. D’après les normes nationales, seulement 27 % des femmes avaient été examinées, 2 % avaient passé des tests de dépistage à leur première visite prénatale, et 47 % avaient reçu les soins recommandés. Bien que 94 % des établissements donnaient des services d’accouchement, une minorité d’entre eux avaient une salle d’accouchement (40 %), des trousses d’accouchement (42 %) ou de l’eau potable (46 %). Des accoucheuses qualifiées avaient supervisé 35 % des accouchements, et les interventions ont été correctement documentées dans à peine 35 % de ces cas. Aucun des cinq centres de soins obstétricaux d’urgence de base ne respectait les normes nationales, et les régions du centre et de l’extrême nord du district étaient celles où l’accès à des soins obstétricaux d’urgence complets était le plus limité.

**Conclusion**

Le secteur de la santé doit surveiller la qualité des interventions prénatales, en plus de mesurer la portée de celles-ci. La qualité des services d’accouchement est compromise par la piètre qualité des infrastructures, le manque de qualifications du personnel, la pénurie de matériel, le mauvais fonctionnement des centres de soins obstétricaux d’urgence de base et les inégalités géographiques dans l’accès aux centres de soins obstétricaux d’urgence complets. Ces conclusions laissent entendre que le secteur de la santé doit sans tarder enquêter sur les inégalités qui perdurent et qui nuisent à l’utilisation des services, et explorer des mécanismes de financement plus durables. Autrement, le pays risque de ne pas atteindre son objectif consistant à réduire le nombre de décès des mères.

**Introduction**

L’Afrique subsaharienne est la région du monde où le nombre de décès des mères diminue le moins vite; ce nombre s’établissait en moyenne à 2,3 % par année entre 1990 et 2015. La région compte pour 66 % de tous les décès des mères dans le monde [1]. En 2015, les 18 pays affichant le taux de mortalité maternelle le plus élevé étaient situés en Afrique.

La Sierra Leone arrive en tête : le taux de mortalité maternelle y est estimé à 1 360 sur 100 000 (intervalle d’incertitude 999-1908) [1]. Ce taux est semblable à un taux de mortalité maternelle naturel estimé à environ 1 000 ou 1 500 sur 100 000, observé dans des situations où aucune mesure n’est prise pour éviter les décès des mères [2]. En Sierra Leone, les femmes enceintes sont plus à risque de mourir (environ 1 sur 17) que les femmes enceintes dans les pays à revenu élevé (1 sur 3 300) [1].

Dans les pays de l’Afrique subsaharienne, cette absence de progrès relativement à la réduction du nombre de décès des mères témoigne des inégalités sociales qui sont perpétuées par les structures sociales et les institutions officielles, divers facteurs socio-économiques, ainsi que des pratiques sociales et culturelles (figure 1). Ces inégalités structurelles influencent la mesure dans laquelle les femmes issues de différentes classes sociales ont facilement accès, et utilisent, des services de santé d’un niveau de qualité correct [3, 4].

**Déterminants structurels des inégalités en matière de santé**

**Gouvernance et politiques**

- Éducation
- Finances et infrastructure de la santé
- Protection sociale
- Lois (égalité entre les sexes, anti-violence, etc.)
- Santé et droits en matière de reproduction

**Valeurs culturelles et sociales**

- Condition de la femme
- Normes sexospécifiques
- Religion
- Croyances sur la santé
- Cohésion sociale

**Déterminants intermédiaires en matière de santé**

**Services de santé**

- Disponibilité des services pertinents (soins prénataux, prestation des services par du personnel compétent, renvois pour des soins obstétricaux d’urgence)
- Compétences générales et techniques du personnel
- Acceptabilité pour la collectivité
- Frais et coûts connexes

**Contexte communautaire**

- Milieu rural/urbain
- Position sociale (classe, richesse, ethnicité)
- Connaissance de la disponibilité des soins
- Perceptions de la qualité des soins
- Éloignement des établissements
- Capital social

**Influence de la famille et des pairs**

- Structure familiale et pouvoir décisionnel
- Relations conjugales
- Communication entre les conjoints
- Revenu
- Accès aux ressources
- Réseaux de soutien

**Attributs individuels**

- Âge
- Nombre d’enfants
- Connaissances
- Auto-efficacité

RÉSULTATS EN MATIÈRE DE SANTÉ MATERNELLE

**Figure 1 :** Les déterminants sociaux de la santé maternelle. Adapté du document de l’OMS (2011) Closing the Gap: Policy into Practise on Social Determinants of Health (3).

Les femmes qui sont désavantagées sur les plans social ou économique et qui sont isolées sur le plan géographique sont plus à risque de mourir pendant une grossesse, même si la plupart de ces décès pourraient être évités si ces femmes recevaient les soins nécessaires à temps [2, 5]. Par exemple, les femmes les plus pauvres des pays de l’Afrique subsaharienne n’ont pas accès en temps opportun à des accoucheuses qualifiées ni à des soins en établissement, contrairement aux femmes dans les pays riches, où les compétences permettant d’utiliser des appareils pouvant aider à sauver des vies et d’effectuer des procédures d’urgence (césarienne, transfusion sanguine et antibiotiques efficaces) sont largement répandues [6]. Dans les situations où les inégalités sont moindres, le nombre de décès maternels est moins élevé. Après que le Rwanda a eu élargi l’accès à l’accouchement en établissement entre 1990 et 2015, la proportion de femmes ayant accouché avec l’aide d’accoucheuses qualifiées a plus que triplée, passant de 25,8 % à 90,7 %, et le taux de mortalité maternelle a chuté de 78 % au cours de la même période [1, 7, 8].

Outre les déterminants associés au système de santé, le revenu du ménage est l’un des facteurs liés à la mortalité maternelle [9]. Dans les pays à faible revenu, les femmes pauvres ne sont pas prêtes à recourir aux services du secteur de la santé si elles doivent payer pour les obtenir [10]. C’est un point important en cela que les pays qui sont parvenus à réduire le nombre de décès des mères avaient également réduit les coûts d’accès à des soins professionnels [5, 6]. Des données indiquent qu’il y a eu une hausse des naissances en établissement et une baisse du nombre de décès de nouveau-nés à la suite de l’élimination des frais [10, 11]. Devant des éléments de preuve aussi probants, les gouvernements de pays en développement ont été pressés de mettre en place des exemptions du paiement des services de santé destinés aux mères en guise de stratégie pour réduire les décès chez ces dernières. Jusqu’à maintenant, 15 pays de l’Afrique subsaharienne ont aboli les frais [10].

En 2010, le gouvernement de la Sierra Leone a lancé l’initiative sur la gratuité des soins de santé [12] (l’« initiative »), dans le cadre de laquelle les femmes enceintes, les mères qui allaitent et les enfants de cinq ans ou moins étaient dispensés du paiement des frais. Le cadre conceptuel à la figure 2 ci-dessous montre comment l’initiative devrait mener à une baisse des décès de mères dans les établissements de santé.

L’hypothèse est que si plus de femmes enceintes utilisent les services gratuits qui leur sont destinés et que la qualité de ces services est améliorée, puis maintenue par un effectif compétent et motivé, alors les résultats en matière de santé maternelle seront meilleurs et le taux de mortalité chez les mères chutera. Dès le départ, l’initiative a fait l’objet d’examens, et des études ont été menées pour évaluer son efficacité. Toutefois, ces études étaient principalement de nature qualitative, visaient relativement peu d’établissements de santé ou portaient sur des aspects précis de l’initiative, et n’ont pas permis d’obtenir des données décentralisées pertinentes et d’établir ainsi l’efficacité de cette dernière [13, 14, 15, 16].

La présente étude, plus étoffée, visait à recueillir plus de données afin d’étayer la prise de décisions à l’échelle infranationale, un aspect important compte tenu des réformes décentralisatrices en cours au pays. Il s’agissait en particulier d’enquêter sur la qualité des services prénataux et d’accouchement fournis dans les établissements de santé du district de Bombali, au nord de la Sierra Leone, où l’initiative avait été mise en place et de trouver des solutions pour faire tomber les obstacles qui minent l’efficacité de l’initiative dans les districts.

**Soins de santé offerts gratuitement aux femmes enceintes**

Augmentation du recours aux composantes du Basic Package of Essential Health Services (BPEHS) liées à la santé maternelle dans les établissements de santé

**Augmentation du financement en santé pour les établissements de santé**

Médicaments, équipement et fournitures en quantité suffisante

Augmentation de la qualité des services fournis

**Augmentation du nombre de travailleurs en santé formés et motivés**

Renforcement de la surveillance pour maintenir la qualité des soins

**Normes de services établies, documentés et surveillées**

**Diminution du taux de mortalité maternelle**

Figure 2 : Modèle conceptuel de la Free Health Care Initiative (FHCI) dans les établissements de santé

**Méthodologie**

**Méthodologie et population**

L’étude transversale sur les établissements de santé a été menée de mars à avril 2014 dans le district de Bombali, l’un des cinq districts du nord de la Sierra Leone. Elle visait l’ensemble des établissements de santé publics et missionnaires, soit 100 établissements, dont trois hôpitaux de district, 94 unités de santé publiques en périphérie et trois établissements à but non lucratif. L’équipe a interviewé les agents responsables le jour de l’étude et observé deux ou trois fournisseurs de soins prénataux au travail dans chacune des 97 unités de santé en périphérie. Elle a rencontré en personne 486 femmes enceintes qui utilisaient les services prénataux donnés dans les établissements de santé ce jour-là. Les rencontres ont eu lieu juste après que les femmes ont eu obtenu les services prénataux.

**Collecte des données**

Nous avons adapté, et mis à l’essai sur le terrain les questionnaires de la maternité sans risque de l’Organisation mondiale de la Santé, ainsi que la grille d’observation des soins prénataux élaborée par l’United States Agency for International Development, dans le cadre du Maternal and Child Health Integrated Programme [17, 18]. Nous avons recueilli des données sur les variables suivantes : le nombre de lits que les établissements réservent aux femmes enceintes; l’infrastructure de la clinique, l’équipement, les médicaments et le matériel qu’on y trouve; les services prénataux et d’accouchement; les complications survenues; les services de laboratoire; les services obstétricaux d’urgence et les aiguillages; le matériel pédagogique et la documentation sur la planification familiale disponibles à la clinique. Nous avons observé et évalué les pratiques des cliniques de soins prénataux à l’aide de la grille d’observation des soins prénataux.

Lors des entrevues menées à la sortie de la clinique, nous avons demandé aux femmes quel âge elles avaient, comment elles s’étaient rendues à la clinique, quel était le coût des services, quels étaient leurs antécédents en matière de maternité et d’accouchement, quels services et conseils elles avaient reçus à la clinique, et ce qu’elles savaient des signes de danger liés à la grossesse. Nous avons examiné rétroactivement plusieurs dossiers, des fiches de soins prénataux, des registres d’accouchement ainsi que des dossiers concernant les accouchements difficiles et les accouchements normaux. Nous avons également recueilli des données sur les mères et les nouveau-nés, notamment sur la méthode d’accouchement, les naissances vivantes, les indices d’Apgar, le poids à la naissance, les morts nés macérés ou non, les morts néonatales immédiates, la santé des mères et les aiguillages.

**Analyse des données**

Les données quantitatives ont été analysées à l’aide du logiciel SPSS. Les mesures sommaires sont des proportions calculées relativement aux variables quantitatives. Des intervalles de confiance ont été établis à l’égard des proportions. Les normes nationales [19] en matière d’interventions prénatales faites dans les unités de santé en périphérie à l’occasion de cliniques de soins prénataux ont été adaptées et utilisées pour évaluer les services prénataux offerts aux femmes dans trois domaines. Il a été jugé qu’une femme avait subi un examen adéquat si, lors de sa première visite prénatale, les six signes physiques recommandés avaient été examinés. Si un nombre inférieur de signes avait été examiné, alors il a été jugé que l’examen n’était pas adéquat. Selon la grille d’évaluation, les femmes qui ont passé les quatre tests de dépistage de base lors de leur première visite prénatale ont été adéquatement évaluées. À l’inverse, si elles ont passé moins de tests, l’examen a été considéré comme étant inadéquat. Les interventions ont été jugées adéquates si les femmes en ont eu trois lors de leur troisième trimestre. Le niveau de soins a été jugé inadéquat s’il y a eu moins d’interventions.

La distance entre chaque établissement donnant des services d’accouchement et le centre de soins obstétricaux d’urgence complets le plus proche a été calculée, et la proportion d’établissements d’une chefferie se trouvant dans un rayon de 15 km de chaque centre a aussi été établie. Une carte thématique a été élaborée pour illustrer les inégalités quant à la prestation de soins obstétricaux d’urgence complets.

L’étude a été approuvée par le comité d’éthique et d’examen de la recherche national du ministère de la Santé et de l’Hygiène. Les femmes enceintes ayant participé à l’étude ont donné leur consentement éclairé.

**Contraintes de l’étude**

Il a été difficile de recueillir des données qualitatives en raison de l’éclosion de la maladie à virus Ebola survenue en mai 2014. Des séances de groupes de discussions prévues ont été annulées à cause de l’entrée en vigueur du règlement sanitaire d’urgence, lequel règlement limitait les déplacements des gens. Par ailleurs, la présente étude ne consistait pas à recueillir des données sur les déterminants intermédiaires et structuraux des inégalités en matière de santé qui ont des répercussions sur les mères.

**Résultats**

L’accès physique aux services prénataux et d’accouchement était répandu. Sur les 97 établissements publics visés par l’étude, 95 % donnaient quotidiennement des services prénataux, 94 % assuraient des services d'accouchement et 86 % fournissaient des services postnataux. Parmi ces établissements, 21 % étaient des centres de santé communautaires, 38 % étaient des postes sanitaires communautaires et 40 % étaient des postes sanitaires pour les mères et les enfants. Sur les 486 femmes interrogées, 93 % avaient reçu des soins gratuits lors de leur dernière grossesse. La qualité des services reçus variait (figure 3). Selon les normes nationales, seulement 27 % des femmes avaient été examinées, 2 % avaient passé des tests de dépistage, et 47 % avaient reçu les soins recommandés.

Protéine urinaire

Hémoglobine

Syphilis

Consultation et dépistage volontaire pour le VIH

Pourcentage

Figure 3 : Pourcentage de femmes recevant des niveaux de services prénataux adéquats ou inadéquats dans les établissements de santé

Moins du tiers des femmes ont passé les tests visant à connaître le taux d’hémoglobine dans le sang, à vérifier la présence d’albumine dans l’urine et à dépister une éventuelle syphilis (de 10 % à 28 %), tandis que 50 % des femmes ont passé un test de dépistage du VIH (tableau 1). Nous avons observé que la plupart des fournisseurs de soins de santé prénataux (plus de 80 %) pesaient les femmes, prenaient la pression artérielle de ces dernières, palpaient leur abdomen et écoutaient le coeur du foetus. Ils étaient toutefois moins nombreux à vérifier les signes d’oedème (45 %) et d’anémie (52 %) (tableau 1).

**Tableau 1 : Pourcentage des femmes qui, lors de leur première visite, ont passé des tests de dépistage et été examinées pour déceler différents signes physiques**

|  | **Femmes enceintes ayant été examinées pendant leur troisième trimestre**  % (n=58) | **IC à 95 %** |
| --- | --- | --- |
| **Test de dépistage** |  |  |
| Protéine urinaire | 10 | 2,5 – 18,2 |
| Syphilis | 22 | 11,7 – 33,2 |
| Hémoglobine | 28 | 16,1 – 39,1 |
| Consultation et dépistage volontaire pour le VIH | 50 | 37,1 – 62,9 |
|  |  |  |
| **Signe physique** |  |  |
| Oedème | 45 | 32,0 – 57,6 |
| Anémie | 52 | 38,9 – 64,6 |
| Coeur du foetus | 76 | 64,9 – 86,9 |
| Poids | 79 | 68,9 – 89,7 |
| Palpation de l’abdomen | 79 | 68,9 – 89,7 |
| Pression artérielle | 83 | 73,0 – 92,5 |

La prophylaxie au fer était l’intervention la plus courante, tandis que 65 % des femmes se souvenaient avoir reçu des conseils pertinents sur les quatre sujets liés à la préparation à l’accouchement (tableau 2).

**Tableau 2 : Pourcentage des femmes qui, lors de leur visite au troisième trimestre, ont reçu des soins prénataux**

| **Intervention** | **Femmes enceintes ayant été examinées pendant leur troisième trimestre**  % (n=244) | **IC à 95 %** |
| --- | --- | --- |
| Préparation à l’accouchement (IEC) | 65 | 58,8 – 70,8 |
| Fansidar/sulfadoxine-pyriméthamine pour le TPI | 89 | 85,5 – 93,2 |
| Fer/acide folique | 98 | 96,1 – 99,7 |

Certains médicaments prénataux essentiels, du matériel et de l’équipement nécessaire aux évaluations prénatales étaient en rupture de stock. La pénurie était la plus sévère du côté du matériel de laboratoire (de 86 % à 97 %) comparativement à la pénurie d’équipement (de 13,3 % à 47,4 %) ou de médicaments (12,4 %) (tableau 3).

**Tableau 3 : Pourcentage des établissements de santé en rupture de stock d’équipement, de médicaments et de matériel destinés aux soins prénataux**

| **Article** | **Établissements (n)** | **%** | **IC à 95 %** |
| --- | --- | --- | --- |
| Trousse de dépistage de la syphilis | 95 | 96,8 | 92,5 – 100 |
| Trousse d’analyse des urines | 93 | 86 | 78,5 – 92,5 |
| Tensiomètre | 97 | 47,4 | 37,1 – 57,7 |
| Stéthoscope obstétrical | 97 | 15,5 | 8,3 – 22,7 |
| Partogramme | 90 | 13,3 | 6,7 – 21,1 |
| Capsules de fer | 89 | 12,4 | 5,6 – 19,1 |

Même si seulement 62 % des 244 femmes ayant reçu des soins prénataux se souvenaient avoir reçu des conseils sur les quatre sujets, la proportion de femmes se souvenant avoir été conseillées sur des sujets en particulier était élevée (tableau 4).

**Tableau 4 : Pourcentage des femmes qui, lors de leur visite au troisième trimestre, ont reçu des conseils sur la préparation à l’accouchement**

| **Sujets abordés** | **Femmes enceintes ayant été examinées pendant leur troisième trimestre**  (n=244) | |
| --- | --- | --- |
|  | % | IC à 95 % |
| Lieu de l’accouchement | 95 | 92 – 97 |
| Quoi faire si un problème survient lors de l’accouchement | 87 | 83 – 91 |
| Avantages d’accoucher dans un établissement de santé | 82 | 77 – 86 |
| Comment se rendre à un établissement de santé en cas d’urgence | 82 | 76 – 86 |

Certains établissements où l’on pratique des accouchements n’avaient pas l’infrastructure, l’équipement, les médicaments et le matériel essentiels. Sur les 97 établissements visés par l’étude, 60 % n’avaient pas de salle d’accouchement, 54 % n’avaient pas d’eau potable, 58 % n’avaient pas l’équipement minimum requis pour pratiquer des accouchements, et 79 % n’avaient pas de table ou de tabouret pour effectuer des examens gynécologiques.

En ce qui concerne les interventions faites pendant le travail, elles n’étaient consignées correctement que dans 35 % des dossiers et à peine plus du tiers des femmes ont pu compter sur une accoucheuse qualifiée (sages-femmes, médecins, infirmières) (tableau 5).

**Tableau 5 : Pourcentage des naissances avec l’assistance d’un professionnel de la santé qualifié**

| **Cadre** | **Femmes qui ont accouché de leur dernier enfant dans un établissement de santé (n=340)** | |
| --- | --- | --- |
|  | **%** | IC à 95 % |
| Infirmière ou sage-femme | 35,6 | 30,6 – 40,6 |
| Médecin ou responsable de clinique | 1,8 | 0,6 – 3,2 |
| Employé du CSME | 56,5 | 51,2 – 61,8 |
| Accoucheuse traditionnelle | 7,4 | 4,7 – 10,3 |
| Membre de la famille ou autre | 5,9 | 3,5 – 8,5 |

Parmi les 97 établissements à l’étude, cinq ont été considérés comme étant des centres de soins obstétricaux d’urgence de base. Aux termes des normes nationales, de tels établissements doivent avoir en stock des médicaments d’importance vitale et compter sur du personnel capable d’effectuer des interventions permettant de sauver des vies, par exemple la réanimation néonatale. Aucun des cinq centres de soins obstétricaux d’urgence de base ne respectait entièrement les normes nationales (tableau 6) ni ne comptait sur du personnel qualifié, en mesure de traiter les urgences courantes dans le district (infection, hémorragie, rétention du placenta, prééclampsie et éclampsie, anémie sévère, présentation du siège).

**Tableau 6 : Établissements de soins obstétricaux d’urgence de base qui satisfaisaient aux normes nationales**

Ne satisfait pas aux normes

Satisfait aux normes

|  | Kamabai | Kalangba | Batkanu | Kagbere | Binkolo |
| --- | --- | --- | --- | --- | --- |
| Laboratoire fonctionnel |  |  |  |  |  |
| Eau |  |  |  |  |  |
| Système d’aiguillage |  |  |  |  |  |
| Médicaments et matériel |  |  |  |  |  |
| Équipement |  |  |  |  |  |
| Électricité |  |  |  |  |  |

Même si les trois hôpitaux de district correspondaient aux critères d’un centre de soins obstétricaux d’urgence complets, l’accès aux services n’était pas équitable. Une analyse géographique a montré que c’est dans les secteurs du centre et de l’extrême nord du district que l’accès aux services était le plus restreint (figure 4).


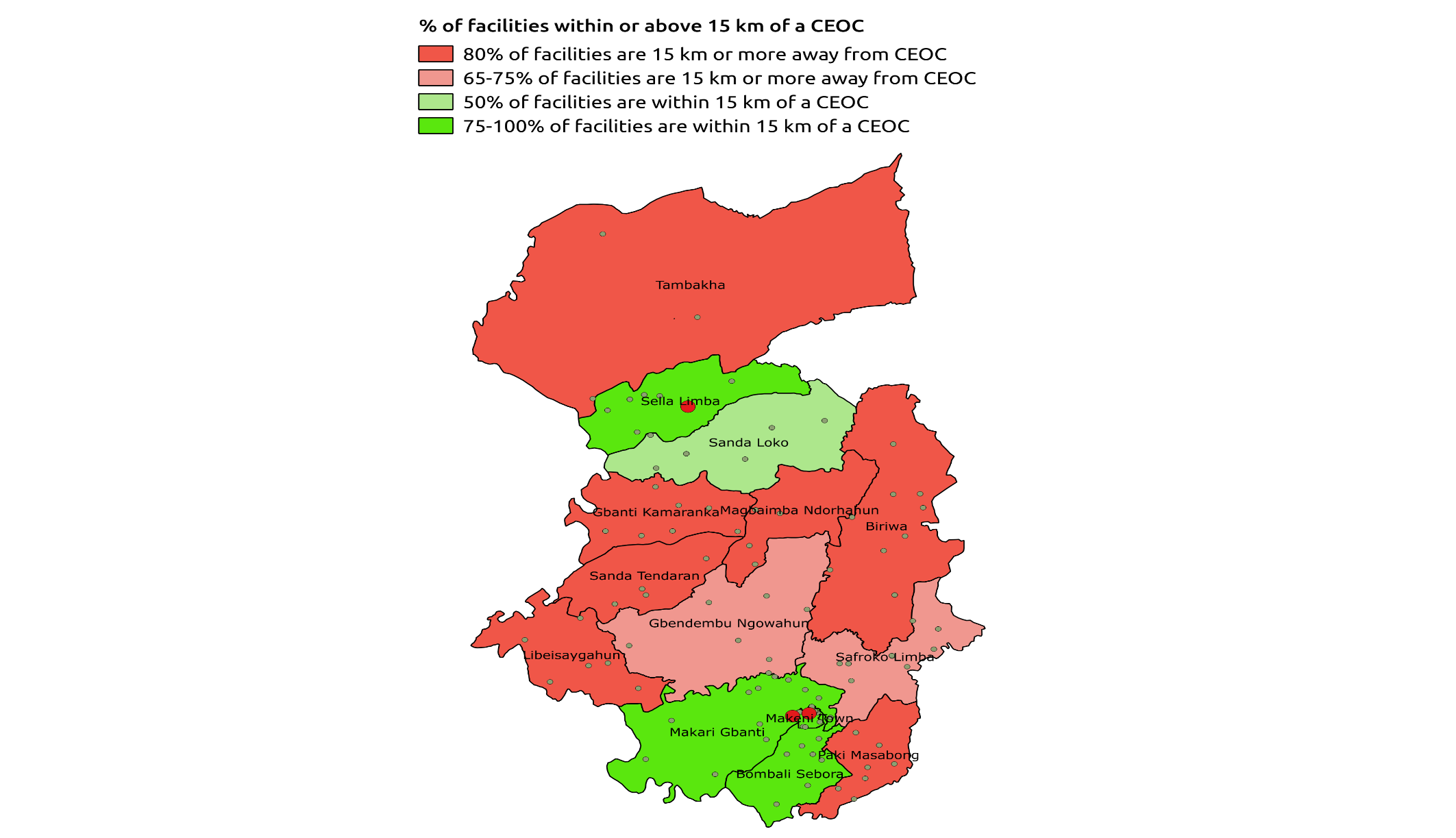


80 % des établissements se trouvent à 15 km ou plus d’un CSOU

De 65 à 75 % des établissements se trouvent à 15 km ou plus d’un CSOU

50 % des établissements se trouvent à 15 km ou plus d’un CSOU

De 75 à 100 % des établissements se trouvent à 15 km ou plus d’un CSOU

**% des établissements se trouvant à 15 km ou plus d’un centre de services obstétriques d’urgence (CSOU)**

**Figure 4 :** Pourcentage d’établissements dans un rayon de 15 km autour d’un centre de soins obstétricaux d’urgence complets

**Discussion**

Les services aux mères gratuits font partie de la stratégie pour améliorer la santé des mères dans les pays de l’Afrique subsaharienne (20). Cependant, malgré la volonté des femmes interrogées à utiliser les services de santé gratuits au nord de la Sierra Leone, plusieurs contraintes les en empêchent de sorte que les services offerts ne respectent pas les normes, en particulier en ce qui concerne l’accouchement.

Des données montrent que la gratuité des services a d’abord amené un plus grand nombre de femmes des sous-groupes à obtenir des soins prénataux. En effet, après la mise en place de la gratuité des services en Sierra Leone, la proportion de femmes ayant fait au moins quatre visites prénatales dans un établissement de santé est passée de 56 % en 2008 à 75 % en 2010, et à 76 % en 2013 [21, 22, 23]. Même si la hausse est observable dans tous les sous-groupes de femmes, l’augmentation la plus notable, entre 2008 et 2013, a eu lieu dans des sous-groupes auparavant désavantagés : les femmes n’ayant pas fait d’études, celles habitant en milieu rural, celles dont le revenu se trouve dans le quintile inférieur, et celles des régions où le pourcentage de femmes ayant fait au moins quatre visites prénatales était le plus bas en 2008 [24].

À l’instar des femmes d’autres pays africains, les femmes de la Sierra Leone reçoivent malgré tout des services inadéquats, comme nous le décrivons dans la présente étude [25, 26, 27]. C’est préoccupant parce que les femmes n’obtiennent pas les soins ou les conseils qui leur permettraient d’améliorer leur santé, sont mal informées des complications possibles et de ce qu’il faut faire pour se préparer à l’accouchement, et ne passent pas les tests permettant de détecter des problèmes qui pourraient affecter leur nouveau-né [6, 28]. Découragées par la piètre qualité des soins, certaines femmes se détourneront du secteur de la santé officiel et verront diminuer leur chance d’obtenir des soins professionnels pendant leur grossesse, période où elles sont pourtant le plus à risque et où le taux de mortalité est le plus élevé [29]. Les conclusions de l’étude montrent que le secteur de la santé doit surveiller à la fois la portée et la qualité des services donnés aux femmes. Il est toutefois plus difficile de déterminer quels indicateurs il faut mesurer pour évaluer la qualité des services prénataux, compte tenu de l’absence d’un cadre agréé dans les pays ayant peu de ressources [26]. Il faut donc poursuivre le travail afin de définir un ensemble d’indicateurs relatifs aux régions pauvres en ressources, car l’absence de consensus nous empêche de suivre les tendances et de comparer directement les résultats de l’étude à différentes mesures de la qualité des soins prénataux.

Le problème est moindre dans le cas des soins obstétricaux d’urgence, du fait qu’il existe des normes internationales à ce sujet. Nous avons constaté qu’il y avait 8 établissements de soins obstétricaux d’urgence pour 500 000 personnes, ce qui est supérieur à la norme internationale, selon laquelle il doit y avoir au moins 5 établissements de soins obstétricaux d’urgence, dont un centre de soins complets, pour 500 000 personnes. Cependant, la qualité générale des soins obstétricaux d’urgence était faible. Selon des études menées en 2011 et en 2012 dans des établissements de santé de la Sierra Leone, un peu moins du tiers des établissements répondait à la définition d’un centre de soins obstétricaux d’urgence de base [30, 31]. Si les femmes n’ont pas accès à des soins d’urgence lorsque des complications surviennent, il est peu probable que le nombre de décès évitables chez les mères diminue [32]. En d’autres mots, la seule existence d’établissements ne suffit pas; il faut aussi penser à l’accès à ces établissements et au fonctionnement de ceux-ci. Des outils d’analyse spatiale permettent de cibler les régions les moins bien desservies [33, 34].

Tant pour les soins d’urgence que pour les services prénataux, on pourrait soutenir que la qualité des services dont la prestation dépend de facteurs externes comme l’approvisionnement en équipement, en matériel de laboratoire et en médicaments se détériorerait si ces articles étaient régulièrement en rupture de stock, comme nous l’indiquions plus haut [30, 31]. Cet aspect risque moins de nuire à la qualité des soins de santé primaires dont le financement vient de sources externes et où l’approvisionnement est plus constant. Voilà qui explique peut-être les écarts constatés dans l’étude entre le grand nombre de femmes ayant fait le test de dépistage du VIH dans le cadre d’un programme national financé par des bailleurs de fonds, et le nombre de femmes ayant passé des tests pour dépister d’autres maladies dans le cadre de programmes financés par le secteur de la santé.

Nous avons été surpris de constater le piètre rendement des fournisseurs de soins dans les cas où l’approvisionnement n’était même pas un facteur. Dans la présente étude, nous avons constaté que les fournisseurs de soins prénataux ne soumettaient pas systématiquement les femmes à un examen complet pour détecter les signes physiques importants, à différents stades de leur grossesse, pas plus qu’ils ne donnaient de conseils sur l’ensemble des sujets pertinents, comme les conseils et le dépistage volontaire pour éviter la transmission du VIH/sida de la mère à l’enfant, ainsi que la préparation à l’accouchement. Le fait qu’aucune donnée n’a été recueillie pour comprendre pourquoi il en est ainsi est d’ailleurs une limite de la présente étude. Le faible moral des employés attribuable à l’augmentation de leur charge de travail est peut-être l’une des explications possibles. En effet, le nombre de fournisseurs donnant des services gratuits aux mères n’a pas augmenté proportionnellement à la demande croissante en matière de services d’accouchement et de soins d’urgence [35, 36]. Cette situation a nui à la qualité des services aux mères [35].

Selon certains, il faudrait embaucher des employés supplémentaires et rémunérer le personnel correctement afin de répondre à la demande croissante à laquelle fait face le secteur de la santé. Il faut néanmoins souligner que les intervenants prennent de plus en plus conscience que le secteur de la santé doit responsabiliser les fournisseurs de soins à l’égard des actions qui perpétuent la mortalité chez les mères [8]. Des voix se font entendre pour demander la mise en place de systèmes où il existe une culture de la qualité des soins et où la qualité découle d’un système de contrôle et de responsabilisation des professionnels, fondé sur des mécanismes, de manière à amener de plus en plus de gens à exiger des soins de qualité, des vérifications, des enquêtes confidentielles et des programmes de mentorat [6, 8, 36]. En mettant en place de telles politiques, et en les appliquant, il est possible, comme on l’a vu au Rwanda, de faire baisser considérablement le nombre de décès de mères en Afrique [8]. Cependant, même si une meilleure gouvernance en matière de santé contribue réellement à l’amélioration de la santé des mères, les inégalités socio-économiques persistantes continuent de freiner les efforts [37]. Parmi les autres limites de la présente étude, mentionnons le fait que nous n’avons pas examiné, ni analysé les données sur les déterminants sociaux de la santé des mères et la manière dont ils se répercutent sur le recours, par exemple, aux services aux mères gratuits. Il faut mieux comprendre pourquoi et comment les inégalités structurelles dans les pays où les taux de mortalité chez les mères sont élevés continuent de nuire aux efforts de réduire ces taux.

Si on offre aux mères des services de piètre qualité, elles seront moins enclines à utiliser ces services. C’est d’ailleurs observable dans certains pays de l’Afrique subsaharienne [38]. De même, les écarts ciblés quant à la qualité ne pourront être rattrapés que si les gouvernements de pays à faible revenu trouvent et maintiennent du financement à long terme pour investir dans les infrastructures et l’équipement, pour assurer un approvisionnement constant en médicaments et en matériel, et pour payer les salaires d’un effectif croissant [39]. Offrir gratuitement des services aux mères coûte cher. Le gouvernement de la Sierra Leone et ses partenaires se sont engagés à verser 91 millions de dollars américains quand l’initiative sur la gratuité des soins de santé a été lancée en 2010; le manque à gagner est de 20 millions de dollars américains [40]. Les dépenses gouvernementales dans le secteur de la santé par rapport aux dépenses nationales totales sont passées de 12 % en 2010 à 9,8 % en 2014, ce qui est inférieur à la cible de 15 % fixée à Abuja [41]. Pour les pays pauvres comme la Sierra Leone peinant à financer leur secteur de la santé, le défi est de déterminer comment et par qui seront financés les investissements qu’il faut faire dès maintenant pour améliorer la qualité des services gratuits aux mères. D’autres études doivent être menées pour enquêter sur les inégalités structurelles qui perpétuent la mortalité maternelle, et pour examiner des stratégies visant à financer de manière durable les services gratuits aux mères et à assurer un niveau de qualité adéquat. Autrement, les gains réalisés jusqu’à maintenant pourraient être annulés et le nombre de décès des mères déjà inacceptables pourrait recommencer à augmenter.

**Conclusion**

Même si les soins prénataux sont répandus dans les régions à l’étude, nous avons constaté qu’ils ne respectaient pas les normes. Les conclusions viennent confirmer la nécessité de surveiller constamment la portée des services offerts aux femmes, mais aussi la qualité de ces services. Même si le manque de fournitures venant de l’externe est en partie responsable de la piètre qualité tant des services prénataux que des services d’accouchement, cette situation n’explique pas les problèmes de rendement observés chez les travailleurs de la santé. Des évaluations qualitatives plus approfondies doivent être faites pour qu’on sache comment accroître la responsabilisation professionnelle à l’égard de la prestation des services de santé en général, et des services aux mères en particulier. Il est urgent que le secteur de la santé investisse pour que davantage de ressources examinent comment financer de façon durable la gratuité des services aux mères et offrir un niveau de qualité adéquat, et qu’elles enquêtent sur les inégalités persistantes qui empêchent les femmes d’accéder à ces services, dans le but ultime de réduire le nombre de décès chez les mères.

**Déclaration**

**Intérêts conflictuels**

Les auteurs déclarent n’avoir pas d’intérêts conflictuels.

**Contribution des auteurs**

MMK était le chercheur principal. Tous les auteurs ont participé à l’élaboration de l’étude. Tous les auteurs ont lu le manuscrit, puis approuvé la version définitive de celui-ci.

**Remerciements**

Les auteurs souhaitent remercier le ministère de la Santé et de l’Hygiène, le comité directeur local et l’équipe de gestion de la santé à l’échelle du district pour leur participation à la supervision de l’étude et pour les documents et les renseignements qu’ils ont fournis. Ils remercient également les adjoints de recherche, le personnel et les femmes rencontrés dans les établissements où l’étude a été menée, ainsi que l’Organisation ouest-africaine de la santé pour le soutien technique et l’encadrement qu’elle a offerts.

**Financement**

Le CRDI a financé l’étude par l’intermédiaire de l’Organisation ouest-africaine de la santé, dans le cadre de la mise en oeuvre d’un projet régional, à savoir l’Initiative ouest-africaine de renforcement des capacités au moyen de la recherche sur les systèmes de santé.

**Références**

1. Organisation mondiale de la Santé (OMS), *Tendances de la mortalité maternelle : 1990-2015*, Estimations de l’OMS, l’UNICEF, l’UNFPA, le Groupe de la Banque mondiale et la Division de la population des Nations Unies, 2015.
2. Khan, K., D. Wojdyla, L. Say, A. Gülmezoglu et P. Van Look, « WHO analysis of causes of maternal death: a systematic review », *Lancet*, vol. 367, 2006, p. 1066-1074.
3. Programme des Nations Unies pour le développement (PNUD), *Discussion Paper: A Social Determinants Approach to Maternal Health*, octobre 2011.
4. Kuruvilla, S., J. Schweitzer, D. Bishai, S. Chowdhury, D. Caramani, R. Cortez, B. Daelmans, A. de Francisco, T. Adam, N.Y. Alfonso, J. Franz-Vasdeki, S. Saadat, B.E. Pratt, B. Eugster, P. Venkatachalam, R. Hinton, S. Murray Arscott-Mills, H. Axelson, I. Sarker, R. Lakshminarayanan, T. Jacobs, S. Jacks, E. Mason, N. Mays, C. Presern, F. Bustreo, S. Bandali, B. Maliqi et A. Ghaffar, au nom des Success Factors for Women’s and Children’s Health study groups, « Facteurs de réussite pour la réduction de la mortalité maternelle et infantile », *Bulletin de l’Organisation mondiale de la Santé*, vol. 92, 2014, p. 533-544.
5. Organisation mondiale de la Santé, « Reducing maternal and newborn mortality in Africa », *African Health Monitor*, vol. 5, n^o^ 1, 2004, p. 5-7.
6. Van Lerberghe, W. et V. De Brouwere, « Of blind alleys and things that have worked: history’s lessons on reducing maternal mortality », *in* De Brouwere, V. et Van Lerberghe W. (éd.), *Safe motherhood strategies: a review of the evidence*, (Studies in Health Services Organisation and Policy), Antwerp, ITG Press, vol. 17, 2001, p. 7-33.
7. Monitoring the status of children and women, UNICEF. Accès : [http://​www.​childinfo.​org/​delivery_​care_​countrydata.​php](http://www.childinfo.org/delivery_care_countrydata.php) [consulté le mercredi 25 mai 2016].
8. Chambers, V. et D. Booth, « Delivering maternal health: Why is Rwanda doing better than Malawi, Niger and Uganda? », *Overseas Development Institute Briefing Paper*, vol. 74, 2012.
9. Alvarez, J.L., R. Gil, V. Hernández et A. Gil, « Factors associated with maternal mortality in Sub-Saharan Africa: an ecological study », *BMC Public Health*, vol. 9, 2009, p. 462.
10. Dzakpasu, S., T. Powell-Jackson et O.M.R. Campbell, « Impact of user fees on maternal health service utilization and related health outcomes: a systematic review », *Health Policy and Planning*, 2014, vol. 29, p. 1-14.
11. McKinnon, B., S. Harper, J.S. Kaufman et Y. Bergevin, « Removing user fees for facility-based delivery services: a difference-in-differences evaluation from ten sub-Saharan African », *Health Policy and Planning*, vol. 30, 2015, p. 432-441.
12. Gouvernement de la Sierra Leone, *Free healthcare services for pregnant and lactating women and young children in Sierra Leone*, Sierra Leone Conference, novembre 2009.
13. Health For All Coalition, Save the Children Fund, Royaume-Uni, *Free Health Care in Sierra Leone, One Year On, National Public and Stakeholder’s Perceptions of the Free Health Care Initiative*. Accès : http://healthforallcoalition.org/publications/ [consulté le 25 mai 2016].
14. Amnistie Internationale, *At a Crossroads: Sierra Leone’s Free Health Care Policy*, 2011.
15. Deen, N., « Assessing maternal healthcare access and quality in Sierra Leone », *The Harvard Undergraduate Research Journal*, 2012. Accès : <http://thurj.org/ss/2013/01/4091/>.
16. Witter, S., H. Wurie et M. Paola Bertone, « The free health care initiative: how has it affected health workers in Sierra Leone? », *Health Policy and Planning*, vol. 31, 2016, p. 1-9.
17. United States Agency for International Development (USAID), *ANC Observation Checklist, Maternal and Newborn Quality of Care Survey*, USAID.
18. Lissner, C., *Maternité sans risque – évaluation des besoins*, *in* Organisation mondiale de la Santé, *WHO/RHT/MSM/96.18*, Genève, 2001. Organisation mondiale de la Santé, *MSM/96.18*, Genève, 2001.
19. Gouvernement de la Sierra Leone, ministère de la Santé et de l’Hygiène, *Basic Package of Essential Health Services for Sierra Leone*, 2010.
20. Organisation mondiale de la Santé, *Rapport sur la santé dans le monde : donnons sa chance à chaque mère et à chaque enfant*, 2005.
21. Statistics Sierra Leone, Freetown, ministère de la Santé et de l’Hygiène de la Sierra Leone, Freetown, Sierra Leone et ICF International, Rockville, *Demographic and Health Survey 2008*, Maryland, États-Unis, juillet 2009.
22. Statistics Sierra Leone et UNICEF, *Sierra Leone Multiple Indicator Cluster Survey 2010*, décembre 2011.
23. Statistics Sierra Leone, Freetown, ministère de la Santé et de l’Hygiène de la Sierra Leone, Freetown, Sierra Leone et ICF International, Rockville, *Demographic and Health Survey 2013*, Maryland, États-Unis, juillet 2014.
24. Fonds des Nations Unies pour la population (FNUAP), *Trends in Maternal Health Indicators in Sierra Leone, 2008-2013*, DHS Further Analysis Reports, n^o^ 97, décembre 2015.
25. Nyamtema, A.S., A. Bartsch-de Jong, D.P. Urassa, J.P. Hagen et J. van Roosmalen, « The quality of antenatal care in rural Tanzania: what is behind the number of visits? », *BMC Pregnancy and Childbirth*, vol. 12, 2012, p. 70.
26. Kyei, N.N.A., C. Chansa et S. Gabrysch, « Quality of antenatal care in Zambia: a national assessment », *BMC Pregnancy and Childbirth*, vol. 12, 2012, p. 151.
27. Fagbamigbe, A.F. et E.S. Idemudia, « Assessment of quality of antenatal care services in Nigeria: evidence from a population-based survey », *Reproductive Health*, vol. 12, 2015, p. 88.
28. Organisation mondiale de la Santé et UNICEF, *Antenatal Care in Developing Countries: Promises, achievements and missed opportunities: An analysis of trends, levels and differentials, 1990-2001*, 2003.
29. Li, X.F., J.A. Fortney, M. Koltelchuck et L.H. Glover, « The postpartum period: the key to maternal mortality », *International Journal of Gynecology and Obstetrics*, vol. 54, 1996, p. 1-10.
30. Gouvernement de la Sierra Leone, ministère de la Santé et de l’Hygiène publique, *Sierra Leone – Service Availability and Readiness Assessment 2011*.
31. Gouvernement de la Sierra Leone, ministère de la Santé et de l’Hygiène publique, *Sierra Leone Service Availability and Readiness Assessment 2012 Report*.
32. Oyerinde, J., « Can Antenatal Care Result in Significant Maternal Mortality Reduction in Developing Countries?», *Journal of Community Medicine and Health Education,* vol. 3, 2013, p. 2.
33. Oyerinde, K., Y. Harding, P. Amara, R. Kanu, R. Shoo et K. Daoh, « The status of maternal and newborn care services in Sierra Leone 8 years after ceaseﬁre », *International Journal of Gynecology and Obstetrics*, vol. 114, 2011, p. 168-173.
34. Bosomprah, S., A.J. Tatem, W. Dotse-Gborgbortsi, P. Aboagye et Z. Matthews, « Spatial distribution of emergency obstetric and newborn care services in Ghana: Using the evidence to plan interventions », *International Journal of Gynecology and Obstetrics*, vol. 132, 2016, p. 130-134.
35. McPake, B., S. Witter, T. Ensor, S. Fustukian, D. Newlands, T. Martineau et Y. Chirwa, « Removing financial barriers to access reproductive, maternal and newborn health services: the challenges and policy implications for Human Resources for Health », *Human Resources for Health*, vol. 11, 2013, p. 46.
36. Ngongo, C., K. Christie, J. Holden, C. Ford et C. Pett, « Striving for excellence: Nurturing midwives’ skills in Freetown, Sierra Leone », *Midwifery*, vol. 29, 2013, p. 1230-1234.
37. Vallières, F., E.L. Cassidy, E. McAuliffe, B. Gilmore, A.S. Bangura et J. Musa, « Can Sierra Leone maintain the equitable delivery of their Free Health Care Initiative? The case for more contextualised interventions: results of a cross-sectional survey », *BMC Health Services Research*, vol. 16, 2016, p. 258.
38. Lang’at, E. et L. Mwanri, « Healthcare service providers’ and facility administrators’ perspectives of the free maternal healthcare services policy in Malindi District, Kenya: a qualitative study », *Reproductive Health*, vol. 12, 2015, p. 59.
39. Fabienne, R., A. Matthieu, S. Witter, A. Kelley, I. Sieleunou, Y. Kafando et B. Meessen, « Fee Exemption for Maternal Care in Sub-Saharan Africa: A Review of 11 Countries and Lessons for the Region », *Global Health Governance*, vol. VI, n^o^ 2, 2013. Accès : http://ghgj.org.
40. Gouvernement de la Sierra Leone, *Free healthcare services for pregnant and lactating women and young children in Sierra Leone*, novembre 2009.
41. Organisation mondiale de la Santé, *Health Expenditure Indicators*, Genève, OMS, 2015. Accès : <http://apps.who.int/nha/database/Select/Indicators/en> [consulté le 2 mai 2016].
