# Supplementary material for: People and research: improved health systems for West Africans, by West Africans - report on special supplement
Source: BMC Proc. 2019 Feb 7;13(Suppl 1):1. doi: 10.1186/s12919-019-0162-0 (PMC6366023; doi:10.1186/s12919-019-0162-0)
Supplement: Supplementary file 4 — Gratuité ou subvention: options d’exemption de paiement des frais de santé, accès aux soins des groupes vulnérables et effets sur le système de santé au Burkina Faso, Yaogo, M. [file 12919_2019_162_MOESM4_ESM.docx]

***Gratuité ou subvention : options d’exemption de paiement des frais de santé, accès aux soins des groupes vulnérables et effets sur le système de santé au Burkina Faso***

**Maurice Yaogo^1,2^**

Correspondance : [myaogo.proline21@gmail.com](mailto:myaogo.proline21@gmail.com)

Unité de Formation et de Recherche/Lettres et Sciences Humaines, Université Catholique d’Afrique de l’Ouest – Unité Universitaire, Bobo-Dioulasso, Burkina Faso

Association Zama Forum pour la Diffusion des Connaissances et des Expériences novatrices en Afrique (Zama Forum/ADCE – Afrique), Bobo-Dioulasso, Burkina Faso

# Résumé

**Contexte :** Les divers types d’exemptions de paiement mis en place au Burkina Faso depuis les années 2000 vont de l’exemption totale (gratuité) à la subvention des coûts. L’article porte sur ces deux options, leurs variantes contextuelles et les manières dont ces interventions influent sur l’accès aux soins des personnes vulnérables et le fonctionnement du système de santé. Ce travail s’inscrit dans le programme régional interdisciplinaire sur l’abolition du paiement des services de santé en Afrique de l’Ouest (Burkina Faso, Mali et Niger).

**Méthode :** Un cadre conceptuel, complété par une revue chronologique des interventions, sert de référence pour la synthèse des résultats des trois études qualitatives présentées. Les repères historiques permettent de décrire la genèse des politiques d’exemption de paiement des soins au Burkina Faso et les événements qui ont influé sur leur adoption. L’analyse conjointe faite des appréciations sur les options d’exemption de paiement est axée sur les répercussions multiformes sur l’accès aux soins des bénéficiaires et sur le fonctionnement du système de santé.

**Résultats :** Les exemptions de paiement des soins ont évolué depuis la colonisation, conjointement avec les obstacles touchant la formulation progressive d’une politique nationale de santé et en réponse aux recommandations internationales. Les limites des changements introduits avec le recouvrement des coûts et les obstacles à l’accès aux soins des plus pauvres ont mené à l’adoption des exemptions sectorielles de paiement en cours. Les résultats renseignent notamment sur les motivations des changements survenus au fil du temps. Les appréciations nuancées de différentes catégories de personnes interrogées sur les options d’exemption montrent qu’au-delà des effets perçus sur l’accès aux soins et sur le système de santé, c’est la question d’une gouvernance plus équitable de la santé qui se pose.

**Conclusions :** En principe, les effets recherchés avec l’application des mesures d’exemption de paiement sont favorables à l’accès amélioré aux soins des groupes vulnérables. Les effets pervers sur le fonctionnement du système de santé plaident en faveur de réformes pour harmoniser les changements liés aux exemptions sectorielles de paiement et les besoins actuels afin de garantir leur efficacité et leur durabilité.

**Mots clés** : exemptions de paiement, accès aux soins, groupes vulnérables, système de santé, Burkina Faso

**Contexte**Les politiques publiques de santé et les exemptions de paiement des soins

Pays à ressources limitées, le Burkina Faso fait face à d’importants défis dans les secteurs prioritaires du développement, soit l’agriculture, l’éducation et la santé. Les politiques publiques sont particulièrement attentives à l’amélioration des indicateurs dans ces domaines prioritaires, et l’adoption et la mise en oeuvre des Objectifs du Millénaire pour le développement (OMD) ont donné lieu à plusieurs réformes visant leur atteinte. C’est le cas de la question cruciale de l’accès aux soins qui a toujours constitué une réalité difficile à concrétiser. Après l’instauration du paiement direct des soins comme modalité principale de financement du système de santé dans les années 1980 au Burkina Faso et dans d’autres pays africains signataires de l’Initiative de Bamako, plusieurs travaux réalisés depuis les années 1990 en contexte africain ont montré les effets négatifs d’une telle option sur l’utilisation des services [1, 2], notamment pour les femmes et les plus pauvres. Malgré un contexte défavorable caractérisé par un niveau de pauvreté élevé au sein de la population (40,1 % en 2014 contre 46 % en 2009 [3]) et des indicateurs nécessitant des efforts accrus pour atteindre les standards, l’atteinte des OMD faisait partie des engagements prioritaires des décideurs nationaux. Relever un tel défi supposait un accès amélioré et plus équitable aux soins conventionnels, d’où une succession de décisions [4, 5] depuis les années 2000 en faveur de la subvention ou de la suppression du paiement direct pour certaines catégories de la population ou pour certaines affections.

Instauré dans les années 1980 par la Banque mondiale et le Fonds monétaire international, l’ajustement structurel appliqué aux économies africaines en crise a contraint les États à réduire leurs dépenses en matière de santé et d’éducation. Ce redressement en référence aux standards internationaux a eu des conséquences dramatiques sur la prestation de services publics, particulièrement dans le domaine de la santé. La gratuité totale des services était censée se poursuivre, mais il manquait de médicaments dans la pharmacie centrale qui approvisionnait les dépôts de médicaments des centres de santé régionaux. De nouvelles solutions s’imposaient, notamment celles concernant les services de santé financés par les utilisateurs et l’instauration du paiement direct. En Afrique de l’Ouest, cela a notamment donné lieu à la création de l’Initiative de Bamako (IB) mise en place avec la participation de l’Organisation mondiale de la Santé (OMS) et de l’UNICEF [6]. L’IB a instauré le paiement direct des soins, en même temps qu’une gestion communautaire, et la délivrance de médicaments génériques. Les sommes perçues étaient gérées par un comité de gestion local composé d’habitants des villages environnant le centre de santé, qui commandait des médicaments génériques.

Deux options d’exemption totale ou partielle de paiement des soins ont été mises en place ces 10 dernières années au Burkina Faso, à la suite de mesures antérieures énoncées dans la revue chronologique présentée ci-après et rappelées dans la genèse des faits historiques. La première option concerne l’offre d’accès gratuit aux services de santé, familièrement qualifiée de « gratuité » des soins selon la signification profane, qui est différente de la formulation technique correspondant à l’exemption totale de paiement [7].

La deuxième concerne la subvention (exemption partielle) des soins, qui varie selon les interventions et les affections. La réflexion à ce sujet s’est engagée à partir de la revue globale de trois études prises en compte dans le cadre du programme régional de recherche sur l’« abolition du paiement des services de santé en Afrique de l’Ouest », de 2009 à 2012. Nous ne traitons pas ici d’une exemption particulière de paiement des soins de santé comme nous l’avons fait dans l’ouvrage collectif renfermant les résultats de ce programme de recherche [8–11]. L’examen porte ici sur les similarités et les spécificités entre les interventions relatives aux exemptions sectorielles totales ou partielles de paiement des services de santé (gratuité ou subvention) dans le contexte sanitaire burkinabè. Des aspects généraux communs à plusieurs pays de la sous-région sont relevés notamment par Olivier de Sardan [11], Van Lerberghe et De Brouwere [12]. Notre analyse se réfère d’abord à l’émergence d’un « paradigme d’exemption » dans les années 2000 qui constitue le point de départ de l’adoption de plusieurs initiatives de réduction des frais de santé. À ce sujet, l’évolution des prises de position officielles sur la question des exemptions pour diverses interventions montre des visions antagonistes entre les défenseurs et les pourfendeurs de l’exemption totale de paiement des soins (gratuité). Les premiers défendent le bien-fondé et la nécessité d’introduire une exemption totale (gratuité) des services au moyen d’arguments tendant à prouver sa faisabilité et son utilité sociale. Quant aux seconds, ils plaident pour une exemption partielle (subvention) comme solution pérenne et supportable pour un pays à faible revenu [13]. Une description plus détaillée est faite dans la présentation des résultats sur les sensibilités existantes et les arguments formulés par les uns et les autres.

Le programme de recherche sur l’abolition du paiement des services de santé en Afrique de l’Ouest a été réalisé de 2009 à 2012 au Burkina Faso, au Mali et au Niger. Dans un contexte de réformes des politiques publiques de santé en réponse aux défis liés aux OMD, l’émergence concomitante d’exemptions de paiement apparaissait comme une rupture paradigmatique [14] à explorer malgré la faible disponibilité de travaux traitant de ces questions en Afrique de l’Ouest. La réalité est que l’analyse des politiques et des systèmes de santé est encore au stade embryonnaire en contexte ouest-africain. Cela est essentiellement attribuable au petit nombre de chercheurs africains, d’où une faible production scientifique. Il est question aussi du fait que la tradition francophone de recherche en santé publique est très médicalisée et axée sur les aspects épidémiologiques, ce qui est peu propice aux remises en question interdisciplinaires concernant les systèmes de santé [15].

Amorcées dans le cadre d’une collaboration entre chercheurs et décideurs de plusieurs institutions du Nord et du Sud, les activités de recherche répondaient au besoin d’éclairer par des données probantes certains aspects des politiques locales en matière d’accès aux soins [16]. S’inscrivant dans la recherche de données sur plusieurs questions omises ou peu prises en compte dans les approches traditionnelles en matière de santé publique, l’objectif était de combler certaines lacunes concernant l’étude des politiques et des systèmes de santé. En effet, de nouvelles approches sont nécessaires concernant notamment la gouvernance des systèmes de santé, les conceptions et les pratiques des parties prenantes, mais aussi les écarts entre ce qui est énoncé dans les documents officiels et le fonctionnement réel des services de santé. Ainsi, plusieurs dimensions des contextes d’émergence, de formulation, de mise en oeuvre, d’exécution et des effets induits des politiques publiques de santé ont été explorées par une équipe sous la coordination de l’auteur.

L’objectif principal du programme de recherche était d’évaluer la question de l’abolition sectorielle du paiement direct des soins de santé pour certains groupes ou certaines maladies comme instrument de politique afin de favoriser l’utilisation des services et l’égalité d’accès aux soins. Cela justifie l’intérêt porté à la genèse des politiques publiques permettant de comprendre les motivations sous-jacentes et les changements d’une période à l’autre de l’histoire. Conjointement, la revue des différentes options d’exemption de paiement adoptées au fil du temps est riche d’enseignements au sujet des effets sur l’accès aux soins des plus pauvres et sur le système de santé. C’est pourquoi le cadre conceptuel et la revue chronologique servent de repères pour orienter l’analyse du contenu des interventions.

La première étude [13] aborde, sous l’angle des conceptions des parties prenantes et des bénéficiaires, l’histoire de la gratuité des soins, en s’intéressant aux changements progressifs dans l’organisation du système de santé. Quant à la deuxième et à la troisième étude [9, 10], elles portent sur le processus de mise en place de deux interventions concomitantes pour accroître l’accès aux soins antipaludéens (gratuité des moustiquaires imprégnées d’insecticide pour la prévention et subvention des thérapies combinées à base d’artémisinine [ACT] pour le traitement du paludisme) ainsi qu’aux médicaments antirétroviraux dans le cadre des interventions financées par le Fonds mondial comparativement au circuit national de traitement gratuit de la tuberculose (TB).

La revue d’ensemble de ces études à l’aide du cadre conceptuel et de la revue chronologique vise à décrire la chronologie des mesures prises, mais aussi les similarités, les différences et les répercussions des interventions, selon les principaux thèmes abordés.

# Méthodes

Une comparaison des interventions prises en compte par les trois études citées est réalisée à partir du cadre conceptuel, en conformité avec l’orientation méthodologique du programme de recherche. Celle-ci a consisté en une combinaison d’approches (qualitatives et quantitatives), opérationnalisées par des études de cas multiples comprenant plusieurs niveaux d’analyse correspondant aux différents échelons (national, régional et local) de la pyramide sanitaire ainsi qu’aux types de services concernés selon les pays. Les études rapportées ici relèvent uniquement de l’approche qualitative utilisée à la fois pour la collecte, le traitement et l’analyse des données autour des centres d’intérêt explorés, en exploitant plusieurs sources de données. En effet, en plus des données documentaires (écrits sur le sujet, exploitation de certaines archives publiques), des entretiens individuels en profondeur ont été réalisés auprès des catégories de parties prenantes concernées (voir le tableau 1).

Il s’agissait de comprendre la manière dont l’abolition est mise en place, ses forces et ses faiblesses, les difficultés éprouvées, les perceptions qu’en ont différentes catégories de parties prenantes, dont les fournisseurs de soins et les patients, ainsi que les stratégies des acteurs sociaux. Pour cela, la collecte et l’analyse détaillée des données ont permis de mieux cerner le point de vue des parties prenantes dans l’arène sociale et professionnelle et de recueillir les perspectives courantes sur les sujets abordés [17]. Les données recueillies concernent à la fois les perceptions et les pratiques courantes des acteurs politiques ou sanitaires en lien avec les interventions et les politiques publiques relatives à l’exemption de paiement des soins.

La figure 1 décrit l’articulation entre les repères chronologiques et les options d’exemption de paiement. La revue chronologique des interventions décrit la succession des événements qui ont influé sur les options d’exemption de paiement des soins depuis l’implantation médicale pendant la période coloniale. Cela permet de comprendre l’ancrage historique des options progressives d’exemption de paiement. S’inscrivant dans des événements liés aux politiques d’exploitation coloniale, suivies de la mise en place ultérieure des politiques nationales de développement et de santé, c’est la référence de base du cadre conceptuel (figure 2). En effet, il s’agit de faits réels qui ont jalonné le cheminement qui a abouti aux options actuelles de suppression ou de subvention des frais de santé.

Quant à la description de la nature et du contenu des options d’exemption à partir de repères typologiques, elle renseigne sur les changements progressifs en rapport avec les événements historiques qui jalonnent l’émergence et la formalisation de la politique nationale de santé. Dans ce cas, l’idée de base est de proposer ce qui décrit le mieux la configuration des interventions dans un contexte d’exemption totale ou partielle de paiement des soins, à des périodes de référence liées à l’axe chronologique. Ainsi, il y a une mise en relation entre les repères historiques et les options successives reflétant la typologie des politiques d’exemption de paiement et leurs changements structurels (cas du passage au recouvrement des coûts après l’exemption totale de paiement) ou conjoncturels (cas des mesures récentes en réponse aux besoins liés à un accès plus équitable aux soins des groupes vulnérables). Cela permet de mettre en relief les conséquences à plusieurs niveaux. Dans le cas présent, l’intérêt porte sur l’accès aux soins des groupes vulnérables et sur l’incidence sur le fonctionnement du système de santé.

La revue chronologique proposée peut être appliquée à l’analyse d’autres domaines d’actions, avec une mise en relation dynamique entre les faits historiques saisis dans leur évolution et leur incidence sur la formalisation des politiques publiques.

Le cadre conceptuel est celui qui a été élaboré et utilisé par Van der Veken et coll. [18] pour analyser les effets d’une politique d’exemption de paiement des soins sur le système de santé local (district). L’idée de base est que les six fonctions essentielles du système de santé définies par l’OMS [19] ne rendent pas suffisamment compte des interactions dynamiques entre les différentes composantes. Dans une perspective différente, le cadre conceptuel proposé décrit les différentes composantes du système de santé local et leurs relations.

**Tableau 1 Description des trois études concernées**

| Type d’étude | Portée de l’analyse | Sources de données | Profils des personnes interrogées | Nombre de personnes interrogées |
| --- | --- | --- | --- | --- |
| Histoire de la gratuité des soins | Nationale | Documentation scientifique, archives administratives et médias imprimés, entretiens semi-structurés approfondis | Santé, agriculture et éducation (décideurs, techniciens et responsables de la mise en oeuvre) | n = 28 |
| Exemptions de paiement pour le paludisme (prévention et traitement) | District  (région des Hauts-Bassins) | Documentation scientifique, données de routine, entretiens semi-structurés approfondis et discussions de groupe | Décideurs, acteurs locaux (organisations non gouvernementales [ONG] et associations) et fournisseurs de soins | n = 63 |
| Exemptions de paiement pour les médicaments antirétroviraux (enfants et adultes) | Régionale (régions du Centre et des Hauts-Bassins) | Documentation scientifique, données de routine et entretiens semi-structurés | Décideurs institutionnels, acteurs locaux (ONG et associations), fournisseurs de soins et utilisateurs des services | n = 30 |

L’axe central du modèle proposé par Van der Veken et coll. [18] pour l’analyse des effets des politiques d’exemption de paiement sur le système de santé local (cartographie des incidences des politiques) consiste en quatre éléments clés, soit la gouvernance et la gérance, le personnel responsable de la prestation des soins de santé, l’offre de soins et la communauté. En revanche, les médicaments, les fournitures, les ressources financières et le système d’information sanitaire sont considérés comme des ressources de l’axe central. Tous les éléments sont liés entre eux et une politique ciblant l’un d’eux peut avoir des effets (attendus ou inattendus, positif ou négatifs) sur un autre élément, ce qui reflète la complexité du système [20].

À partir de ce modèle qui complète la revue chronologique présentée ci-dessus, l’intérêt porte sur l’incidence des exemptions sectorielles de paiement sur l’offre de services (régime officiel) aux groupes ciblés, plus précisément les obstacles à l’accès des groupes défavorisés, mais aussi les conséquences positives et négatives sur le système de santé. Une autre dimension importante concerne la provenance des ressources financières (bailleurs de fonds internationaux et institutions nationales) qui conditionne la durée des exemptions sectorielles de paiement.

# Résultats

Une synthèse globale des résultats des trois études est présentée en nous référant aux deux axes thématiques (repères contextuels et typologiques) représentés dans la revue chronologique. Tout d’abord, un aperçu général est donné des faits marquants qui ont influé sur la prise de décisions en faveur des différentes formes d’exemptions de paiement. Par la suite, il est question du contexte d’émergence et de formulation de ces options de suppression ou de réduction des frais de service en faisant ressortir leurs similarités et leurs différences. Enfin, les points de vue variés de différentes catégories socioprofessionnelles sont abordés, en considérant les enjeux de ces mesures d’exemption de paiement sous l’angle des projections faites pour l’atteinte des objectifs de santé qui fait l’objet de cet article.

**Politiques publiques et exemptions de paiement des soins : quelques repères chronologiques (première étude)**

Comme le montre la revue chronologique proposée, cinq grandes périodes caractérisent l’évolution historique de la gratuité :

- la période coloniale, où la gratuité totale était prise en charge conjointement par l’administration coloniale et les missions chrétiennes (1920-1960);
- la période de l’indépendance, où la gratuité des soins s’est poursuivie et où les enjeux sanitaires étaient intégrés à la mise en oeuvre de plans de développement particuliers;
- la période des réformes internationales des soins de santé (santé et économie), où l’on a assisté à l’application de politiques d’ajustement structurel défavorables aux secteurs sociaux, dont la santé, et au recouvrement des coûts avec l’Initiative de Bamako;
- la période de remise en question caractérisée par la prise en compte à l’échelle internationale des inégalités, ainsi que par les grandes initiatives axées sur l’amélioration de l’accès aux soins (OMD, Fonds mondial, etc.);
- la période actuelle qui a commencé dans les années 2000 par l’introduction de plusieurs exemptions sectorielles de paiement des soins.

Cette section retrace les grandes lignes des exemptions multiformes de paiement des soins mises en place pendant les périodes de référence susmentionnées. Pour faire le lien avec la chronologie des politiques publiques adoptées, les grandes dates suivantes constituent des repères :

1. la mise en oeuvre du *Plan-cadre* (Plan-cadre de politique en matière de développement : initialement de 1967 à 1970, puis de 1972 à 1976) consacré aux secteurs considérés comme prioritaires pour le développement local (agriculture et économie), à la différence des secteurs dits « sociaux » (santé et éducation);
2. l’adoption du premier *Code de santé publique* en 1970, réactualisé en 1994 sous la forme d’une loi adoptée à l’Assemblée nationale pour préciser les dispositions réglementaires et les conditions d’exercice des différentes professions liées à la santé humaine;
3. la mise en place en 1978 du premier *Document de programmation sanitaire nationale* avec l’appui de l’OMS qui servira de base à l’élaboration de la politique sanitaire [21];
4. l’élaboration du *Document de politique sanitaire nationale* [22];
5. l’élaboration du *Plan national de développement sanitaire* pour la période de 2001 à 2010, suivie d’une prolongation de 2011 à 2020;
6. l’adoption officielle de la première *Politique nationale de santé* [23].

## Tableau 2 Revue comparative des interventions examinées

|  | Type d’exemption appliquée | Engagement budgétaire du gouvernement | Planification et mise en oeuvre |
| --- | --- | --- | --- |
| Tuberculose | Soins gratuits (sauf les tests de diagnostic) | Budget disponible (ministère de la Santé) | – Existence de documents officiels – Diffusion d’information par l’intermédiaire des médias et d’associations – Service de communication institutionnelle |
| Médicaments antirétroviraux (ARV) pour les enfants | Médicaments ARV gratuits; ne comprend pas les examens de suivi biologiques et les médicaments de soutien (conditions différentes d’une organisation à l’autre) | Aucun engagement budgétaire officiel | – Aucun document officiel – Diffusion d’information limitée (centres de soins de santé et associations) |
| Médicaments ARV pour les adultes | Médicaments ARV gratuits; ne comprend pas les examens de suivi biologiques et les médicaments de soutien (conditions différentes d’une organisation à l’autre) | Aucun engagement budgétaire officiel outre des subventions externes (Fonds mondial) | – Déclaration officielle dans Tate, 2009 – Diffusion d’information adéquate (centres de soins de santé, associations et médias) |
| Thérapies combinées à base d’artémisinine | Subventions dont les taux varient en fonction de l’âge (2 à 11 mois, 1 à 5 ans, 6 à 13 ans, et 14 ans et plus) | Financées entièrement par le Fonds mondial; permet d’offrir des soins gratuitement | – Service lancé sans diffusion d’information au préalable (aux fournisseurs ou aux utilisateurs) – Entrée en vigueur en avril 2009 – Aucun document officiel |
| Moustiquaires de lit imprégnées d’insecticide (femmes enceintes et nouveau-nés) | Offertes gratuitement (distribuées aux femmes lors des consultations précédant et suivant l’accouchement) | Financement externe (Fonds mondial) dans le cadre d’un projet pilote avant la mise à l’échelle | – Conditions de diffusion d’information inégales; limité aux femmes recevant des soins postnatals |
| Moustiquaires de lit imprégnées d’insecticide (campagne de distribution nationale de 2010) | Offertes gratuitement (quota par ménage) | Financées entièrement par le Fonds mondial (Programme d’appui au développement sanitaire et Plan Burkina) | – Obligations contractuelles (demandes de financement du Fonds mondial) – Campagne de communication dans les médias publics (radio, télévision et médias imprimés) – Existence d’un calendrier de mise en oeuvre des directives et des conditions, mais cibles non atteintes |


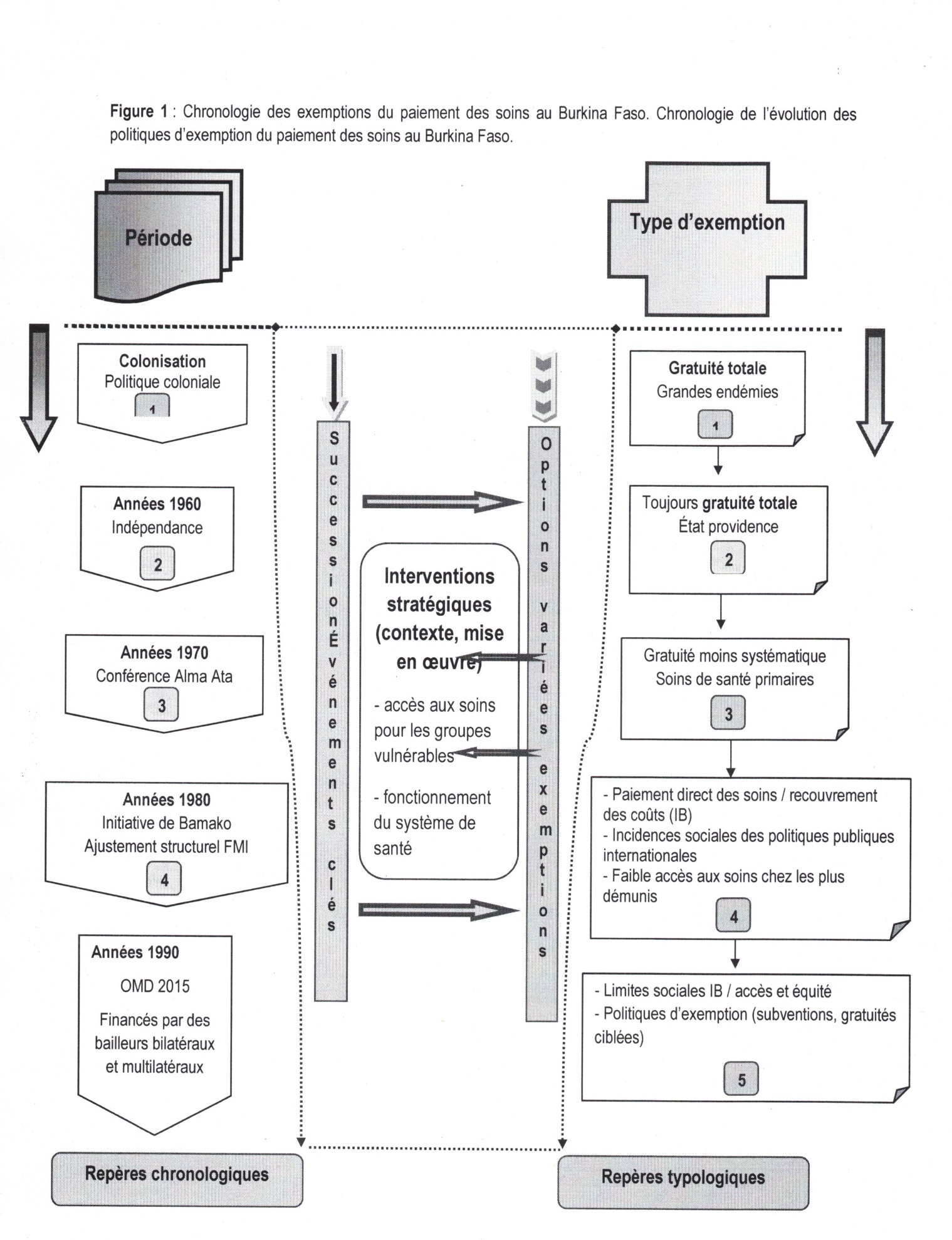


S’il est difficile de faire un lien direct entre l’application des politiques de santé et les mesures d’exemption de paiement, le fait majeur que représente l’instauration de la subvention des accouchements et des soins obstétricaux et néonatals d’urgence en 2006 marque une rupture avec les pratiques antérieures. En effet, l’engagement financier à long terme pour une action à l’échelle nationale a été adopté dans le budget de l’État dans le cadre des OMD 2015. Cependant, d’autres faits marquants ont précédé cette intervention. L’un des documents de référence [24] donne un aperçu des expériences antérieures, notamment la subvention des soins obstétricaux d’urgence dans les hôpitaux et l’initiative de partage des coûts.

Diverses formes d’interventions dans le domaine de la santé ont été entreprises depuis l’avènement de la médecine moderne dans les années 1930.


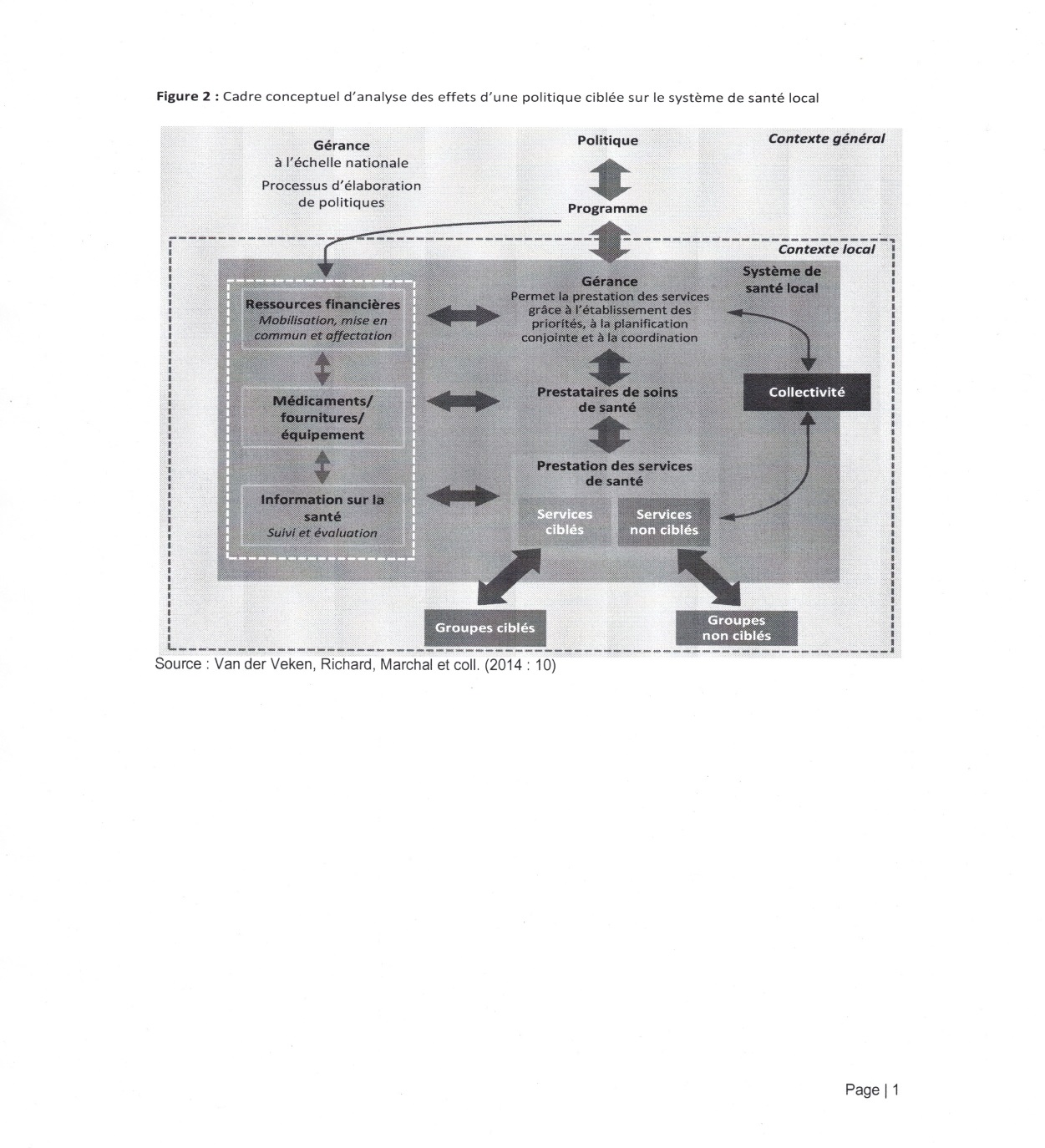


Pour bien comprendre l’évolution jusqu’à la période actuelle, un petit aperçu est utile pour cerner les difficultés liées à la mise en place progressive de la politique de santé. Cela permet de mettre en relief ce qui a prévalu avant l’adoption de la gratuité des soins depuis la formation du gouvernement après la colonisation. Jusqu’à la période de l’indépendance, les activités de santé s’inscrivaient dans la « perspective élargie du développement socio-économique en général » (traduction [21], p. 2 [25]). Ainsi, le premier plan de développement nommé *Plan-cadre* a été réalisé dans la période allant de 1967 à 1970, après plusieurs tentatives infructueuses entre 1962 et 1966. Plusieurs écrits ont montré un lien entre deux domaines d’intervention de ce plan, soit la santé et l’agriculture; ainsi, de grandes interventions sanitaires (cas de la lutte contre les grandes endémies) ont précédé les mesures de développement agricole dans cette période. À l’époque, le contrôle des maladies endémiques et l’encadrement agricole étaient considérés comme des cibles pour la réalisation de la politique agricole qui visait à former des entrepreneurs agricoles pour l’exploitation de périmètres hydro-agricoles aménagés sur toute l’étendue du territoire. De même, l’exploitation de grandes superficies de terres fertiles infestées par l’onchocercose ne pouvait se faire qu’après les mesures de grande envergure de lutte contre les grandes endémies [26].

Les grandes lignes d’une politique générale de santé avaient été définies dans le *Plan-cadre* conjointement à une politique du personnel. La première orientation mentionnait à la fois la poursuite des programmes de contrôle de certaines maladies transmissibles et le renforcement des activités de base de santé publique (statistiques, éducation de base, nutrition et hygiène). La deuxième orientation portait sur le renforcement de la formation du personnel, les investissements nécessaires et les sources de financement. Les recommandations issues du premier *Plan-cadre* ont été reprises à quelques nuances près pour le deuxième, qui couvrait la période de 1972 à 1976. Elles ont servi de référence pour l’élaboration du premier *Document de programmation sanitaire nationale* pour la période de 1980 à 1990. Jusqu’à la fin des années 1980, c’est le *Code de santé publique* de 1970 qui orientait les actions du ministère de la Santé. Ce Code se limitait à réglementer les services et les soins médicaux. L’adoption en mars 1979 des recommandations de la Conférence d’Alma-Ata sur les soins de santé primaires est considérée comme le point de départ d’une véritable politique de santé avec l’élaboration consécutive du *Document de programmation sanitaire nationale*. Conformément à l’orientation globale qui était d’assurer une meilleure couverture sanitaire à la population, une structure sanitaire pyramidale, qui sert encore de référence aujourd’hui, devait être établie, à savoir : 1) un établissement de soins de santé primaires (ESSP) par village, 2) un centre de santé et de promotion sociale (CSPS) pour une population de 15 000 à 20 000 habitants dans un rayon maximum de 20 km, 3) un centre médical par sous-préfecture, 4) deux hôpitaux nationaux dans les deux plus grandes villes du pays (Ouagadougou et Bobo-Dioulasso), et 5) une école des sciences de la santé. Actuellement, le premier niveau du système de santé n’est plus l’ESSP qui n’est plus opérationnel, mais le CSPS. Il y a également un changement dans la répartition spatiale des structures de santé en raison des nouveaux découpages administratifs successifs et de la réorganisation consécutive du système de santé, en plus de l’adoption d’une convention instituant les districts sanitaires comme unités fonctionnelles du système de santé. La formation du personnel médical est assurée principalement par deux universités publiques, initialement à Ouagadougou puis à Bobo-Dioulasso, et plus récemment, par des instituts privés agréés par l’État et le *Conseil Africain et Malgache pour l’Enseignement Supérieur.* Trois établissements publics de recherche en santé ont également été mis en place, dont le Centre Muraz à Bobo-Dioulasso dans les années 1940 pour la lutte contre les grandes endémies.

Des changements survenus dans l’organisation et le fonctionnement du système de santé, on retiendra qu’après avoir connu une centralisation notoire de 1960 à 1979, un mouvement de décentralisation progressive a été amorcé. Quatre périodes distinctes caractérisent cette évolution [27] :

1. La période de 1960 à 1979 est présentée comme celle de la centralisation de l’administration sanitaire.
2. La période de 1980 à 1989 est considérée comme celle de la décentralisation progressive de l’administration de la santé et de l’amorce d’une décentralisation technique. C’est le début de l’autonomie accordée par des textes juridiques à des services publics consacrés à la santé, par exemple, le statut d’établissement public à caractère industriel et commercial ou celui de société d’économie mixte. Dans le secteur pharmaceutique, cela a été le cas de l’Office national d’approvisionnement pharmaceutique, devenu en 1985 une société d’État dénommée Société nationale d’approvisionnement pharmaceutique, qui a été privatisée en 1994.
3. La période de 1990 à 1993 est décrite comme celle de la décentralisation technique avec l’augmentation du nombre de districts, qui passe de 45 à 53, puis à 63 aujourd’hui.
4. La période actuelle est considérée comme celle de l’approfondissement de la décentralisation sanitaire avec ses difficultés. Ainsi, après l’organisation du système de santé en districts (arrêté ministériel du 30 novembre 1993), des mesures prises en 2001 introduisaient un nouveau découpage du territoire national en 13 régions administratives qui sert de référence pour la réorganisation du système de santé. La possibilité de faire coïncider la décentralisation territoriale et celle des services de santé avait suscité un débat sur l’efficacité fonctionnelle d’une telle option.

Dans le contexte des changements progressifs touchant l’organisation et le fonctionnement du système de santé, les politiques d’exemption sectorielles, telles qu’elles sont appliquées aujourd’hui, sont décrites en suivant l’évolution de deux grandes interventions. Celles-ci ont grandement contribué aux décisions d’instaurer la réduction ou la suppression totale du paiement des frais de santé. La première grande composante consiste en un ensemble de mesures progressives pour asseoir les grands axes de la politique de santé à partir des orientations de la Conférence d’Alma-Ata sur les soins de santé primaires (1987) et des recommandations de l’Initiative de Bamako (1987). Ces deux grandes initiatives convergentes dans les objectifs ont servi de modèles durant la période postcoloniale jusqu’à l’avènement des exemptions sectorielles de paiement des soins. De ce point de vue, nous conviendrons de caractériser l’avènement des soins de santé primaires en évoquant le paradigme de la participation communautaire et du paiement direct des soins au point de service. Il précède celui de l’exemption de paiement des soins, en référence aux interventions multiformes depuis les années 2000. Quelques difficultés liées à leur application seront passées en revue pour montrer ce qui a conduit à un changement de paradigme dicté par les obstacles à l’accès aux soins, notamment pour les plus pauvres.

Selon l’OMS [19], « les soins de santé primaires sont des soins essentiels fondés sur des méthodes pratiques, scientifiquement valables et socialement acceptables, et des technologies universellement accessibles aux individus et aux familles, dans les communautés, à travers leur complète participation à un coût que la communauté et le pays peuvent prendre en charge à chaque étape de leur développement dans un esprit d’autosuffisance. Ils forment une partie intégrale aussi bien du système de santé du pays dont ils constituent le pilier, que du développement économique et social global de la communauté. »

L’Initiative de Bamako (IB) est une approche visant à renforcer les soins de santé primaires grâce à ce qu’il est convenu d’appeler la participation communautaire au financement et à la gestion des services de santé. La vente des médicaments essentiels génériques constitue le moyen principal de financement des soins de santé primaires. Le mécanisme de base de l’IB est le recouvrement des coûts au moyen d’une démarche de mobilisation des utilisateurs locaux. Pour cela, un premier stock de médicaments est fourni à la population; il est demandé aux structures locales de gestion d’assurer la vente des médicaments essentiels génériques aux utilisateurs à des prix abordables, avec une marge bénéficiaire modeste. Les sommes recueillies servent en priorité à renouveler le stock de médicaments auprès de la structure de distribution qui est la Centrale d’achat des médicaments essentiels génériques (CAMEG). Les profits servent au financement de certains coûts liés aux structures de santé et à la distribution de primes au personnel des services de santé. Le succès de l’IB requiert une forte participation de la population. Une formation des parties prenantes est nécessaire pour assurer leur participation à la définition des activités de santé et à la gestion financière. L’objectif principal est l’amélioration de l’efficacité des services, la limitation des coûts et la continuité des services sur fond de réorganisation du système de santé.

Au sujet de l’adoption des soins de santé primaires au Burkina Faso et dans plusieurs pays d’Afrique subsaharienne, l’expérience montre qu’une solution était proposée pour la résolution des problèmes sanitaires des pays en développement, en particulier en Afrique subsaharienne. C’était en même temps une remise en question des initiatives antérieures avec leurs limites ou leurs échecs, particulièrement en matière de prévention et de médecine de masse (dans le cas de la forte prévalence des maladies transmissibles et d’un taux de mortalité maternelle et infantile élevé), ainsi que de la faible efficacité d’une médecine hospitalière trop coûteuse et dysfonctionnelle [28, 29]. Cependant, plusieurs critiques ont été faites de l’idée de soins de santé primaires dont la formulation initiale souffre de plusieurs imprécisions et ambiguïtés, notamment les expressions « scientifiquement valables », « socialement acceptables », « universellement accessibles », « communautés » et « coût que la communauté et les pays peuvent prendre en charge ». La possibilité d’adapter les pratiques médicales aux conditions locales a été envisagée, tout particulièrement dans les pays à faible revenu. Toutefois, une lecture culturaliste fort optimiste a été faite des modalités de participation des parties prenantes locales en partant d’un consensus local, collectif ou communautaire relativement aux décisions et aux mesures en matière de santé à prendre [30, 31]. L’une des raisons du rejet initial des soins de santé primaires au Burkina Faso est liée à la récupération politique qui en avait été faite par le régime progressiste entre 1983 et 1987, selon une stratégie populiste de l’État central consistant à mobiliser les agriculteurs en délégitimisant le pouvoir des chefferies traditionnelles [32], d’où des conflits entre structures étatiques et leaders traditionnels. Ainsi, les difficultés en contexte burkinabè liées à l’approche des soins de santé primaires, à partir des établissements de soins de santé primaires, étaient de deux ordres : d’une part, les enjeux sociaux liés aux conflits entre le pouvoir central et les chefferies traditionnelles; d’autre part, les conditions défavorables de l’offre qui ne satisfaisait pas les utilisateurs, d’où un faible recours aux ESSP. Cela a abouti à l’abandon de cette stratégie et à une réorganisation des structures du système de santé avec comme référence les CSPS qui sont devenus la structure de premier niveau, en remplacement des ESSP.

Que ce soit les recommandations d’Alma-Ata ou de Bamako, l’idée d’une médecine de proximité est contenue dans les propositions, mais les mesures envisagées, telles qu’elles sont formulées dans ces recommandations, sont fortement influencées par une idéologie dominante concernant la participation communautaire. En principe, l’objectif était que les structures de soins locales soient fonctionnelles et autogérées, en favorisant la participation de la population à leur fonctionnement pour une meilleure prise en charge des besoins de santé. Autrement dit, la question est de savoir dans quelle mesure la population locale peut satisfaire les besoins de santé. La démarche initiale depuis l’avènement de la médecine dans le pays a été la prestation de certains services gratuits pendant la colonisation et les premières années après l’indépendance. Après cette période, la nécessité d’une prise en charge par les utilisateurs locaux de leurs besoins de santé s’est imposée, d’où l’idée de « participation » avec ses connotations idéologiques et les interprétations tendancieuses dont elle a fait l’objet.

La mise en oeuvre de l’Initiative Bamako dans les structures publiques s’est faite au moyen de plusieurs expériences de recouvrement des coûts effectuées de diverses façons, notamment la vente des médicaments, le paiement des prestations à l’acte, la mise en place de forfaits, et la création de systèmes de prépaiement et de mutuelles. Tous ces systèmes ont en commun, d’une part, de chercher à mobiliser des ressources financières pour les structures publiques de soins en raison de la forte diminution de l’enveloppe budgétaire de l’État, et, d’autre part, de ne pas prendre en considération l’incapacité dans laquelle se trouve la majorité des ménages ruraux à faire face au coût des soins calculés sur la base de la tarification en vigueur [33]. Dans la pratique, la tarification mise en place dans les hôpitaux publics est dissuasive pour une grande partie de la population. L’absence de mécanismes opérationnels pour la prise en charge des personnes dans le besoin constitue un autre obstacle à l’accès aux soins des plus pauvres, qui n’ont souvent pas accès aux soins conventionnels. Ce recouvrement des coûts marque une étape importante dans l’évolution des systèmes de santé et des conditions de l’offre de soins. La nouvelle organisation mise en place pour assurer la participation communautaire par l’intermédiaire de comités de gestion était la cheville ouvrière des nouveaux mécanismes basés essentiellement sur la tarification des actes et l’achat de médicaments génériques moins coûteux provenant des dépôts décentralisés. Les conditions de mise en oeuvre de l’IB, les résultats et les enjeux qui ont conduit à un retour progressif vers des exemptions sectorielles ou modulées de paiement ont été bien documentés [34, 35].

Les avis convergent sur le fait que l’IB n’était pas en soi une mauvaise politique, mais qu’elle est intervenue dans un contexte de plus en plus défavorable lié à la fois à la déliquescence des finances publiques et des structures de soins, avec une baisse dramatique de la qualité des soins. Les répercussions sociales étaient donc d’ores et déjà prévisibles, notamment l’exclusion des plus pauvres de ce système de paiement direct des soins. En effet, en dehors d’un système de mutuelles ou d’assurance maladie, les plus pauvres faisant déjà face à des difficultés de survie existentielle sont manifestement incapables de payer les frais qui s’imposent pour les services de santé proposés. Le problème de l’accès aux soins demeure distinct de la participation des membres des comités de gestion qui présente certains avantages fonctionnels pour les services de santé.

En principe, la prestation des soins à titre gratuit fonctionnait de manière similaire à l’époque de l’administration coloniale et des missions chrétiennes en Afrique. En effet, dans tous les cas, il s’agissait d’une gratuité accordée à partir de subventions ou d’allocations externes, que cela relève d’un investissement direct (dons provenant du gouvernement ou d’institutions internationales) ou des bonnes oeuvres (dons et aides multiformes) dans le cadre des missions chrétiennes. Les mécanismes de fonctionnement sont également semblables, puisqu’il s’agissait d’atteindre de diverses manières les utilisateurs sans aucune contrepartie directe. Sur cette base, l’implantation sanitaire s’est faite progressivement dans tout le territoire à partir de ces deux niveaux d’intervention.

Durant la période de l’indépendance des nouveaux États dans les années 1960, la gratuité a fonctionné selon les mêmes principes initiaux, à savoir une gratuité totale entièrement financée par les ressources publiques, avec l’appui additionnel de bailleurs de fonds externes. La dotation en médicaments se faisait à partir d’un magasin central devenu la CAMEG. L’application de la gratuité totale durant cette période a concerné plusieurs niveaux d’accès aux services et de participation des fournisseurs de soins. En effet, l’accès était libre ou obligatoire, en poste fixe ou mobile, pour des besoins particuliers (épidémies ou autres maladies courantes), selon le circuit étatique ou par l’intermédiaire des missions chrétiennes. La gratuité totale était définie comme le non-paiement des actes thérapeutiques, y compris la mise en observation ou l’hospitalisation en cas de besoin, la vaccination préventive, ainsi que l’accès aux médicaments. La gratuité totale des soins accordée au début a forgé une logique d’État providence dont on se rappelle encore aujourd’hui.

En ce qui concerne l’avènement des soins de santé primaires et de l’IB dans les années 1980 à 1990, l’instauration d’une nouvelle organisation fonctionnelle de centres de santé (comités de gestion des centres de santé, dépôts de médicaments et rôle plus affirmé des parties prenantes locales) marque le début de la privatisation des soins. En effet, l’État n’intervient plus directement, sauf pour le financement des infrastructures et des salaires, la formation des professionnels du domaine et l’élaboration de documents fondamentaux. Le désengagement de l’État était dicté aussi par les politiques internationales d’ajustement structurel qui préconisaient que le secteur social soit moins financé par rapport aux secteurs productifs. Ces options ont précédé la situation présente marquée par des contraintes d’accessibilité aux soins pour les couches sociales défavorisées. Le retour à des politiques ciblant des exemptions multiformes s’explique aisément dans un tel contexte.

L’étape actuelle du fonctionnement des services de santé est caractérisée par un retour à des gratuités suscité par la mondialisation des décisions et des mesures en matière de santé [36], et rendu possible par une vaste mobilisation de ressources externes. Cela a été possible grâce notamment aux fonds d’appui (Fonds européen de développement, fonds d’aide aux pays pauvres très endettés, Fonds mondial, etc.) et aux initiatives en lien avec les OMD. Ces initiatives externes de financement de la santé posent la question qui reste sans réponse de la responsabilité des politiques publiques locales, compte tenu de la nécessité de maintenir les engagements financiers assez longtemps pour avoir des effets durables. Le cas du financement de la lutte contre le VIH/sida est un exemple éloquent qui montre comment des structures devenues familières dans le paysage institutionnel sont rattachées directement à l’échelon le plus élevé de l’État (présidence). De ce fait, elles fonctionnent avec des ressources particulièrement importantes provenant des bailleurs de fonds internationaux (en particulier du Fonds mondial) qui échappent à l’usage et au contrôle des instances habituelles du ministère de la Santé. Cela crée un fonctionnement asymétrique de ce ministère avec de nombreux programmes verticaux relativement autonomes, difficiles à coordonner et à mettre en cohérence avec les stratégies sectorielles.

L’autre domaine d’application de la gratuité des soins au Burkina Faso qui fait l’objet de notre revue chronologique a concerné ce qu’il est convenu d’appeler les grandes endémies, plus précisément l’onchocercose, la trypanosomiase, la lèpre et la tuberculose. Outre la description de la contribution inégale de la médecine coloniale à la prise en charge de ces maladies récurrentes par le passé [37], des témoignages mettent en exergue l’engagement des parties prenantes en cette période de grands défis sanitaires et d’interventions diverses [38]. Les pratiques évoquées renvoient également aux épidémies [39] qui ont fait beaucoup de ravages et dont certaines perdurent aujourd’hui (méningite, rougeole et fièvre jaune). Il s’avère que l’importance des actions significatives dans le processus d’implantation médicale a constitué un argument favorable à l’utilisation des soins biomédicaux, malgré les réticences manifestées au début qui ont conduit parfois à faire preuve d’habileté (p. ex. équipes mobiles) ou de force, selon les circonstances.

La lutte contre les grandes endémies a constitué le point de départ de l’action sanitaire au moment où elles étaient de nature à réduire à néant les efforts de développement colonial. Qu’il s’agisse de la trypanosomiase, de la tuberculose, de la lèpre ou de l’onchocercose, ces maladies redoutées constituaient une véritable menace à la fois pour la population constamment exposée et pour l’atteinte des objectifs de production en contexte local visant cette population, d’où les efforts déployés pour les éradiquer. La réponse apportée dans le cadre de la politique coloniale a été positivement appréciée pour son efficacité et a bâti depuis cette période la renommée de la médecine moderne comme disposant des moyens pour arrêter la propagation des épidémies qui décimaient la population. Il en va de même pour les mesures de prévention de certaines de ces maladies par la vaccination de masse. Cependant, ces campagnes étaient essentiellement orientées vers l’arrêt des maladies à fort potentiel épidémique, à la différence d’autres affections comme le paludisme qui persistent encore aujourd’hui.

L’exemple du programme de lutte contre l’onchocercose illustre la contribution importante des interventions initiées dans le cadre de la lutte contre les grandes endémies. C’est incontestablement l’un des plus grands projets sanitaires réalisés en Afrique. Lancé en 1974 pour enrayer la maladie dans une zone couvrant initialement sept pays (le Bénin [anciennement le Dahomey], le Burkina Faso [anciennement la Haute-Volta], le Ghana, le Mali, le Niger et le Togo), le programme a été étendu par la suite à quatre autres pays (Guinée, Guinée-Bissau, Sénégal et Sierra Leone). Plus de 30 ans après son démarrage, il est considéré comme l’un des programmes régionaux de santé les plus efficaces jamais mis en oeuvre. Cette intervention sanitaire de grande envergure a profité du parrainage des grandes institutions de développement, notamment l’Organisation des Nations-Unies pour l’alimentation et l’agriculture, le Programme des Nations-Unies pour le développement, l’OMS et la Banque mondiale. Cette participation se justifiait par le double objectif sanitaire et économique qui était poursuivi, à savoir, d’une part, éradiquer cette maladie transmissible, et d’autre part, promouvoir le développement économique des zones libérées de la maladie. Le défi sanitaire a été relevé puisqu’actuellement, l’onchocercose n’est plus un danger pour la santé publique dans les pays de la zone initiale d’intervention.

La réalité d’antan était que plusieurs villages étaient abandonnés à cause de la forte prévalence de la maladie dans les régions les plus exposées, notamment dans les zones riveraines fortement infestées par l’onchocercose. Cependant, les résultats de plusieurs études [40] indiquent que les abandons des villages n’étaient pas seulement attribuables à des causes sanitaires, comme les flambées d’onchocercose. En effet, deux types d’événements sont à différencier : d’une part, les mouvements de population non spécifiques aux zones touchées par l’onchocercose qui sont caractéristiques d’une évolution démographique régionale liée à des raisons autres que sanitaires, et d’autre part, pour les zones résidentielles qui étaient infestées par l’onchocercose, la prévalence de la maladie qui était certainement l’une des causes d’abandon des villages dits de « première ligne ».

Une fois que les exemptions de paiement des soins ont pris fin pendant la période coloniale et postcoloniale (jusqu’aux années 1970), l’accès aux soins est demeuré gratuit dans le cadre de programmes verticaux seulement pour quelques maladies ciblées, y compris la tuberculose, la lèpre et l’onchocercose. Bien que ces grandes endémies aient été éradiquées à vaste échelle, elles étaient toujours présentes, mais à une faible prévalence. Cela s’applique également à la vaccination de masse contre les épidémies et au suivi vaccinal des femmes en âge de procréer et des nourrissons. L’accès est également gratuit pour les consultations prénatales et postnatales, les soins préventifs contre le paludisme et le traitement du paludisme grave. La vague d’exemptions partielles de paiement dans les années 2000 complète ce panorama des mesures prises pour améliorer l’accès aux soins des utilisateurs. Les années 2000 représentent indéniablement pour plusieurs pays d’Afrique subsaharienne un tournant décisif quant à la mise en place de politiques d’exemption de paiement des soins pour les couches vulnérables de la population. En raison des limites et de l’échec du recouvrement des coûts préconisé par l’Initiative de Bamako [6] et à la suite des recommandations de la Conférence d’Alma-Ata sur les soins de santé primaires, plusieurs mesures d’exemption ont été mises en place. Au Burkina Faso, ces initiatives ciblaient initialement les femmes enceintes et les enfants de moins de cinq ans, puis certaines ont été étendues aux autres catégories d’utilisateurs.

En recoupant les différentes sources de données exposées, ce sont des préoccupations d’ordre conjoncturel qui ont conduit à l’introduction de nouvelles interventions basées sur les exemptions sectorielles de paiement pour éviter l’exclusion de l’accès aux soins primaires des plus pauvres. Le sujet du présent article découle des opinions exprimées à propos de l’adoption de politiques privilégiant l’un ou l’autre pôle d’exemption de paiement des frais de santé (gratuité ou subvention). C’est pourquoi la question est abordée en mettant en perspective les points de vue contrastés des différentes parties prenantes (décideurs politiques et professionnels de la santé) privilégiant l’une ou l’autre de ces options, plutôt que les points de vue et les opinions des communautés bénéficiaires qui plaident naturellement en faveur de l’exemption totale de paiement des soins. Des précisions sont données dans la section suivante sur l’émergence et l’élaboration des exemptions pour les interventions abordées dans les deux autres études.

## Émergence et élaboration des exemptions sectorielles liées au paludisme et au VIH/sida

### Cas du paludisme (deuxième étude)

À l’instar d’autres services et maladies, plusieurs mesures d’exemption partielle ou totale de paiement des soins liés au paludisme ont été adoptées au Burkina Faso ces douze dernières années. Il est question ici de la subvention des ACT depuis 2006 et de la distribution gratuite des moustiquaires imprégnées d’insecticide à longue durée d’action (MILDA).

Sous l’angle chronologique, des mesures antérieures précèdent l’adoption des exemptions actuelles de paiement des soins pour le paludisme. Il y a d’abord eu la création de deux centres pour la recherche et les interventions sur le paludisme, soit à Bobo-Dioulasso (deuxième ville du pays) en 1941, puis à Ouagadougou en 1987. Ensuite, le pays a souscrit à plusieurs recommandations internationales, notamment celles de la conférence de l’Union africaine à Abuja en 2000 ainsi que de l’initiative Faire reculer le paludisme en 2002, ce qui a donné lieu à la mise en place de plans d’action et de programmes particuliers. Récemment, les requêtes du Burkina Faso pour la composante portant sur le paludisme aux rondes 7 (2007) et 8 (2008) du Fonds mondial de lutte contre le VIH-sida, la tuberculose et le paludisme ont permis d’obtenir des fonds importants pour plusieurs formes d’exemption de paiement des soins (48 millions USD de 2003 à 2008 [41]). La forte participation de cette organisation et d’autres institutions de coopération a permis d’instaurer la gratuité totale pour certains services.

La décision de subventionner les ACT a été prise depuis le remplacement de la chloroquine en 2003 à la suite de la publication de directives par l’OMS. Or, le prix initial de vente locale fixé à 3 000 FCFA (6 USD) était jugé élevé, et aucun médicament générique moins onéreux n’était disponible. En 2006, la Banque mondiale a commencé à verser des fonds permettant de subventionner le traitement (100 FCFA ou 0,2 USD) pour les enfants de moins de cinq ans, alors que la gratuité des traitements du paludisme grave avait déjà été adoptée. Par la suite, l’application de la subvention aux adultes a été rendue possible grâce aux ressources acquises lors de la ronde 8 du Fonds mondial, ce qui a permis le passage à grande échelle, avec une composante communautaire, de la distribution par des agents de santé communautaire de MILDA et de médicaments subventionnés. L’accord entre la Banque mondiale et l’institution nationale chargée de gérer le « panier commun » de fonds externes (Programme d’appui au développement sanitaire) pour l’exécution des interventions avant les rondes 7 et 8 du Fonds mondial prévoyait que le recouvrement des coûts serait utilisé uniquement pour financer le renouvellement des intrants, et non pour d’autres besoins, comme l’équipement ou la gestion du personnel.

L’un des défis majeurs au début de la mise en oeuvre était de pouvoir constituer un stock suffisant de médicaments pour satisfaire d’abord les besoins initialement pris en charge (traitement des femmes enceintes et des enfants de moins de cinq ans), puis la demande générée par l’application des interventions à l’ensemble de la population. Il s’avère que le stock initial pour la première composante de l’intervention (financement de la Banque mondiale pour les femmes enceintes et les enfants de moins de cinq ans, puis celui du Fonds mondial pour la vente communautaire subventionnée) a pu être constitué selon les prévisions. Par contre, des difficultés d’approvisionnement en ACT compliqueront le passage à l’échelle (à partir de 2008) à cause de l’insuffisance d’ACT sur le marché international et du fait que la distribution selon le principe du « panier commun » ne respectait pas les disponibilités par source de financement.

Par ailleurs, deux types d’interventions d’ordre préventif concernant la distribution gratuite de MILDA ont été mises en place dans la même période. La première concerne la mise à disposition de MILDA dans les districts sanitaires pour les femmes enceintes venues en consultations prénatales à la suite de la décision officielle prise en 2002 d’instaurer la gratuité des soins préventifs pour les groupes vulnérables. À partir de 2008, une application aux enfants de moins de cinq ans sera possible grâce aux subventions du Fonds mondial et d’autres organisations de coopération, notamment la Croix-Rouge canadienne. Quant à la deuxième mesure concernant la distribution gratuite de MILDA en 2010 à l’échelle du pays, elle a pu être mise en place grâce au financement acquis dans la ronde 8 du Fonds mondial (26 milliards FCFA) pour le passage à l’échelle de cette intervention. La participation d’une ONG (Plan Burkina, maintenant le Plan international) et d’acteurs régionaux (quatre ONG nationales) indépendants du système de santé était une expérience nouvelle de partenariat public-privé.

On retiendra que la mise en place soudaine des exemptions à la carte, sans préparation suffisante des parties prenantes, a été faite en l’absence de dispositions officielles appropriées, contrairement aux mécanismes existant pour d’autres interventions (p. ex. gratuité du traitement antituberculeux). Des documents de référence ont également été produits dans le processus de préparation et de formulation de la subvention des soins de santé maternelle décidée en 2006.

### Exemptions sectorielles liées à la tuberculose et au VIH/sida (troisième étude)

**TB** Depuis les années 1950, certaines maladies endémiques comme la tuberculose sont prises en charge gratuitement. Dans les années 1980, les enjeux politiques et sociaux liés à la pandémie du VIH ont contraint progressivement les États à instaurer la gratuité des médicaments antirétroviraux (ARV). La gratuité du traitement de la tuberculose était en vigueur depuis la période coloniale dans le contexte de la lutte à grande échelle contre les grandes endémies [42]. Comme pour d’autres maladies, cette gratuité s’est poursuivie après l’indépendance (1960) afin de faciliter l’accès au traitement, mais l’incidence de la tuberculose a augmenté avec l’apparition du VIH.

En ce qui concerne le sida, la gratuité des médicaments ARV a été instaurée en 2009 au Burkina Faso, soit beaucoup plus tard que dans d’autres pays comme le Sénégal (2003) et le Mali (2004). Le problème de l’accès financier au traitement s’est imposé depuis la mise en circulation des premiers médicaments efficaces contre la maladie en 1996. Il est reconnu que c’est le militantisme associatif en faveur de l’accès aux médicaments dans les pays du Sud qui a permis de réduire considérablement les prix des médicaments ARV et de les offrir gratuitement par la suite dans la plupart des pays d’Afrique subsaharienne.

La gratuité du traitement de la tuberculose a connu deux grandes phases qui montrent l’évolution des interventions publiques dans ce domaine. La première phase concerne la période coloniale tandis que la deuxième, qui est en cours, est basée sur les recommandations de l’OMS sous la coordination du Programme national de lutte contre la tuberculose (PNT). Outre la description de la contribution inégale de la médecine coloniale à la prise en charge de ces maladies récurrentes par le passé [37], des témoignages mettent en exergue l’engagement des parties prenantes en cette période de grands défis sanitaires et d’interventions diverses [38]. Plus tard, à partir de l’indépendance en 1960, le traitement est assuré par les centres de lutte antituberculeux, principalement dans les deux premières villes et des centres moins importants dans cinq villes secondaires (Koudougou, Ouahigouya, Fada N’Gourma, Dédougou et Banfora). Vu la faible décentralisation du traitement, les malades devaient parcourir de longues distances pour se faire diagnostiquer et traiter à une période où le traitement n’était pas encore standardisé. Pendant cette période, la gratuité était offerte pour l’hospitalisation, ainsi que les examens de laboratoire et de radiologie au cours du traitement et de la surveillance, comme l’évoquent les principaux documents officiels. Cependant, l’expérience montre que l’application des mesures d’exemption a connu des dysfonctionnements liés au manque d’intrants et à l’absence d’une ligne budgétaire réservée au traitement de cette maladie. Les patients devaient donc payer certains frais, mais le montant différait selon les structures. En plus des médicaments, certains examens, notamment la radiographie des poumons, étaient payants, ce qui traduit le décalage entre les dispositions et la pratique.

Avec la création du PNT en 1995 commence une autre étape avec une meilleure organisation de la prise en charge et une application concrète de la gratuité des soins. Bénéficiant d’un appui de l’OMS, d’autres partenaires et de l’État pour la subvention des médicaments, le PNT dispose de ressources plus importantes pour la mise en application des directives. Son rôle est de coordonner les activités dans les différentes structures de diagnostic et de traitement aux différents échelons du système de santé national (directions régionales de la santé, districts sanitaires, et centres de diagnostic et de traitement du paludisme). En outre, il bénéficie de l’appui des centres de référence pour la prise en charge médicale des cas de résistance aux antituberculeux, du Programme d’appui au monde associatif et communautaire, d’associations de thérapeutes traditionnels et d’anciens patients.

**VIH/sida** Progressivement mise en place, l’exemption de paiement pour le VIH/sida s’appliquait d’abord aux enfants grâce à l’ONG ESTHER qui avait lancé en 2003 un programme pilote de prise en charge médicale de 200 enfants répartis entre trois centres médicaux des villes de Ouagadougou (n = 91), de Bobo-Dioulasso (n = 30) et d’Ouahigouya (n = 17). En 2008, une décentralisation progressive de la prise en charge médicale dans certains hôpitaux régionaux, hôpitaux de district (centres médicaux avec antennes chirurgicales) et centres médicaux associatifs a été entreprise. L’approvisionnement en médicaments ARV se faisait auprès du Comité interministériel de lutte contre le sida. Par ailleurs, les structures associatives apportent de l’aide aux parents au moyen de l’offre de préparation pour nourrissons, de l’achat de médicaments contre les infections opportunistes et du paiement des frais d’examens biologiques. Les fournisseurs de soins indiquent que les médicaments ARV sont gratuits [43], mais tel n’est pas le cas pour l’hospitalisation, les examens biologiques et les médicaments contre les infections opportunistes dont les coûts diffèrent d’une structure à l’autre.

La gratuité des médicaments ARV pour les adultes a été déclarée à la fin de 2009, en réponse à une forte pression exercée par de nombreuses associations de lutte contre le sida organisées en réseaux très actifs. Elles ont été aidées par d’autres actions convergentes, notamment le plaidoyer initié par Amnistie internationale en 2009 pour la gratuité des soins de santé maternelle [44]

## Gratuité ou subvention : les arguments contrastés des acteurs et des utilisateurs

Les perceptions des différentes formes d’exemption de paiement des frais ne sont pas étrangères aux opinions courantes véhiculées sur l’idée de « gratuité » en général. Il en va de même pour les perceptions des effets induites par les directives officielles. Il est apparu des repères chronologiques susmentionnés que la gratuité totale a été introduite pendant la colonisation, puis modulée après l’indépendance. Les significations courantes du mot « gratuité » dans l’une des trois principales langues véhiculaires, comme l’a rappelé un répondant clé, illustrent les perceptions négatives qui y sont rattachées. En effet, les connotations sémantiques du terme *zaalem* en langue mooré, soit *néant, rien, cadeau* ou *sans raison* [45], reflètent certaines valeurs culturelles traditionnelles. Comme l’a précisé un autre répondant clé, [traduction] « on se dit que quand quelque chose est gratuit, ce n’est pas bon, mais que quand on paye, c’est bon » (enseignant du primaire). Selon ces indicateurs de l’influence des traditions locales, l’individu tient à assurer lui-même les soins liés à sa santé et à celle de ses proches, ou au moins à y contribuer pour préserver sa dignité humaine. Il en va de même pour les besoins essentiels. Il est question ici de l’influence toujours présente de certaines traditions locales qui ne dictent toutefois pas la conduite au sein de la population malgré les changements continuels liés à la modernité et aux mesures de développement multiformes [46].

Les réactions se recoupent sur certains aspects, notamment les critiques fréquentes contre une gratuité totale à la différence des subventions incluant une petite contrepartie de l’utilisateur. Ainsi, à l’image des acteurs du système de santé qui ont des réactions semblables, la prestation de soins entièrement gratuits sans une contrepartie symbolique des bénéficiaires est remise en question en se référant pour certains aux significations culturelles et pour d’autres aux perspectives de pérennisation difficilement envisageables selon eux sans un apport des bénéficiaires. Dans le même ordre d’idées, un répondant a dit en parlant d’une initiative liée à l’agriculture [traduction] :

« Même si c’est 10 francs, ou encore 25 francs, il faut que ça serve à éduquer, à donner un sens, de la valeur aux semis; même en reboisant, on dépense quelque chose. Ça coûte quelque chose, ça a un prix; même si c’est subventionné, il ne faut pas encourager l’idée que tout est gratuit » (ancien décideur politique et responsable d’une institution publique).

Par ailleurs, un constat fait à la fois par les fournisseurs de soins et certains répondants évoque le mauvais usage de la gratuité, selon l’idée que ce qui est offert gratuitement a une moindre valeur. Ainsi, certains comportements décrits sont contraires aux attentes ou aux objectifs établis, notamment une utilisation abusive des services de santé dans certains cas ou, au contraire, une méfiance ou une réticence manifeste, la non-utilisation des comprimés dont les vertus thérapeutiques ne sont pas perçues comme légitimes (le cas des médicaments génériques est un exemple connu), ainsi que le gaspillage des intrants agricoles subventionnés (le cas des semences agricoles données aux animaux est illustratif). Ce genre de comportements marginaux est certainement observé, mais il faut relativiser à cause des effets positifs sur la majorité des utilisateurs respectueux des prescriptions et des avantages à grande échelle relevés par les défenseurs de la gratuité.

Un raisonnement semblable est fait en présentant la gratuité comme étant incompatible avec une pérennisation des interventions ou en remettant en question la « responsabilité » des bénéficiaires de la gratuité totale [13]. Des opinions semblables sont formulées par des parties prenantes déçues par les mauvais usages de la gratuité ou ses effets pervers difficiles à maîtriser (mises en oeuvre sans mesures adéquates de soutien, manque d’intrants, insuffisance de personnel et d’équipement, et menaces supposées à la solvabilité des ressources publiques). À l’inverse, les défenseurs avisés, mais moins nombreux de la gratuité sélective de certains services défendent le bien-fondé d’un accès équitable aux soins en favorisant une exemption totale ou plus importante pour les populations défavorisées, à la différence des autres catégories d’utilisateurs. Il y a aussi les utilisateurs eux-mêmes, dont certains sont nostalgiques de l’État providence [47], qui attendent au moins un allègement des coûts d’accès aux services de santé, à défaut d’une suppression. Ces résultats montrent que les fournisseurs de soins et les autres catégories de répondants clés ont des perceptions contrastées sur l’exemption totale de paiement des soins. Pour les uns, elle permet l’accès des plus pauvres aux services de santé, tandis que pour les autres, la gratuité n’est pas bien comprise et est dévalorisée par les utilisateurs des services de santé, d’où l’évocation de la subvention et d’une contribution, aussi minime soit-elle, des bénéficiaires.

## Certains répondants qui rejettent la gratuité totale envisagent d’autres options, notamment :

1. Une approche progressive dans le cadre de laquelle les tarifs sont établis en fonction de la capacité de payer des utilisateurs et des capacités limitées de l’État, mais aussi de l’exigence de maintenir un niveau satisfaisant de qualité des soins, comme l’illustrent les propos suivants [traduction] : « Certaines personnes n’ont vraiment rien, et pour elles, ça devrait être gratuit. Mais la gratuité ne devrait pas s’appliquer à tout le monde. L’État ne peut pas assumer cela. J’espère toutefois, en disant que je suis contre la gratuité, que les services de santé pourront améliorer leur prestation et leurs structures » (enseignant et responsable d’une organisation de la société civile).
2. Une approche sectorielle et temporaire axée sur les maladies nécessitant la prise de mesures rapides est proposée par un autre répondant [traduction] :

« Je pense qu’on pourrait réduire davantage les coûts et essayer de lutter contre les maladies les plus meurtrières, comme le paludisme et le VIH. On pourrait subventionner cette lutte entièrement le temps de contrôler et d’éliminer la maladie, et ce, jusqu’à ce que les tarifs normaux puissent être réappliqués » (responsable d’un programme sanitaire national proposant une exemption des frais).

1. Une formule consistant à subventionner les médicaments et incluant des mécanismes de protection sociale est suggérée par l’un des répondants clés [traduction] :

« La formule de subvention des médicaments est une bonne formule; des mutuelles et d’autres options d’assurance maladie sont mises en place un peu partout. Je pense que ça se rapproche de la recherche d’une solution avec les populations en vue de leur donner la capacité de payer » (ancien décideur politique et responsable actuel d’une institution publique).

À l’inverse, d’autres arguments ont été exprimés en faveur de la gratuité totale, en présentant notamment les inconvénients d’une exemption partielle de paiement en lien avec l’accès équitable aux soins [traduction] :

« Vous savez quel est le problème avec les subventions partielles par rapport à la gratuité ? C’est la complexité des subventions partielles. Des problèmes d’équité se posent, parce que les critères d’obtention de telles subventions, les taux de subvention, les critères et autres ont toujours été très complexes » (professionnel de la santé et responsable d’une ONG sanitaire).

En outre, contrairement aux conceptions morales de la gratuité inspirées des traditions locales, les utilisateurs veulent et même réclament autant que possible l’accès gratuit aux soins, certains évoquant avec nostalgie la gratuité de l’État providence du passé. Dans une situation d’exemption à la carte, certains utilisateurs voudraient que les options d’exemption soient harmonisées de sorte que la gratuité totale déjà appliquée aux soins préventifs du paludisme englobe les soins curatifs. Cependant, certains effets pervers sont imputables à la fois aux fournisseurs de soins, aux utilisateurs et au mauvais fonctionnement du système de santé. Premièrement, il a été relevé par exemple que les critères d’attribution des moustiquaires imprégnées d’insecticide aux femmes à l’occasion des consultations prénatales (selon le niveau d’utilisation ou d’autres conditions officieuses) diffèrent d’une structure à l’autre, ce qui ouvre la voie aux abus de certains fournisseurs de soins mal intentionnés. Cette situation a également été observée lorsqu’il a été décidé que les centres de santé distribueraient gratuitement des médicaments aux pauvres. Cette décision avait été prise par le ministère de la Santé en 2005 sans qu’aucun critère officiel d’admissibilité ne soit établi. Les agents de santé avaient donc dû improviser et faire preuve d’initiative [48]. En outre, plusieurs cas de vente par des utilisateurs indélicats de moustiquaires distribuées gratuitement ont été signalés. Il en va de même pour le recours non justifié aux centres de santé par certains utilisateurs pour obtenir des médicaments qui seront ensuite distribués à des amis, des membres de la famille ou d’autres personnes.

Ces pratiques, qui s’éloignent de l’esprit humaniste d’une gratuité permettant de lever les obstacles financiers à l’accessibilité aux soins, surtout pour les personnes pauvres, montrent la nécessité de réformes supplémentaires permettant au système de santé d’atteindre les objectifs ciblés en minimisant autant que possible les effets pervers. Cela suppose d’avoir recours à une approche plus systémique et non verticale en s’éloignant de certaines prescriptions du paradigme pasteurien [14].

Les résultats des trois études confirment qu’il existe des appréciations divergentes entre les fournisseurs de soins qui sont généralement défavorables à la gratuité totale et les utilisateurs qui expriment leur adhésion à la gratuité totale à grande échelle et leur satisfaction envers celle-ci. Il existe également des différences entre les professionnels de la santé qui n’admettent pas le principe d’une gratuité sans contrepartie qu’ils jugent difficile à pérenniser et ceux qui l’acceptent en suggérant d’introduire des critères pour un accès équitable en fonction des statuts sociaux et économiques. Dans ce cas, une attention est accordée aux personnes pauvres qui sont censées profiter de l’exemption totale de paiement des soins, à la différence des autres catégories de personnes pour lesquelles il est établi que l’exemption applicable doit être proportionnelle au niveau de revenu.

Certaines spécificités se dégagent pour les interventions relatives au paludisme, à la différence de celles liées au VIH/sida. Au sujet des exemptions de paiement pour les soins préventifs et curatifs du paludisme, deux aspects sont mis en relief. Il y a d’abord la préférence des parties prenantes (décideurs et fournisseurs de soins) pour la subvention. Ensuite, plusieurs répondants ont relevé l’imprécision des mesures prises ou l’absence de directives claires pour leur application qui sont à l’origine de dysfonctionnements multiformes pendant la mise en oeuvre et viennent s’ajouter aux dysfonctionnements courants du système de santé [29]. Cela montre la difficulté d’inclure ces mesures dans des politiques publiques stabilisées et répondant à des exigences d’efficacité fonctionnelle [49] ou sensibles à des réformes de proximité [50].

Pour la tuberculose et le VIH/sida, il semble que la quasi-totalité des malades interrogés ont profité de la gratuité des examens de contrôle, des médicaments antituberculeux et de l’hospitalisation, comme il est indiqué dans les documents de référence. La situation est différente pour le VIH/sida, car des retards ont été rapportés dans la mise sous traitement, des diminutions d’affectation normale des intrants ont été observées, et le paiement de médicaments pour les infections opportunistes, ainsi que le paiement de frais d’examens biologiques qui sont pourtant gratuits, ont été exigés. De nombreuses pénuries de médicaments et de réactifs et des pannes d’appareil pour les examens ont été également signalées par les fournisseurs de soins.

# Discussion

Une comparaison entre différentes caractéristiques des interventions considérées est présentée dans le tableau 2.

Les éléments suivants ont servi de base de comparaison :

1. La nature de l’exemption totale (gratuité) ou partielle (subvention à des degrés variables)
2. La pathologie (TB, VIH/sida ou paludisme)
3. L’approche visée (préventive ou curative)
4. Le groupe cible
5. L’existence de documents officiels pour la mise en oeuvre
6. L’engagement budgétaire (État et partenaires internationaux)
7. Les modalités d’accès à l’information sur l’intervention et les services offerts

Il ressort de l’analyse chronologique et typologique que, malgré les efforts de l’État pour assurer une extension spatiale des infrastructures sanitaires [51], certaines régions sont défavorisées dans la répartition globale. En effet, des difficultés structurelles liées à l’offre de soins s’ajoutent aux risques situationnels (financements irréguliers, manque d’intrants, et manque d’infrastructures, d’équipement et de personnel). Vue sous cet angle, la subvention nationale des soins liés au paludisme profite d’une plus grande couverture en matière d’offre de services à tous les échelons du système de santé et à l’échelle communautaire, y compris la prise en charge à domicile du paludisme. En revanche, l’offre de services liés au VIH/sida et à la tuberculose est plus limitée dans l’espace (cas des structures de soins autorisées). De ce fait, il y a une inégalité d’accès aux soins entre les zones disposant d’établissements traditionnels et celles qui en ont moins.

Les expériences décrites montrent les vastes possibilités offertes à l’État burkinabè de financer des exemptions ponctuelles de paiement pour des services ciblés à partir de subventions importantes accordées par des organisations internationales. L’émergence soudaine et la formulation asymétrique des exemptions prises en compte dans les études illustrent les difficultés d’instauration d’une politique d’exemption pouvant intégrer à la fois la variété des services couverts ainsi que la prise en considération des besoins des groupes cibles selon la nature de l’intervention. L’existence d’un financement pérenne (cas de la tuberculose) assuré depuis longtemps par des partenaires et l’État burkinabè tranche avec les ressources ponctuelles accordées pour la plupart des exemptions de paiement à partir des financements octroyés par des organisations internationales. Même si la question de la pérennité [52] doit être mise en perspective en accordant beaucoup plus d’attention aux résultats de santé en faveur notamment des personnes pauvres et du renforcement du système de santé, l’existence d’une ligne budgétaire distincte pour une exemption particulière est un atout important. Il en va de même pour l’existence de textes réglementaires ou législatifs (cas de la tuberculose pour les deux aspects évoqués, contrairement aux autres interventions relatées) qui constitue une différence importante dans la typologie des régimes d’exemption, selon notre perspective d’analyse.

À l’exception de la tuberculose, du paludisme et du VIH/sida, les décisions d’exemption de paiement des soins ont été prises soudainement à la suite de faits majeurs. Premièrement, la décision de l’OMS d’utiliser un nouveau médicament pour le traitement du paludisme, deuxièmement, la pression des ONG et des organismes communautaires ainsi que des organisations multilatérales (notamment l’OMS et la Banque mondiale) pour le passage à la gratuité (cas des médicaments ARV pour les adultes), et troisièmement, les possibilités de financement offertes par les bailleurs de fonds, en particulier le Fonds mondial. La conjonction de ces facteurs a été décisive pour l’avènement concomitant de plusieurs exemptions sectorielles de paiement. L’importance des fonds mobilisés sur une période relativement courte rappelle les expériences entreprises antérieurement pour la lutte contre les grandes endémies. En revanche, les enjeux étaient différents, tant sur le plan de la nature des interventions que de l’incidence sur le fonctionnement du système de santé. En effet, pour les grandes endémies, il s’agissait de campagnes d’éradication dans des zones déterminées à partir de programmes verticaux disposant de ressources humaines et financières non directement intégrées au système de santé. Il en va autrement pour les exemptions entreprises avec les recommandations internationales dont la formulation et la mise en place relèvent de l’État. Celles-ci sont enchâssées dans le système de santé et présentent des lacunes et des difficultés dans la mise en application à cause d’une préparation insuffisante et de ressources matérielles et humaines souvent inadaptées.

Le constat général est que les exemptions sont mises en place sans évaluation préalable des besoins des structures de santé concernées, notamment en personnel, en infrastructures et en équipement approprié pour pouvoir répondre aux nouvelles exigences. La situation est différente pour les interventions liées à la tuberculose qui sont réalisées dans des structures spécialisées mises en place depuis le début de la lutte contre les grandes endémies. Cela contribue à diminuer les capacités de plusieurs établissements de soins qui ne peuvent profiter d’un appui immédiat de l’État ou d’occasions de financement pour renforcer leur capacité à répondre aux nouvelles exigences techniques. Certes, le ministère de la Santé fournit les infrastructures et l’équipement, mais selon un calendrier en décalage avec les besoins liés à des interventions particulières, comme les mesures d’exemption de paiement souvent prises soudainement et appliquées rapidement malgré les contraintes déjà indiquées.

Dans certains cas (médicaments ARV et TB), l’exemption ne prend en charge qu’une partie des coûts (médicaments ARV pour le VIH/sida, mais pas les frais de suivi biologique) ou ne commence qu’après confirmation du diagnostic à la suite d’un processus coûteux (cas de la TB). Dans ces cas, les effets négatifs concernent davantage l’accès aux services des personnes pauvres qui, contrairement à leurs attentes, doivent souvent payer des frais indirects non prévus qui peuvent être catastrophiques et qu’elles n’ont pas les moyens d’assumer [53].

Théoriquement, l’allègement ou la suppression totale du paiement des soins devrait profiter d’abord aux personnes pauvres, qui sont incapables de payer les frais de santé. À ce sujet, nous avons relevé deux situations contrastées. Premièrement, les prestations liées aux traitements préventifs et curatifs du paludisme dans le cadre des interventions étudiées (distribution de moustiquaires imprégnées d’insecticide et d’ACT dans les centres de santé et parmi les parties prenantes des communautés) bénéficient d’un circuit de distribution étendu et bien accessible à la demande. En revanche, celles liées aux interventions sur le VIH/sida et la TB (examens biologiques, distribution des médicaments ARV et suivi des patients) sont concentrées dans les structures spécialisées ou autorisées (hôpitaux de district et hôpitaux régionaux ou nationaux, ou centres de traitement ambulatoire du VIH/sida, et centres de dépistage et de traitement de la TB). C’est dire que l’accès aux prestations est d’abord limité par la disponibilité géographique des services de santé, ce qui, dans un pays comme le Burkina Faso, montre des disparités importantes entre les zones urbaines et les zones rurales et repose sur une mise en place inégale de structures de santé nationales.

En ce qui a trait aux conditions d’accès aux prestations de soins, d’autres contraintes existent pour les utilisateurs des services de santé, notamment les pauvres. Les exemptions ont pour but d’éliminer ou d’amoindrir les obstacles en matière d’accessibilité financière. Cependant, il apparaît que l’application des mesures rapportées expose les femmes et les enfants admissibles aux dysfonctionnements habituels du système de santé qui ne permettent pas toujours d’assurer un accès efficace à la gratuité des prestations. Cela signifie que, paradoxalement, il n’est pas toujours possible pour les utilisateurs d’avoir vraiment accès à la gratuité des soins pour au moins quatre raisons [11]. En effet, la gratuité est loin d’être totale en raison des lacunes suivantes : 1) les coûts directs non pris en charge officiellement dans le régime de prestations gratuites, 2) les coûts indirects (transports, frais divers non liés aux soins assumés par la famille et les amis), 3) les coûts illicites (factures indues, cadeaux aux agents de santé, vente illicite de médicaments ou autres faveurs demandées frauduleusement en contrepartie de l’accès aux soins), et 4) les achats imposés aux utilisateurs en raison de ruptures de stock de médicaments gratuits ou d’autres intrants. Il est question ici des difficultés qu’éprouvent les utilisateurs à accéder aux soins dans des structures répondant aux normes requises. Ainsi, l’étude sur les médicaments ARV et les prestations associées a montré que les patients doivent se déplacer de leurs domiciles, souvent éloignés des centres de soins, ce qui nécessite de faire face à des coûts indirects liés au transport et à l’hébergement pendant le traitement, ainsi qu’à des frais liés à certains services payants et autres durant le séjour (alimentation et hébergement). Ces dépenses peuvent être catastrophiques pour les plus démunis.

Concernant le financement des exemptions de paiement relatives aux interventions étudiées, l’expérience montre que deux modalités principales mises en application ont des répercussions sur le fonctionnement du système de santé. En effet, l’exemption totale ou partielle de paiement financée principalement par le budget de l’État (cas de la TB parmi les études considérées et d’autres interventions, comme les consultations prénatales et postnatales pour les femmes, la vaccination pour les enfants de cinq ans et moins et les femmes enceintes, et la prise en charge du paludisme grave) est différente de l’exemption totale ou partielle de paiement financée principalement par des organisations internationales avec une petite contribution habituellement demandée à l’État. Dans le premier cas, il s’agit d’un financement stable et durable (depuis la période coloniale pour la TB) prenant souvent appui sur des documents officiels (c’est le cas plus récemment de la subvention des accouchements et des soins obstétricaux et néonatals d’urgence). Cela permet d’effectuer un suivi et des évaluations à long terme pour évaluer notamment les effets sur les destinataires et le système de santé. Dans le deuxième cas, on retrouve les demandes successives de financement au Fonds mondial pour le passage à l’échelle de la lutte contre le VIH/sida, la tuberculose et le paludisme depuis une dizaine d’années. Il s’agit d’un financement temporaire qui suscite des interrogations sur la possibilité ou non de poursuivre les interventions après la fin de l’engagement des bailleurs de fonds.

C’est autour de ces actions que se clarifie le débat entre les partisans de l’exemption totale de paiement et ceux qui misent plutôt sur la subvention. Ainsi, l’idée d’État providence revient dans les esprits et l’absence de contribution des bénéficiaires est remise en question en évoquant une moindre « responsabilité » et d’autres arguments parfois inspirés de conceptions culturelles. Contrairement aux effets pervers de la dépendance à l’aide [54], les initiatives récentes d’exemption exclusivement couvertes par le budget de l’État montrent qu’il existe des solutions endogènes au financement d’interventions importantes dans le domaine de la santé ou d’autres domaines. En effet, on observe un engagement sans précédent depuis mars 2016 en faveur de la gratuité totale de paiement des services de santé offerts aux mères et aux enfants (gratuité des soins pour les femmes enceintes et les enfants de moins de cinq ans, gratuité des césariennes, et gratuité du traitement des fistules obstétricales). Dans ce contexte, la question de fond ne porte plus sur la pérennité des interventions, mais bien sur la capacité de l’État à maintenir ce niveau d’engagement financier avec les quotas budgétaires alloués à la santé (7,29 % du budget de 2015). En effet, une volonté politique est affichée, mais plusieurs défis, dont l’introduction de l’assurance maladie universelle, sont difficiles à relever pour un pays à faible revenu bénéficiant d’un appui budgétaire important de l’aide internationale.

Un autre aspect est que les moins bien nantis sont moins enclins à avoir recours par eux-mêmes aux services de santé, même lorsque les soins sont gratuits. À ce sujet, une recherche-action menée en zone rurale [48] illustre les effets de l’exclusion sociale plus répandue que la stigmatisation dont sont victimes les personnes pauvres. L’expérience montre que ces effets s’observent par le faible recours aux soins par les personnes pauvres bénéficiant de soins gratuits totalement pris en charge par les comités de gestion des centres de santé périphériques. Il en va de même pour les soins visés par un régime d’exemption qui profitent d’emblée aux personnes ayant la volonté et les moyens de se rendre dans les structures autorisées, à la différence des personnes pauvres qui font face à d’autres obstacles liés aux dispositions personnelles, aux tabous sociaux et aux contraintes en matière d’accessibilité géographique. Il s’avère qu’un accompagnement est nécessaire pour garantir l’accès aux soins de cette catégorie de personnes vulnérables [55].

Nous retiendrons que les exemptions de paiement des soins introduisent des dispositions favorables à un meilleur accès aux soins des personnes pauvres, mais cela n’est pas possible dans les conditions décrites pour les personnes vulnérables visées par les politiques publiques. Dans la pratique, les personnes pauvres ont un droit d’accès garanti et souvent facilité aux soins gratuits, mais la jouissance de ce droit est soumise aux contraintes décrites ci-dessus. On devrait donc mettre en place des dispositions supplémentaires (facilité d’accès pour des groupes précis, particulièrement vulnérables) pour rendre plus efficaces les mesures d’exemption.

# Conclusion

## Au-delà des exemptions de paiement, la quête d’équité et d’efficacité des politiques publiques

L’attention portée sur des domaines différents d’exemptions ciblées de paiement des soins montre à la fois l’intérêt pour de telles interventions censées contribuer positivement à l’amélioration de l’accès aux soins des plus vulnérables et au renforcement du système de santé. L’expérience montre que les effets sont mitigés, et cela s’explique en partie par la mise en place souvent soudaine, sans préparation suffisante et sans documents d’orientation officiels ni une diffusion élargie de l’information auprès de toutes les catégories de la population. En tant que réponse perfectible aux besoins des groupes cibles et aux faiblesses de l’offre de soins, les exemptions de paiement constituent une occasion de renforcer le système de santé [56, 57]. En principe, des exemptions ciblées s’inscrivant dans des politiques publiques particulières constituent un catalyseur pour l’accès aux soins des groupes vulnérables. À l’inverse, les conditions défavorables de mise en place de telles initiatives révèlent les dysfonctionnements ordinaires du système de santé [58]. Par exemple, le manque de matériel médico-technique pour un centre de santé qui accueille une vingtaine de personnes par jour peut passer inaperçu. Toutefois, lorsque les effectifs sont triplés à cause d’une exemption ciblée de paiement, on comprend l’incidence que cela peut avoir sur la disponibilité et la qualité de l’offre de soins. Les contextes d’application des exemptions de paiement des soins renseignent sur la complexité des problèmes à résoudre par les politiques publiques de santé, mais aussi sur les ressources humaines, financières et matérielles, ainsi que sur les réformes nécessaires. Dans certains cas, des incohérences ou des décalages ont été observés entre les prévisions et la réalité dans la mise en oeuvre des interventions. Ainsi, les exemptions se voulant totales ne s’appliquent pas toujours en temps réel (variation d’une structure à l’autre pour le paiement de certains services). Il en va de même pour la gratuité sélective lorsqu’une partie du régime de soins est concernée (cas des médicaments) à la différence d’autres prestations payantes (cas des examens biologiques pour le VIH/sida et la tuberculose).

L’évolution des options gouvernementales au Burkina Faso (réticence quant aux exemptions totales de paiement à l’échelle nationale après les gratuités de l’État providence et l’adoption du recouvrement des coûts) montre la part d’imprévisibilité d’une politique publique de santé qui n’est pas conforme à une ligne directrice permanente. Dans le cas présent, les changements politiques récents ont vraisemblablement joué un rôle catalyseur dans l’adoption d’une série de mesures d’exemption totale de paiement attendues depuis plusieurs années. Cependant, l’État s’engage dans des proportions inégalées jusque-là qui requièrent un investissement soutenu pendant la durée des interventions concernées. Les enjeux sont à la fois d’ordre sanitaire (atteindre des objectifs de santé qui justifient l’engagement étatique et renforcer l’efficacité du système de santé par la mise en oeuvre à grande échelle), politique (mise en oeuvre réussie d’interventions s’inscrivant dans la politique nationale de santé et de développement) et social (faire profiter la population couverte, notamment les personnes pauvres, des services inclus dans les plans officiels). Comme pour d’autres grandes interventions antérieures (cas de la subvention nationale des accouchements et des soins obstétricaux et néonatals d’urgence qui prend fin logiquement avec une mesure élargie pour le même groupe cible), l’absence de données probantes dans le cadre du processus de mise en oeuvre a constitué jusqu’à présent le maillon faible du système national d’exemption de paiement des soins. Comment justifier les décisions d’allocations de ressources sans se fonder sur des données probantes issues d’évaluations validées ? Cela montre plus que jamais l’intérêt de revoir l’approche globale en matière de politiques publiques nationales de santé en ayant une vision plus prospective permettant de prendre des décisions en temps réel et d’éviter la mise en place rapide sans préparation suffisante qui est le lot commun des grandes interventions entreprises jusque-là.

#### Abréviations

ACT : Thérapie combinée à base d’artémisinine

ARV : Antirétroviraux

CAMEG : Centrale d’achat des médicaments essentiels génériques

CSPS : Centre de santé et de promotion sociale

MILDA : Moustiquaire imprégnée d’insecticide à longue durée d’action

OMD : Objectifs du Millénaire pour le développement ONG : Organisation non gouvernementale

ESSP : Établissement de soins de santé primaires

PNT : Programme national de lutte contre la tuberculose

TB : Tuberculose

#### Remerciements

Je tiens à remercier sincèrement les coordonnateurs de programme (Pr Jean-Pierre Olivier de Sardan et D^r^ Valéry Ridde), les membres des équipes de chaque pays, particulièrement ceux du Burkina Faso, le Centre de recherches pour le développement international et l’Agence française de Développement, qui ont assuré le financement, ainsi que tous les répondants pour leur contribution à l’atteinte des résultats scientifiques poursuivis.

#### Financement

La publication de cet article a été financée par le Centre de recherches pour le développement international.

#### Disponibilité des données et du matériel

Les données sont disponibles et peuvent être utilisées par l’équipe de recherche, sous réserve du respect strict des clauses éthiques concernant surtout l’anonymat et la confidentialité des répondants interrogés.

#### Contribution des auteurs

Maurice Yaogo a coordonné les activités de recherche qualitative au Burkina Faso et a rédigé l’article.

#### Intérêts conflictuels

L’auteur n’a aucun intérêt conflictuel à déclarer.

#### Consentement à la publication

Ne s’applique pas.

#### Approbation éthique et consentement à la participation

Le protocole de recherche a reçu l’approbation éthique du Comité national d’éthique en santé du Burkina Faso. Les règles éthiques prévues ont été respectées et matérialisées au moyen de la signature d’un formulaire de consentement des répondants avant toute activité de recherche.

Date de la publication : 12 juillet 2017

**Références**

1. Palmer, N. et coll., « Health financing to promote access in low income settings – how much do we know ? », *Lancet*, vol. 364, 2004, p. 1365-1370.
2. Robert, E. et V. Ridde, *Les paiements directs des soins dans les pays à faible revenu ne font plus l’unanimité au sein de la communauté internationale : une analyse documentaire*, Montréal, Les Cahiers du CIRDIS – Collection Recherche, 2012.
3. Banque mondiale, *Burkina Faso : vue d’ensemble*. Accès : [http://www.banquemondiale.org/fr/country/burkinafaso/overview](http://www.worldbank.org/en/country/). Consulté le 26 juillet 2016.
4. Ridde, V. et A. Bicaba, *Revue des politiques d’exemption-subvention du paiement au Burkina Faso : la stratégie de subvention des soins obstétricaux et néonatals d’urgence*, rapport de recherche, Ouagadougou, 2009.
5. Gali Gali, I. et M. Yaogo, *Revue documentaire sur les politiques d’exemption des soins de santé maternelle au Burkina Faso*, rapport de recherche, Bobo-Dioulasso, 2012.
6. Ridde, V. et J.-E. Girard, « Douze ans après l’Initiative de Bamako : constats et implications politiques pour l’équité d’accès aux services de santé des indigents africains », *Santé publique*, vol. 16, 2004, p. 37-51.
7. Ridde, V. et J.P.O. de Sardan, *Les politiques de suppression et de subvention du paiement des soins au Burkina Faso, Mali et Niger*, Montréal, CRCHUM/LASDEL, 2012.
8. Olivier de Sardan, J.P. et V. Ridde, *Une politique publique de santé et ses contradictions : la gratuité des soins au Burkina Faso, au Mali et au Niger*, Paris, Karthala, 2014.
9. Yaogo, M., A. Kagambega et D. Dabiré, « Émergence et formulation des exemptions du paiement des services liés au paludisme au Burkina Faso », *in* de Sardan, J.P.O. et V. Ridde (éd.), *Une politique publique de santé et ses contradictions : la gratuité des soins au Burkina Faso, au Mali et au Niger*, Paris, Karthala, 2014, p. 87-99.
10. Konaté, B., R. Zerbo et M. Yaogo, « Mise en oeuvre de la gratuité des traitements contre la tuberculose et le sida au Burkina Faso », *in* de Sardan, J.P.O. et V. Ridde (éd.), *Une politique publique de santé et ses contradictions : la gratuité des soins au Burkina Faso, au Mali et au Niger*, Paris, Karthala, 2014, p. 199-210.
11. Olivier de Sardan, J.P., « La quantité sans la qualité ? Mises en forme et mises en oeuvre des politiques d’exemptions de paiement au Sahel », *in* de Sardan, J.P.O. et V. Ridde (éd.), *Une politique publique de santé et ses contradictions : la gratuité des soins au Burkina Faso, au Mali et au Niger*, Paris, Karthala, 2014, p. 51-84.
12. Van Lerberghe, W. et V. De Brouwere, « État de santé et santé de l’État en Afrique subsaharienne », *Afrique contemporaine*, vol. 195, 2000, p. 175-190.
13. Yaogo, M., R. Zerbo et V. Ridde, *L’histoire de la gratuité des soins de santé au Burkina Faso*, rapport de recherche, Bobo-Dioulasso, 2012.
14. Olivier de Sardan, J.P. et V. Ridde, « L’exemption de paiement des soins au Burkina Faso, au Mali et Niger : les contradictions des politiques publiques », *Afrique contemporaine*, 2012, p. 243-246.
15. Olivier de Sardan, J.P. et V. Ridde, « Les spécificités des politiques publiques et des systèmes de santé en Afrique sahélienne », *in* de Sardan, J.P.O. et V. Ridde (éd.), *Une politique publique de santé et ses contradictions : la gratuité des soins au Burkina Faso, au Mali et au Niger*, Paris, Karthala, 2014, p. 15-30.
16. Yaogo, M. et S. Yaya, « Problématique de l’accès aux soins et de l’équité en santé : émergence et enjeux autour de la mise en pratique », Yaya, S. (éd.), *Les maux et les choses de la santé : acteurs, pratiques et systèmes de santé dans le tiers-monde*, Québec, Presses de l’Université Laval, 2010, p. 123-146.
17. Olivier de Sardan, J.P., *La rigueur du qualitatif : les contraintes empiriques de l’interprétation socio-anthropologique*, Louvain-La-Neuve, Academia-Bruylant, 2008.
18. Van Der Veken, K., F. Richard, B. Marchal, S. Witter, J.P. Dossou, A. Essolbi et coll., *POEM (Policy Effects Mapping): a framework to assess the effects of a targeted policy on a local health system*, rapport scientifique, Antwerp, 2014.
19. Organisation mondiale de la Santé, *Les soins de santé primaire*, Genève, Organisation mondiale de la Santé, 1978.
20. Marchal, B., S. Belle, V. De Brouwere et S. Witter, « Studying complex interventions: reflections from the FEMHealth project on evaluating fee exemption policies in West Africa and Morocco », *BMC Health Services Research*, vol. 13, 2013, p. 469.
21. Ministère de la Santé publique, *Document de programmation sanitaire nationale 1980-1990 (Avant-projet)*, rapport, Ouagadougou, 1978.
22. Ministère de la Santé, *Document de politique sanitaire nationale*, rapport, Ouagadougou, 2000.
23. Ministère de la Santé, *Politique nationale de santé*, rapport, Ouagadougou, 2011.
24. Ministère de la Santé, *Stratégie nationale subvention des accouchements et des soins obstétricaux et néonatals d’urgence au Burkina Faso*, rapport, Ouagadougou, 2006.
25. Kafando, T.W., « Les perspectives 1960-1975 », *Notes et documents voltaïques*, vol. 7, n^o^ 2, 1974, p. 3-27.
26. Hervouët, J.P., « Aménagement hydro-agricole et onchocercose (Loumana, Haute-Volta) », *in* *De l’épidémiologie à la géographie humaine*, Paris, ACCT, CNRS, 1983. p. 271-274.
27. Monné, R., *Secteurs sanitaires confessionnels et publics dans la ville de Ouagadougou (Burkina Faso). Quelle articulation* ?, rapport de recherche, Ouagadougou, 1996.
28. Dozon, J.P., « Ce que valoriser la médecine traditionnelle veut dire », *Politique africaine*, vol. 28, 1987, p. 9-20.
29. Jaffré, Y. et J.P.O. de Sardan, *Une médecine inhospitalière : les difficiles relations entre soignants et soignés dans cinq capitales d’Afrique de l’Ouest*, Paris, Karthala, 2003.
30. Pottier, R., « Réhabilitation de la médecine traditionnelle et des soins de santé primaires : deux idéologies d’encadrement des masses rurales », *in* Retel-Laurentin, A. (éd.), *Une anthropologie médicale en France, Exposés et débats de table ronde*, “Santé et sciences humaines”, Paris, CNRS (éd.), 1983, p. 107-115.
31. Hours, B., « La santé publique entre soins de santé primaires et management », *Cahiers de sciences humaines*, vol. 28, 1992, p. 132.
32. Soubeiga, A., *Les stratégies des soins de santé primaires au Burkina : mobilisation communautaire et logiques paysannes*, thèse de doctorat, Bordeaux, 1992.
33. Foulon, G. et R.T. Somé, « Quel système de financement de l’accès aux soins dans les pays en développement : le cas des districts de santé au Burkina Faso », *Mondes en développement*, 2005, p. 131-154.
34. Ridde, V. et F. Morestin, « A scoping review of the literature on the abolition of user fees in healthcare services in Africa », *Health Policy and Planning*, vol. 26, 2011, p. 1-11.
35. Olivier de Sardan, J.P., « L’exemption de paiement des soins au Burkina Faso, au Mali et au Niger : les contradictions des politiques publiques », *Afrique contemporaine*, vol. 243, 2012, p. 11-32.
36. Hours, B., *Systèmes et politiques de santé : de la santé publique à l’anthropologie*, Paris, Karthala, 2001.
37. Bado, J.P., *Médecine coloniale et grandes endémies en Afrique*, Paris, Karthala, 1996.
38. Massa, G. et G. Madiega, *La Haute-Volta coloniale, témoignages, recherches, regards*, Paris, Karthala, 1995.
39. Becker, C. et R. Collignon, « Épidémies et médecines coloniales en Afrique de l’Ouest », *Cahiers Santé*, vol. 8, n^o^ 6, 1998, p. 411-416.
40. Hervouët, J.P., « La mise en valeur des vallées des Volta Blanche et Rouge : un accident historique », *Cahiers ORSTOM/Série Sciences humaines*, vol. 11, n^o^ 1, 1978, p. 81-97.
41. Yaogo, M., T. Some, B. Konaté et coll., *Évaluation multi-pays de l’impact du passage à l’échelle de la lutte contre le VIH/sida, la tuberculose et le paludisme avec référence spéciale au Fonds Mondial : Enquête auprès des organisations de la Société civile*, rapport de recherche, Bobo-Dioulasso, 2009.
42. M’Bokolo, E., « Histoire des maladies – Histoire et maladies : l’Afrique », *in* Augé, M., C. Herzlich (éd.), *Le sens du mal : anthropologie, histoire, sociologie de la maladie*, Paris, Édition des Archives contemporaines, 1984, p. 155-186.
43. Bernède, M., « Afrique en attendant les ARV pédiatriques », *Transversal*, vol. 21, 2004, p. 14-18.
44. Amnistie Internationale. Accès : [https://www.amnesty.org/en/documents/afr60/001/2009/fr/](https://www.amnesty.org/en/documents/afr60/001/). Consulté le 13 août 2016.
45. Nikiema, N. et J. Kinda, *Dictionnaire orthographique du mooré*, dictionnaire, Ouagadougou, 1997.
46. de Sardan, J.P.O., *Anthropologie et développement : essai en socio-anthropologie du changement social*, Paris, APAD-Karthala, 1995.
47. Yaogo, M., *La mobilisation physique et financière dans le cadre du développement local : exemples pris dans les provinces du Bazega, du Boulgou et du Zoundweogo*, rapport de recherche, Ouagadougou, 2004.
48. Ridde, V., M. Yaogo, Y. Kafando, K. Kadio, M. Ouedraogo, A. Bicaba et coll., « Targeting the worst-off for free health care: a process evaluation in Burkina Faso », *Evaluation and Program Planning*, vol. 34, 2011, p. 333-342.
49. Richard, F., S. Witter et V. De Brouwere, *Réduire les barrières financières aux soins obstétricaux dans les pays à faibles ressources*, Studies in Health Services Organisation and Policy, Antwerp, ITGPress, 2008.
50. Jaffré, B., « Prolégomènes à une réforme des services de santé : de l’identification des dysfonctionnements à la redéfinition d’outils efficaces », *Médecine tropicale*, vol. 64/6, 2004, p. 527-532.
51. Meunier, A., « L’État et l’offre de soins au Burkina Faso », *Populations et Sociétés*, vol. 18, n^o^ 1, 2000, p. 33-41.
52. Ridde, V., P. Pluye et L. Queuille, « Évaluer la pérennité des programmes de santé publique : un outil et son application en Haïti », *Revue d’épidémiologie et de santé publique*, vol. 54, 2006, p. 421-431.
53. Borghi, J., T.S. Katerini et V. Filippi, « Les coûts des soins obstétricaux et leurs conséquences sociales et économiques pour les ménages », *Studies in Health Services Organization and Policies*, vol. 25, 2008, p. 27-52.
54. de Sardan, J.P.O., « Addiction à l’aide, sevrage ou impuissance ? Les ONG médicales face aux systèmes de santé », *in* de Sardan, J.P.O. et V. Ridde (éd.), *Une politique publique de santé et ses contradictions : la gratuité des soins au Burkina Faso, au Mali et au Niger*, Paris, Karthala, 2014, p. 239-302.
55. Yaogo, M., V. Ridde, Y. Kafando et K. Kadio, « Enjeux disciplinaires, éthiques et politiques d’une recherche-action sur l’accès aux soins de santé des indigents au Burkina Faso », *in* Mondain, N. et E. Bologo (éd.), *La recherche en contexte de vulnérabilité : engagement du chercheur et enjeux éthiques*, Paris, Harmattan, 2012, p. 65-85.
56. Ridde, V., B. Meessen et S. Kouanda, « L’abolition sélective du paiement direct des soins en Afrique subsaharienne : une opportunité pour le renforcement des systèmes de santé ? », *Santé publique*, vol. 23, n^o^ 1, 2011, p. 61-67.
57. Ridde, V. et L. Queuille, *L’exemption du paiement, un pas vers l’accès universel aux soins de santé : expériences pilotes au Burkina Faso*, rapport de recherche, Montréal-Ouagadougou, 2010.
58. Touré, L., « La gratuité comme révélateur des dysfonctionnements ordinaires du système de santé au Mali », *in* de Sardan, J.P.O. et V. Ridde (éd.), *Une politique publique de santé et ses contradictions : la gratuité des soins au Burkina Faso, au Mali et au Niger*, Paris, Karthala, 2014, p. 183-198.
