# Supplementary material for: People and research: improved health systems for West Africans, by West Africans - report on special supplement
Source: BMC Proc. 2019 Feb 7;13(Suppl 1):1. doi: 10.1186/s12919-019-0162-0 (PMC6366023; doi:10.1186/s12919-019-0162-0)
Supplement: Supplementary file 5 — État de la santé mobile: étude qualitative sur les attentes relatives à la santé mobile dans le district sanitaire rural de Nouna, au Burkina Faso, Duclos, V., Yé, M., Moubassira, K., Sanou, H., Sawadogo, H., Bibeau, G., Sié, A. [file 12919_2019_162_MOESM5_ESM.docx]

***État de la santé mobile : étude qualitative sur les attentes relatives à la santé mobile dans le district sanitaire rural de Nouna, au Burkina Faso***

**Vincent Duclos^1§^, Maurice Yé^2^, Kagoné Moubassira^2^, Hamidou Sanou^2^, Hélène Sawadogo^2^, Gilles Bibeau^3^, Ali Sié^2^**

^1^ Département d’études sociales de la médecine de l’Université McGill, 3647, rue Peel, Montréal, Québec, Canada, H3A 1X1

^2^ Centre de Recherche en Santé de Nouna, boîte postale 02, Nouna, Burkina Faso

^3^ Département d’anthropologie, Université de Montréal, Pavillon Lionel-Groulx, 3150, rue Jean-Brillant, Montréal, Québec, Canada, H3T 1N8

§Auteur-ressource

Adresses électroniques :

VD : [vincent.duclos@mcgill.ca](mailto:vincent.duclos@mcgill.ca)

MY : [yemaure@yahoo.fr](mailto:yemaure@yahoo.fr)

KM : [kmoubache@yahoo.fr](mailto:kmoubache@yahoo.fr)

HS : [hsanou@ymail.com](mailto:hsanou@ymail.com)

GB : [gilles.bibeau2@sympatico.ca](mailto:gilles.bibeau2@sympatico.ca)

AS : [alisie.crsn@fasonet.bf](mailto:alisie.crsn@fasonet.bf)

**Résumé**

**Contexte :** Les projets de santé mobile réalisés dans des pays à faible revenu et à revenu intermédiaire (PFR-PRI) suscitent des attentes élevées et bien connues chez les organismes, les décideurs et les chercheurs qui oeuvrent au développement. En revanche, on se penche rarement sur les attentes des utilisateurs directs et indirects des services de santé mobile. En préparation à une intervention proposée dans le district sanitaire de Nouna, au Burkina Faso rural, cette étude visait à déterminer les retombées, les défis et les limites associés à la santé mobile. On a abordé les attentes comme étant une forme de connaissance contextuelle qui est indissociable des conditions, des pratiques et des expériences locales.

**Méthodologies :** L’étude a été réalisée dans le district sanitaire de Nouna (DSN). Nous avons utilisé une approche qualitative et avons réalisé des entrevues individuelles semi-structurées et des entrevues collectives (n=10). Les participants comprenaient des travailleurs de la santé (n=19), des marraines (n=24), des femmes enceintes (n=19), des mères d’enfants âgés de 12 à 24 mois (n=33) et des femmes en âge de procréer (n=92). On a réalisé des analyses qualitatives de thèmes et de contenu.

**Résultats :** Les participants s’attendent à ce que les services de santé mobile aident à récupérer les patients dont le suivi a été interrompu, à améliorer le suivi des soins gynécologiques et à renforcer les relations entre les femmes enceintes et les centres de soins de santé primaires. Les retombées attendues ne peuvent pas se réduire à une réalisation technologique (envoi de messages); elles consistent plutôt en un réseau de soutien plus vaste. La mise en oeuvre de la santé mobile devrait présenter des défis considérables, dont des obstacles technologiques, des difficultés organisationnelles, des problèmes liés à la sexospécificité, des préoccupations sur le plan de la confidentialité et des répercussions imprévues. On s’attend également à ce que la santé mobile comporte des limites intrinsèques, notamment en ce qui a trait à l’accès des femmes enceintes aux soins gynécologiques, un problème sur lequel la santé mobile ne devrait pas avoir d’incidence notable.

**Conclusions :** Les attentes relatives à la santé mobile semblent être des connaissances contextuelles indissociables des expériences, des pratiques et des contraintes locales en matière de santé. Cela complique l’utilisation d’approches universelles des connaissances en matière de santé mobile, tout en laissant entrevoir des avantages concrets et prévisibles. Les conclusions de cette étude aideront à orienter la conception et la mise en oeuvre des projets de santé mobile, ce qui maximisera leurs chances de réussite.

**Mots clés**: santé mobile, attentes, Burkina Faso, connaissance contextuelle, santé maternelle, santé mondiale

**Contexte**

Sous l’impulsion d’organismes gouvernementaux, d’institutions internationales, d’organisations non gouvernementales (ONG) et d’acteurs du secteur privé, la santé mobile gagne rapidement en importance dans le programme de santé mondiale. [1-5] La santé mobile, qui fait partie de la catégorie plus générale de la télématique de santé (ou télésanté), peut être définie comme des pratiques médicales et de santé publique fondées sur l’utilisation d’appareils mobiles, comme des téléphones, des dispositifs de surveillance des patients et d’autres appareils sans fil. [1] L’expansion de la santé mobile est particulièrement rapide en Afrique subsaharienne, en raison de l’amélioration de l’accès aux téléphones mobiles. [6, 7] À la mi-2015, on comptait 386 millions d’abonnements individuels à des services de téléphonie cellulaire en Afrique subsaharienne. En 2020, un peu moins de la moitié (49 %) de la population sera abonnée à un service de téléphonie cellulaire. [8] Dans ce contexte, les projets de santé mobile en Afrique subsaharienne ont pris toutes sortes de formes. [9] Les applications les plus courantes comprennent l’utilisation de téléphones cellulaires pour améliorer le suivi des patients et de la prise de médicaments [10, 11], le soutien des travailleurs de la santé dans les régions éloignées [12-14] et la collecte de données pour la santé publique et la surveillance des maladies. [15]

Les interventions en santé mobile en Afrique subsaharienne et dans les PFR-PRI en général suscitent des attentes élevées chez les organismes, les décideurs et les chercheurs qui oeuvrent au développement. [16-18] Les avantages les plus souvent associés à la santé mobile comprennent l’amélioration de l’accès aux soins de santé et à l’information sur la santé, l’amélioration du rapport coût-efficacité de la prestation des services, l’amélioration des capacités de diagnostic, de traitement et de surveillance des maladies et l’accès opportun à l’information sur la santé publique. [19] Jusqu’ici, la santé maternelle a été l’un des principaux objets des interventions en santé mobile en Afrique subsaharienne et dans les PFR-PRI en général. [20] On a notamment réalisé des projets de santé mobile dans le but d’améliorer le suivi [21] durant la grossesse et le post-partum [22], ou encore d’accroître la présence de personnes compétentes lors des accouchements. [23] De manière générale, on considère que la santé mobile devrait faciliter la réalisation des objectifs du Millénaire pour le développement (OMD) en matière de santé des enfants et des mères. [24-26] On suppose par exemple que les technologies de communication mobile pourraient réduire les inégalités en matière de santé maternelle qui sont liées au coût, à la distance et à une infrastructure de santé inadéquate. [27]

Malgré ces attentes, on déplore souvent dans la littérature savante l’écart entre les avantages attendus et les avantages réels, ainsi que le manque de données probantes en ce qui concerne les répercussions réelles des projets de santé mobile. [3, 6, 28] Les données probantes sont éparses, incomplètes et peu convaincantes quand vient le temps d’évaluer l’incidence économique et durable de la technologie mobile sur les indicateurs de santé. Parmi les principales inquiétudes, on note le manque de données probantes indiquant une mise à l’échelle réussie, car la majorité des projets pilotes de santé mobile réalisés dans les PFR-PRI n’ont pas pu être intégrés au système de santé. [29, 30] Cela a amené les organismes oeuvrant à la recherche et à la réalisation des projets à exiger des données probantes pour la prise de décisions éclairées en matière d’investissements dans le secteur. [31, 32] Cette situation s’applique également aux attentes relatives à la santé mobile et à ses répercussions concrètes sur la santé maternelle en Afrique subsaharienne. [20]

Jusqu’ici, la production de connaissances universelles et fondées sur des données probantes en matière de santé mobile n’a tenu compte des attentes locales des utilisateurs directs et indirects que dans une faible mesure. Dans bien des cas, les expériences et les points de vue locaux tendent à se réduire aux questions d’acceptation par les utilisateurs et de faisabilité, et s’inscrivent dans des préoccupations plus générales concernant l’adoption des interventions. [12, 33, 34] Cela pose problème à de nombreux égards. D’abord, la relation entre l’acceptation de la technologie et la réalisation réussie des projets de santé mobile n’est pas évidente. [35] Les réactions favorables à la santé mobile ne se traduisent pas forcément par un fort taux d’adoption, car la réceptivité est indissociable de différentes conditions comme les facteurs socioéconomiques, les obstacles géographiques et la qualité des soins. [6, 23] Qui plus est, en réduisant les attentes locales en matière de santé mobile à des questions d’acceptation et de faisabilité, on néglige la complexité inhérente aux comportements favorables à la santé [36-38] et aux processus d’adoption de la technologie. [39-41] Cela frise parfois le déterminisme technologique, le contexte local étant considéré comme une page blanche sur laquelle la santé mobile pourra être inscrite sans grande difficulté. [42] Par conséquent, la recherche en santé mobile risque d’aboutir à des connaissances qui n’ont, en bonne partie, rien à voir avec la manière dont les communautés locales déterminent, expliquent et gèrent les problèmes de santé. [43] Cette divergence peut à son tour miner l’influence des connaissances locales sur les politiques et la conception des projets de santé mobile. Le présent article peut être considéré comme un effort visant à combler cet écart à partir de la base, en se concentrant sur les attentes relatives à la santé mobile comme *connaissance contextuelle*, indissociable des expériences, des pratiques et des conditions de vie précises.

**MOS@N : Santé mobile à Nouna**

L’article suivant porte sur l’incidence que la santé mobile devrait avoir sur les soins gynécologiques dans le district sanitaire de Nouna (DSN) du Burkina Faso. Les données présentées ici ont été recueillies dans le cadre d’un grand projet de recherche sur la santé mobile MOS@N en cours de réalisation dans le DSN. Le projet MOS@N (santé mobile Nouna), qui est soutenu par le Centre de recherches pour le développement international (CRDI, Canada), est réalisé par le Centre de Recherche en Santé de Nouna (CRSN), un centre national de recherche situé dans le Burkina Faso rural. [44, 45] Ce grand projet consiste à étudier l’incidence des technologies mobiles sur l’utilisation des services de santé par les femmes enceintes et les personnes atteintes du VIH/sida. Dans cet article, nous nous concentrerons sur la santé maternelle.

Malgré d’importantes améliorations [46, 47], le taux de mortalité maternelle élevé (341 morts maternelles sur 100 000 naissances vivantes) demeure un problème de santé publique majeur au Burkina Faso. Cela est particulièrement vrai dans les zones rurales, où vit la majorité des 17 millions d’habitants du pays. Selon les estimations réalisées par le ministère de la Santé, la proportion des femmes qui ont eu au moins deux consultations de soins prénataux (SP) en 2013 était de 72 % pour l’ensemble du Burkina Faso, et de 70 % pour le DSN. [48] On estime que la proportion de femmes ayant accouché dans un établissement de santé du Burkina Faso rural en 2010 était de 61,5 %. [48] Dans les régions rurales, les obstacles à l’accès aux soins gynécologiques demeurent nombreux. Ils comprennent des obstacles sociaux, géographiques et économiques, des pénuries de professionnels de la santé, un manque d’information sur la santé sexuelle et reproductive, le coût des traitements médicaux et la perception négative de la qualité des soins. [49-53]

Dans de telles circonstances, MOS@N vise à examiner l’incidence de la santé mobile sur les comportements favorables à la santé chez les femmes enceintes et sur le suivi de la grossesse dans le DSN. L’objectif général de MOS@N est de comprendre comment la technologie de santé mobile peut mener à des systèmes de santé plus équitables, tout en examinant les principaux défis, les limites et les effets imprévus. Le projet consiste, entre autres, à envoyer des rappels de rendez-vous médicaux et des conseils médicaux aux marraines, qui sont les agents communautaires responsables du suivi des femmes enceintes de leur village. À cette fin, on équipe les marraines d’un téléphone cellulaire (pour recevoir des messages) et d’un vélo (pour se déplacer et transmettre les messages). MOS@N, auquel participent des centres de santé et de promotion sociale (CSPS), des travailleurs de la santé, des techniciens en technologies de l’information et de la communication, des marraines et des chercheurs en santé publique, est en cours de réalisation dans 26 villages rattachés à cinq CSPS (auxquels s’ajoutent cinq autres CSPS à titre de groupe témoin).

Dans le présent article, nous présentons les données qualitatives recueillies durant la première phase du projet MOS@N, c’est-à-dire au début de sa mise en oeuvre. Le but de la première phase consistait à déterminer les attentes des femmes et des travailleurs de la santé locaux en matière de santé mobile. La recherche présentée dans le présent document tente de répondre aux questions suivantes :

- Quelle incidence les femmes et les travailleurs de la santé de Nouna s’attendent-ils à ce que la santé mobile ait sur l’accès aux soins de santé ?
- Quels sont les défis et les limites prévus en ce qui a trait à la santé mobile ?
- Quel est le lien entre ces attentes (avantages, défis et limites) et les pratiques, les expériences et les contraintes locales en matière de santé ?

**Méthodologies**

**Conception de la recherche**

L’étude a été menée dans le DSN, au Burkina Faso. Le DSN se trouve à environ 300 kilomètres au nord-ouest de Ouagadougou, la capitale du Burkina Faso. Il s’agit d’un des six districts de la région sanitaire de la Boucle du Mouhoun. Le DSN englobe la région géographique de la province de Kossi, dans l’ouest du pays. Le DSN comprend la ville de Nouna, qui compte 29 297 habitants, et une région rurale où vivent environ 331 020 personnes. L’infrastructure de santé du DSN consiste en un hôpital de district (HD), à Nouna, et 43 centres de santé et de promotion sociale, dont dix participent au projet MOS@N.

MOS@N est un projet de 36 mois qui comprend un volet qualitatif et un volet quantitatif. Dans cet article, nous nous concentrons sur le volet qualitatif, et plus particulièrement sur les données recueillies au début du projet. En nous concentrant sur la première phase de collecte de données, nous cherchons à examiner les attentes relatives à la santé mobile avant la mise en oeuvre complète du projet MOS@N. Les données ont été recueillies dans le cadre d’entrevues individuelles semi-structurées et d’entrevues collectives. La recherche a été conçue et réalisée par une équipe de chercheurs du CRSN, de l’Université McGill et de l’Université de Montréal. Le comité d’éthique du ministère de la Santé du Burkina Faso et le conseil éthique de recherche institutionnel du CRSN ont approuvé l’éthique de l’étude.

**Échantillon et recrutement**

Les données qualitatives présentées dans cet article ont été recueillies en mai et en juin 2014. On a employé des méthodes d’échantillonnage raisonné mixtes pour sélectionner les participants. [54] Comme c’est souvent le cas en recherche qualitative, le but n’était pas d’obtenir un échantillon représentatif des différentes catégories de participants, mais plutôt de recueillir une importante quantité d’information auprès d’eux. [55] Les participants peuvent être divisés en cinq groupes, soit 1) les travailleurs de santé des CSPS du DSN, 2) les marraines participant au projet MOS@N, 3) les femmes enceintes, 4) les mères d’enfants âgés de 12 à 24 mois et 5) les femmes en âge de procréer. Le tableau 1 présente la répartition des participants.

Selon une méthode d’échantillonnage raisonnée experte, chaque travailleur de la santé (n=19) des CSPS participants (n=10) a été interrogé. Les travailleurs de la santé appartiennent à deux sous-groupes, soit les infirmiers-chefs (« infirmiers-chefs de poste » ou ICP) [n=8] et les sages-femmes (n=11). Les ICP supervisent les activités médicales courantes des CSPS. Tous les ICP qui travaillaient dans les CSPS participants étaient des hommes. Les sages-femmes supervisent les services de santé maternelle offerts par les CSPS. Toutes les sages-femmes qui travaillaient dans les CSPS participants étaient des femmes.

On a également recueilli des données au moyen d’entrevues individuelles et collectives réalisées auprès de femmes issues de la population locale du DSN. On a d’abord réalisé des entrevues semi-structurées avec toutes les marraines récemment sélectionnées pour participer au projet MOS@N. Au moment des entrevues, 48 marraines vivant dans 26 villages venaient d’être sélectionnées. Selon une méthode d’échantillonnage raisonné, on a interrogé la moitié (n=24) d’entre elles durant la première phase de collecte de données qualitatives. Les entrevues avec les marraines ne portaient pas sur leurs expériences réelles relatives au projet MOS@N, qui venait tout juste de commencer, mais plutôt sur leur point de vue et leurs attentes générales. On a ensuite réalisé des entrevues semi-structurées avec des femmes enceintes (n=20) participant au projet MOS@N. On les a sélectionnées selon une méthode d’échantillonnage raisonné à quota non-proportionnel, soit au moins une participante par CSPS participant, et une moyenne de deux participantes par CSPS. On a ensuite réalisé des entrevues semi-structurées avec des mères d’enfants âgés de 12 à 24 mois (n=33). On les a sélectionnées selon une méthode d’échantillonnage raisonné à quota non proportionnel, dont le seul critère d’inclusion était d’être cliente d’un des dix CSPS participants. Au moins deux clientes de chaque CSPS ont été interrogées. Enfin, des femmes en âge de procréer (n=92) ont été recrutées pour participer à des entrevues collectives (n=10). On les a sélectionnées selon une méthode d’échantillonnage raisonné à quota non proportionnel, dont le principal critère d’inclusion était géographique, puisque l’on a réalisé une entrevue collective (moyenne de neuf participantes par CSPS) dans chacun des CSPS participants.

**Collecte des données**

Nous avons réalisé des entrevues semi-structurées auprès de travailleurs de la santé, de marraines, de femmes enceintes et de mères d’enfants âgés de 12 à 24 mois. Les entrevues ont duré 30 minutes en moyenne. On a abordé les femmes enceintes et les mères d’enfants âgés de 12 à 24 mois qui se présentaient aux CSPS pour recevoir des soins prénataux ou une consultation avec un ou plusieurs enfants. Les travailleurs de la santé et les marraines ont été abordés dans le cadre de leur participation plus générale au projet MOS@N, à leur CSPS local. Les entrevues ont été réalisées par des chercheurs formés du CRSN. Les entrevues avec les travailleurs de la santé ont été réalisées en français, et celles avec les marraines, les femmes enceintes et les mères d’enfants âgés de 12 à 24 mois ont été réalisées en dioula. Elles ont été enregistrées sur support numérique et transcrites. Les entrevues en dioula ont été transcrites en français. Elles ont été réalisées selon un guide d’entrevue préétabli portant sur différents aspects de la santé mobile, l’accès aux soins gynécologiques et les téléphones cellulaires en général. Comme c’est souvent le cas avec les entrevues qualitatives semi-structurées, le but principal consistait à poser des questions ouvertes qui peuvent donner lieu à des réponses inattendues et qui se prêtent particulièrement aux échanges sur des sujets délicats relatifs à la santé. [56] On a préféré les entrevues semi-structurées aux entrevues non structurées, car elles permettent à l’intervieweur de dévier du guide d’entrevue et d’aborder plus facilement des questions précises lorsque l’objet de la recherche est déjà suffisamment clair. [57] Elles offrent aussi plus d’uniformité si plusieurs chercheurs s’occupent de la collecte de données, comme c’était le cas ici. Les entrevues qualitatives visent à recueillir une description de la vie de la personne interrogée, tout en laissant de la place aux ambiguïtés et aux changements. [58] Se reporter aux encadrés 1 et 2 pour obtenir une illustration de nos guides d’entrevue semi-dirigée.

Les entrevues collectives, qui ont été réalisées avec des femmes en âge de procréer, ont duré une heure à une heure et demie. Elles ont aussi été effectuées en dioula, enregistrées sur support numérique et transcrites en français. Les entrevues collectives sont particulièrement utiles dans le cadre d’une telle conception comportant plusieurs méthodes, afin de clarifier, d’extrapoler, de qualifier et de contester les données recueillies dans le cadre d’autres entrevues semi-dirigées. [59] De huit à dix personnes ont participé à chaque entrevue collective. Les entrevues ont été menées par des chercheurs formés du CRSN, dont le rôle consistait à diriger la discussion et à encourager la participation de tous les membres. [60]

**Analyse des données**

L’analyse des données suivait des lignes directrices couramment utilisées pour l’analyse de données qualitatives. La première étape de l’analyse consistait à organiser et à indexer les données. Les entrevues enregistrées ont été transcrites en français et ont été lues et relues par trois des auteurs, qui ont également noté leurs idées initiales à cette étape. Nous avons ensuite utilisé des méthodes d’analyse de contenu et de thèmes. [61] Dans un premier temps, deux des auteurs ont effectué une analyse du contenu en procédant au codage in vivo/émergent et ouvert des données pertinentes. Le logiciel de données qualitatives ATLAS.ti a été utilisé pour le codage. Ce logiciel a permis aux chercheurs de créer des catégories, de regrouper les codes sous des rubriques plus générales et de formuler une description générale du sujet de la recherche. [62] Un autre auteur a ensuite organisé les données en catégories thématiques, en commençant par déterminer les thèmes, puis en les passant en revue, en les définissant et en les nommant. [63] On a retenu des exemples convaincants, puis on les a analysés et examinés de nouveau du point de vue des questions de recherche et de la littérature. Les auteurs ont ensuite comparé l’analyse thématique et l’analyse du contenu, en passant à un codage plus précis, en mettant l’accent sur les concepts associés aux attentes relatives à la santé mobile. Comme c’est souvent le cas avec l’analyse qualitative, le but n’était pas la représentativité, mais plutôt l’identification de tendances et de variations significatives. [64]

**Résultats**

Nos conclusions principales sont présentées en quatre parties. La première partie présente les perceptions générales et les utilisations courantes des téléphones cellulaires dans le DSN, tout en nous concentrant sur les utilisations liées à la santé. La deuxième partie porte sur les attentes relatives à la santé mobile et au projet MOS@N, tant au sein de la population que chez les travailleurs de la santé. La troisième partie traite des obstacles auxquels les participants s’attendent à ce que la réalisation du projet MOS@N et la santé mobile en général soit confrontée. La quatrième partie présente les limites inhérentes à la santé mobile, en examinant les obstacles à l’accès aux soins gynécologiques que les téléphones cellulaires ne devraient pas permettre de surmonter.

1. **Téléphones cellulaires à Nouna**

Le niveau d’expérience de la population de Nouna dans l’utilisation des téléphones cellulaires est variable. Les participants savent tous ce qu’est un téléphone cellulaire, puisque l’on en voit dans les villages depuis quelques années, et ont, de manière générale, une opinion très positive de ceux-ci. Les participants s’en servent surtout pour rester en contact avec leurs proches qui vivent dans d’autres villages ou régions du pays. Ils s’en servent également pour écouter de la musique.

Les femmes du DSN utilisent aussi les téléphones cellulaires pour des questions de santé. La communication avec des parents à propos d’une maladie reste l’utilisation relative à la santé la plus souvent mentionnée. Les femmes munies d’un téléphone cellulaire qui se rendent seules à un CSPS appellent parfois à la maison pour informer leur famille de leur état ou de l’état d’un enfant. Elles se servent également de leur téléphone pour demander à leur mari de les amener à un CSPS lorsqu’elles ne sentent pas bien au cours de leur grossesse. D’autres femmes préfèrent appeler leur CSPS pour savoir si un travailleur de la santé se trouve sur place avant de se déplacer. Dans l’ensemble, les femmes considèrent le téléphone cellulaire comme un outil leur permettant de gagner du temps et réduisant la nécessité de voyager dans des conditions difficiles.

*« Le téléphone cellulaire est une bonne chose, parce qu’il réduit la distance et nous évite les bris de vélo, car nous pouvons rester où nous sommes et recevoir l’information dont nous avons besoin. »* (Biron Marka, femme en âge de procréer)

De nombreuses femmes du DSN n’ont toutefois pas facilement accès à un téléphone cellulaire. La plupart des participantes ont mentionné avoir peu d’expérience dans l’utilisation des téléphones cellulaires, et la majorité n’en a jamais possédé un. Seul un tiers (n=16) des marraines sélectionnées pour participer au projet MOS@N (n=48) avait déjà possédé un téléphone cellulaire, et la moitié d’entre elles en avait possédé un pendant moins d’un an. Dans de nombreux cas, leur mari ou d’autres membres de leur famille possèdent un téléphone, mais les femmes peuvent rarement s’en servir. Les femmes qui ont un téléphone cellulaire peuvent aussi avoir des difficultés à l’utiliser. Les téléphones cellulaires coûtent parfois cher, ou les maris leur interdisent parfois de s’en servir pour appeler. Voici ce que deux femmes avaient à dire à ce sujet :

*« J’ai acheté un téléphone cellulaire l’année dernière. J’y avais mis une carte SIM, mais mon mari l’a retirée et la garde avec lui parce qu’il ne me fait pas confiance. Je me sers donc du téléphone pour écouter de la musique. »* (Sobon, femme en âge de procréer)

*« Nous pouvons nous en servir pour appeler, mais nos maris ne tolèrent pas que nous y mettions une carte SIM. Nous ne pouvons donc nous en servir que pour écouter de la musique. »*  (Sobon, femme en âge de procréer)

Bon nombre des femmes interrogées ont parlé de ces obstacles quand on a discuté de la présence des téléphones cellulaires dans le DSN.

À l’inverse, tous les travailleurs de la santé participant à l’étude possédaient déjà un téléphone cellulaire depuis un certain temps. Les travailleurs de la santé avaient une expérience considérable de l’utilisation des téléphones cellulaires à des fins personnelles et professionnelles. Tous les CSPS sont équipés d’un téléphone cellulaire et de cartes de téléphone prépayées. Bien que les travailleurs de la santé ne parlent pas de santé mobile, les téléphones jouent un rôle important dans les activités quotidiennes des CSPS depuis quelques années. La communication avec Nouna et l’hôpital de district reste peut-être l’utilisation la plus fréquente des téléphones dans les CSPS. Les travailleurs de la santé s’en servent pour transférer des données épidémiologiques à l’administration du district toutes les semaines dans le cadre d’un programme national de surveillance des maladies. Ils s’en servent également pour appeler une ambulance afin de transférer des patients à l’hôpital de district lorsqu’ils ne peuvent les soigner au CSPS. Le téléphone est également utilisé pour prendre des rendez-vous médicaux. De plus, quand un CSPS est à court d’un médicament, on appelle la pharmacie de Nouna pour savoir si elle l’a en stock. Les travailleurs de la santé appellent parfois des patients qui ont un téléphone cellulaire pour s’informer de leur état ou de celui d’un membre de leur famille. Ces appels peuvent parfois remplacer un rendez-vous de suivi. En règle générale, les travailleurs de la santé considèrent que les téléphones cellulaires facilitent la transmission de renseignements sur la santé entre les CSPS éloignés, leurs collègues de Nouna et, dans une certaine mesure, leurs patients et leur famille.

1. **Avantages escomptés : ce que la santé mobile peut faire**

On remet un carnet de santé à toutes les femmes enceintes qui se présentent à un CSPS pour une consultation. Les travailleurs de la santé y notent leurs prochains rendez-vous de soins prénataux pour faciliter le suivi médical. Toutefois, la plupart des femmes du DSN sont illettrées et ont de la difficulté à lire les dates de rendez-vous de soins prénataux inscrites dans leur carnet. Comme un ICP l’a fait remarquer, en parlant des rendez-vous manqués, *« la plupart du temps, ce n’est pas une question de mauvaise foi, mais d’analphabétisme et d’oubli ».* (ICP, CSPS Labarani). Les femmes enceintes et les travailleurs de la santé élaborent de nombreuses stratégies pour composer avec la situation. Dans bien des cas, les femmes demandent à un membre de leur famille qui sait lire, comme un enfant scolarisé, de leur lire la date :

*« La date est écrite dans le carnet. Il faut donc la mémoriser. Pour ce faire, en arrivant à la maison, je dis la date à mon mari, mais je la montre aussi à mon fils, qui sait lire, puisqu’elle est écrite. Je peux donc savoir quand mon prochain rendez-vous aura lieu. »* (Femme enceinte)

Une autre stratégie utilisée par les travailleurs de la santé consiste à demander aux femmes enceintes de revenir pour leur prochain rendez-vous de soins prénataux quand elles auront pris tous les suppléments de fer quotidiens (30 comprimés) qu’on leur a prescrits. Quand ces stratégies ne fonctionnent pas, les travailleurs de la santé essaient parfois de communiquer avec les femmes qui ne se sont pas présentées à leur rendez-vous de soins prénataux en passant par d’autres membres de la communauté, en commençant par les *agents de santé communautaire* (ASC). Les agents de santé communautaire sont des bénévoles que l’on trouve dans tous les villages. Ils jouent un rôle important en transmettant à la population locale de l’information sur des questions et des événements de santé. Les travailleurs de la santé appellent parfois les agents de santé communautaire pour leur demander d’informer des patientes de rendez-vous manqués.

*« Pour les patientes dont le suivi est interrompu, nous dressons la liste des femmes qui ont manqué leurs consultations de soins prénataux et nous la remettons à l’agent de santé communautaire. Il informera ces femmes. »*  (Sage-femme)

Dans certains villages, les agents de santé communautaire ont également mis en place des *cellules d’urgence* pour gérer les urgences obstétriques. Les travailleurs de la santé demandent parfois à ces cellules de recenser les femmes enceintes qui ne se présentent pas à leurs rendez-vous de soins prénataux et de leur demander de communiquer avec le CSPS.

Malgré ces solutions, trop de consultations de soins prénataux sont manquées. Les travailleurs de la santé qui participent à notre étude croient que la santé mobile et le projet MOS@N en particulier peuvent améliorer le suivi des patients et réduire le nombre de patientes dont le suivi est interrompu. On s’attend à ce que les ordinateurs fournis aux CSPS dans le cadre du projet MOS@N jouent un rôle important à ce chapitre. Comme c’est souvent le cas avec la santé mobile, le système de dossiers de santé informatisés (DSI), dans lequel les travailleurs de la santé enregistrent les patientes qui se présentent pour leur premier rendez-vous de soins prénataux, est un élément essentiel du projet. Le DSI permet aux travailleurs de la santé de suivre les rendez-vous de soins prénataux de leurs patientes.

*« Je trouve que c’est très utile, parce qu’une fois que nous avons enregistré une femme enceinte dans l’ordinateur, nous pourrons identifier les patientes dont le suivi a été interrompu. L’ordinateur nous préviendra des patients qui manquent des rendez-vous afin que nous puissions les retrouver. »* (Sage-femme)

En outre, le DSI crée automatiquement un message vocal de rappel des prochaines consultations de soins prénataux. Ces messages sont reçus par une marraine à laquelle on a affecté un groupe de patientes. Les travailleurs de la santé locaux s’attendent donc à ce que la santé mobile remplace et améliore les anciennes voies de communication plus officieuses qui passent par les ASC. Au lieu de dresser une liste de patientes à l’intention des ASC en utilisant des dossiers papier et en se fiant à leur mémoire, les travailleurs de la santé peuvent désormais laisser le DSI produire automatiquement la liste et transmettre des rappels aux marraines, dont le rôle est mieux défini que celui des ASC.

Les participants sont presque tous favorables aux marraines et au rôle qu’elles devraient jouer. *« Je crois qu’elles ont un rôle important à jouer en nous aidant à nous rappeler de la date de notre consultation. »* (Démé Salimata, femme enceinte, CSPS Dara) Les participantes croient toutefois que le rôle des marraines ne devrait pas être limité à la transmission de l’information reçue au moyen de téléphones cellulaires. Elles croient plutôt que la santé mobile devrait être considérée comme un élément fondamental d’un réseau conçu pour sensibiliser à la santé maternelle et les aider pendant leur grossesse. Par exemple, certaines femmes ont mentionné que les marraines pourraient collaborer plus étroitement avec les travailleurs de la santé pour se former et diffuser ce savoir au sein de leur communauté. Elles s’attendent à ce que la santé mobile aboutisse à la création d’un réseau de soutien qui compléterait les ressources existantes. Les marraines conviennent, quant à elles, que leur rôle ne se limite pas à la transmission d’information sur les prochains rendez-vous de soins prénataux. Une marraine a résumé cette façon générale de voir les choses de la manière suivante :

*« Notre rôle consiste à offrir des conseils afin que les femmes se rendent au CSPS et à fournir de l’information à la population. […] J’ai accepté ce rôle parce que nous servons d’intermédiaire entre les travailleurs de la santé et notre communauté. »* (Marraine)

La plupart des travailleurs de la santé partagent cette idée de jouer un rôle d’intermédiaire. Voici ce que deux ICP avaient à dire à ce sujet :

*« Étant donné que les marraines vivent au sein des communautés, ces dernières se sentiront plus concernées par nos activités et comprendront encore mieux que le CSPS leur appartient. »* (ICP)

*« Elles sont extrêmement utiles, puisqu’ici, au CSPS, nous ne connaissons aucun membre de leur communauté. Comme elles vivent dans les villages, elles peuvent devenir notre bouche et nos oreilles auprès de la population. […] Je suis un étranger ici, mais elles connaissent tout le monde. Qui d’autre pourrait les convaincre de participer à nos activités ? »* (ICP)

Autrement dit, tous les participants considèrent le téléphone cellulaire comme un outil contribuant à établir de nouvelles relations avec les CSPS. On s’attend à ce que la santé mobile transforme les pratiques locales favorables à la santé en mettant en place un réseau de soutien dans les villages et en tissant des liens communautaires.

1. **Défis prévus : éléments auxquels il faut prêter attention en matière de santé mobile**

Les participants s’attendent à ce que la mise en oeuvre de la santé mobile dans le DSN se heurte à plusieurs obstacles, que l’on peut classer en six catégories : a) les obstacles technologiques, b) les défis organisationnels, c) les problèmes de sexospécificité, d) les préoccupations relatives à la confidentialité et e) les conséquences imprévues.

1. Les participants s’attendent à ce que la mise en oeuvre de solutions de santé mobile dans le DSN nécessite de surmonter d’importants obstacles technologiques. Selon de nombreuses femmes, l’accès au réseau cellulaire et la stabilité de ce dernier dans leur village pourraient être des obstacles majeurs aux activités courantes des marraines. Dans certains villages, il n’est pas rare de ne pas avoir d’accès réseau pendant quelque temps. On a aussi mentionné que l’absence d’électricité représentait un obstacle, puisqu’elle signifie que les projets de santé mobile doivent comprendre une source d’électricité fiable. Par exemple, on fournit un chargeur à panneau solaire aux marraines dans le cadre du projet MOS@N. Toutefois, une telle solution ne règle pas forcément le problème d’alimentation électrique quand les participants sont appelés à se déplacer ou à s’absenter de la maison pour la nuit. Comme l’explique une marraine :

*« Le problème se pose quand nous devons dormir en forêt, pour travailler dans les champs. Quand la batterie est à plat, nous ne pouvons pas voyager de nuit pour la brancher au panneau. »* (Marraine)

Les obstacles technologiques s’appliquent aussi aux CSPS. Ceux-ci doivent notamment composer avec le manque d’expérience des travailleurs de la santé dans l’utilisation des ordinateurs. *« Le téléphone est facile à utiliser, contrairement aux ordinateurs. Quand on n’en a jamais eu un, une formation est nécessaire, sinon on aura de la difficulté à s’en servir. »* (Sage-femme)

1. Les participants s’attendent également à ce que la santé mobile présente des défis organisationnels. Une augmentation possible de la charge de travail des travailleurs de la santé représente le plus important de ces défis. À quelques exceptions près, les travailleurs de la santé s’attendent à ce qu’un projet comme MOS@N augmente leur charge de travail, malgré l’automatisation de certaines tâches.

*« Le projet augmentera notre charge de travail. Si nous commençons à nous servir d’ordinateurs, nous devrons quand même continuer de faire tout ce que nous faisions sur papier, avant de le transcrire à l’ordinateur. Au début, il s’agira sûrement d’un fardeau supplémentaire. »* (Sage-femme)

Les travailleurs de la santé considèrent que cette augmentation de leur charge de travail pourrait mettre en péril la réussite de la mise en oeuvre de la santé mobile, car ils n’ont déjà pas assez de temps pour réaliser leurs activités courantes. Les défis organisationnels prévus comprennent également la nécessité d’offrir une formation de qualité aux participants des projets, de veiller à ce que l’équipe de mise en oeuvre effectue un suivi rigoureux des activités de santé mobile, de définir clairement les tâches et de prendre des mesures pour entretenir la motivation des participants.

1. Les femmes locales, notamment les marraines, ont également mentionné que des questions de sexospécificité pourraient compromettre la réussite de la mise en oeuvre de la santé mobile. Comme nous l’avons mentionné plus tôt, certains maris ne leur permettent pas de posséder et d’utiliser un téléphone pour appeler. Les femmes s’attendent donc à ce que certaines marraines ne soient pas en mesure de remplir leurs obligations en raison de pressions maritales.

*« Ici, on vient de choisir les marraines, mais elles ne pourront pas faire leur travail, car leur mari les en empêchera. »* (Bagala, femme en âge de procréer)

Les marraines ont elles-mêmes mentionné que leurs tâches liées à la santé mobile pouvaient engendrer des conflits au sein de leur ménage. D’autres femmes croient que le téléphone fourni dans le cadre du projet MOS@N pourrait aboutir entre les mains de leur mari.

1. La possibilité que l’accès à des téléphones utilisés pour transmettre des renseignements de santé confidentiels puisse être contrôlé par le mari des marraines suscite évidemment toutes sortes d’inquiétudes d’un point de vue éthique. Même si l’on fait abstraction de ce scénario, de nombreuses femmes se sont dit préoccupées par la confidentialité des renseignements de santé transmis sur le réseau.

*« Je ne sais pas vraiment, mais ceux qui le savent pensent qu’il s’agit d’une bonne chose. Mais je sais que ce n’est pas entièrement positif. Ne peut-il pas s’agir d’une source de conflits ? Les téléphones peuvent révéler des secrets, et ça, ce n’est pas une bonne chose. »* (Marraine)

La confidentialité peut être particulièrement problématique, car la grossesse est souvent tenue secrète au Burkina Faso rural, surtout lorsqu’il s’agit d’une jeune femme. Quelques travailleurs de la santé ont exprimé des inquiétudes relatives à la discrétion des marraines à cet égard. D’autres ont mentionné que la confidentialité pourrait être compromise si l’information transmise sur le réseau est interceptée par les fournisseurs d’accès locaux.

1. Quelques travailleurs de la santé ont mentionné que la santé mobile pourrait avoir des conséquences imprévues à long terme. On a insinué qu’après la mise en oeuvre de MOS@N, les femmes s’attendront à obtenir un suivi médical de la part des marraines, qui s’attendront elles-mêmes à recevoir des rappels automatisés. La santé mobile pourrait finir par perturber les pratiques existantes et créer une dépendance excessive au réseau.

*« L’inconvénient, c’est que lorsque les femmes se seront habituées à ce système, elles cesseront de venir si nous ne les appelons pas. Elles attendront que nous les appelions avant de venir. Alors, si le projet n’est pas là à long terme, des problèmes se poseront avec nos patients. »* (Sage-femme)

En somme, la santé mobile pourrait avoir une incidence négative à long terme sur les comportements favorables à la santé chez les femmes enceintes sans une adoption permanente dans le cadre du système de santé local.

1. **Limites intrinsèques : ce que la santé mobile ne peut pas faire**

Lorsqu’on leur pose des questions sur les principaux obstacles à l’accès aux soins gynécologiques dans le district, les participants ne considèrent habituellement pas qu’une communication médiocre en fait partie. Par exemple, selon les travailleurs de la santé, la plupart des femmes enceintes dans le DSN sont déjà conscientes de l’importance des consultations de soins prénataux. Elles essaient aussi de ne pas manquer leurs rendez-vous. Toutefois, les femmes enceintes sont confrontées à de nombreux obstacles, et la santé mobile ne devrait pas y changer grand-chose. Les obstacles en question peuvent être classés en trois catégories, soit l’accessibilité géographique, les facteurs économiques et les inégalités entre les sexes.

Selon les participants, le plus grand obstacle aux soins gynécologiques dans le DSN est le mauvais accès géographique aux CSPS. Sachant qu’elles sont nombreuses à vivre à une distance de cinq à dix kilomètres du CSPS le plus proche, les déplacements peuvent être compliqués pour les femmes enceintes. C’est notamment le cas durant la saison des pluies, quand les routes deviennent impraticables en raison de l’accumulation de sable et d’argile, si elles ne sont pas complètement inondées pendant des semaines. La plupart des femmes se rendent au CSPS à pied ou à vélo. Quand la date d’accouchement approche, elles utilisent parfois une motocyclette. Ainsi, lorsque les routes deviennent impraticables, elles doivent renoncer à utiliser une motocyclette ou même une bicyclette. La marche aussi peut devenir périlleuse. Certains villages se retrouvent littéralement coupés de leur CSPS. Dans de telles circonstances, les travailleurs de la santé répondent sans hésitation que la saison des pluies est le facteur le plus important dans les consultations de soins prénataux et dans le nombre d’accouchements à domicile.

*« Malgré nos efforts de sensibilisation, les femmes ne viennent pas durant la saison des pluies à cause des mauvaises conditions routières. Même si nous demandons à l’ASC ou à une marraine d’aller les voir, elles ne viennent pas. Durant la saison des pluies, il y a de nombreux accouchements à domicile. »* (Sage-femme)

*« Les femmes qui ont commencé à avoir des contractions et qui partent de Kongoba ou de Karasso peuvent accoucher en chemin. Elles peuvent aussi rebrousser chemin et rentrer chez elles. Durant la saison des pluies, de nombreuses femmes ne se présentent pas à leurs consultations de soins prénataux ou de soins postnataux. »* (Sage-femme)

Quelques participants ont laissé entendre que la santé mobile peut contribuer à atténuer les effets de cet isolement.

*« L’introduction des téléphones cellulaires dans le système de santé sera une très bonne chose, parce que notre village est entouré d’eau. Pendant la saison des pluies, il nous arrive d’être isolés, et les femmes ne peuvent pas se rendre au CSPS pour leurs consultations prénatales. Grâce au téléphone, nous pourrions communiquer et trouver une solution dans ces cas-là. »* (Marraine)

De manière générale, les participants ne considèrent toutefois pas la santé mobile comme une solution adéquate au mauvais accès géographique. Ils souhaitent plutôt une augmentation du nombre de CSPS. Sinon, on s’attend à ce que l’accès géographique limite l’incidence positive de la santé mobile dans le DSN.

Les obstacles à l’accès aux soins gynécologiques comprennent aussi les difficultés économiques, qui peuvent revêtir différentes formes. Bien que les consultations de soins prénataux soient offertes gratuitement et que l’accouchement soit relativement abordable (900 francs CFA, ou 1,5 USD), les médicaments et l’hospitalisation peuvent représenter des dépenses importantes pour les ménages. Les transferts à l’hôpital de district de Nouna représentent un fardeau encore plus important. Certains participants ont expliqué que lorsqu’ils contractent une dette envers leur CSPS, à la suite de dépenses qui ne sont pas liées aux soins gynécologiques, par exemple, ils évitent parfois d’y retourner tant qu’ils ne peuvent pas le rembourser. Les moyens de subsistance des ménages de la campagne de Nouna constituent un autre obstacle économique. Comme l’a expliqué une sage-femme :

*« Des femmes enceintes se présentent régulièrement au CSPS durant la saison sèche, mais durant la saison des pluies, il se peut qu’elles viennent quand même, mais qu’elles manquent leurs rendez-vous. Elles doivent travailler aux champs, ce qui complique les choses. »* (Sage-femme)

Encore une fois, l’incidence éventuelle de la santé mobile sur ces facteurs économiques est intrinsèquement limitée.

Les inégalités entre les sexes ont également été mentionnées par les participants comme étant un obstacle à l’accès aux soins gynécologiques. Chez la plupart des ménages, les décisions en matière de santé sont essentiellement prises par des hommes.

*« Quand nous sommes enceintes, c’est notre mari qui prend les décisions concernant notre grossesse. Nous partageons certaines responsabilités, mais la décision finale appartient au mari, qui peut nous amener au CSPS si nous sommes malades. »* (Toni, femme en âge de procréer,)

La plupart des femmes ont mentionné que leur capacité à payer des soins de santé dépend de la volonté de leur mari. Bien qu’il semble que la plupart des maris estiment que les soins maternels sont importants, d’autres ne prennent pas convenablement soin de leur femme ou ne les encouragent pas à se rendre au CSPS.

*« C’est pour leur propre santé que les femmes se rendent au CSPS pour des consultations prénatales. Si une femme a un vélo, elle peut s’en servir. Sinon, elle doit marcher. Ici, les maris ne disent pas à leur femme d’aller au CSPS pour leur consultation. »* (Sobon, femme en âge de procréer)

Une autre femme explique que les maris refusent parfois de payer des médicaments ou insistent pour qu’elles travaillent au lieu de se rendre au CSPS.

*« Si nous avons de l’argent, nous pouvons payer nos propres médicaments, mais sinon, nous rentrons à la maison avec l’ordonnance que nous remettons à notre mari pour qu’il paie. Puis, notre mari nous dira qu’il ne nous a jamais donné la permission d’aller au CSPS. Pour cette raison, de nombreuses femmes ne veulent pas se rendre au CSPS. »* (Sobon, femme en âge de procréer)

Les inégalités entre les sexes contribuent ainsi à éloigner certaines femmes enceintes des CSPS. Bien que l’incidence de la santé mobile sur les décisions déterminées par le sexe au sein des ménages nécessite un examen plus approfondi, il ne s’agit pas d’une attente relative à la santé mobile exprimée par la plupart des participants. Comme nous l’avons mentionné plus tôt, on s’attend parfois à ce que les inégalités entre les sexes soient un obstacle à la réussite de la santé mobile dans le DSN.

**Discussion**

Nos résultats indiquent que les attentes relatives à la santé mobile dans le DSN devraient être considérées comme des connaissances contextuelles, ce qui complique le savoir universel et la collecte de données probantes relatifs à la santé mobile. [65, 66] Comme nous l’avons mentionné plus tôt, il n’y a pratiquement pas, à notre connaissance, de recherche sur les attentes locales relatives à la santé mobile en Afrique subsaharienne, si l’on fait abstraction de l’intérêt habituellement manifesté pour l’acceptabilité et à la faisabilité. Les résultats de la présente étude ne sont toutefois pas surprenants. Depuis un certain temps déjà, les chercheurs des sciences sociales ont employé des méthodes qualitatives pour montrer que le savoir relatif à la santé et à la maladie dépend toujours du lieu et de l’époque. [67-69] En parlant de connaissances contextuelles, on insiste sur le fait qu’elles représentent la compréhension dont les acteurs locaux disposent d’un phénomène donné construit dans une communauté de praticiens. [70] Cela s’applique aux attentes relatives à la santé mobile dans le DSN, ainsi qu’à ses avantages, défis et limites.

En règle générale, les personnes qui ont participé à l’étude s’attendent à ce que la santé mobile ait des retombées positives et concrètes. Les travailleurs de la santé croient que la santé mobile améliorera le suivi des patientes en les aidant à recenser les femmes enceintes qui ont manqué des rendez-vous de soins prénataux. Ils insistent aussi sur le rôle des ordinateurs et du DSI dans l’automatisation des rappels de rendez-vous et l’amélioration du suivi des patientes. Il convient toutefois de noter que les participants ne voient pas la santé mobile, et le projet MOS@N en particulier, comme une intervention technologique en soi. Autrement dit, le principal apport de la santé mobile n’est pas un nombre quelconque de messages envoyés et reçus grâce à un réseau technologique donné, mais plutôt l’établissement d’un réseau de soutien qui va de pair avec les comportements favorables à la santé et les contraintes connexes qui existent déjà. À ce chapitre, l’un des besoins locaux pressants que les participants associent à la santé mobile est le renforcement des relations entre les CSPS et les communautés. Cela est particulièrement vrai dans le cas du projet MOS@N, puisque celui-ci est fondé sur la participation de marraines, des agents de liaison communautaire qui se consacrent aux soins gynécologiques. On ne s’attend donc pas à ce que le téléphone cellulaire soit un simple instrument de transmission de l’information, mais à ce qu’il aide les marraines à jouer un rôle de médiateur entre les CSPS et les communautés. Ce travail de médiation devrait aboutir à la création d’un réseau, et non l’inverse, réseau qui aura une influence positive sur les comportements favorables à la santé des femmes enceintes qui vivent dans le DSN. En suggérant que ces avantages prévus sont contextuels, on souligne le fait qu’ils représentent des connaissances collectives, partielles, changeantes et pratiques, qui sont façonnées par les situations et les réseaux auxquels les gens participent. [71]

Cela vaut également pour les défis prévus. Les défis prévus, qu’ils soient de nature technologique, organisationnelle ou sexospécifique, ou qu’ils soient liés à la confidentialité, doivent être considérés du point de vue des expériences (comme le manque d’expérience avec la technologie), des pratiques (charge de travail, nuit dans les champs, etc.), des contraintes quotidiennes (manque d’électricité, d’accès réseau, etc.) et des relations de pouvoir (inégalités entre les sexes, etc.) qui constituent l’univers personnel des participants. Si l’on n’accorde pas une attention suffisante à ces expériences, on risque de mal interpréter les défis imminents et, en fin de compte, d’obtenir une conception de projet et des résultats décevants. [35, 41, 72] Si nous prenons les relations entre les sexes comme exemple, une analyse documentaire de l’influence des interventions de santé mobile sur les relations entre les sexes dans les pays en développement révèle que les projets peuvent entraîner une augmentation de l’autonomie des femmes dans la recherche de services de santé, mais que les projets qui ne sont pas soigneusement conçus peuvent accentuer les inégalités sur le plan du pouvoir. [73]

Les résultats de la présente étude constituent une mise en garde contre les tentatives de concevoir des projets de santé mobile qui ne tiennent pas compte des particularités des sites où ils sont censés être réalisés et des situations qu’ils sont censés améliorer. Cela est d’autant plus important dans le cas des projets de santé mobile qui visent à modifier les stratégies de recours aux soins de santé locaux, comme le projet MOS@N. Comme les chercheurs universitaires en sciences sociales l’on si bien démontré, les stratégies de recours aux soins de santé sont influencées par de nombreux facteurs, comme la perception et l’expérience de la maladie, les variations saisonnières, les expériences passées relatives aux services de santé, le soutien familial et la condition socioéconomique. [74-76] De toute évidence, les comportements favorables à la santé au Burkina Faso rural n’y font pas exception. [52, 77, 78] Par exemple, une étude a révélé qu’un retard dans le recours à des services de santé offerts par des personnes compétentes durant les grossesses au Burkina Faso est causé par de nombreux facteurs, dont la pauvreté et le manque d’autonomisation des femmes, qui jouent souvent les seconds violons dans le processus décisionnel. [79] Nos participants, dont le point de vue correspond à celui exposé dans ce document, ne s’attendent pas à ce que la santé mobile modifie les comportements favorables à la santé si l’on ne s’occupe pas des facteurs sous-jacents. Dans le cas de l’accessibilité géographique, par exemple, il ne suffit pas toujours d’être au courant d’un rendez-vous de soins prénataux à venir. Les femmes enceintes doivent quand même marcher jusqu’au CSPS, souvent dans des conditions difficiles. De nombreuses études ont démontré que la distance à parcourir pour se rendre dans un établissement de santé est un facteur déterminant dans le recours aux soins gynécologiques, ce qui comprend la probabilité que l’on cherche à obtenir des soins prénataux et à accoucher dans un établissement de santé du Burkina Faso. [52, 80, 81]

Les limites prévues par les participants correspondent aussi à l’analyse documentaire, qui indique que la santé mobile est très prometteuse en ce qui a trait à l’amélioration de l’utilisation et de la qualité des services de santé maternelle, à condition que les femmes ne soient pas restreintes en raison de la place qu’elles occupent dans la société, d’un manque de fonds ou de problèmes de transport. [25] Par exemple, des études ont montré que si les téléphones cellulaires peuvent améliorer les liens entre les femmes enceintes et leur fournisseur de soins primaires, d’autres obstacles, comme la distance géographique, la pauvreté, la qualité des soins et des facteurs socioculturels, peuvent influencer la manière dont on accueille les interventions qui comprennent l’utilisation de téléphones cellulaires. [23] Cela correspond aussi aux recherches plus générales en matière de santé mobile, qui suggèrent que l’incidence de la santé mobile sur le respect des traitements, ce qui comprend les rappels de rendez-vous, est limitée dans les régions où l’accès aux services de santé est limité ou intermittent. [31] La santé mobile peut compléter, mais ne peut pas remplacer un accès adéquat aux soins de santé.

Dans le cas du projet MOS@N, les données recueillies dans le cadre de la première phase du projet nous ont permis de modifier le projet de manière à l’harmoniser avec les attentes locales relatives aux avantages, aux défis et aux limites. Dans un souci de concision, nous mentionnerons les trois principaux domaines dans lesquels ces attentes seront prises en considération. Premièrement, les données présentées ici nous ont convaincus de la nécessité de prêter une attention particulière à la relation entre la santé mobile et les inégalités entre les sexes. Nous avons donc été particulièrement attentifs aux possibles effets néfastes du projet MOS@N à ce chapitre en poursuivant sa mise en oeuvre et sa surveillance. Nous avons également mené des entrevues portant précisément sur ce sujet, et qui n’étaient pas prévues au départ, plus tard dans l’étude, notamment auprès des maris des marraines. Ces entrevues sont en cours d’analyse et devraient aboutir à d’autres publications. Deuxièmement, on a été confronté, dans le cadre du projet MOS@N, à certains des défis technologiques que les participants de l’étude avaient anticipés. L’infrastructure technique de MOS@N a donc subi des modifications substantielles depuis le lancement du projet. [45] Certaines des caractéristiques technologiques plus évoluées ont été abandonnées, et nous avons dû fignoler et remanier le projet pour en assurer la poursuite. Troisièmement, le rôle des marraines a subi une transformation importante à la suite des entrevues initiales. Les marraines, dont le rôle consistait initialement à recevoir et à transmettre des messages, ont progressivement assumé un rôle plus important dans le soutien des femmes enceintes. Par exemple, la plupart des marraines a décidé d’accompagner les femmes enceintes à leurs rendez-vous et d’aider les sages-femmes durant les accouchements. En nous appuyant sur les données recueillies ici, nous avons encouragé ces initiatives, qui n’étaient toutefois pas prévues au départ. Bien que nous examinions toujours l’incidence de cette transformation du rôle des marraines, les données présentées nous ont convaincus que ce rôle accru répond mieux aux besoins des communautés dans lesquelles le projet MOS@N est mis en oeuvre. Ce nouveau rôle a des répercussions sur tout le projet MOS@N, puisqu’il semble que la technologie mobile, malgré son importance, ne soit plus qu’un aspect d’un réseau de soutien bien plus vaste à l’échelle des villages. Par conséquent, il se pourrait que les modifications apportées au projet MOS@N nous ont permis, du moins partiellement, de contourner certaines des limites de la santé mobile afin d’améliorer des comportements favorables à la santé.

**Limites**

L’étude comporte des limites. Premièrement, il se peut que le fait que les données aient été recueillies dans le cadre d’un projet de santé mobile (MOS@N) ait eu une influence sur les attentes des participants en matière de santé mobile. Il en va probablement ainsi chez les participants qui se préparent à être des utilisateurs actifs de MOS@N (marraines et travailleurs de la santé) ou qui pourraient en profiter (femmes enceintes). On ne peut donc pas prétendre que l’étude témoigne des attentes de l’ensemble de la population du DSN en matière de santé mobile, car de nombreux participants ont été choisis en raison de leur participation au projet MOS@N. Le fait que les femmes qui participent à l’étude aient été recrutées dans les CSPS locaux représente une autre limite. Cette procédure d’échantillonnage a facilité le recrutement des participants en nous permettant de recueillir des données avant la mise en oeuvre complète de MOS@N. Cependant, les femmes qui n’utilisent pas les services de CSPS ont été écartées de l’étude. Il est permis de croire que ces femmes ont une idée différente des projets de santé mobile qui visent à les encourager à recevoir des soins gynécologiques dans les CSPS. De plus, il se peut que le fait de recruter les femmes dans les CSPS ait influencé leur discours sur les CSPS concernant la qualité des soins offerts, par exemple. Il se peut aussi que l’étude soit limitée par le fait que la plupart des entrevues aient été réalisées par des hommes, ce qui peut avoir influencé le discours des participantes à propos notamment des relations entre les sexes. Enfin, étant donné que les entrevues (à l’exception des travailleurs de la santé) ont été effectuées en dioula, il est possible que le sens de certaines expressions locales et certains propos aient été déformés dans le processus de traduction.

**Conclusion**

La présente étude indique que les attentes relatives à la santé mobile devraient être considérées comme des connaissances collectives, partielles et pratiques qui sont façonnées par le contexte et les réseaux auxquels les personnes participent. Elles ne sont pas immuables; il s’agit plutôt d’expressions dynamiques de la manière dont les communautés locales recensent, expliquent et gèrent les problèmes de santé, et ces manières de faire sont indissociables des expériences, des pratiques et des contraintes propres à chaque communauté. En insistant ici sur la nature contextuelle des attentes relatives à la santé mobile, on complique l’adoption d’approches universelles de la santé mobile, qui tendent à supposer une rationalité supérieure dont découlent des attentes et des objectifs universels, même lorsque l’on tient compte du contexte local. Il faut donc faire preuve de prudence dans l’élaboration des politiques et la conception des projets, et tenir compte des connaissances et des pratiques des communautés locales. En fin de compte, les conclusions de cette étude pourraient aider à orienter la conception et la mise en oeuvre des projets de santé mobile, ce qui maximisera leurs chances de réussite.

**Liste des abréviations utilisées**

**ASC :** Agent de santé communautaire

**CRSN :** Centre de recherche en santé de Nouna

**CSPS :** Centre de santé et de promotion sociale

**DSI :** Dossier de santé informatisé

**DSN :** District sanitaire de Nouna

**HD :** Hôpital de district

**ICP :** Infirmier-chef de poste

**ONG :** Organisation non gouvernementale

**PFR-PRI :** Pays à faible revenu et pays à revenu intermédiaire

**SP :** Soins prénataux

**Déclarations**

**Contribution des auteurs**

Tous les auteurs ont participé activement à l’étude. VD a travaillé à la conception de l’étude, ainsi qu’à la collecte et à l’analyse des données, et a rédigé le manuscrit initial. MY a coordonné l’étude, a participé à sa conception et a supervisé la collecte des données. KM a participé à la conception de l’étude et à la collecte et à l’analyse des données. HS a participé à la conception de l’étude et à la collecte et à l’analyse des données. HS a participé à la conception de l’étude et à la collecte des données. GB a participé à la conception de l’étude et à l’analyse des données. AS a participé à la supervision de l’étude et a fourni un soutien institutionnel essentiel.

**Remerciements**

Les auteurs souhaitent tout d’abord remercier le Centre de recherches pour le développement international (CRDI/Canada) pour son soutien continu à toutes les phases ayant mené à cette publication. Ils souhaitent aussi souligner le travail essentiel accompli par les chercheurs et le personnel de soutien du CRSN. Ils tiennent spécialement à souligner le travail de l’équipe de recherche du projet MOS@N. Les auteurs désirent aussi remercier toutes les personnes qui ont aimablement accepté de donner de leur temps au cours des entrevues.

**Approbation éthique et consentement à la participation**

Conformément à des lignes directrices strictes en matière d’éthique, les objectifs et les enjeux de l’étude ont été exposés clairement à tous les participants, qui ont donné leur consentement avant d’être interrogés. On a lu un formulaire de consentement à tous les participants, qui l’ont signé ou y ont apposé une empreinte digitale. Le comité d’éthique du ministère de la Santé du Burkina Faso et le conseil éthique de recherche institutionnel du CRSN ont approuvé l’éthique de l’étude.

**Consentement à la publication**

Sans objet.

**Disponibilité des données et des documents**

Sans objet.

**Intérêts conflictuels**

Les auteurs déclarent n’avoir aucun conflit d’intérêts.

**Financement**

L’étude a été financée par le Centre de recherches pour le développement international (CRDI/Canada) en vertu de l’accord de subvention 106229-009/2013 dans le cadre des

programmes SEARCH (Promotion de l’équité au moyen du renforcement des capacités de recherche appliquée en cybersanté) dans les pays à faible revenu et à revenu intermédiaire. Vincent Duclos a également reçu une bourse de recherche Steinberg en santé mondiale attribuée par les programmes en santé mondiale de l’Université McGill.

**Références**

1. WHO: *mHealth: New horizons for health through mobile technologies*. Geneva; 2011.

2. mHealth Alliance: *Five Years of Mobilizing for Health Impact: Key Achievements & Future Opportunities* Washington, DC; 2013.

3. Chib A, van Velthoven MH, Car J: **mHealth Adoption in Low-Resource Environments: A Review of the Use of Mobile Healthcare in Developing Countries.** *Journal of Health Communication* 2015, **20**: 4-34.

4. Royston G, Hagar C, Long L-A, McMahon D, Pakenham-Walsh N, Wadhwani N: **Mobile health-care information for all: a global challenge.** *The Lancet Global Health* 2015, **3**: e356-e357.

5. Piette JD, Lun KC, Moura LA, Jr., Fraser HS, Mechael PN, Powell J, Khoja SR: **Impacts of e-health on the outcomes of care in low- and middle-income countries: where do we go from here?** *Bull World Health Organ* 2012, **90**: 365-372.

6. Aranda-Jan CB, Mohutsiwa-Dibe N, Loukanova S: **Systematic review on what works, what does not work and why of implementation of mobile health (mHealth) projects in Africa.** *BMC Public Health* 2014, **14**: 188-188.

7. Madon S, Amaguru JO, Malecela MN, Michael E: **Can mobile phones help control neglected tropical diseases? Experiences from Tanzania.** *Social Science & Medicine* 2014, **102**: 103-110.

8. GSMA: *The Mobile Economy: Sub-Saharan Africa 2015*. London, UK; 2015.

9. Labrique AB, Vasudevan L, Kochi E, Fabricant R, Mehl G: **mHealth innovations as health system strengthening tools: 12 common applications and a visual framework.** *Global Health: Science and Practice* 2013, **1**: 160-171.

10. Wakadha H, Chandir S, Were EV, Rubin A, Obor D, Levine OS, Gibson DG, Odhiambo F, Laserson KF, Feikin DR: **The feasibility of using mobile-phone based SMS reminders and conditional cash transfers to improve timely immunization in rural Kenya.** *Vaccine* 2013, **31**: 987-993.

11. Mbuagbaw L, Thabane L, Ongolo-Zogo P, Lester RT, Mills EJ, Smieja M, Dolovich L, Kouanfack C: **The Cameroon Mobile Phone SMS (CAMPS) Trial: A Randomized Trial of Text Messaging versus Usual Care for Adherence to Antiretroviral Therapy.** *PLoS ONE* 2012, **7**: e46909.

12. Agarwal S, Perry HB, Long LA, Labrique AB: **Evidence on feasibility and effective use of mHealth strategies by frontline health workers in developing countries: systematic review.** *Trop Med Int Health* 2015, **20**: 1003-1014.

13. Lemay NV, Sullivan T, Jumbe B, Perry CP: **Reaching remote health workers in Malawi: baseline assessment of a pilot mHealth intervention.** *J Health Commun* 2012, **17 Suppl 1**: 105-117.

14. Medhanyie AA, Little A, Yebyo H, Spigt M, Tadesse K, Blanco R, Dinant G-J: **Health workers’ experiences, barriers, preferences and motivating factors in using mHealth forms in Ethiopia.** *Human Resources for Health* 2015, **13**: 2.

15. Brinkel J, Kramer A, Krumkamp R, May J, Fobil J: **Mobile phone-based mHealth approaches for public health surveillance in sub-Saharan Africa: a systematic review.** *Int J Environ Res Public Health* 2014, **11**: 11559-11582.

16. Kaplan WA: **Can the ubiquitous power of mobile phones be used to improve health outcomes in developing countries?** *Globalization and Health* 2006, **2**: 9-9.

17. Lewis T, Synowiec C, Lagomarsino G, Schweitzer J: **E-health in low- and middle-income countries: findings from the Center for Health Market Innovations.** *Bull World Health Organ* 2012, **90**: 332 - 340.

18. Kahn JG, Yang JS, Kahn JS: **‘Mobile’ Health Needs And Opportunities In Developing Countries.** *Health Affairs* 2010, **29**: 252-258.

19. Mechael P, Batavia H, Kaonga N, Searle S, Kwan A, Goldberger A, Fu L, Ossman J: *Barriers and gaps affecting mHealth in low and middle income countries: Policy white paper.* Columbia university. Earth institute. Center for global health and economic development (CGHED): with mHealth alliance; 2010.

20. Al Dahdah M, Desgrées Du Loû A, Méadel C: **Mobile health and maternal care: A winning combination for healthcare in the developing world?** *Health Policy and Technology* 2015, **4**: 225-231.

21. Ngabo F, Nguimfack J, Nwaigwe F, Mugeni C, Muhoza D, Wilson DR, Kalach J, Gakuba R, Karema C, Binagwaho A: **Designing and Implementing an Innovative SMS-based alert system (RapidSMS-MCH) to monitor pregnancy and reduce maternal and child deaths in Rwanda.** *Pan Afr Med J* 2012, **13**: 31.

22. Andreatta P, Debpuur D, Danquah A, Perosky J: **Using cell phones to collect postpartum hemorrhage outcome data in rural Ghana.** *Int J Gynaecol Obstet* 2011, **113**: 148-151.

23. Lund S, Hemed M, Nielsen BB, Said A, Said K, Makungu MH, Rasch V: **Mobile phones as a health communication tool to improve skilled attendance at delivery in Zanzibar: a cluster-randomised controlled trial.** *Bjog* 2012, **119**: 1256-1264.

24. Moahi KH: **ICT and health information in Botswana: Towards the Millennium Development Goals.** *Information Development* 2009, **25**: 198-206.

25. Noordam AC, Kuepper BM, Stekelenburg J, Milen A: **Improvement of maternal health services through the use of mobile phones.** *Tropical Medicine & International Health* 2011, **16**: 622-626.

26. World Bank: *Information and Communications for Development 2012: Maximizing Mobile*. Washington, DC; 2012.

27. Jennings L, Omoni A, Akerele A, Ibrahim Y, Ekanem E: **Disparities in mobile phone access and maternal health service utilization in Nigeria: A population-based survey.** *International Journal of Medical Informatics International Journal of Medical Informatics* 2015, **84**: 341-348.

28. Agarwal S, LeFevre AE, Lee J, L’Engle K, Mehl G, Sinha C, Labrique A: **Guidelines for reporting of health interventions using mobile phones: mobile health (mHealth) evidence reporting and assessment (mERA) checklist.** *BMJ* 2016, **352**.

29. Tomlinson M, Rotheram-Borus MJ, Swartz L, Tsai AC: **Scaling Up mHealth: Where Is the Evidence?** *PLoS Med* 2013, **10**: e1001382.

30. Labrique A, Vasudevan L, Chang LW, Mehl G: **H_pe for mHealth: More “y” or “o” on the horizon?** *International journal of medical informatics* 2013, **82**: 10.1016/j.ijmedinf.2012.1011.1016.

31. Mechael P, Batavia H, Kaonga N, Searle S, Kwan A, Fu L, Ossman J: **Barriers and Gaps Affecting mHealth in Low and Middle Income Countries: Policy White Paper.** 2010.

32. Amoroso C, Flores Arango J, Bailey C: Call to action on global eHealth evaluation. In *Consensus Statement of the WHO Global eHealth Evaluation Meeting Bellagio: The Bellagio eHealth Evaluation Group*. 2011

33. Haberer JE, Kiwanuka J, Nansera D, Wilson IB, Bangsberg DR: **Challenges in using mobile phones for collection of antiretroviral therapy adherence data in a resource-limited setting.** *AIDS Behav* 2010, **14**: 1294-1301.

34. Palazuelos D, Diallo AB, Palazuelos L, Carlile N, Payne JD, Franke MF: **User Perceptions of an mHealth Medicine Dosing Tool for Community Health Workers.** *JMIR Mhealth Uhealth* 2013, **1**: e2.

35. Chaiyachati KH, Loveday M, Lorenz S, Lesh N, Larkan L-M, Cinti S, Friedland GH, Haberer JE: **A Pilot Study of an mHealth Application for Healthcare Workers: Poor Uptake Despite High Reported Acceptability at a Rural South African Community-Based MDR-TB Treatment Program.** *PLoS ONE* 2013, **8**: e64662.

36. Kleinman A: *Patients and healers in the context of culture: An exploration of the borderland between anthropology, medicine, and psychiatry.* Univ of California Press; 1980.

37. Nyamongo I: **Health care switching behaviour of malaria patients in a Kenyan rural community.** *Social science & medicine* 2002, **54**: 377-386.

38. Kroeger A: **Anthropological and socio-medical health care research in developing countries.** *Social Science & Medicine* 1983, **17**: 147-161.

39. Jensen CB: **Technologic: Conceptualising Health Care Transformation With the Electronic Patient Record.** *Systems, Signs & Actions* 2006, **2**: 41-59.

40. Sánchez-Criado T, López D, Roberts C, Domènech M: **Installing Telecare, Installing Users: Felicity Conditions for the Instauration of Usership.** *Science, Technology & Human Values* 2014.

41. Duclos V: **The map and the territory: an ethnographic study of the low utilisation of a global eHealth network.** *J Inf technol* 2016.

42. Oudshoorn N: **How places matter: Telecare technologies and the changing spatial dimensions of healthcare.** *Social Studies of Science* 2012, **42**: 121-142.

43. Bibeau G: **At work in the fields of public health: the abuse of rationality.** *Medical anthropology quarterly* 1997, **11**: 246-251.

44. Yé M, Cheik Bagagan, Millogo O, Tinto I, Sié A, Vincent D, Gilles B: **Use of Mobile Phone to Promote Governance and Equity within the Health System: Experience of Rural Health District in Burkina Faso.** *Journal of Healthcare Communications* 2016, **1**: 17.

45. Sanou H, Yé M, Duclos V, Kagoné M, Brice B, Tinto I, Millogo O, Bagagan C, Zabré P, Sié A, Bibeau G: **Notes sur le processus de mise en place d’une plateforme de santé mobile: design, défis et perspectives à venir.** *Cahiers REALISME* 2016, **10**: 1-32.

46. Becher H, Becher H, Muller O, Dambach P, Gabrysch S, Sankoh O, Schoeps A, Stieglbauer G, Niamba L, Simboro S, et al: **Decreasing child mortality, spatial clustering and decreasing disparity in North-Western Burkina Faso.** *Trop Med Int Health Tropical Medicine and International Health* 2016, **21**: 546-555.

47. Ridde V, Richard F, Bicaba A, Queuille L, Conombo G: **The national subsidy for deliveries and emergency obstetric care in Burkina Faso.** *Health Policy and Planning* 2011, **26**: ii30-ii40.

48. Institut national de la statistique et de la démographie (INSD): *Annuaire Statistique 2013*. Ouagadougou; 2014.

49. Amnesty International: *Giving Life, Risking Death: Maternal Mortality in Burkina Faso*. London, UK; 2009.

50. Belaid L, Ridde V: **Contextual factors as a key to understanding the heterogeneity of effects of a maternal health policy in Burkina Faso?** *Health Policy and Planning* 2014.

51. Cocking C, Flessa S, Reinelt G: **Improving access to health facilities in Nouna district, Burkina Faso.** *SEPS Socio-Economic Planning Sciences* 2012, **46**: 164-172.

52. De Allegri M, Ridde V, Louis VR, Sarker M, Tiendrebéogo J, Yé M, Müller O, Jahn A: **Determinants of utilisation of maternal care services after the reduction of user fees: a case study from rural Burkina Faso.** *Health Policy* 2011, **99**: 210-218.

53. Prytherch H, Kagoné M, Aninanya GA, Williams JE, Kakoko DC, Leshabari MT, Yé M, Marx M, Sauerborn R: **Motivation and incentives of rural maternal and neonatal health care providers: a comparison of qualitative findings from Burkina Faso, Ghana and Tanzania.** *BMC health services research* 2013, **13**: 1.

54. Palys T: **Purposive sampling.** In *The Sage Encyclopedia of Qualitative Research Methods (Vol 2).* Edited by Given LM. Los Angeles, Sage; 2008: 697-698

55. WHO: *How to investigate the use of medicines by consumers*. Geneva; 2004.

56. Low J: **Unstructured and semi-structured interviews in health research.** In *Researching Health: Qualitative, quantitative and mixed methods.* Edited by Saks M, Allsop J. Cornwall, Sage Publications; 2012: 87

57. Bryman A: *Social Research Methods 4th Edition.* Oxford and New York, Oxford University Press; 2012.

58. Kvale S: **The qualitative research interview: A phenomenological and a hermeneutical mode of understanding.** *Journal of Phenomenological Psychology* 1983, **14**: 171-196.

59. Gill P, Stewart K, Treasure E, Chadwick B: **Methods of data collection in qualitative research: interviews and focus groups.** *Br Dent J* 2008, **204**: 291-295.

60. Leung F-H, Savithiri R: **Spotlight on focus groups.** *Canadian Family Physician* 2009, **55**: 218-219.

61. Vaismoradi M, Turunen H, Bondas T: **Content analysis and thematic analysis: Implications for conducting a qualitative descriptive study.** *Nursing & Health Sciences* 2013, **15**: 398-405.

62. Elo S, Kyngas H: **The qualitative content analysis process.** *J Adv Nurs* 2008, **62**: 107-115.

63. Braun V, Clarke V: **Using thematic analysis in psychology.** *Qualitative Research in Psychology* 2006, **3**: 77-101.

64. Emerson R, Fretz R, Shaw L: **Processing Fieldnotes: Coding and Memoing.** In *Writing Ethnographic Fieldnotes.* Edited by Emerson R, Fretz R, Shaw L. Chicago, University of Chicago Press; 1995

65. Haraway D: **Situated knowledges: The science question in feminism and the privilege of partial perspective.** *Feminist studies* 1988, **14**: 575-599.

66. Nygren A: **Local Knowledge in the Environment-Development Discourse: From dichotomies to situated knowledges.** *Critique of Anthropology* 1999, **19**: 267-288.

67. Lock M, Nguyen VK: *An Anthropology of Biomedicine.* Malden, MA, Wiley-Blackwell; 2010.

68. Good B: *A reader in medical anthropology : theoretical trajectories, emergent realities.* Chichester, West Sussex, UK; Malden, Mass., Wiley-Blackwell; 2010.

69. Nichter M: *Global health: Why cultural perceptions, social representations, and biopolitics matter.* University of Arizona Press; 2008.

70. Clarke A, Montini T: **The many faces of RU486: Tales of situated knowledges and technological contestations.** *Science, Technology & Human Values* 1993, **18**: 42-78.

71. Blackler F: **Knowledge, Knowledge Work and Organizations: An Overview and Interpretation.** *Organization Studies* 1995, **16**: 1021-1046.

72. Heeks R: **Health information systems: Failure, success and improvisation.** *International Journal of Medical Informatics* 2006, **75**: 125-137.

73. Jennings L, Omoni A, Akerele A, Ibrahim Y, Ekanem E: **Disparities in mobile phone access and maternal health service utilization in Nigeria: A population-based survey.** *International Journal of Medical Informatics* 2015, **84**: 341-348.

74. Boonmongkon P, Nichter M, Pylypa J: **Mot Luuk problems in northeast Thailand: why women’s own health concerns matter as much as disease rates.** *Social Science & Medicine* 2001, **53**: 1095-1112.

75. Kolling M, Winkley K, von Deden M: **“For someone who’s rich, it’s not a problem”. Insights from Tanzania on diabetes health-seeking and medical pluralism among Dar es Salaam’s urban poor.** *Globalization and Health* 2010, **6**: 1-9.

76. Fortin S, Gauthier A, Gomez L, Faure C, Bibeau G, Rasquin A: **Uncertainty, culture and pathways to care in paediatric functional gastrointestinal disorders.** *Anthropology & medicine* 2013, **20**: 311-323.

77. Sauerborn R, Nougtara A, Hien M, Diesfeld HJ: **Seasonal variations of household costs of illness in Burkina Faso.** *Social Science & Medicine* 1996, **43**: 281-290.

78. Beiersmann C, Sanou A, Wladarsch E, De Allegri M, Kouyaté B, Müller O: **Malaria in rural Burkina Faso: local illness concepts, patterns of traditional treatment and influence on health-seeking behaviour.** *Malaria Journal* 2007, **6**: 1.

79. Somé DT, Sombié I, Meda N: **How decision for seeking maternal care is made-a qualitative study in two rural medical districts of Burkina Faso.** *Reproductive health* 2013, **10**: 1.

80. Hounton S, Chapman G, Menten J, De Brouwere V, Ensor T, Sombié I, Meda N, Ronsmans C: **Accessibility and utilisation of delivery care within a Skilled Care Initiative in rural Burkina Faso.** *Tropical medicine & international health* 2008, **13**: 44-52.

81. Dugas M, Dubé E, Kouyaté B, Sanou A, Bibeau G: **Portrait of a lengthy vaccination trajectory in Burkina Faso: from cultural acceptance of vaccines to actual immunization.** *BMC international health and human rights* 2009, **9**: 1.

**Tableau 1 : Répartition des participants**

| **Participants** | **Nombre** | **Description** |
| --- | --- | --- |
| Travailleurs de la santé | 19 | 19 entrevues dans 10 CSPS |
| Marraines | 24 | 24 entrevues dans 23 villages participants |
| Mères d’enfants âgés de 12 à 24 mois | 33 | 33 entrevues dans 10 CSPS |
| Femmes enceintes | 19 | 19 entrevues dans 19 CSPS |
| Femmes en âge de procréer | 92 | 92 femmes dans dix entrevues collectives |
| **Nombre total de participants** | **187** |  |

**Encadré 1 : Exemples de questions, femmes enceintes**

1. **Accès et pratiques en matière de santé**
2. Quand avez-vous utilisé les services d’un CSPS ? Qu’en avez-vous pensé ? Pourriez-vous nous parler de la capacité des travailleurs de la santé à diagnostiquer et à traiter les maladies ?
3. Quels sont les principaux obstacles à l’accès aux soins de santé ? À quelle distance de chez vous le CSPS se trouve-t-il ? Quel est le coût du traitement ou de l’hospitalisation ?
4. Qui prend les décisions relatives à la grossesse dans votre ménage ? Quel est le rôle de votre conjoint ? Quel est le rôle de votre belle-mère ?
5. Quel est le principal traitement que vous chercherez à obtenir en cas de maladie ?
6. Quelle importance les soins et les rendez-vous prénataux ont-ils pour vous ? Pourquoi ?
7. À quel point la grossesse est-elle confidentielle ? Comment et quand d’autres personnes l’apprennent-elles ?
8. **Circulation de l’information sur la santé**
9. Comment l’information sur la santé circule-t-elle au sein de votre communauté ?
10. Comment êtes-vous informé des campagnes de vaccination à venir, par exemple ?
11. Comment faites-vous pour savoir quand vos prochains rendez-vous médicaux auront lieu ?
12. Trouvez-vous que cette façon de faire fonctionne bien ? Que pourrait-on améliorer?
13. **Perception et expérience des téléphones cellulaires**
14. Que pensez-vous des téléphones cellulaires ?
15. En avez-vous déjà utilisé ? À quelle fin ?
16. Avez-vous eu accès à un téléphone cellulaire depuis ?
17. Qui supervise l’utilisation du téléphone cellulaire à la maison ? Qui garde le téléphone ?
18. **Téléphone cellulaire et soins de santé**
19. Selon vous, comment pourrait-on se servir de téléphones cellulaires pour améliorer les soins de santé ?
20. Les travailleurs de la santé du CSPS savent-ils comment vous joindre, s’il y a lieu ?
21. **Impression générale du projet MOS@N**
22. Que pensez-vous de ce projet ? À quel point peut-il être utile ?
23. Que pensez-vous d’être suivie par des marraines ?
24. Selon vous, quels sont les défis ou les obstacles relatifs à la réalisation d’un tel projet ?

**Encadré 2 : Exemples de questions, travailleurs de la santé**

1. **Satisfaction globale à l’égard des services de santé**
2. Pouvez-vous décrire votre rôle et vos obligations principaux au CSPS ?
3. À quel point les soins de santé sont-ils accessibles à la population locale ?
4. Quels sont les principaux défis à relever pour améliorer l’accès aux soins de santé ?
5. Quels sont les principaux obstacles à la prestation de services de santé de qualité au CSPS ?
6. À quel point les soins et les rendez-vous prénataux sont-ils importants pour la population locale ?
7. **Circulation de l’information sur la santé**
8. Comment l’information sur la santé circule-t-elle au sein des communautés ?
9. À quel point cette forme de communication est-elle efficace ? En êtes-vous satisfait ?
10. Comment gérez-vous l’information sur la santé au CSPS ?
11. Comment surveillez-vous l’interruption du suivi ? Comment communiquez-vous avec vos patientes ?
12. **Santé mobile : technologies de l’information et de la communication et soins de santé**
13. À quelles fins utilisez-vous des téléphones cellulaires dans les activités courantes du CSPS ?
14. Quels sont les principaux avantages et inconvénients de l’utilisation de téléphones cellulaires ?
15. À quelles fins utilisez-vous des ordinateurs dans les activités courantes du CSPS ?
16. Avez-vous déjà utilisé un ordinateur ?
17. Quels pourraient être les avantages et les inconvénients d’utiliser des ordinateurs au CSPS ?
18. **Impression générale du projet MOS@N**
19. Quelle est votre impression générale du projet MOS@N ?
20. Que pensez-vous de travailler avec des marraines ?
21. Quels seront, selon vous, les principaux avantages du projet MOS@N ?
22. Vous attendez-vous à ce que le projet entraîne une augmentation ou une diminution de votre charge de travail ?
23. À quel point le projet MOS@N s’intègre-t-il bien à vos activités courantes ?
24. Quels seront, selon vous, les principaux obstacles à la réussite de la mise en oeuvre du projet ?
