# Supplementary material for: People and research: improved health systems for West Africans, by West Africans - report on special supplement
Source: BMC Proc. 2019 Feb 7;13(Suppl 1):1. doi: 10.1186/s12919-019-0162-0 (PMC6366023; doi:10.1186/s12919-019-0162-0)
Supplement: Supplementary file 6 — Jeter un pont entre le programme sur la santé des mères, des nouveau-nés et des enfants (SMNE) et les recherches sur les systèmes de santé: facteurs propices et défavorables liés aux systèmes de santé pour améliorer les incidences de la SMNE en Afrique de l’Ouest, Agyepong, I., Kwamie, A., Frimpong, E., Defor, S., Ibrahim, A., Aryeetey, G., Lokossou, V., Sombie, I. [file 12919_2019_162_MOESM6_ESM.docx]

***Jeter un pont entre le programme sur la santé des mères, des nouveau-nés et des enfants (SMNE) et les recherches sur les systèmes de santé : facteurs propices et défavorables liés aux systèmes de santé pour améliorer les incidences de la SMNE en Afrique de l’Ouest***

**Irene Akua Agyepong (1)*, Aku Kwamie (1), Edith Frimpong (1), Selina Defor (1), Abdallah Ibrahim (2), Genevieve C. Aryeetey (2), Virgil Lokossou (3), Issiaka Sombie (3)**

*Auteur-ressource

1 Services de santé du Ghana, Division de la recherche-développement, C. P. MB-190, Grande région d’Accra, Ghana

2 École de la santé publique, Université du Ghana, C. P. LG13, Legon, Accra, Ghana

3 Organisation ouest-africaine de la santé, Bobo-Dioulasso, 01BP 153 Bobo-Dioulasso 01 Burkina Faso

Adresses électroniques :

IAA : [iagyepong@hotmail.com](mailto:iagyepong@hotmail.com)

AK : [agkwamie@hotmail.com](mailto:agkwamie@hotmail.com)

EF : [amakobi18@yahoo.com](mailto:amakobi18@yahoo.com)

SD : [sellydel@yahoo.com](mailto:sellydel@yahoo.com)

AI : [ibdallah4@gmail.com](mailto:ibdallah4@gmail.com)

GCA : [okailey.aryeetey@gmail.com](mailto:okailey.aryeetey@gmail.com)

VL : [vlokossou@wahooas.org](mailto:vlokossou@wahooas.org)

IS : [isombie@wahooas.org](mailto:isombie@wahooas.org)

**Résumé**

**Contexte :** En dépit d’améliorations réalisées au fil du temps, l’Afrique de l’Ouest accuse un retard à l’échelle mondiale de même que par rapport aux moyennes enregistrées en Afrique subsaharienne en ce qui concerne les incidences du programme sur la santé des mères, des nouveau-nés et des enfants (SMNE). Cette situation existe malgré la disponibilité d’un corpus de connaissances de plus en plus important sur les interventions qui améliorent de telles incidences. Au-delà de la connaissance des interventions qui donnent de bons résultats, il nous faut des perspectives sur d’autres facteurs qui facilitent ou freinent l’amélioration des incidences de la SMNE. La présente étude visait à examiner les facteurs liés aux systèmes de santé qui sont propices ou défavorables à la mise en oeuvre de politiques et de programmes et aux incidences en matière de SMNE en Afrique de l'Ouest, ainsi qu’à déterminer comment et pourquoi ils donnent de bons résultats en contexte.

# Méthodologies : Nous avons effectué une étude de cas multipays à méthodes mixtes mettant principalement l’accent, mais pas exclusivement, sur les six pays d’Afrique de l'Ouest (Burkina Faso, Bénin, Mali, Sénégal, Nigéria et Ghana) qui participent à l’initiative Innovation pour la santé des mères et des enfants d’Afrique (ISMEA). La collecte de données comprenait un examen non exhaustif de la littérature publiée et de la littérature grise, ainsi que 48 entrevues avec des informateurs clés (IC). Nous avons validé nos constatations et nos conclusions lors de deux réunions multilatérales distinctes organisées par l’Organisation ouest-africaine de la santé (OOAS). Pour orienter la collecte et l’analyse de données, nous avons élaboré un cadre théorique unique du lien entre les systèmes de santé et la SMNE, dans lequel nous avons conceptualisé les systèmes de santé à titre de fondations, de piliers et de toiture d’un abri pour la SMNE, et le contexte à titre de sol sur lequel les fondations sont érigées.

# Résultats

Une multitude de politiques et d’interventions liées à la SMNE ont fait l’objet de projets pilotes, de recherches ou ont été mises en oeuvre à grande échelle dans la sous-région. Presque toutes ont fait face à de nombreux facteurs propices et défavorables à une mise en oeuvre efficace en interaction avec les systèmes de santé, ainsi qu’à des défis contextuels. Le contexte a agi en exerçant ses effets sur les facteurs des systèmes de santé, ainsi que sur les déterminants sociaux de la santé.

**Conclusions**: Pour accélérer et soutenir les améliorations des incidences en matière de SMNE en Afrique de l'Ouest, il faut adopter une approche intégrée de la recherche et de la pratique pour aborder simultanément les systèmes de santé et les facteurs contextuels, de concert avec les interventions liées à la prestation de services pour la SMNE. Ceci nécessite des approches axées sur la participation de nombreux intervenants, à divers niveaux et dans tous les secteurs qui jettent un pont entre les limites actuelles des collectivités géographiques, linguistiques, de la recherche et de la pratique en Afrique de l'Ouest, et contribuent effectivement à unir les efforts des acteurs intéressés par le renforcement des systèmes de santé et de ceux qui s’intéressent à l’amélioration des incidences en matière de SMNE.

**Mots/expressions clés** : Santé des mères, des nouveau-nés et des enfants, systèmes de santé, contexte, limites, complexité, CÉDÉAO

# Contexte

Les pays de la Communauté économique des États de l’Afrique de l'Ouest (CÉDÉAO) constituent la majeure partie de la sous-région de l’Afrique de l'Ouest et tous ensemble affichent une population estimée à près de 350 millions d’habitants. La CÉDÉAO comprend 15 pays, lesquels sont tous classés dans la catégorie des pays à revenu faible et intermédiaire. La sous-région accueille une immense diversité de peuples, de cultures, de langues et de religions. Superposé à la diversité ethnique, religieuse et linguistique traditionnelle, et contribuant à en accroître la complexité, on retrouve l’héritage colonial de la fragmentation selon la langue officielle en pays anglophone, francophone et lusophone.

Dans le passé, la stabilité politique sociale et économique dans la sous-région a été gâchée par des coups d’État, des conflits civils internes et des programmes d’ajustement structurel. Plus récemment, l’instabilité a inclus des menaces terroristes comme Boko Haram et Al-Quaida au Maghreb, l’insécurité alimentaire due aux changements climatiques, l’utilisation accrue de certains pays à titre de voie de transit pour le trafic de drogue et l’épidémie la plus massive de la maladie à virus Ebola jamais enregistrée. Elle a franchi les frontières de trois pays, a entraîné des cas importés dans trois autres pays de la sous-région et même au-delà. Malgré la myriade de défis qu’elle doit affronter, la CÉDÉAO est aussi une sous-région qui se démarque par ses réalisations et son potentiel. Sa population est jeune et nombre de ses économies sont en croissance. Une démocratie pluripartite et la stabilité politique sont en passe de caractériser lentement l’histoire du présent.

Les taux actuels de mortalité maternelle et des enfants de moins de cinq ans au sein de la CÉDÉAO sont les résultats d’un recul progressif lent par rapport aux niveaux des décennies précédentes, et s’engagent clairement dans la bonne direction. Toutefois, en termes relatifs, ces taux accusent un retard par rapport aux moyennes mondiales et en Afrique subsaharienne [^[[1]](#endnote-1)^]. Les taux de mortalité néonatale ont été estimés élevés, par exemple avec 182 décès par 1 000 naissances au Sierra Leone, 128 décès par 1 000 naissances au Mali et 124 décès par 1 000 naissances au Nigéria. Les estimations de taux de mortalité maternelle sont d’importance similaire en Côte D’Ivoire (720/100 000), au Niger (630/100 000) et en Gambie (430/100 000) [^[[2]](#endnote-2)^]. De plus, à l’intérieur de ces tendances mondiales moyennes en matière de mortalité pour l’Afrique de l'Ouest on constate de larges variations à l’échelle nationale et sous-nationale [^[[3]](#endnote-3),^ ^[[4]](#endnote-4),^ ^[[5]](#endnote-5),^ ^[[6]](#endnote-6),^ ^[[7]](#endnote-7)^]. Ce qui soulève la question essentielle suivante, pourquoi les améliorations au taux de mortalité des mères, des nouveau-nés et des enfants (SMNE) en Afrique de l'Ouest accusent-elles un retard par rapport aux moyennes en Afrique subsaharienne et à l’échelle mondiale ?

Il existe de plus en plus un grand nombre de travaux de recherche sur les interventions efficaces pour obtenir l’amélioration de la SMNE [^[[8]](#endnote-8)^]. S’il était suffisant de posséder des connaissances au sujet des interventions efficaces éprouvées, l’Afrique de l'Ouest ne ferait pas face à ses difficultés présentes et persistantes. De toute évidence, d’autres facteurs nuisent à la capacité de mettre en oeuvre ces interventions à grande échelle, de soutenir la mise en oeuvre et d’accélérer les améliorations des incidences en matière de SMNE.

L’une de ces grappes de facteurs qui justifient une étude plus approfondie est le lien entre les systèmes de santé, les interventions et les incidences en matière de SMNE. La faiblesse des systèmes de santé compte parmi plusieurs facteurs auxquels on impute la lenteur des progrès réalisés dans l’atteinte des objectifs liés à la santé en Afrique subsaharienne [^[[9]](#endnote-9),^ ^[[10]](#endnote-10)^]. Les interventions visant à améliorer les incidences en SMNE sont mises en oeuvre à l’intérieur des systèmes de santé. Quels facteurs à l’intérieur de ces systèmes sont propices ou défavorables à la mise en oeuvre efficace à grande échelle de ces interventions ? Alors que les systèmes de santé existent à l’intérieur d’un contexte mondial, national et sous-national, est-ce que ce contexte influence aussi la mise en oeuvre efficace à grande échelle des interventions en SMNE ?

Sheikh et coll. [^[[11]](#endnote-11)^] soulignent la nature quelque peu fragmentée de la santé mondiale compte tenu des nombreux territoires de pratique et cadres ainsi que des appels pour que la pratique en matière de santé mondiale inclue des efforts pour rapprocher ces frontières si on veut qu’elle devienne plus pertinente pour les collectivités qui sont visées à titre de bénéficiaires. Les champs de la recherche en SMNE et de la recherche sur les politiques et les systèmes de santé sont deux de ces territoires de la santé mondiale qui pourraient tirer profit d’un tel mouvement visant à *« franchir les frontières (…) pour établir des relations, des interactions et une interdépendance »* [^[[12]](#endnote-12)^] ou à consentir des efforts pour partager des connaissances.

La présente étude visait donc à examiner les facteurs liés aux systèmes de santé qui sont propices ou défavorables à la mise en oeuvre à grande échelle de politiques et de programmes en SMNE en vue de l’amélioration des incidences en matière de santé dans la CÉDÉAO. Elle cherchait aussi à déterminer comment et pourquoi ces facteurs fonctionnent ou sont susceptibles de fonctionner en contexte pour influencer les incidences en SMNE.

# Méthodologies

La conception de l’étude reposait sur une étude de cas à méthodes mixtes sur la SMNE et les systèmes de santé en Afrique de l'Ouest. La collecte de données comprenait notamment un examen non exhaustif de documents de la littérature publiée et grise, ainsi que des entrevues avec des informateurs clés. En Afrique de l'Ouest, nous nous sommes attachés plus particulièrement, mais non exclusivement aux six pays cibles de l’initiative ISMEA, c’est-à-dire le Bénin, le Burkina Faso, le Ghana, le Mali, le Nigéria et le Sénégal. Ces pays réunis représentent près des trois quarts de la population de l’Afrique de l'Ouest. Ils comprennent des pays anglophones (Nigéria et Ghana) ainsi que francophones (Bénin, Burkina Faso, Mali et Sénégal) et représentent la répartition géographique qui s’étend de la région côtière du sud jusqu’à la savane au nord de l’Afrique de l'Ouest.

L’examen de documents a consisté principalement à établir la portée des travaux de recherche existants, et nous avons scruté les recherches publiées et grises anglophones et francophones sur les systèmes de santé (RSS) et sur la SMNE ainsi que les documents portant sur les politiques, les programmes et la mise en oeuvre en Afrique de l'Ouest, de manière générale, et dans les six pays cibles, en particulier pendant la période de 1990 à 2015. Les bases de données que nous avons consultées en ligne sont PubMed, Scopus, ScoIndex, CAIRN.Info, CINHAL, Google Scholar, Africa Journals online, JSTOR et Embase, la Cochrane Library et l’EPPI-Centre. Nous avons aussi interrogé des informateurs clés en vue d’obtenir des liens vers de la littérature grise pertinente, comme des rapports annuels, des programmes de travail, des aide-mémoires et des examens du rendement. Les documents obtenus et examinés comprenaient notamment des publications évaluées par les pairs, des examens systématiques, des rapports nationaux, des projets de recherche, des documents et des rapports d’agences et d’organisations non gouvernementales (ONG).

Voici un aperçu des termes utilisés pour effectuer la recherche en ligne : santé des mères; soins prénataux; soins postnataux; santé des nouveau-nés OU santé néonatale; santé des enfants OU santé des enfants de moins de cinq ans; planification familiale; santé reproductive; avortement; mortalité maternelle; morbidité maternelle; mortalité néonatale; mortalité infantile ET ressources humaines OU infirmier* OU sage-femme* OU effectif du secteur de la santé; gouvernance, leadership, gestion; responsabilité; médicaments, technologies; produits; systèmes d’information sur la santé; prestation de service; financement OU exemption des frais OU assurance santé ET transport; ambulance, communauté; préparation à l’accouchement ET Bénin, Burkina Faso, Ghana, Mali, Nigéria, Sénégal, Afrique de l'Ouest ET pouvoir, confiance, processus décisionnel, politique, parties prenantes, politique, contexte.

Les entrevues avec des informateurs clés (EIC) visent à compléter ainsi qu’à combler les lacunes dans l’analyse documentaire. La sélection des répondants a été intentionnelle afin d’assurer la représentation des acteurs et des parties prenantes clés à l’échelle nationale et sous-nationale ainsi que des partenaires du développement qui participaient aux systèmes de santé et à la SMNE dans les six pays cibles. Nous disposions d’une liste de personnes-ressources fournie par l’Organisation ouest-africaine de la santé (OOAS) d’acteurs et de parties prenantes susceptibles de satisfaire à ces critères d’inclusion dans les six pays cibles. L’OOAS entretient des liens avec les secteurs de la santé et les ministères de la santé des 15 pays de la CÉDÉAO et se trouvait donc dans une position idéale pour nous aider à repérer les principaux répondants dans les pays. La liste définitive des personnes à interroger a été commodément établie à partir de celles qui avaient pu être jointes lors de nos appels téléphoniques et courriels exploratoires, et qui se disaient disponibles et disposées à être interrogées en personne, par courriel ou par Skype. Nous mettions fin aux entrevues lorsque ces dernières ne débouchaient sur aucune question ou perspective. Nous avons mené au total 48 entrevues auprès d’informateurs clés.

Toutes les entrevues se sont déroulées entre le 21 décembre 2015 et le 2 février 2016 et ont été menées par les mêmes deux membres de l’équipe (un anglophone et une personne bilingue). Les informateurs clés occupaient des postes tels que directeur national, directeur général, conseiller national, directeur divisionnaire ou sous-divisionnaire, conseiller principal, spécialiste et expert-conseil. On trouvera au tableau 1 plus de renseignements sur les répondants.

Toutes les entrevues ont été menées après avoir obtenu le consentement libre et éclairé. Lorsque l’on avait obtenu la permission, les entrevues étaient enregistrées et transcrites. Lorsque les informateurs clés refusaient d’être enregistrés, nous utilisions les notes prises par l’intervieweur.

Une réunion nationale de l’OOAS au Ghana, en novembre 2015 et une réunion sous-régionale tenue à Dakar, en février 2016 ont été utilisées à titre d’occasions de présenter les constatations et les conclusions préliminaires en vue d’obtenir la validation, les critiques et les commentaires de parties prenantes à divers niveaux du secteur de la santé dans les pays de la sous-région qui participaient à ces réunions.

**Tableau 1 Un résumé des informateurs**

| Sexe | n % | % |
| --- | --- | --- |
| Femme | 23 | 48% |
| Homme | 25 | 52% |
| Agence de travail |  |  |
| Partenaire de développement (par exemple USAID, UNFPA, WHO, UNICEF) | 15 | 31% |
| Ministère de la santé / agence de santé publique (par exemple Ghana Health Service) | 17 | 35% |
| ONG (national, par exemple Federation of Muslum Women, Members of Coalition of NGO in Health, et international, par exemple Family Care, Health Keepers, Marie Stoppes) | 9 | 19% |
| Représentant d’une association professionnelle | 3 | 6% |
| Spécialiste Clinique (par exemple obstétricien, gynécologue, pédiatre) | 3 | 6% |
| Chercheure universitaire (un répondant exerce une double fonction dans le ministère de santé et à l’université | 2 | 4% |
| Pays |  |  |
| Ghana | 17 | 35% |
| Nigeria | 5 | 10% |
| Burkina | 8 | 17% |
| Benin | 6 | 13% |
| Mali | 6 | 13% |
| Senegal | 6 | 13% |

# Cadre conceptuel pour l’étude, sujets à explorer et analyse de données

Les sujets à explorer ont été tirés d’un cadre conceptuel unique élaboré à partir de la manière dont les systèmes de santé sont liés aux interventions et aux incidences de la SMNE. Nous avons élaboré ce cadre à la suite de l’examen d’une série de cadres existants proposés pour faire la description et l’analyse des systèmes de santé [^[[13]](#endnote-13)^ ^,^ ^[[14]](#endnote-14) ,^ ^[[15]](#endnote-15) ,^^[[16]](#endnote-16) ,^^[[17]](#endnote-17) ,^^[[18]](#endnote-18) ,^^[[19]](#endnote-19)^ ^,^^[[20]](#endnote-20)^], ainsi que d’une séance de remue-méninges. Dans tous ces cadres, la « santé » des individus au cours de leur vie, souvent mesurée en fonction des issues fatales et non fatales, était un objectif intrinsèque et l’objectif déterminant des systèmes de santé.

Au-delà de cet objectif déterminant, deux autres objectifs se dégagent fréquemment à titre d’objectifs intrinsèques dans la majorité, sinon la totalité, des cadres recensés dans la documentation : ce sont la réceptivité et l’équité dans les contributions financières ou la protection contre les risques financiers. La réceptivité fait référence aux attentes légitimes des personnes quant aux aspects non liés à la santé des systèmes de santé, comme la manière dont les gens sont traités (dignité, respect, autonomie, choix, confidentialité) et l’environnement dans lequel ils sont soignés lorsqu’ils cherchent à obtenir des soins de santé [^[[21]](#endnote-21)^ ^,^^[[22]](#endnote-22)^]. La réceptivité est un objectif social et un objectif qui ne se limite pas nécessairement aux systèmes de santé. Le système de santé, à l’instar d’autres systèmes sociaux comme la justice et l’éducation, est censé, au-delà de son objectif fondamental, remplir aussi cet objectif social commun. L’équité dans les contributions financières ou la protection contre les risques financiers prennent de plus en plus d’importance compte tenu du mouvement vers la couverture santé universelle. Elle fait référence au fait que l’on veille à ce que les frais payés pour les soins de santé ne soient pas catastrophiques et n’exposent pas les gens à un risque excessif.

Essentiels pour déterminer la capacité des systèmes de santé d’assurer l’atteinte de leurs objectifs intrinsèques, ils sont ce que les cadres de travail des systèmes de santé décrivent fréquemment comme les éléments clés ou le matériel. Ils comprennent notamment les ressources (humaines, infrastructure, équipement, outils et fournitures), les modalités de financement, les systèmes d’information sur la santé, la gouvernance ou l’intendance, les médicaments et la technologie.

De plus en plus reconnus comme des facteurs déterminants du fonctionnement des systèmes de santé sont les gens qui y évoluent [^[[23]](#endnote-23)^], leur pouvoir et la manière dont ils choisissent de l’exercer; ainsi que les processus qu’ils mettent en place et qu’ils utilisent pour administrer le système de santé. Cet aspect des systèmes de santé est parfois appelé le logiciel. Les gens sont au coeur des systèmes de santé, et ils en sont le moteur, un peu comme le font les logiciels dans les systèmes informatiques. Tout dépendant du système d’exploitation et du logiciel utilisés, le même matériel informatique peut effectuer diverses fonctions qui produisent des résultats différents. Dans les systèmes de santé, selon les gens qui s’y trouvent, le pouvoir qu’ils exercent, leurs intérêts, réseaux et relations, etc., on constatera des variations dans leur rendement, et ce, même avec le même matériel ou les mêmes éléments clés.

Puisant dans ces cadres existants, les synthétisant et leur apportant une réflexion critique ainsi que sur la manière dont ils sont liés aux interventions et aux incidences de la SMNE, nous avons conceptualisé les systèmes de santé comme s’il s’agissait d’une maison qui met à l’abri la santé de la population, notamment des mères, des nouveau-nés et des enfants (figure 1).


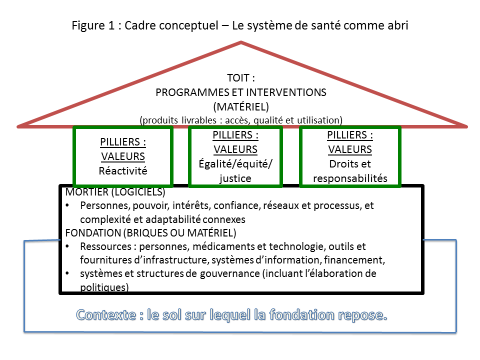


## Pierres d’assise : éléments clés des systèmes de santé classiques (matériel)

À l’intérieur de ce cadre, ce que l’on appelle communément les composantes de base du système de santé sont conceptualisées comme étant les pierres d’assise de la maison. La force et la capacité de ces pierres d’assise de soutenir la structure et de résister couramment aux chocs, ainsi que lors de crises graves (résilience), contribuent à la solidité du système de santé.

## Mortier : personnes, processus, pouvoir (logiciel)

Ce que l’on qualifie de logiciel dans certains modèles de systèmes de santé (personnes, processus, pouvoir, etc.), nous le considérons comme le mortier qui détermine dans quelle mesure les pierres d’assise sont solidement jointoyées. C’est ainsi qu’elles contribuent à la solidité, la stabilité et la résilience du système de santé. Pouvoir et influence au sein des organisations sont *« la capacité d’entraîner (ou d’affecter) les résultats organisationnels »; « de faire en sorte que les choses souhaitables se réalisent, de produire des incidences – les actions et les décisions qui les précèdent* ». [^[[24]](#endnote-24)^] Les gens en tant qu’acteurs et parties prenantes sont les responsables des décisions et de la mise en oeuvre. Qui possède le pouvoir ou l’influence nécessaire pour faire quelque chose, ainsi que la manière dont il utilise le pouvoir ou les manifestations de ce pouvoir revêtent de l’importance. Ainsi, qui exerce son pouvoir sur qui et sur quoi, qui exerce son pouvoir avec qui ou par l’entremise de qui, sont tous des facteurs essentiels et importants qui peuvent être défavorables ou propices à la mise en oeuvre et aux incidences des interventions et des programmes en SMNE. Réseaux, renseignement et information, transparence, confiance et respect, conflit, intérêts et perspectives des parties prenantes font tous partie des interactions humaines et des processus qui constituent le mortier.

## Piliers : valeurs

Chaque système de santé possède des valeurs sous-jacentes explicites et implicites qui influencent les décisions et les actions. À titre d’exemple, la conception d’un système de santé comme les modalités de financement et la répartition des ressources humaines dans un système où la santé est reconnue de façon explicite et implicite comme un droit fondamental de la personne, et où l’équité revêt une grande valeur peut s’avérer très différente de celle d’un système de santé où ce n’est pas le cas. Nous avons déterminé que les valeurs sous-jacentes des systèmes de santé sont les piliers de la maison ou de l’abri.

Nous avons déterminé que les trois principaux piliers ou les trois principales valeurs sous-jacentes des systèmes de santé sont la réceptivité, l’équité (impartialité et justice) ainsi que les droits et les responsabilités. Même si, habituellement, dans les cadres de travail des systèmes de santé, la réceptivité est décrite comme un résultat intrinsèque, dans le cas présent, la réceptivité est une valeur au même titre qu’un résultat intrinsèque. Nous avons choisi de lui accorder une place prépondérante en tant que valeur dans le présent cadre de travail étant donné que nous pensons que les valeurs stimulent et façonnent les incidences des systèmes de santé. Le traitement des personnes – qu’il s’agisse de clients (clients externes) ou de travailleurs de la santé (clients internes) – avec dignité, respect, confidentialité, autonomie, en leur accordant une attention prompte, ainsi que les réseaux de soutien social et le choix [^[[25]](#endnote-25)^] sont un résultat des systèmes de santé. Toutefois, ils sont également une manifestation des valeurs accordées aux droits de la personne dans un système donné. Ils reflètent donc les valeurs tout autant que les résultats. L’équité ou la justice et l’impartialité sont des concepts comportant des significations comparables, et nous les utilisons de façon interchangeable. Ils font référence au fait de se montrer impartial, d’agir avec intégrité, moralité, et d’attribuer ce qui est dû dans la répartition des ressources, des gains, des pertes, des récompenses et des punitions dans les sociétés et dans les relations sociales. L’équité, comme la réceptivité est une valeur et un processus, de même qu’un résultat [^[[26]](#endnote-26)^]. Qui a le droit de faire quoi, comment et pourquoi ainsi que le concept connexe de qui est responsable de quoi, comment et pourquoi sont des valeurs à l’intérieur des systèmes de santé qui influenceront la manière dont les personnes sont traitées, de même que la responsabilité.

## Toiture : interventions et programmes

Nous avons conceptualisé les interventions et les programmes à titre de toiture de l’abri. Les pierres d’assise et les piliers soutiennent cette toiture pour servir la population. Ils influencent la faisabilité, l’efficacité et le résultat de la mise en oeuvre de ces interventions. Cela comprend les interventions destinées à améliorer la santé des mères, des nouveau-nés, des nourrissons, des enfants et des adolescents. Si on soutient les interventions mais que l’on néglige le système de santé et ses valeurs, cela revient à élever une structure dont les assises et les piliers sont faibles pour soutenir la toiture. Elle manquera de résilience et sera susceptible de s’effondrer si elle est exposée au stress.

## Le sol sur lequel les systèmes de santé sont construits : le contexte

Par contexte, on entend « *l’ensemble de circonstances ou de faits qui entourent un événement, une situation, etc*. » [^[[27]](#endnote-27)^] En tant que cadre à l’intérieur duquel les systèmes de santé existent et les événements se produisent, le contexte doit être pris en considération pour bien comprendre la situation, le phénomène ou les événements qui nous intéressent. Si on conserve l’image de notre cadre de travail qui représente le système de santé comme un abri, le contexte peut être vu comme le sol sur lequel les fondations sont montées.

Les types de contexte, ou les circonstances dans lesquelles les systèmes de santé sont élaborés et fonctionnent comportent plusieurs couches, de l’échelle mondiale, à l’échelle nationale jusqu’à l’échelle sous-nationale. Ils comprennent notamment les tendances démographiques, les facteurs socioculturels, y compris les valeurs et les normes de la société, les conditions et les tendances macroéconomiques, l’histoire, la politique et l’idéologie [^[[28]](#endnote-28),^ ^[[29]](#endnote-29)^]. Le contexte joue un rôle essentiel dans la stabilité et la résilience des systèmes de santé. Comme la parabole de la maison construite sur le sable et de celle qui est construite sur le roc, les systèmes de santé se trouvant dans des contextes faibles peuvent s’apparenter à des maisons construites sur le sable qui sont susceptibles de s’effondrer en cas d’exposition à un stress. Un exemple récent en Afrique de l'Ouest est l’effet dévastateur de l’épidémie de la maladie à virus Ebola en Guinée, au Libéria et en Sierra Leone; tous ces pays étaient des États fragiles ou se relevant d’un conflit, et marqués par des histoires de sous-développement économique et humain [^[[30]](#endnote-30)^]. Les faiblesses des systèmes de santé que la maladie à virus Ebola a exposées étaient en partie dues à ces graves difficultés contextuelles.

Nous avons analysé nos données selon les thèmes ou les catégories de ce cadre conceptuel. Toute l’analyse de données a été effectuée manuellement, et nous avons cherché les points communs, ainsi que les contrastes et les contradictions. Nous avons procédé au recoupement de données issues de l’analyse documentaire et de celles fournies par les informateurs clés dans le cadre d’une vérification de la qualité et de la validité interne.

## Limitations du cadre

Notre cadre de travail est un outil utile pour faciliter l’organisation de l’enquête et de l’analyse, mais comme tous les cadres, il comporte ses limites. En pratique, il est presque impossible de tracer une délimitation précise entre les processus et les structures, les institutions et les acteurs qui les amorcent, les maintiennent et y mettent fin. Les processus au sein des systèmes de santé sont activés par les personnes et l’usage, et ils sont influencés par les composantes de base ou le matériel et le contexte. Les processus se déroulent à l’intérieur des structures et des institutions et entre celles-ci.

# Résultats et discussion

La section sur les résultats présente et, le cas échéant, compare et met en opposition simultanément les résultats de l’analyse documentaire et ceux des entrevues avec les informateurs clés. Nous présentons d’abord nos constatations selon une forme plus descriptive à l’aide des catégories du cadre de travail. L’interdépendance entre les catégories signifie toutefois que pour éviter la répétition, il n’est pas possible de tracer des lignes très nettes. Cette interdépendance offre l’explication la plus complète du « comment » et du « pourquoi » les systèmes de santé et les facteurs contextuels entrent en jeu pour influer sur la mise en oeuvre et les incidences des politiques et des programmes en SMNE. À la fin, nous décrivons donc un simple cadre de travail pour cette interdépendance établie à partir de nos constatations et nous l’illustrons à l’aide d’exemples tirés des données.

## Programmes et interventions (prestation de service) : la toiture

Au moyen de l’analyse documentaire et des entrevues menées auprès d’informateurs clés, nous avons repéré plusieurs interventions faisant l’objet de projet pilote, de travaux de recherche ou d’une mise en oeuvre à grande échelle dans plusieurs pays en vue d’améliorer les incidences en SMNE dans la CÉDÉAO (encadré 1). La diversité de la profondeur, de l’ampleur et des méthodes de mise en oeuvre était telle que pour les cartographier en détail, il aurait fallu mener une étude séparée.

Encadré 1 – Interventions en SMNE en Afrique de l'Ouest

1. Audits de la mortalité
   1. Audit communautaire (verbal)
   2. Examens menés dans les établissements
   3. Enquête confidentielle sur les décès maternels
2. Audits sur les incidents évités de justesse
3. Audit clinique fondé sur des critères
4. Aiguillage
5. Interventions en santé au moyen d’appareils électroniques/mobiles
6. Interventions en matière de financement
   1. Interventions visant l’introduction, la réduction ou l’élimination des frais d’utilisation remboursables
      1. Exemptions, par exemple césarienne gratuite, soins anténataux, accouchement, etc.
      2. Régime d’assurance-maladie communautaire (RAMC)
      3. Régime d’assurance santé national (RASN)
   2. Transferts d’espèces conditionnels
   3. Financement fondé sur le rendement
7. Déplacement des tâches
8. Soins préventifs
   1. Soins anténataux
   2. Soins postnataux
   3. Planification familiale

L’accès à ces interventions et leur utilisation se sont avérés des facteurs propices (accès adéquat, utilisation) tout autant que défavorables (accès inadéquat, non-utilisation) à partir des constatations de l’analyse documentaire. L’accès aux interventions en SMNE a été influencé par des facteurs géographiques tels que la situation et la distance des établissements par rapport aux ménages et aux collectivités, comme on a pu le constater dans plusieurs études réalisées au Ghana et au Burkina Faso [^[[31]](#endnote-31),^ ^[[32]](#endnote-32),^ ^[[33]](#endnote-33),^ ^[[34]](#endnote-34),^ ^[[35]](#endnote-35)^]. Parfois, même lorsque le service était accessible sur le plan géographique, le contenu du service et l’absence de types et de niveaux particuliers de service se sont avérés défavorables [^[[36]](#endnote-36)^ ^,^ ^[[37]](#endnote-37)^].

Le contenu du service a aussi exercé une influence sur la qualité des soins en raison de son effet sur les grappes de facteurs liées à d’autres facteurs du système de santé comme la quantité et la qualité des ressources humaines, l’infrastructure, les outils et les fournitures, ainsi que les difficultés sur le plan des procédures et des capacités. Ces derniers ont influé sur les soins offerts pendant le transport dû à un aiguillage, les retards dans les soins à l’arrivée et les procédures de prestation de service. Ils ont aussi eu une influence sur l’expression de valeurs comme la réceptivité.

*« La logistique suivie, et puis aussi l’infrastructure, l’électricité, l’eau, le respect de la vie privée, tous ces facteurs sont inadéquats »* [Directeur de la santé du secteur public, Ghana]

*L’aménagement des établissements de santé rend encore plus difficile de garantir la confidentialité. Les lieux utilisés pour donner les consultations ne sont pas privés, il y a donc un va-et-vient de personnes pendant les consultations, du début de l’interrogation jusqu’au diagnostic et à la prescription du traitement. C’est ce qui explique pourquoi la majorité des patients préfèrent se rendre dans des établissements privés où l’on peut assurer un minimum d’anonymat et de confidentialité.* [Directeur à l’échelle nationale, ministère de la Santé, Mali]

La solution de ces problèmes a été propice à la prestation de services en SMNE. Par exemple, on a observé en milieu rural, au Burkina Faso, que la fourniture d’une infrastructure, d’équipement, d’outils et de fournitures avait amélioré l’accès et l’utilisation des services en SMNE [^[[38]](#endnote-38)^]. Au Nigéria, assurer une affectation adéquate des ressources pour combler les lacunes repérées dans les secteurs de la santé et des services sociaux, s’est révélé propice à l’établissement d’examens des décès maternels [^[[39]](#endnote-39)^].

Les facteurs liés au système de santé qui étaient propices et ceux qui étaient défavorables pour l’accès et l’utilisation des interventions étaient parfois étroitement liés à d’autres secteurs et à des facteurs contextuels. Par exemple, mis à part la disponibilité de l’ambulance [^[[40]](#endnote-40)^], le transport en cas d’aiguillage était aussi facilité ou freiné par la qualité des routes et des systèmes de communication, comme les appareils mobiles, qui permettaient d’établir la communication entre les travailleurs de la santé qui effectuaient différentes tâches dans le même établissement ou dans plusieurs établissements [^[[41]](#endnote-41)^]. L’examen systématique de tous les audits sur la mortalité publiés dans les pays à revenu faible et à revenu intermédiaire (1965-2011) pour déterminer les facteurs évitables les plus fréquents dans les décès liés à l’accouchement a révélé que dans 7 % des cas, les facteurs liés au transport, comme l’absence de moyen de transport ou le retard du moyen transport, ou le moyen de transport déficient entre les établissements et de la maison jusqu’à l’établissement avaient contribué au décès [^[[42]](#endnote-42)^].

Les facteurs défavorables mentionnés par les informateurs clés étaient comparables à ceux qui sont ressortis de l’analyse documentaire. Ils comprenaient notamment la qualité des soins, les politiques qui influent sur la prestation de service et qui ne se traduisent pas adéquatement dans le travail en situation réelle, l’emplacement géographique, la distribution des services y compris des inégalités dans la distribution, et l’absence d’arrangements fonctionnels en matière de soins de santé primaires, y compris les systèmes d’aiguillage et les marches à suivre.

*« (…) les gens sont privés de services, il faut donc prévoir une certaine forme de système de soins de santé primaires afin que les différents niveaux des soins de santé soient interdépendants, (…) il faudrait mettre en place une marche à suivre en cas d’aiguillage vers différents niveaux de système de santé, (...) ou un système de transport bon marché lorsqu’il est nécessaire de procéder (…) à l’aiguillage des femmes qui connaissent des complications soit pendant la grossesse ou pendant l’accouchement(...) »* [Consultante en sage-femmerie, Ghana]

*« (…) les gens n’ont pas accès à des moyens de transport, qu’ils soient gratuits ou bon marché, lorsqu’il est nécessaire d’aiguiller les femmes en cas de complications soit pendant la grossesse ou l’accouchement (…) »* [Partenaire du développement multilatéral, Ghana]

Il s’avère parfois difficile de faire en sorte que les programmes et les interventions soient en phase avec les connaissances actuelles.

*« Il faut toujours du temps pour que les services émergents en lien avec la SMNE soient introduits parce que les protocoles et les cadres politiques nationaux ne sont pas mis à jour régulièrement de manière à refléter les constatations de recherche actuelles et les normes de l’OMS ».* [Partenaire du développement bilatéral, Sénégal]

De même, concrétiser les interventions et les mettre en oeuvre sur le terrain plutôt que de simplement les inscrire au programme d’action ou même de les faire passer du programme d’action à la formulation s’est parfois avéré un défi.

*« Nous devons faire beaucoup plus en ce qui concerne la traduction des politiques en gestes concrets dans les points de prestation de services (…) »* [Directeur national de la santé, Nigéria]

*« Je suis impliqué personnellement dans un système de surveillance de la mortalité maternelle qui est censé produire des données sur les décès des femmes. Ces données sont supposées être analysées et réintroduites dans un système d’information national afin d’améliorer le système de santé et de renseigner la programmation des interventions pour améliorer le service destiné aux mères et aux enfants, mais cette activité n’a jamais vu le jour, et ce, en dépit de toutes les formations qui ont été dispensées dans pratiquement tous les districts, sans parler de toutes les lois qui ont été adoptées à cet effet. »* [Directeur d’un département universitaire, Bénin]

Les facteurs propices repérés étaient généralement à l’opposé des facteurs défavorables, comme la disponibilité de l’infrastructure, ou d’un service d’ambulance.

« (…) *nous avons plus de 35 000 établissements de santé; des établissements de santé primaire, secondaire et tertiaire, et ils sont dispersés aux quatre coins du pays. (…) le fait qu’ils soient disponibles, et qu’un grand pourcentage d’entre eux assurent la prestation de services est un facteur propice, et nous devons travailler à le renforcer » [Directeur de programme, Nigéria]*

## Valeurs des systèmes de santé : les piliers

*« L’une des faiblesses de notre système de santé tient au manque de respect pour les droits des utilisateurs de nos établissements, les conditions d’hospitalisation et les consultations laissent beaucoup à désirer; l’aménagement des lieux ne garantit pas le respect minimum de la vie privée dont les patients ont besoin. En plus de lieux qui ne conviennent pas vraiment, on note aussi le problème que représente l’accueil déficient démontré par les fournisseurs de services ».* [Représentant d’une association professionnelle, Burkina Faso]

La réceptivité déficiente à l’égard des clients est apparue comme un facteur défavorable au cours de l’analyse documentaire, de même que lors des entrevues auprès d’informateurs clés. Plusieurs articles ainsi que de nombreux répondants aux entrevues auprès d’informateurs clés ont mentionné les expériences vécues par des clients qui affirmaient avoir fait l’objet d’intimidation et de réprimandes, se plaignaient que les choix étaient limités, que le traitement leur était administré en « silence » et du manque d’intimité. L’attitude négative du fournisseur de soins envers les clients et ses répercussions sur l’utilisation du service ont également été mentionnées. À titre d’exemple, les attitudes déficientes des sage-femmes qualifiées ont été citées parmi les raisons pour lesquelles les mères préféraient accoucher à la maison et refusaient les sage-femmes qualifiées. On a aussi entendu des rapports positifs faisant état de services réceptifs, doublés d’une attitude appropriée de la part des fournisseurs de soins de santé envers les clients et de la disponibilité de sage-femmes à visage humain dans les établissements de santé et on a cité ces éléments comme des raisons de faire appel aux soins en établissement [^[[43]](#endnote-43)^ ]. Ces raisons ont joué le rôle de facteurs propices.

En plus de la réceptivité déficiente envers les clients, on a également mentionné que la réceptivité déficiente des organisations envers les travailleurs (clients internes) semblait être un problème. Une étude réalisée au Ghana a montré que les perceptions d’injustice dans les relations et les processus organisationnels et le sentiment que l’organisation ne traitait pas les travailleurs de la santé avec le respect et la bienveillance auxquels ces derniers s’attendaient constituaient une source de démotivation qui se répandait et nuisait à la qualité des soins [^[[44]](#endnote-44)^]. Les conflits entre les membres du personnel contribuaient aussi à démotiver ces derniers et à influer négativement sur la qualité des soins [^[[45]](#endnote-45)^].

La recherche sur l’équité, la justice ou l’impartialité en tant que valeur dans les systèmes de santé en Afrique de l’Ouest et sur les répercussions de la mesure dans laquelle elles sont considérées comme une valeur dans le cadre des interventions, de la prestation de service et des incidences en SMNE représentait un secteur où l’on enregistrait des lacunes importantes dans la documentation. Les articles que nous avons examinés portaient de manière générale sur l’équité et on y déclarait qu’il s’agissait d’un résultat plutôt que d’une valeur. Par exemple, plusieurs faisaient état d’injustice eu égard à l’accès et au financement – comme dans l’élimination des frais – qui, de manière disproportionnée profitait aux groupes les plus aisés au Mali. Les femmes appartenant à la catégorie de revenus les plus pauvres étaient moins susceptibles d’être assurées, et ce, en dépit du montant modeste et fortement subventionné des frais d’inscription, et les ménages les plus riches affichaient le plus important recul dans les paiements de frais remboursables avec l’introduction de l’assurance santé. Toutefois, on pouvait en déduire que l’équité en tant que valeur était implicite dans la myriade de politiques sur l’exemption des frais, les régimes d’assurance-maladie communautaires (RAMC) et régimes d’assurance santé nationaux (RASN) qui avaient été mises à l’essai, et qui l’étaient toujours, dans plusieurs pays de la sous-région afin d’améliorer l’accès pour les pauvres.

De la même façon, lors des entrevues avec les informateurs clés, l’idée de l’équité, de la justice ou de l’impartialité en tant que valeurs susceptibles d’étayer de manière explicite les systèmes de santé et d’influer sur les décisions et les incidences, ne semblait pas avoir suscité le même degré d’attention que d’autres enjeux comme les interventions, la prestation de service et les composantes de base du système de santé. De manière implicite, toutefois, elle semblait présente, y compris lorsqu’un répondant mentionnait qu’il se produisait parfois un conflit de valeurs entre les perspectives d’efficience davantage axées l’économie et les perspectives d’équité et d’efficacité davantage axées sur le caractère social.

*« L’adhésion des contrôleurs financiers des systèmes de santé est très importante. Toutefois, ils n’apprécient pas toujours le fait que le système de santé est une organisation sociale qui fournit des soins de santé à la population en fonction d’un tarif social qui ne se prête pas facilement au recouvrement des coûts. »* [Directeur régional, Burkina Faso]

On a recensé de même très peu de documentation sur les droits, les responsabilités et les questions connexes à la responsabilisation. Plus particulièrement, nous faisons référence à ceux qui sont responsables de quelque chose et à ceux à qui ils doivent rendre des comptes. Nous faisons aussi référence à ceux qui ont droit à quelque chose, pour quelles raisons et à la manière dont ces droits sont pris en considération dans les systèmes de santé en Afrique de l'Ouest et aux répercussions sur les incidences en SMNE. Toutefois, certaines de ces questions sont ressorties indirectement lors d’entrevues avec des informateurs clés.

*« Sur le plan organisationnel, tout est en place, nous disposons d’une bonne structure organisationnelle à tous les niveaux et nous pouvons compter sur un cadre et une politique appropriés, mais ils sont inefficaces et comportent des lacunes sur le plan de la responsabilisation (…) personne n’est comptable devant qui que ce soit* » [Personnel d’une ONG locale, Ghana]

## Pierres d’assise (composantes de base ou matériel)

### Gouvernance

La gouvernance correspond à la manière dont le processus décisionnel est organisé et partagé entre les divers niveaux du système de santé (national, sous-national, hôpital, centre de santé et collectivité), aux capacités de gestion et de leadership entourant l’élaboration et la mise en oeuvre des politiques et des programmes en SMNE et aux mécanismes redditionnels existants dans le pays pour les systèmes de santé et la SMNE. L’analyse documentaire, de même que les entrevues avec des informateurs clés ont mis en lumière un large éventail de facteurs défavorables en lien avec la gouvernance. Ceux-ci comprenaient notamment l’établissement des priorités, la vision et la capacité de leadership. Inversement, un leadership amélioré et d’autres capacités se sont révélés propices [^[[46]](#endnote-46)^].

*« Lorsque la gouvernance fait erreur dans la détermination des priorités : il arrive parfois que des enjeux plus importants se présentent, mais que la gouvernance choisit de faire quelque chose d’autre, que les collectivités cherchent à obtenir quelque chose d’autre et ainsi de suite, donc on fait face à une erreur dans la détermination des priorités. [Personnel d’une ONG locale, Nigéria]*

« (*….) certains n’ont aucune vision, ils se retrouvent à ce poste simplement parce qu’ils y ont été nommés (….) (Ils ne) possèdent pas les connaissances ni les compétences requises pour occuper un poste de direction. Certains se retrouvent à un poste de direction sans avoir reçu la formation nécessaire, ils sont donc dans l’impossibilité de donner un bon rendement »* [Directeur national de la santé, Ghana]

On a déclaré que les hiérarchies du pouvoir institutionnel étaient fortes dans plusieurs pays et qu’elles influaient sur le processus décisionnel et sur la mise en oeuvre à bien des égards. Par exemple, l’une des conclusions de travaux menés au Sénégal en vue de déterminer les obstacles, et les facilitateurs de la mise en oeuvre des examens des décès maternels par les établissements fut que le leadership et la hiérarchie institutionnels freinaient la mise en oeuvre et les incidences de l’intervention. Plus précisément, la non-participation du chef de département lors des réunions d’audit et l’absence de rétroaction au personnel qui n’avait pas assisté aux réunions d’audit furent indiquées comme des obstacles. Les hiérarchies traditionnelles fortes dans la relation entre les médecins et les autres catégories de personnel agissaient comme un obstacle à l’établissement d’équipes multidisciplinaires. Inversement, les principaux facilitateurs étaient la participation du responsable de l’unité de la maternité, qui agissait comme modérateur lors des réunions d’audit, et la participation des gestionnaires lors de la séance d’audit en vue de planifier des mesures appropriées et réalistes pour prévenir d’autres décès maternels [^[[47]](#endnote-47)^].

La nature et la profondeur de la mise en oeuvre de la décentralisation et l’insuffisance du pouvoir décisionnel décentralisé ont joué le rôle d’un facteur limitatif compte tenu de leurs effets sur la capacité des gestionnaires intermédiaires et d’étage de réagir avec souplesse et de façon appropriée dans le contexte pour mettre les politiques et les programmes en oeuvre efficacement. Des travaux réalisés au Ghana sur l’espace décisionnel du gestionnaire de district ont montré que l’autorité hiérarchique et l’incertitude liée aux ressources avaient restreint l’espace décisionnel du gestionnaire de district. Ces contraintes ont donné naissance à un type de leadership enclin à se mettre au service des fonctions bureaucratiques du système de santé. De ce fait, la direction au niveau du district et le leadership se retrouvaient parfois limités dans leur capacité à réagir aux difficultés posées par la prestation de services liés à la santé des nouveau-nés [^[[48]](#endnote-48)^].

Dans une étude sur l’impact de la décentralisation sur les services de santé sexuelle et reproductive au Ghana, Mayhew [^[[49]](#endnote-49)^] a constaté que même si certaines décisions au sujet de l’affectation des ressources devaient être prises au niveau du district et régional, en pratique, ces décisions continuaient d’être prises centralement. Même si cette situation aurait pu être une mesure de protection nécessaire pour les services de santé sexuelle et reproductive, elle contribuait aussi à freiner certains aspects de la mise en oeuvre à l’échelle locale

Toutefois, la décentralisation n’avait pas toujours un effet positif sur les incidences. Abimbola et coll. [^[[50]](#endnote-50)^] ont trouvé que la décentralisation au Nigéria avait influencé négativement le maintien en poste des travailleurs de la santé en milieu rural de deux manières. Premièrement, le salaire des travailleurs en soins de santé primaires était souvent versé en retard et irrégulièrement en raison de retards dans les virements de fonds entre les administrations nationale et sous-nationale. Deuxièmement, la principale responsabilité eu égard aux soins de santé primaires (SSP) était souvent confiée au palier le plus faible du gouvernement : le gouvernement local. En conséquence, les travailleurs en soins de santé primaires étaient attirés par la possibilité de travailler aux niveaux des soins secondaires, administrés par le gouvernement de l’État, et aux niveaux des soins tertiaires, administrés par le gouvernement fédéral. Ces niveaux de soins se retrouvaient souvent en milieu urbain où les salaires étaient plus élevés et versés de façon plus régulière.

On pouvait aussi faire face à des défis complexes relatifs à l’uniformité de la politique nationale ainsi qu’à l’acceptation et à la mise en oeuvre du programme, selon le modèle de décentralisation.

*« (…) Un autre goulot d’étranglement est créé par le système de gouvernance complexe (…) au Nigéria, il n’est pas unitaire, par conséquent il n’est pas obligatoire pour un État de se conformer aux politiques nationales en matière de santé, nous devons déployer des trésors d’habileté dans nos démarches de défense des politiques pour susciter l’adhésion »* [Directeur national de la santé, Nigéria]

Les problèmes de gouvernance, que ce soit au niveau de l’étage ou de l’établissement, comme le leadership, les relations interpersonnelles et interprofessionnelles, les conflits entre membres du personnel, le fait que les responsables de la direction ne reconnaissent pas suffisamment les problèmes liés à la disponibilité en ressources de travailleurs de première ligne, à la motivation et aux conflits et ne prennent pas de mesures à ce sujet ont également nui à la mise en oeuvre des interventions et des programmes [^[[51]](#endnote-51)^].

*« l’ingrédient important pour renforcer le développement du système de santé est l’harmonie entre les cadres. On constate un grand manque d’harmonie dans ce secteur, et cela influe négativement sur le développement des systèmes de santé, aussi nous devons trouver le moyen d’assurer l’harmonie dans le secteur, nous devons également assurer la responsabilisation dans le cadre de la prestation de service* » [Chef de département, Nigéria]

L’obligation de rendre des comptes au public pour ceux qui prennent les décisions et qui agissent a également ressorti comme un facteur propice ou défavorable, selon les circonstances. Lodenstien et coll. [^[[52]](#endnote-52)^] ont constaté que dans les régions rurales du Mali, si les politiques de décentralisation ne tiennent pas compte de l’obligation de rendre des comptes au public, elles échoueront à modifier fondamentalement la gestion des ressources humaines, la qualité et l’équité de la dotation. Des informateurs clés ont aussi formulé des commentaires sur l’obligation de rendre des comptes et sur la question connexe de la corruption.

*« En ce qui concerne la responsabilisation, j’admets en toute sincérité que l’absence des outils nécessaires et des mécanismes redditionnels pertinents à l’intérieur du système de santé rend difficile pour les gens de se montrer responsables, même lorsqu’ils sont disposés à le faire »* [Représentant d’une ONG, Burkina Faso]

*« (…) et aussi la question de la corruption, laquelle, que ça nous plaise ou non, fait la honte de notre peuple »* [ONG locale, Nigéria]

*« Corruption et népotisme minent le système de santé au Mali. Vous savez, un directeur a besoin de personnel et de personnes compétentes pour que les choses se fassent, mais il n’est tout simplement pas possible de nommer ou de recruter des gens, quelle que soit leur compétence. À titre de directeur, je sais ce que je souhaite trouver chez un chef de département, mais tous les efforts déployés pour faire en sorte que certaines personnes occupent des postes précis n’ont pas porté fruit pour des raisons politiques. La plupart des gens qui occupent un poste dans le système de santé l’ont obtenu pour des raisons politiques, et non pour leur compétence*. [Directeur à l’échelle nationale, ministère de la Santé, Mali]

La réglementation et l’accréditation des fournisseurs de soins de santé, y compris de ceux qui fournissent des soins en SMNE, ont aussi semblé un secteur marqué par des lacunes en matière de gouvernance.

*« Étant donné que nous n’avons pas de système d’accréditation, les fournisseurs de services ne ressentent pas vraiment le besoin de mettre leurs connaissances à jour, certains n’ont même jamais suivi aucune formation additionnelle depuis l’obtention de leur diplôme »* [Directeur d’un département universitaire, Burkina Faso]

Les facteurs propices en matière de gouvernance comprenaient notamment une hiérarchie assouplie du pouvoir institutionnel, un mode de fonctionnement axé sur des équipes égalitaires, comme la prise de décisions en commun et la responsabilité eu égard aux résultats, la facilitation de l’innovation au niveau local et l’amélioration continue de même que la participation de nombreux intervenants, à divers niveaux, à la gouvernance afin d’améliorer le processus décisionnel et des arrangements rigoureux et fonctionnels en matière de responsabilisation.

### Médicaments et technologies

Parmi les facteurs propices et défavorables influençant la disponibilité et l’utilisation des médicaments et des technologies, il convient de mentionner la chaîne d’approvisionnement, la qualité des médicaments et les enjeux connexes des conditions d’entreposage pour les médicaments essentiels.

La sous-région a connu quelques réussites.

« *Le programme des produits indispensables des Nations Unies s’avère un facteur propice très convaincant, si les pays de la région peuvent maintenant l’adopter; quant au Nigéria, il l’a déjà adopté et veille à ce que ces produits soient mis à la disposition (…) Le gouvernement, en collaboration avec des partenaires, s’est engagé à rendre les produits essentiels à la planification familiale, qui comptent parmi les produits indispensables, disponibles pour tous gratuitement. (…) on produit localement la chlorhexidine qui compte aussi parmi les produits indispensables au Nigéria »* [Chef de département, Nigéria]

Malheureusement, comme c’est le cas pour de nombreux facteurs des systèmes de santé, les récits les plus répandus que la présente étude a permis de mettre au jour concernaient les difficultés liées aux pénuries, à l’insuffisance et à la non-disponibilité de médicaments, d’outils, de fournitures (y compris le sang) et de technologies essentiels, ainsi que des problèmes en lien avec l’infrastructure.

Les interventions en lien avec l’information, la communication et la technologie prennent de plus en plus d’importance dans la sous-région de l’Afrique de l'Ouest dans le cadre de projets pilotes à petite et à grande échelle. Un examen systématique du rôle des interventions en matière de santé mobile ciblant les travailleurs de la santé en vue d’améliorer les incidences liées aux grossesses dans les pays à revenu faible et à revenu intermédiaire a permis de recenser neuf études en Afrique qui satisfaisaient aux critères d’inclusion, parmi lesquelles trois s’étaient déroulées en Afrique de l'Ouest (Ghana, Nigéria et Liberia). La santé mobile a été définie comme « *une pratique médicale et de santé publique appuyée par des appareils mobiles comme les téléphones mobiles, les tablettes et autres appareils sans fil* ». Les études ont montré qu’en dépit du potentiel des interventions en matière de santé mobile, il existait des lacunes dans la base de connaissances qui concerne la manière dont elles influent sur les incidences en santé maternelle et néonatale [^[[53]](#endnote-53)^].

### Ressources humaines

Les ressources humaines influaient sur la qualité des soins, l’accessibilité, la disponibilité, l’abordabilité, l’acceptabilité et la pertinence des services en SMNE. Parmi les facteurs propices, on a noté la mise en oeuvre réussie de stratégies et d’interventions comme la délégation des tâches, la mise en place de milieux organisationnels, de climats et de cultures de travail qui encouragent et soutiennent le rendement, et la disponibilité de personnel qualifié et d’établissements de formation à l’échelle locale. Parmi les facteurs défavorables, on a noté le nombre inadéquat d’employés, la répartition inéquitable, la migration et des ressources insuffisantes pour offrir de la formation, ainsi des lacunes sur le plan de la logistique et des outils de travail. En étroite relation avec les difficultés posées par le nombre inadéquat d’employés, on a cité des problèmes liés à la compétence et aux aptitudes du personnel disponible et la pertinence des interventions en matière de renforcement des capacités. Ces carences se manifestaient de diverses manières, comme des lacunes dans les connaissances des signes de danger en obstétrique et dans la capacité de déterminer le moment et la manière appropriés pour diriger les clientes vers un autre niveau [^[[54]](#endnote-54)^].

*« On assiste à la prolifération des établissements de formation, mais les diplômés de ces établissements n’affichent plus les compétences requises pour effectuer correctement des accouchements sur le terrain »* [Directeur régional, Burkina Faso]*.*

La motivation, le degré d’empressement des travailleurs de la santé [^[[55]](#endnote-55)^] de maintenir les efforts en vue d’atteindre les buts de l’organisation [^[[56]](#endnote-56)^ ^,^^[[57]](#endnote-57),^^[[58]](#endnote-58)^], s’est avérée un important facteur propice ou défavorable. Parmi les facteurs ayant eu une incidence négative sur la motivation, on a constaté les mauvaises conditions de service, la perception de l’injustice dans la distribution des mesures d’encouragement, l’absence de protection des lieux de travail, le manque de respect et de traitement respectueux, la faible rémunération, la non-disponibilité du matériel, des outils et des fournitures essentiels, et la mauvaise qualité du milieu de travail. Une étude a fait état d’épuisement professionnel, et l’exprimait comme un épuisement émotionnel et le sentiment de dépersonnalisation. La même étude mentionnait qu’en dépit des difficultés, le personnel conservait un fort sentiment d’accomplissement et de confiance dans son travail [^[[59]](#endnote-59)^].

Le fait que les travailleurs étaient mal rémunérés a conduit, en plus de nuire à la motivation, à des comportements contre-productifs.

*« Compte tenu du fait qu’ils sont mal rémunérés, la majorité des médecins qui occupent un poste comportant des responsabilités administratives sont atteints de ce que j’appelle la « réunionite » (participation régulière à des réunions), ne serait-ce que pour obtenir assez d’indemnités journalières pour compléter leurs maigres salaires, ce qui les amène à déléguer leurs propres responsabilités à n’importe qui, y compris les employés les moins compétents »* [Directeur d’un département universitaire, Burkina Faso]

Sur le plan positif, et par conséquent, propice, une occasion d’acquérir plus de formation a été mentionnée comme le facteur le plus important pour motiver les étudiantes sage-femmes à décider à quel endroit elles iraient par la suite travailler.

Des facteurs contextuels comme l’insécurité, les conflits et l’insurrection ont influé sur l’empressement des employés à accepter des affectations et, de ce fait, sur la disponibilité, la distribution et le maintien en poste. La migration était alimentée par des facteurs contextuels et des facteurs liés au système de santé qui incitaient les employés à quitter leur emploi, de même que par des facteurs externes qui n’encourageaient pas les employés à rester.

*« La migration d’experts vers des pâturages plus verdoyants ou vers des endroits où la sécurité est mieux garantie ou encore l’avenir mieux assuré* » [Directeur national de la santé, Nigéria]

### Financement

« *Le versement des fonds ne s’effectue pas très régulièrement, aussi au niveau du district et du sous-district, on se retrouve un peu restreint dans la mise en oeuvre et on dépend majoritairement des bailleurs de fonds. De plus, lorsque le financement fourni par les bailleurs de fonds vient à manquer, la prestation de service est au point mort. »* [Partenaire du développement bilatéral, Ghana]

L’insuffisance de ressources financières pour développer et maintenir le système de santé et pour soutenir la prestation de service était un problème dans toute la sous-région. Les frais d’utilisation qui se présentent sous la forme de paiements remboursables au point de service étaient un mécanisme répandu pour essayer de mobiliser les fonds requis. Cependant, ces frais étaient un facteur défavorable comme l’ont illustré plusieurs études. Ils agissaient à titre de mesure dissuasive de l’utilisation des services, et exposaient les femmes et leur famille à des dépenses catastrophiques. Plusieurs interventions, comme les exemptions de frais d’utilisation ciblées, les régimes d’assurance-maladie communautaires et les régimes d’assurance santé nationaux avaient été mis en place ou faisaient l’objet d’un projet pilot dans la sous-région en vue d’éliminer complètement ou de réduire considérablement l’exposition des mères et des enfants à ces frais.

Plusieurs de ces interventions ont eu des effets positifs documentés dont des éléments probants de la réduction des injustices relatives à l’accès une fois que les frais d’utilisation remboursables furent éliminés. Par exemple, El-Khoury et coll. [^[[60]](#endnote-60)^] ont observé que la politique offrant gratuitement les accouchements et les césariennes au Mali avait entraîné une augmentation du nombre d’accouchements et de césariennes dans les établissements. De plus, les décès maternels et néonataux après césarienne ont chuté dans la plupart des régions entre 2006 et 2009, et cette situation est probablement due au raccourcissement des délais pour obtenir des soins d’urgence et du temps d’attente plus court dans les établissements.

Une étude réalisée dans trois pays d’Afrique de l'Ouest (Mali, Sénégal et Ghana) a montré que l’inscription à un RAMC comportait un lien positif avec l’utilisation des services de santé maternelle. Cette situation s’est vérifiée plus particulièrement dans les régions où les taux d’utilisation étaient très bas, et pour des soins liés à l’accouchement plus coûteux [^[[61]](#endnote-61)^].

Malheureusement, l’effet des interventions visant à éliminer les frais remboursables a été souvent modifié par d’autres facteurs du système de santé comme la capacité de financer la politique, la disponibilité du service, la qualité perçue du service et les contraintes liées aux ressources humaines. La politique d’exemption pour les enfants de moins de cinq ans au Ghana n’a pas fonctionné comme prévu, en partie parce que les fournisseurs n’avaient pas été remboursés en temps opportun et complètement [^[[62]](#endnote-62)^ ]. En réaction aux retards et aux défauts de remboursement, les fournisseurs de première ligne ont cessé d’offrir des exemptions et rétabli les frais d’utilisation. Le RASN faisait face à des problèmes comparables.

« *Il se peut que les gens soient inscrits au RASN. Mais je suis sûr que vous avez entendu parler de la situation de quelques établissements qui se sont retirés du Régime d’assurance santé national parce qu’ils n’avaient pas été remboursés. Donc, même si les gens se sont inscrits au Régime, étant donné que ces établissements ne leur offriront pas les services gratuitement, ils ne pourront pas bénéficier de leur assurance pour des soins de santé. (…) Il se trouve que le Régime n’a pas réglé ses dettes avec la majorité des établissements; il y a des arriérés. Cela signifie que la plupart des établissements ne seront pas en mesure d’acheter la majeure partie des choses dont ils ont besoin pour offrir des services en SMNE. Cela limite donc leur capacité à assurer un très bon service à ces clients »* [Professionnel de la santé, Ghana]

La politique visant à offrir gratuitement les césariennes au Mali que nous avons déjà mentionnée a montré qu’elle comportait des injustices sur le plan de l’accès et de l’utilisation en lien avec la disponibilité du service puisque les femmes plus aisées financièrement comptaient de manière disproportionnée parmi celles qui bénéficiaient d’une césarienne gratuite [^[[63]](#endnote-63)^ ^,^ ^[[64]](#endnote-64) ,^ ^[[65]](#endnote-65),^ ^[[66]](#endnote-66)^]. Les femmes appartenant aux deux quintiles les plus riches comptaient pour 58 % de toutes les césariennes, tandis que les femmes appartenant aux deux quintiles les plus pauvres ne représentaient que 27 % des césariennes. Dans les régions rurales du Mali [^[[67]](#endnote-67)^], certains ménages ont continué de faire face à des dépenses catastrophiques en santé pour avoir requis les services de santé maternelle, et ce, malgré la politique en vigueur. Le fait de vivre dans des régions rurales éloignées était associé avec le risque de faire face à des dépenses catastrophiques. Les femmes qui subissaient une césarienne continuaient d’engager des dépenses catastrophiques, surtout lorsque les médicaments prescrits n’étaient pas inclus dans les trousses fournies par le gouvernement pour les césariennes.

Fournier et coll. (2014) [^[[68]](#endnote-68)^ ] ont observé que pour les femmes vivant dans les villes et qui fréquentent les hôpitaux de district offrant des césariennes, les taux sont passés de 1,7 % avant que la politique n’entre en vigueur à 5,7 % 83 mois plus tard. Aucun changement significatif dans les tendances n’a pu être observé parmi les femmes vivant dans les villages dotés d’un centre de santé ou sans établissement de santé. L’abolition des frais pour les soins obstétricaux d’urgence et pour les nouveau-nés a réduit les décès maternels grâce à l’augmentation du nombre de césariennes. Cependant, l’accès aux césariennes n’était pas équitable ni accessible à celles qui vivaient dans les régions rurales [^[[69]](#endnote-69)^].

Un informateur clé a formulé un commentaire sur des problèmes comparables avec le programme au Burkina Faso.

*« En dépit du système de subvention mis en place au Burkina Faso, il reste encore des femmes incapables d’avoir accès à une césarienne dans certaines régions en raison de leur incapacité de payer pour le 20 % des frais qui restent. »* [Représentant d’une association professionnelle nationale, Burkina Faso]

### Systèmes d’information

Les lacunes en matière d’information et de documentation dans les dossiers médicaux ont nui à la qualité des données devant servir au processus décisionnel et à l’établissement des priorités. Parmi les obstacles que l’on a décelés et qui empêchaient la tenue d’audits sur les incidents évités de justesse au Bénin on a signalé une documentation inexistante ou médiocre [^[[70]](#endnote-70),^ ^[[71]](#endnote-71)^]. Les principaux obstacles à la mise en oeuvre d’examens des décès maternels au Sénégal comprenaient notamment la mauvaise qualité des renseignements dans les dossiers médicaux [^[[72]](#endnote-72)^].

La diffusion en temps opportun, l’accès à l’information tirée des recherches et d’autres sources en vue de son utilisation pour éclairer le processus décisionnel et la mise en oeuvre ont été signalés comme des facteurs défavorables.

*« Il faut toujours du temps pour que les services émergents en lien avec la SMNE soient introduits parce que les protocoles et les cadres politiques nationaux ne sont pas mis à jour régulièrement de manière à refléter les constatations de recherche actuelles et les normes de l’OMS ».* [Partenaire du développement bilatéral, Sénégal]

Sur un plan positif, on a signalé d’importantes possibilités d’améliorer l’accès aux données, et leur utilisation en vue de la prise de décision; de plus, le déploiement de données probantes pour montrer aux décideurs l’existence d’un problème pourrait influencer le processus décisionnel. Une étude sur l’établissement de programme au Ghana a montré que lorsque des données étaient disponibles, les décideurs s’en servaient pour cerner plus facilement les problèmes en santé maternelle de manière à les intégrer au programme et à les y conserver [^[[73]](#endnote-73)^].

Les systèmes d’enregistrement des faits d’état civil, en dépit de leur importance pour effectuer la surveillance, l’évaluation et la prise de décision en SMNE manquaient de ressources et leur mise en oeuvre laissait à désirer.

## Le mortier des systèmes de santé (logiciel)

*« Les relations fondées sur la confiance qui améliorent la participation de la collectivité à la prise de décision sont propices à l’amélioration des incidences en SMNE. Un médecin-chef du district et ses collaborateurs ont mis en place un système novateur pour motiver les travailleurs en santé communautaire et ont constaté une amélioration radicale de leurs indicateurs en SMNE en l’espace de deux ans. Cette relation fondée sur la confiance qui existait au sein de la collectivité a donné des résultats impressionnants, aussi nous essayons de l’adapter à grande échelle dans d’autres secteurs locaux de la santé. »***.** [Directeur à l’échelle nationale, ministère de la Santé, Bénin]

Malgré l’importance des acteurs, des processus et de l’autorité, dans le fonctionnement des systèmes de santé, la majorité des documents que nous avons trouvés étaient axés sur les composantes de base ou les interventions des systèmes de santé. Le rôle essentiel du mortier n’apparaissait souvent qu’à titre de sous-thème. Par exemple, un article qui visait principalement à évaluer une intervention sur la délégation de tâches au Sénégal pour résoudre une pénurie de longue date d’obstétriciens (en formant des équipes de district constituées d’un anesthésiste, d’un omnipraticien et d’un aide opérateur en obstétrique d’urgence) s’est heurté à plusieurs facteurs défavorables liés au « mortier ». Sur les 11 équipes chirurgicales formées entre 2001 et 2006, seulement six fonctionnaient en 2006, et le rythme de la formation n’était pas assez rapide pour couvrir tous les districts en 2015. Les raisons invoquées comprenaient notamment les perspectives diverses et concurrentes des parties prenantes eu égard au programme, aux relations, à la confiance, au pouvoir et à la motivation. Les décideurs centraux considéraient la politique plus viable que la formation de gynécologues pour les hôpitaux du district. En revanche, les médecins cliniciens universitaires principaux résistaient au programme. Un manque de possibilités d’avancement professionnel chez les médecins ayant reçu la formation, et le manque de coordination du programme étaient vus comme des obstacles par ces groupes. Les médecins étaient d’avis que le travail était utile, mais ils se plaignaient de la faible hausse de salaire et de ne pas être remplacés pendant la formation. Les collectivités appréciaient le fait que les services permettaient de sauver des vies et de l’argent, mais elles exigeaient une amélioration de l’information et de la continuité des services [^[[74]](#endnote-74)^].

L’information et l’asymétrie de l’information peuvent se révéler d’importantes sources de pouvoir ou d’absence de pouvoir. Plusieurs articles ont fait remarquer que l’accès du client à l’information et l’utilisation de l’information jouaient un rôle à titre de logiciel propice ou habilitant des systèmes de santé. Par exemple, Mills et coll. 2008 [^[[75]](#endnote-75)^] ont observé à partir d’une étude réalisée dans le nord du Ghana que la connaissance qu’avaient les femmes de l’existence de la politique sur les accouchements gratuits dans leur établissement de santé et sur les services de soins prénataux était un facteur propice à l’utilisation.

Les acteurs mondiaux clés dans le développement des systèmes de santé et de la SMNE en Afrique de l'Ouest mentionnés par les informateurs clés étaient des partenaires du développement comme l’OMS, USAID et le FNUAP. Ces acteurs et leurs agents étaient actifs à l’échelle mondiale de même qu’à l’échelle nationale. Au niveau sous-régional ouest-africain, on considérait que les acteurs clés étaient l’OOAS et le comité ou le conseil des ministres. Le rôle de l’OOAS était décrit comme à la fois politique et technique. Les informateurs clés ont également mentionné que les politiciens, surtout les titulaires de charge publique de haut niveau comme les présidents et les ministres de la santé, échangeaient avec le niveau sous-régional dans le contexte de la CÉDÉAO dans le cadre de leurs interactions avec des collègues politiciens d’autres pays faisant partie de la sous-région.

À l’intérieur des pays, on attribuait au leadership politique beaucoup de pouvoir comme celui du contrôle des ressources pour les systèmes de santé et la SMNE.

« ….. les dirigeants politiques, (…) possèdent beaucoup de pouvoir et contrôlent beaucoup d’argent; ils exercent aussi une forte influence quant à l’orientation des politiques [Gynécologue obstétricien, Ghana 27 janvier 2016]

Au niveau du pays, à part les dirigeants politiques, les hauts fonctionnaires et les administrateurs centraux nationaux comme les administrateurs en chef, les directeurs et les directeurs adjoints étaient considérés comme les principaux titulaires du pouvoir. Les acteurs non étatiques, comme la Christian Health Association of Ghana (CHAG), ont aussi été mentionnés comme des intervenants importants à l’échelle nationale. À l’échelle sous-nationale, les fournisseurs de soins de première ligne comme les médecins, les infirmières et les administrateurs pouvaient exercer un pouvoir considérable découlant souvent de leurs connaissances techniques et de leur maîtrise des services et de l’expertise.

« (…) les fournisseurs de soins de santé, qu’il s’agisse de médecins, d’infirmières, vous savez, tous ces gens, les pharmaciens, etc., qui travaillent dans le milieu des soins de santé, jouent aussi un rôle très important en regard de la SMNE. » [Gynécologue obstétricien, Ghana 27 janvier 2016]

Les administrations locales, les collectivités et les clients ont aussi été mentionnés par plusieurs répondants à titre d’acteurs clés possédant un certain pouvoir susceptible d’influer sur les incidences en SMNE à l’échelle sous-nationale.

## Contexte

L’étude a permis de déceler des facteurs contextuels à divers niveaux qui jouent un rôle important dans les systèmes de santé et la SMNE. Ils peuvent être regroupés dans les catégories socioculturelle, économique, politique, idéologique, historique, internationale et sont en interaction avec d’autres systèmes comme les axes routiers et les moyens de transport qui peuvent influer directement sur la santé. Ces facteurs contextuels agissaient à titre de facteurs propices et défavorables pour la SMNE en raison de leurs répercussions sur les systèmes de santé, ainsi que sur les déterminants sociaux de la santé.

Le contexte est en interdépendance avec le système de santé et ses composantes, et il peut entraîner une certaine variation dans les incidences. Par exemple, on a enregistré des variations dans la mortalité non seulement selon la zone géographique, mais aussi en fonction de conditions socioéconomiques et contextuelles variables comme milieu rural ou urbain, quintile de revenu, niveau d’instruction et origine ethnique de la mère. Dans une étude, le statut socioéconomique et la religion (musulmane) influençaient fortement le recours à des accoucheuses qualifiées [^[[76]](#endnote-76)^]. Les effets de la qualité de la route sur l’aiguillage, et plus particulièrement le fait que les routes n’étaient pas très sécuritaires pendant la nuit, ont déjà été mentionnés [^[[77]](#endnote-77)^]. D’autres systèmes comme l’approvisionnement en eau et les installations sanitaires, ainsi que la sécurité alimentaire et nutritionnelle influaient aussi sur les incidences en SMNE.

Les facteurs socioculturels comprenaient notamment la condition féminine, et l’influence des autres ménages et des membres de la collectivité sur le recours aux soins et la prise de décision dans les contextes où les décisions appartiennent aux membres de la famille élargie plutôt qu’à l’individu. Les autres facteurs comprenaient notamment une condition féminine déplorable qui se traduit par l’absence de contrôle sur la prise de décision, et la faible valeur accordée à l’éducation des filles, ce qui ne fait que renforcer le déséquilibre du pouvoir domestique entre les hommes et les femmes, ainsi que la condition inférieure et la dépendance des femmes. L’alphabétisation (l’éducation des mères) était un indicateur de maternité sans risque. Certains articles indiquaient que l’on attendait parfois des femmes qu’elles justifient des besoins qui paraissaient moins évidents dans le cadre d’un processus de négociation inégal avec des possibilités de recours ambivalentes, et qu’elles étaient susceptibles de faire l’objet de retards, ou même d’une exclusion des soins de santé, d’une faible estime de soi et d’un déséquilibre sur le plan du pouvoir domestique. Tous ces facteurs se traduisaient par des répercussions sur l’accès aux services et leur utilisation.

Des facteurs macroéconomiques, l’accès du ménage à un revenu, de même que la capacité d’avoir accès aux soins de santé requis par l’entremise de leurs répercussions sur les revenus nationaux et des ménages. Les transitions politiques, les idéologies, les priorités et l’existence de défenseurs du programme exerçaient aussi une influence sur le bon déroulement de la SMNE.

« *Au Ghana, la volonté politique et l’engagement sont très forts (en regard de la SMNE)(…). On a constaté une forte volonté politique, et des ressources nationales ont été mobilisées à cet effet. »* [Partenaire du développement bilatéral, Ghana]

# Conclusions

Tout effort visant à atteindre les objectifs 4 et 5 du Millénaire pour le développement en Afrique de l'Ouest doit reconnaître la futilité d’investir principalement dans les interventions et de négliger relativement le système de santé et le contexte. Il est en effet essentiel de rapprocher plus efficacement ces frontières traditionnelles et de veiller à ce que les investissements dans les trois aspects s’effectuent de concert, en mettant l’accent sur le renforcement des systèmes de santé et du contexte afin de permettre la mise en oeuvre efficiente et efficace d’interventions vitales éprouvées. Les modèles conceptuels qui s’éloignent de l’approche cloisonnée actuelle joueront un rôle essentiel dans la réflexion sur les enjeux et les solutions à l’échelle mondiale, nationale et sous-nationale.

Tablant sur les constatations de cette étude, nous proposons la figure 2 à titre de simple heuristique pour guider les chercheurs, les décideurs et les responsables de la mise en oeuvre dans leur analyse, la production de données probantes et la prise de décision afin d’élaborer des politiques et programmes mieux intégrés pour accélérer les améliorations en matière de SMNE en Afrique de l'Ouest.

**FIGURE 2 – systèmes de santé et heuristique de la smne**


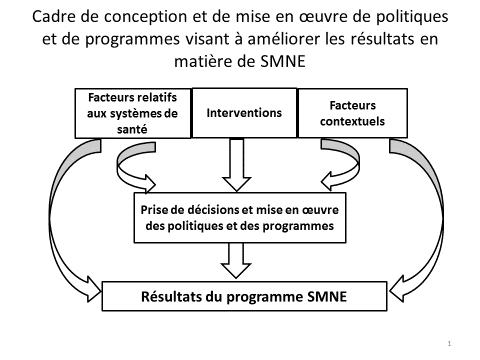


À l’instar de toute autre heuristique, la figure 2 est un support pratique pour l’analyse et la prise de décision plutôt qu’un modèle explicatif parfait. Il s’agit d’un cadre dont l’utilisation encouragera les décideurs à tous les niveaux des systèmes de santé en Afrique subsaharienne à se démarquer de la focalisation prépondérante actuelle sur les interventions en matière de SMNE. De telles approches passent sous silence les facteurs propices et défavorables liés aux systèmes de santé et les facteurs contextuels qui sont étroitement liés à la manière dont les politiques et les programmes ou interventions correspondants fonctionnent en pratique et qui, par conséquent, au bout du compte, influent sur leur efficacité et contribuent d’une manière ou d’une autre à améliorer les incidences en SMNE.

Des interventions en matière de SMNE « éprouvées » ou « présumées efficaces » sont mises en place en partant de l’hypothèse qu’elles amélioreront les incidences en SMNE. Cependant, tous les effets sont modifiés par des mécanismes eux-mêmes influencés par la décision consistant à déterminer comment l’intervention est mise en oeuvre en pratique, ainsi que par les conditions dans lesquelles le système de santé est mis en oeuvre, et le contexte dans lequel le système de santé évolue. L’un de ces facteurs ou chacun de ces facteurs peut agir indépendamment ou en synergie pour devenir propice ou défavorable aux processus de mise en oeuvre.

On trouvera à la figure 3 une simple illustration de l’analyse qui mise sur ce cadre à partir de l’aiguillage vers des soins obstétricaux d’urgence.


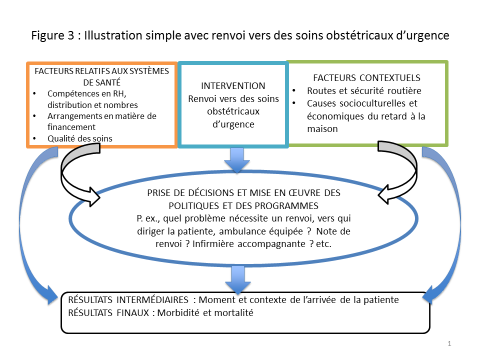


Dans les systèmes de santé, plusieurs interventions sont simultanément mises en place. Ainsi, plusieurs cycles sont interdépendants et s’influencent les uns les autres, ce qui ajoute à la complexité. Mais cette situation complexe est illustrée, sur le mode simplifié, à la figure 4.


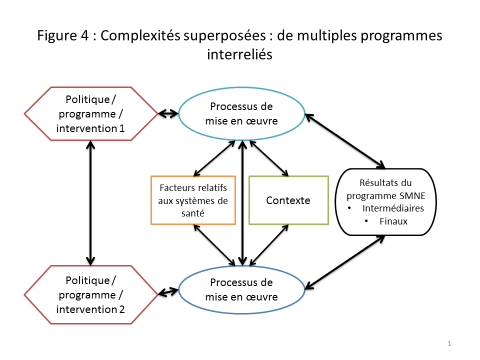


**Limitations de l’étude**

Nous avons dû exclure la documentation en portugais pour des raisons pratiques liées au coût de l’inclusion. Cela soulève la possibilité que des différences existent dans les pays lusophones du Cap-Vert et de la Guinée Bissau ainsi que dans le reste de la CÉDÉAO et que ces différences aient échappé à notre analyse. Deuxièmement, notre étude était entièrement qualitative et nous sommes dans l’impossibilité de fournir des données empiriques relatives à l’ordre de grandeur de nos observations dans toute l’Afrique de l'Ouest. Cet aspect fera partie du programme de recherches futures. Il convient toutefois de mentionner que tous les facteurs propices et défavorables mentionnés dans le présent article ne se prêtent pas à la quantification. Valeurs, pouvoir et processus dans les systèmes de santé et leur rôle à titre de facteurs propices et défavorables en regard de l’amélioration de la SMNE ne sont pas nécessairement quantifiables. Le programme de recherche pour ces facteurs sera la nécessité de disposer de plus de recherche qualitative pour améliorer la compréhension.

**Liste des abréviations utilisées**

ASN = Assurance santé nationale

CÉDÉAO = Communauté économique des États de l’Afrique de l’Ouest

FNUAP = Fonds des Nations Unies pour la population

IC = Informateur clé

ISMEA = Innovation pour la santé des mères et des enfants d’Afrique

OMS = Organisation mondiale de la Santé

ONG = Organisation non gouvernementale

OOAS = Organisation ouest-africaine de la santé

OSC = Organisation de la société civile

RAMC = Régimes d’assurance-maladie communautaires

RASN = Régime d’assurance santé national

RSS = Recherche sur les systèmes de santé

SMNE = Santé des mères, des nouveau-nés et des enfants

SSP = Soins de santé primaires

USAID = United States Agency for International Development

**Contribution des auteurs**

Tous les auteurs ont participé à l’élaboration du concept de l’étude. IAA, AK, SD, EF, GEA et AI ont effectué l’analyse documentaire. VL a fourni son assistance pour prendre les arrangements nécessaires, et SD (français) et EF (anglais) ont mené les entrevues avec les informateurs clés. IAA, SD et EF ont analysé les entrevues avec les informateurs clés. IAA, AK, VL et IS ont participé aux ateliers de validation des parties prenantes à l’échelle sous-régionale. IAA a conceptualisé et rédigé l’article. Tous les auteurs ont lu et approuvé la version définitive de l’article.

**Attestations de conformité à l’éthique**

Les attestations de conformité à l’éthique ont été délivrées par la Division de la recherche-développement du Service de santé du Ghana. N^o^ d’id. du protocole : GHS-ERC 07/09/15. Toutes les entrevues ont été menées après avoir obtenu le consentement libre et éclairé.

**Intérêts divergents**

Aucun intérêt divergent n’a été signalé.

**Financement**

L’étude a été subventionnée par le programme Innovation pour la santé des mères et des enfants d’Afrique (ISMEA) du Centre de recherches pour le développement international (CRDI) du Canada, les Instituts de recherche en santé du Canada (IRSC) et Affaires mondiales Canada; ainsi que par la commission de la Communauté économique des États de l’Afrique de l’Ouest (CÉDÉAO).

# Références

1. IHME juillet 2014. Données sur la charge mondiale de morbidité 2013. https://www.healthdata.org/results [↑](#endnote-ref-1)
2. Victoria C.G., Requejo J.H., Barros A.J.D. et coll. Countdown to 2015: a decade of tracking progress for maternal, newborn, and child survival. Lancet 2015 [www.thelancet.com](http://www.thelancet.com). Publié en ligne le 16 octobre 2015. <http://dx.doi,org/10.1016/50140.6736(15000519.X> [↑](#endnote-ref-2)
3. L’Agence Nationale de la Statistique et ICF International 2012. 2010-11 Senegal Demographic and Health and Muliple Indicator Survey. Key Findings. Calverton, Maryland. USA. ANSD et ICF Int. <https://dhsprogram.com/pubs/pdf/SR192/SR192.pdf> [↑](#endnote-ref-3)
4. http://www.who.int/maternal_child_adolescent/epidemiology/profiles/neonatal_child/en/ [↑](#endnote-ref-4)
5. Rutstein S., Ayad M., Ruilin Ren., Rathavuth H. 2009. Benin Further Analysis. Changing Health Conditions and Decline of Infant and Child Mortality in Benin. Further analysis of the 2006 Benin DHS. ICF Macro. Calverton, Maryland, USA. Avril 2009. <https://dhsprogram.com/pubs/pdf/FA65/FA65.pdf> [↑](#endnote-ref-5)
6. Cellule de Planification et de Statistique (CPS/SSDSPF), Institut National de la Statistique (INSTAT/MPATP), INFO-STAT et ICF International 2014. Enquête Démographique et de Santé au Mali 2012-2013 [↑](#endnote-ref-6)
7. Ghana Statistical Service (GSS), Ghana Health Service (GHS), et ICF International 2015. Ghana Demographic and Health Survey 2014. Rockville, Maryland, USA: GSS, GHS et ICF International [↑](#endnote-ref-7)
8. Bhutta Z., Das J.K., Bahl R., Lawn J.E., Salam R.A., Paul V.K., Sankar M.J., Blencowe H., Rizvi A., Chou V.B., Walker N., (2014) Can available interventions end preventable deaths in mothers, newborn babies, and stillbirths, and at what cost? Lancet 2014; 384: 347 – 70 [↑](#endnote-ref-8)
9. Sambo L.G. Kirigia J.M. Investing in health systems for universal health coverage in Africa. BMC International Health and Human Rights. 2014; 14:28. http:/www.biomedcentral.com/1472-698X/14/28 [↑](#endnote-ref-9)
10. Kirigia J.M., Nabyonga-Orem & Dovlo D.Y.T. Space and place for WHO health development dialogues in the African Region. BMC Health Services Research 2016. 16 (Suppl ): 221 DOI 10.1186/s12913-016-1452-0 [↑](#endnote-ref-10)
11. Sheikh K., Schneider H., Agyepong I.A., Lehmann U., Gilson L. (2016) Boundary Spanning: Reflections on the practices and principles of Global Health. BMJ Global Health 2016; 1:e000058. Doi:10.1136/bmjgh-2016-000058 [↑](#endnote-ref-11)
12. Williams P. The Competent Boundary Spanner. Public Admin. 2002; 80:103 – 124 [↑](#endnote-ref-12)
13. Murray C.J.L. et Frenk J. Cadre pour l’évaluation des services de santé. Bulletin de l’Organisation mondiale de la Santé 2000; 78(6). pages 717-722 [↑](#endnote-ref-13)
14. Hsiao W.C. (2003) What is a Health System? Why Should We Care? Cambridge, MA: Havard School of Public Health. http://www.econ.boun.edu.tr/content/document/ec48801/7150.pdf (Jan 6, 2015) [↑](#endnote-ref-14)
15. Banque mondiale (2007) Pour un développement sain : Stratégie de la Banque mondiale pour l’amélioration des résultats en matière de santé, nutrition et population [pages 44-47] [↑](#endnote-ref-15)
16. Shakrishvili et coll. (2000) Converging Health Systems Frameworks: Towards A Concepts-to-Actions Roadmap for Health Systems Strengthening in Low and Middle Income Countries. Global Health Governance, Volume iii, no. 2 [↑](#endnote-ref-16)
17. van Olmen J., Criel B., van Damme W., Marchal B., van Belle S., van Dormael M., Hoerée T., Pirard M. et Kegels G. (2010) Analyzing Health Systems To Make Them Stronger. Studies in Health Services Organisation & Policy, 27 [↑](#endnote-ref-17)
18. OMS (2007) Everybody’s business : strengthening health systems to improve health outcomes : WHO’s framework for action [↑](#endnote-ref-18)
19. OMS (2000) Rapport sur la santé dans le monde 2000. Pour un système de santé plus performant [↑](#endnote-ref-19)
20. Smith P.C., Mossialos E., Papanicolas I., Leatherman S. (2010). Performance Management for Health Improvement – Experiences, Challenges and Prospects. Health Economics, Policy and Management. [pages 1-6] [↑](#endnote-ref-20)
21. Amala de Silva (…) A Framework for measuring responsiveness. GPE Discussion Paper Series No 32. EIP/GPE/EBD Organisation mondiale de la Santé [↑](#endnote-ref-21)
22. Robone S. Rice N. Smith P.C. (2011) Health System Responsiveness and its characteristics. A Cross Country Comparative Analysis. Health Services Research 46:6 part II. Pp 2079 – 2100 [↑](#endnote-ref-22)
23. Sheikh K., Ransom M.K., Gilson L. (2014) Explorations on people centeredness in health systems. Health Policy and Planning Volume 29, Supplement 2, octobre 2014. Editorial [↑](#endnote-ref-23)
24. Henry Mintzberg (1983) Power In and Around Organizations. Prentice Hall Inc. Englewood Cliffs, N.J. 07632. ISBN 0-13-686857-6 [↑](#endnote-ref-24)
25. Amala de Silva (…) A Framework for measuring responsiveness. GPE Discussion Paper Series No 32. EIP/GPE/EBD Organisation mondiale de la Santé [↑](#endnote-ref-25)
26. Leventhal, G.S. (1976), *What should be done with equity theory? New approaches to the study of fairness in social relationships*. Washington, DC: National Science Foundation, septembre 1976. Consultable auprès du département de psychologie, Wayne State University, Detroit, Michigan 48202. Aussi à http://www.eric.ed.gov/ERICWebPortal. [↑](#endnote-ref-26)
27. <http://www.dictionary.com/browse/context> [↑](#endnote-ref-27)
28. Grindle M.S. et Thomas J.W. (1991) Public *Choices and Policy Change. The Political Economy of reform in developing countries.* The John Hopkins University Press. Chapitre 2, p. 37 – 40 The context of policy choice

    Grindle M.S., Thomas J.W. (1991) Public Choices and Policy Change. The Political Economy of Reform in Developing Countries. The John Hopkins University Press [↑](#endnote-ref-28)
29. Collins C. Green A. et Hunter D. (1999) Health Sector Reform and the interpretation of policy context. Health Policy 47: 69 – 83 [↑](#endnote-ref-29)
30. Bausch D.G. et Schwarz L. (2014) Outbreak of Ebola Virus Disease in Guinea: Where Ecology Meets Economy. PLoS Neglected Tropical Diseases. Vol 8, numéro 7. E3056. www.plosntds.org [↑](#endnote-ref-30)
31. Afulani PA (2015) Rural/Urban and Socioeconomic Differentials in Quality of Antenatal Care in Ghana. *PLoS ONE* 10(2): e0117996. Doi:10.1371/journal.pone.0117996 [↑](#endnote-ref-31)
32. Appiah-Kubi K (2004). Access and utilization of safe motherhood services of expecting mothers in Ghana*. Policy & Politics*; Vol. 32, No. 3: 387-407 [↑](#endnote-ref-32)
33. Coffie L et coll. (2015). Birth location preferences of mothers and fathers in rural Ghana: Implications for pregnancy, labor and birth outcomes. *BMC Preg. & Childbirth*; DOI 10.1186/s12884-015-0604-2 [↑](#endnote-ref-33)
34. Mills S et coll. (2008). Use of Health Professionals for Delivery Following the Availability of Free Obstetric Care in Northern Ghana. *Matern Child Health Journal*; DOI 10.1007/s10995-007-0288-y [↑](#endnote-ref-34)
35. Hounton et coll. Accessibility and utilisation of delivery care in rural Burkina Faso. *Tropical Medicine and International Health* Vol. 13 suppl. 1:44–52 [↑](#endnote-ref-35)
36. Ansong-Tornui et coll. (2007). Hospital-based maternity care in Ghana: Findings of a confidential enquiry into maternal deaths. *Ghana Medical Journal*; Vol. 41 No.3 [↑](#endnote-ref-36)
37. Nikiema et coll. (2010). Quality of Antenatal Care and Obstetrical Coverage in Rural Burkina Faso. *J HEALTH POPUL NUTR*; 28(1):67-75 [↑](#endnote-ref-37)
38. Graham et coll. (2008). Evaluating skilled care at delivery in Burkina Faso: principles and practice. *Tropical Medicine and International Health.* Doi:10.1111/j.1365-3156.2008.02082.x [↑](#endnote-ref-38)
39. Achem F.F., Agboghormoma C.O. Setting up facility based maternal death reviews in Nigeria. Commentary. Royal College of Obstetricians and Gynecologists. 2014 [www.bjog.org](http://www.bjog.org) DOI:10.1111/1471-0528.12817 [↑](#endnote-ref-39)
40. Afari H, et coll. (2014). Quality improvement in emergency obstetric referrals: qualitative study of provider perspectives in Assin North district, Ghana. *BMJ Open*; Doi:10.1136/bmjopen-2014-005052 [↑](#endnote-ref-40)
41. Afari H, et coll. (2014). Quality improvement in emergency obstetric referrals: qualitative study of provider perspectives in Assin North district, Ghana. *BMJ Open*; Doi:10.1136/bmjopen-2014-005052 [↑](#endnote-ref-41)
42. Merali H.S., Lipsitz S., Hevelone N., Gawande A.A., Lashoher A., Agrawal P. Spector J. Audit-identified avoidable factors in maternal and perinatal deaths in low resource settings: a systematic review. BMC Pregnancy and Childbirth 2014. 14:280 <http://www.biomedcentral.com/1471-2393/14/280> [↑](#endnote-ref-42)
43. Coffie L et coll. (2015). Birth location preferences of mothers and fathers in rural Ghana: Implications for pregnancy, labor and birth outcomes. *BMC Preg. & Childbirth 2015*; DOI 10.1186/s12884-015-0604-2 [↑](#endnote-ref-43)
44. Aberese Ako M., van Djik H., Gerrits T., Arhinful D.K., **Agyepong I.A.** (2014) “Your health our concern, our health whose concern?”: perceptions of injustice in organizational relationships and processes and frontline health worker motivation in Ghana. Health Policy and Planning Volume 29 Supplement 2; octobre 2014 ISSN 0268-1080. Pp ii15 – ii28 [↑](#endnote-ref-44)
45. Aberese-Ako M., **Agyepong I.A**, Gerrits T., Van Djik H. (2015) “I used to fight with them but now I have stopped!”: Conflict and Doctor-Nurse-Anaesthetists Motivation in Maternal and Neonatal care provision in a Specialist Referral hospital. PLoS One 10(8): e0135129.doi:10.1371/journal [↑](#endnote-ref-45)
46. Taylor 2015. The role of health professional organizations in improving maternal and newborn health: The FIGO LOGIC experience [↑](#endnote-ref-46)
47. Alexandre Dumont, Caroline Touringny, Pierre Fournier (2009) Improving obstetric care in low-resource settings: implementation of facility-based maternal death reviews in five pilot hospitals in Senegal. Human Resources for Health 2009. 7:61 doi:10.1186/1478-4491-7-61. http://www.human-resources-health.com/content/7/1/61 [↑](#endnote-ref-47)
48. What Governs District Manager Decision Making? A Case Study of Complex Leadership in Dangme West District, Ghana (Kwamie et coll., 2015) [↑](#endnote-ref-48)
49. The Impact of Decentralisation on Sexual and Reproductive Health Services in Ghana

    (Mayhew 2003) [↑](#endnote-ref-49)
50. How decentralisation influences the retention of primary health care workers in rural Nigeria

    (Abimbola et coll. 2014) [↑](#endnote-ref-50)
51. Aberese Ako M., van Djik H., Gerrits T., Arhinful D.K., **Agyepong I.A.** (2014) “Your health our concern, our health whose concern?”: perceptions of injustice in organizational relationships and processes and frontline health worker motivation in Ghana. Health Policy and Planning Volume 29 Supplement 2; octobre 2014 ISSN 0268-1080. Pp ii15 – ii28 [↑](#endnote-ref-51)
52. Devolution and human resources in primary healthcare in rural Mali (Lodenstein et Dao 2011) [↑](#endnote-ref-52)
53. Amoakoh-Coleman M. (2016) Chapter 7 in Pregnancy Outcomes in Ghana. Université d’Utrecht, Pays-Bas, thèse de doctorat [↑](#endnote-ref-53)
54. Afari H, et coll. (2014). Quality improvement in emergency obstetric referrals: qualitative study of provider perspectives in Assin North district, Ghana. *BMJ Open*; Doi:10.1136/bmjopen-2014-005052 [↑](#endnote-ref-54)
55. MCLAUGHLIN, C. et KALUZNY, A. 1994. *Continuous quality improvement in Health care: Theory, implementation and application. 2^e^ éd.,* Gaithersburg, Maryland, Aspen publishers, Inc. [↑](#endnote-ref-55)
56. FRANCO L M, BENNETT S, KANFER R & P., S. 2004. Determinants and consequences of health worker motivation in hospitals in Jordan and Georgia. *Social Science and Medicine,* 58**,** 343-355. [↑](#endnote-ref-56)
57. FRANCO, L. M., BENNETT, S. et KANFER, R. 2002. Health sector reform and public sector health worker motivation: a conceptual framework. *Social Science and Medicine,* 54**,** 1255-1266. [↑](#endnote-ref-57)
58. KANFER, R. 1999. Measuring Health Worker Motivation in Developing Countries. *Major Applied Research 5.* [↑](#endnote-ref-58)
59. Rouleau D., Fournier P., Philibert A., Mbengue B., Dumont A. Human Resources for Health 2012. 10.9 http://www.human-resources-health.com/content/10/1/9 [↑](#endnote-ref-59)
60. El-Khoury et coll. 2011 (op. cit.) [↑](#endnote-ref-60)
61. Smith, K. V., et Sulzbach, S. (2008). Community-based health insurance and access to maternal health services: Evidence from three West African countries. *Social Science & Medicine, 66*(12), 2460-2473. doi: <http://dx.doi.org/10.1016/j.socscimed.2008.01.044> [↑](#endnote-ref-61)
62. Agyepong IA, Nagai RA. (2011) ["We charge them; otherwise we cannot run the hospital" front line workers, clients and health financing policy implementation gaps in Ghana.](http://www.ncbi.nlm.nih.gov/pubmed/21071106) Health Policy 99. Pp 226 - 233 [↑](#endnote-ref-62)
63. Marianne El-Khoury, Timothee Gandaho, Aneesa Arur, Binta Keita, et Lisa Nichols. Avril 2011. Improving Access to Life-saving Maternal Health Services: The Effects of Removing User Fees for Caesareans in Mali. Bethesda, MD: Health Systems 20/20, Abt Associates Inc. [↑](#endnote-ref-63)
64. Pierre Fournier, Alexandre Dumont, Caroline Tourigny, Aline Philibert, Aliou Coulibaly, Mamadou Traore (2014). The Free Caesareans Policy in Low-Income Settings: An Interrupted Time Series Analysis in Mali (2003–2012). *PloS One* DOI: 10.1371/journal.pone.0105130 [↑](#endnote-ref-64)
65. Marion Ravit, Aline Philibert, Caroline Tourigny, Mamadou Traore, Aliou Coulibaly, Alexandre Dumont, Pierre Fournier (2015) The Hidden Costs of a Free Caesarean Session Policy in West Africa (Kayes Region, Mali) [↑](#endnote-ref-65)
66. El-Khoury M, Hatt L, Gandaho T. User fee exemptions and equity in access to caesarean sections: an analysis of patient survey data in Mali. *International Journal for Equity in Health*. 2012;11:49. doi:10.1186/1475-9276-11-49. [↑](#endnote-ref-66)
67. Arsenault, Catherine, Fournier, Pierre, Philibert, Aline, Sissoko, Koman, Coulibaly, Aliou, Tourigny, Caroline, Traoré, Mamadou, et Dumont, Alexandre. (2013). Accès aux soins obstétricaux d’urgence au Mali : dépenses catastrophiques et conséquences au sein des ménages. *Bulletin of the World Health Organization*, *91*(3), 207-216 [↑](#endnote-ref-67)
68. Fournier et coll. 2014 (op. cit.) [↑](#endnote-ref-68)
69. Ravit, M., Philibert, A., Tourigny, C., Traore, M., Coulibaly, A., Dumont, A., et Fournier, P. (2015). The Hidden Costs of a Free Caesarean Section Policy in West Africa (Kayes Region, Mali). *Maternal and Child Health Journal, 19*(8), 1734-1743. doi: 10.1007/s10995-015-1687-0 [↑](#endnote-ref-69)
70. Hutchinson C., Lange I., Kanhonou L., Filippi V., Borchert M. Exploring the sustainability of obstetric near-miss case review: a qualitative study in the South of Benin. Midwifery 26 (2010) 537 – 543 [↑](#endnote-ref-70)
71. Saizonou J., de Brouwere V., Vangeenderhuysen C., Dramaix-Wilment M., Buekens P., Dujardin B. Audit de la qualité de prise en charge des « échappées belle » (near miss) dans les maternités de référence du Sud Bénin. Cahiers Santé, vol. 16 no. 1, janvier-février-mars 2006 p. 33 – 42 [↑](#endnote-ref-71)
72. Improving obstetric care in low-resource settings: implementation of facility-based maternal death reviews in five pilot hospitals in Senegal (Dumont et coll. 2009) [↑](#endnote-ref-72)
73. Koduah A., Dijk H., **Agyepong I.A.** (2015) The role of policy actors and contextual factors in policy agenda setting and formulation: maternal fee exemptions policies in Ghana over four and a half decades. Health Research Policy and Systems. Doi:10.1186/512961-015-0016-9 [↑](#endnote-ref-73)
74. Vincent De Brouwere, Thierro Dieng, Mohamed Diadhiou, Sophie Witter, Ernest Denerville (2009) Task shifting for emergency obstetric surgery in district hospitals in Senegal

    Ernest Denerville (2009) Reproductive Health Matters 2009; 17(33): 32 – 44 [↑](#endnote-ref-74)
75. Mills S et coll. (2008). Use of Health Professionals for Delivery Following the Availability of Free Obstetric Care in Northern Ghana. *Matern Child Health Journal*; DOI 10.1007/s10995-007-0288-y [↑](#endnote-ref-75)
76. Mills S et coll. (2008). Use of Health Professionals for Delivery Following the Availability of Free Obstetric Care in Northern Ghana. *Matern Child Health Journal*; DOI 10.1007/s10995-007-0288-y [↑](#endnote-ref-76)
77. Afari et coll. 2014 (op. cit.) [↑](#endnote-ref-77)
