# Supplementary material for: People and research: improved health systems for West Africans, by West Africans - report on special supplement
Source: BMC Proc. 2019 Feb 7;13(Suppl 1):1. doi: 10.1186/s12919-019-0162-0 (PMC6366023; doi:10.1186/s12919-019-0162-0)
Supplement: Supplementary file 7 — Les modèles voyageurs à l’épreuve des contextes et des normes pratiques: le cas de la santé maternelle, Olivier de Sardan, J-P., Diarra, A., Moha, M. [file 12919_2019_162_MOESM7_ESM.docx]

| ***Les modèles voyageurs à l’épreuve des contextes et des normes pratiques: le cas de la santé maternelle***  **Jean-Pierre Olivier de Sardan*, Aïssa Diarra and Mahaman Moha**  * Correspondance : [jeanpierre.olivierdesardan@ird.fr](mailto:jeanpierre.olivierdesardan@ird.fr) LASDEL, Niamey, Niger Résumé Comme c’est le cas dans d’autres domaines du développement international, nous assistons à la prolifération de « modèles voyageurs » mis au point par des experts internationaux et introduits sous une forme presque identique dans de nombreux pays pour améliorer certains aspects des systèmes de santé maternelle dans les pays à revenu faible et intermédiaire. Ces politiques et protocoles sont fondés sur des « mécanismes miracles » qui ont été sortis de leur contexte original, mais dont on croit qu’ils sont intrinsèquement efficaces à la lumière de leurs dispositifs opérationnels.  En réalité, les interventions standardisées sont, en Afrique et ailleurs, confrontées à des contextes de mise en oeuvre pragmatiques, toujours variés et spécifiques, qui conduisent à des dérives, des distorsions, des démembrements et des contournements. Le partogramme, les soins prénataux ciblés, la prévention de la transmission du VIH de la mère à l’enfant ou le paiement basé sur la performance illustrent tous ces écarts de mise en oeuvre, souvent causés par le comportement de routine du personnel de santé qui suit des normes pratiques (et une culture professionnelle) qui sont souvent distinctes des normes officielles – comme c’est le cas pour les sages-femmes.  Les expériences en matière de santé maternelle et infantile en Afrique suggèrent qu’une nouvelle stratégie consisterait à commencer par la réalité quotidienne des normes sociales et pratiques au lieu de s’appuyer sur des modèles, et à promouvoir les innovations qui émergent des systèmes de santé locaux. |
| --- |

# Contexte

Dans le domaine de la santé maternelle (comme dans le domaine de la santé publique et même, plus généralement, dans le domaine du développement), les interventions promues par les organisations internationales, les coopérations bi-latérales et les ONG en direction des LMIC, et plus encore de l’Afrique, sont le plus souvent fortement standardisées, tant dans un souci de simplification (déficits importants en infrastructures et en compétences) que dans un souci de large diffusion (recherche de hauts facteurs d’impact). La culture bio-médicale, qui repose largement sur des approches statistiques et des protocoles harmonisés, amplifie cette tendance. On assiste donc à la multiplication de « modèles voyageurs », élaborés par des experts internationaux en vue d’améliorer tel ou tel aspect des systèmes de santé maternelle dans les LMIC, qui sont introduits dans un format quasi identique dans un grand nombre de pays.

Ces interventions standardisées sont, en Afrique comme ailleurs, soumises à l’épreuve redoutable des contextes de mise en oeuvre, toujours très divers, et largement méconnus des promoteurs des interventions. Les aléas et les difficultés de la mise en oeuvre de politiques publiques ont été depuis longtemps mis en évidence du côté des sciences politiques [1–5], et du côté de l’anthropologie du développement [6, 7], mais restent encore relativement peu documentés du côté de la santé publique, malgré une prise de conscience progressive [8–13]. La santé publique est en effet dominée par l’assimilation de toute « médecine fondée sur les données probantes » (et de toute « politique fondée sur les données probantes ») à des données essentiellement quantitatives et expérimentalistes (pour un document de plaidoyer concernant la santé maternelle, voir Miller et coll. [14]), qui favorisent et justifient de fait la standardisation des interventions. Ces dernières années toutefois, divers courants théoriques ont insisté sur l’importance des contextes face aux interventions de santé, que ce soit les approches réalistes en évaluation [15, 16], les approches insistant sur la complexité des systèmes de santé [17, 18], les « études sur la mise en oeuvre » [19, 20] ou les appels à prendre en compte les « contextes réels » [21, 22]. La plupart d’entre eux utilisent une approche qualitative ou mixte

Néanmoins, la standardisation des interventions en santé publique reste peu contestée au niveau opérationnel, elle semble « intouchable », et son rôle majeur dans la méconnaissance ou la sous-estimation des contextes est très généralement négligé.

Nous voudrions ici développer une approche théorique ancrée dans la réalité (grounded theory) [23] permettant de mieux comprendre la relation entre interventions standardisées et contextes de mise en oeuvre, et les nombreux effets inattendus, invisibles ou pervers qui en découlent. Pour ce faire, nous proposerons quelques concepts élaborés à partir de nos recherches en Afrique de l’Ouest dans le domaine de la santé maternelle, tels que les « mécanismes » et « dispositifs » des modèles voyageurs, la distinction entre les « contextes structurels » et « contextes pragmatiques », et finalement les « normes pratiques » et les « cultures professionnelles ». Nous utilisons donc divers exemples de mise en oeuvre d’interventions standardisées que nous avons étudiés au fil de nos enquêtes (le partogramme, la consultation prénatale recentrée, la prévention de la transmission du VIH de la mère à l’enfant, le paiement basé sur la performance), qui sont particulièrement significatifs des « épreuves » que les contextes font subir à ces interventions. Nous nous appuyons ici sur les recherches, menées depuis 15 ans au Niger et en Afrique de l’Ouest, par le LASDEL (Laboratoire d’études et Recherches sur les Dynamiques Sociales et le Développement Local) sur la santé et plus particulièrement sur la santé maternelle [24–30]. Depuis 2012, ces recherches sont insérées dans un programme financé par le CRDI sur « Les problèmes négligés des systèmes de santé en Afrique : une incitation aux réformes » qui comprend en particulier un volet sur les sages-femmes. Il s’agit de comprendre comment les multiples et incessantes réformes et interventions standardisées, sous la forme de nouvelles politiques ou de nouveaux protocoles en « prêt-à-porter », sont fondées sur la promotion par des experts internationaux de « mécanismes » miracles, extraits de leurs contextes d’émergence, et censés avoir une efficacité intrinsèque grâce à leurs « dispositifs » opérationnels; et comment ces « modèles voyageurs » (qui relèvent toujours de l’ingénierie sociale quel que soit leur fondement biomédical) font l’objet, lorsqu’ils sont mis en oeuvre dans des contextes très différents, d’adoptions toujours sélectives par les acteurs locaux, et souvent de rejets dissimulés, et sont assez généralement « contournées » ou désarticulées.

Faute d’études qualitatives approfondies, les diverses « revanche des contextes » restent très peu documentées, et la course à de nouveaux modèles voyageurs continue de plus belle. Pourtant, comme nous le verrons en conclusion, il existe des alternatives, soit en permettant aux modèles voyageurs de se transformer eux-mêmes en fonction des réactions qu’ils suscitent, soit en promouvant des réformes « sur mesure » à partir des contextes locaux et des pratiques routinières des agents de santé.

## Le partogramme : un cas parmi bien d’autres

Le partogramme est un outil simple et standardisé, qui est présenté comme fondamental dans la lutte contre la mortalité maternelle en Afrique. Toutes les écoles de santé des pays africains initient leurs élèves à l’usage du partogramme. De nombreuses formations à son sujet sont dispensées en outre aux sages-femmes déjà en activité.

Et pourtant, dans la majorité des cas, au Niger (mais aussi dans les pays voisins) le partogramme n’est pas rempli pendant le travail, mais après, souvent à la fin du service, en reportant des chiffres standards. Lorsqu’une sage-femme décide de référer, c’est en général sur la base de son seul jugement, et non sur la base d’un partogramme. Elle remplit alors celui-ci avant l’évacuation (il doit être joint au dossier), en prenant soin d’y indiquer de « bonnes données » (factices, mais standardisées) justifiant la référence.

Certes il existe des sages-femmes qui remplissent les partogrammes pendant le travail, et s’en servent comme guide. Mais elles sont, selon les multiples entretiens menés par les chercheurs du LASDEL auprès de sages-femmes, une très petite minorité. D’autres chercheurs, travaillant également avec des méthodes qualitatives et en Afrique de l’Ouest, ont noté que les partogrammes n’étaient pas remplis pendant le travail [31, 32]. La « norme pratique » (cf infra.) suivie par la plupart des sages-femmes est de ne remplir le partogramme qu’après l’accouchement.

Bien que tous les agents de santé de première ligne (et de nombreux fonctionnaires ou experts) soient au courant de cette situation, dans les colloques et séminaires, dans les publications officielles, dans les rapports des organisations internationales ou des ONG qui interviennent dans le domaine de l’accouchement, rien n’est dit sur les usages effectifs du partogramme dans les centres de santé et les maternités de premier niveau. Autrement dit, tout le monde parle et agit comme si les partogrammes étaient utilisés de façon fiable et routinière. Comme s’il n’y avait pas de problème quant aux usages du partogramme et comme si comme le « modèle » du partogramme était efficace.

Pourquoi les sages-femmes nigériennes, ou béninoises, ou maliennes, ne remplissent-elles pas les partogrammes pendant le travail ? Diverses explications ont été recueillies auprès des sages-femmes elles-mêmes lors de nos enquêtes : la surcharge de travail est la principale (« *avec plusieurs accouchements simultanés, le temps manque pour remplir le partogramme* » cette justification est couramment énoncée par les intéressées) : mais cette situation ne se présente fréquemment que dans de rares maternités, ce qui rend l’argument assez contestable dans la grande majorité des cas. Le primat accordé à l’expérience (et au ‘flair’ qui en découle) est un autre facteur explicatif, plus crédible. On peut aussi citer une faible culture de l’écriture chez les sages-femmes, une répugnance (« reluctance ») profonde face aux tâches « bureaucratiques », une éthique professionnelle souvent défaillante, une sous-traitance des accouchements aux matrones, aux stagiaires, ou aux filles de salle, et l’absence ou la non fonctionnalité des instruments (tensiomètre, thermomètre) nécessaires à la prise des variables [33–36]. Enfin, le fait que les partogrammes soient un outil d’évaluation lors des supervisions a un effet pervers ([27, 31] : ceci incite à écrire les « bonnes données », autrement dit des données standards, plutôt que les données réelles (qui pourraient signaler des défaillances de la sage-femme).

Toutes ces raisons renvoient, d’une façon ou d’une autre, aux contextes professionnels locaux, qui « contournent » le modèle proposé.

Le partogramme est un instrument qui relève en fait de l’ingénierie sociale (et non de l’ingénierie bio-médicale comme une molécule ou un vaccin), comme un très grand nombre des éléments qui constituent une politique nationale ou internationale de santé. Autrement dit, son usage et son efficacité dépendent essentiellement de la façon dont il est mis en oeuvre (ou non) par un système de santé et en particulier par les travailleurs de première ligne. Tout dépend, finalement, des sages-femmes, de leurs motivations, de leur compétence, de leur culture professionnelle, de leurs « normes pratiques ». D’autre part, bien d’autres acteurs interviennent dans la diffusion et dans la mise en oeuvre (ou non) du partogramme : les directrices de maternité (et le type de leadership qu’elles exercent), les équipes-cadres de districts sanitaires (et leur mode de supervision), les formateurs en partogrammes, la hiérarchie sanitaire du système de santé national, les organisations internationales et les ONG travaillant dans le domaine de la santé maternelle. Même si les sages-femmes sont les premières concernées, le non usage du partogramme pendant l’accouchement est la résultante des comportements de toutes ces catégories d’acteurs.

Pour les experts internationaux (et nationaux) qui l’ont mis au point et l’ont diffusé à travers le monde, le partogramme est pourtant un instrument très simple, à la portée de tout agent de santé, et utilisable dans des contextes peu équipés en matériels : c’est ce qui en fait sa valeur postulée pour les systèmes de santé fragiles des LMIC, par contraste avec le monitoring informatisé sophistiqué des salles d’accouchements des pays du Nord. Sa simplicité est en outre au service de plusieurs fonctions : suivi de l’accouchement, aide à la décision (référence), partage de l’information entre professionnels de santé, outil d’évaluation du personnel, et même document médico-légal en cas de décès. D’une certaine façon, il a tout du « mécanisme miracle » vu d’un bureau.

La standardisation du partogramme (les agents de santé suivent des formations ad-hoc, et reçoivent de leur ministère, ou de l’UNICEF, ou d’ONG internationales, des formulaires pré-imprimés) et son internationalisation (ces formations et ces formulaires sont à peu près identiques à travers le monde) font du partogramme un « modèle voyageur » typique, parmi tant d’autres. La santé maternelle ne manque pas de « modèles voyageurs » : ces dix dernières années ont vu ainsi l’arrivée en rafale au Niger de la prévention de la transmission du Sida de la mère à l’enfant (prevention of mother to child transmission of HIV (PMTCT) des soins obstétricaux et néo-nataux d’urgence (SONU), des soins obstétricaux et néo-nataux essentiels (SONE), de la gestion active de la troisième phase de l’accouchement (GATPA), des exemptions de paiement pour la césarienne, de la consultation prénatale recentrée (focused antenatal care, FANC ), de la prise en charge intégrée des maladies de l’enfant (PCIME), du paiement basé sur la performance (PBF). Certains de ces modèles sont, vu du terrain, plutôt des échecs (au Niger en tout cas), comme la PTME ou la FANC, les autres ont des résultats plus ou moins mitigés dont le bilan reste à faire. La gestion active de la troisième phase du modèle de travail, par exemple, est généralement considérée comme un succès, en grande partie parce qu’il s’agit d’un acte médical (injection d’ocytocine) où le rôle de l’ingénierie sociale est relativement faible. Cependant, même ce modèle souffre de plusieurs défauts dus aux contextes locaux, tels que des retards dans l’injection, des ruptures de stock, des interruptions de la chaîne du froid et des massages utérins peu fréquents (voir le programme Rational Pharmaceutical Management Plus [Gestion rationnelle des produits pharmaceutiques] de 2007, concernant le Bénin). Les goulots d’étranglement et les effets involontaires des modèles voyageurs dans le domaine de la santé restent souvent à documenter en profondeur, même si cela a été fait pour l’exemption des frais d’utilisation au Mali [37] et au Niger [38], et pour le PBF au Bénin [39].

La santé est un domaine où la standardisation et l’internationalisation des interventions, des politiques ou des procédures sont particulièrement développées. Cette standardisation internationale a certes des justifications économiques ou managériales, mais elle repose aussi sur une croyance en l’efficacité intrinsèque des « modèles voyageurs » dans la lutte contre les maladies (dont on peut trouver une des origines dans la lutte contre les grandes endémies) et sur une « protocolisation » croissante des soins [40, 41]. Elle est largement due au poids des grandes organisations internationales qui financent et diffusent ces modèles voyageurs (OMS, UNICEF, Fonds mondial, Fondation Bill Gates, etc.), mais les autorités sanitaires nationales ou les ONG médicales y contribuent aussi.

Et pourtant, comme pour le partogramme, la plupart des modèles voyageurs standardisés se heurtent à diverses formes de résistance des contextes dans lesquels ils sont implémentés. Ils ne « marchent pas » comme les experts l’ont prévu. Cela ne signifie certes pas qu’ils n’auraient pas d’effets positifs : il serait tout aussi absurde de dire qu’ils ne marchent jamais que de dire qu’ils marchent toujours. Mais le problème est que les outils d’enquête quantitative couramment utilisés en santé publique (comme les randomized control trials, RCT) ne permettent pas de mettre en évidence le processus de mise en oeuvre [19], les effets inattendus et les stratégies des intervenants face aux modèles voyageurs standardisés [42]. Bien que les méthodes expérimentales aient été remises en question, y compris dans le domaine des initiatives de maternité sans risques [43], la plupart des décideurs les considèrent toujours comme l’outil idéal pour des interventions fondées sur le rapport coût-efficacité. Seules des méthodes qualitatives rigoureuses peuvent décrire comment ces modèles sont reçus « dans le monde réel » : lorsqu’ils sont adoptés, c’est partiellement, parfois à reculons, parfois sous la contrainte ou sous pression, parfois de façon cosmétique, rarement avec enthousiasme ou conviction. Ils sont souvent contournés, détournés, démembrés, désarticulés.

## La fabrique d’un « modèle voyageur »

Le concept de « modèle » étant très polysémique, son usage ici doit être clairement spécifié. Nous ne parlons pas ici des modèles informatiques fondés sur des algorithmes (simulations ou projections, largement utilisées en santé publique et en économie de la santé). Nous nous intéressons à une tout autre catégorie de modèles, à savoir les programmes standardisés d’intervention (ici, en santé) visant à induire des changements de comportements, chez les agents de santé et/ou chez les patients.

Nous empruntons l’expression de « travelling model» à Rottenburg [44, 45], et nous la définissons comme toute intervention institutionnelle standardisée, quelles qu’en soient les échelles ou les domaines (une politique publique, un programme, une réforme, un projet, un protocole), en vue de produire un quelconque changement social, à travers des changements de comportements d’une ou plusieurs catégories d’acteurs, et qui repose sur un « mécanisme » et des « dispositifs » (cf. infra) censés avoir des propriétés intrinsèques permettant d’induire ce changement dans des contextes de mise en oeuvre variés. Dans le domaine de la santé maternelle, ces interventions visent à modifier les comportements des agents de santé et/ou des populations.

Les modèles voyageurs en vue de réduire la mortalité maternelle et néonatale sont donc des interventions standardisées, relevant de l’ingénierie sociale, qui peuvent être de formats très différents : depuis de simples protocoles (le partogramme, la consultation prénatale recentrée) jusqu’à des politiques de santé sectorielles (la prévention de la transmission du sida de la mère à l’enfant, les exemptions de paiement pour les accouchements ou les césariennes).

Le voyage des modèles est en science politique volontiers qualifié de « transfert » d’une politique publique. Dans le domaine du développement, qui n’est qu’une famille particulière de politiques publiques, élaborées et financées de l’extérieur, les modèles sont nombreux. Dans le champ spécifique de la santé, ils le sont encore plus. Ils se succèdent régulièrement et ils s’empilent les uns sur les autres, ils voyagent beaucoup, et ils voyagent loin.

A l’origine d’un modèle, on trouve toujours une expérience fondatrice (une histoire de réussite), quelque part dans le monde, dont des experts internationaux se saisissent pour la diffuser hors de son contexte d’origine. Le modèle est alors fabriqué autour d’un mécanisme causal qui est considéré par les experts comme explicatif du succès de l’expérience fondatrice. Ce mécanisme est censé garantir l’efficacité intrinsèque du modèle, quels que soient les nouveaux contextes dans lesquels il sera mis en oeuvre. Sa diffusion dans d’autres contextes sera assurée par des réseaux combinant experts et décideurs, appuyés sur des institutions internationales.

Autrement dit, la production spécifique d’un modèle voyageur passe par trois processus principaux, qui se chevauchent largement : la mise en récit (une histoire de réussite fondatrice), la mise en forme (la construction d’un mécanisme et de ses dispositifs), et la mise en réseau (la diffusion mondiale). Bien évidemment, ces processus prennent place dans un environnement que les analyses des politiques publiques ont déjà abondamment décrit, soit selon une perspective séquentielle (émergence, formulation, décision, mise en oeuvre [46, 47]; soit par la métaphore du « couplage » entre trois « courants » [48] : les problèmes, les solutions, les orientations politiques.

## Le cas du financement axé sur le rendement (PBF)

Prenons l’exemple du PBF ou « financement axé sur les résultats » (RBF) ou « rémunération au rendement », qui est promu à grands frais par la Banque mondiale dans tous les LMIC, en particulier dans le domaine de la santé [49, 50]. Il concerne directement la santé maternelle : les femmes enceintes et les enfants de moins de 5 ans sont les cibles privilégiées.

Le PBF, comme beaucoup de modèles voyageurs en santé, s’est appuyé sur une première histoire de réussite, en l’occurrence dans un pays développé, la Grande-Bretagne. La mise en récit de cette histoire de réussite à travers de nombreuses publications élogieuses ignore toutefois les réserves formulées ou les analyses critiques : en effet, plusieurs revues systématiques n’ont pas montré un impact positif significatif du PBF sur l’efficacité et la qualité du système de santé en Grande-Bretagne [51, 52]. Puis le PBF a été importé en Afrique par le biais d’une « histoire de réussite relais », le Rwanda en l’occurrence [53–57]. Le PBF rwandais, expérimenté en 2002 et généralisé en 2006, a été abondamment présenté comme un modèle pour l’Afrique, y compris dans une revue prestigieuse comme *The Lancet* [58]. Mais, dans le cas du Rwanda aussi, les analyses critiques ont été ignorées, comme l’étude de Kalk et coll. [55].

Un modèle voyageur, en effet, a besoin de se référer à une expérience certifiée exemplaire, quelque part dans le monde. C’est une étape indispensable pour sa production puis son exportation. Mais une expérience locale, avec son contexte, son originalité et ses spécificités, ne devient pas toute seule, par la seule vertu de sa réussite ou du bien qui en a été dit, un modèle exportable. Il faut d’abord extraire de cette expérience le « mécanisme » explicatif de son efficacité, et traduire ce mécanisme en dispositifs opérationnels. Le mécanisme du PBF est simple en son principe, il repose sur l’indexation du salaire d’un professionnel de santé sur sa performance (nombre d’actes et qualité des actes), autrement dit déterminer des indicateurs permettant à celui qui travaille plus et mieux de gagner plus. Le mécanisme du partogramme est lui aussi simple, il préconise de prendre manuellement des variables et de les reporter sur un diagramme pré-imprimé qui permet de définir des seuils d’alerte. Mais l’opérationnalisation d’un mécanisme est, quant à elle, toujours complexe. Il faut en effet traduire le mécanisme en « dispositifs » (devices) d’intervention, qui relèvent de l’ingénierie sociale et passent par de multiples « instruments » [59, 60]. Nous entendons par « dispositifs » les composantes organisationnelles fondamentales et les mesures opérationnelles, techniques, institutionnelles qui permettent de mettre en oeuvre un mécanisme au sein d’une intervention ou d’une politique publique de santé. Dans le cas du PBF, les dispositifs sont sophistiqués. Par exemple, au Burkina Faso, il y a 102 indicateurs d’évaluation de la performance des agents de santé que la coordination du programme PBF doit compiler et contrôler, sans compter les 61 indicateurs de l’évaluation de base financée par la Banque Mondiale. Pour les seuls centres de santé de premier échelon, pas moins de 23 indicateurs quantitatifs et 11 indicateurs qualitatifs ont été élaborés par les experts.

Un mécanisme n’apparait pas tel quel dans le cerveau des experts, il est élaboré par ces derniers à partir d’une opération de simplification et de « réduction » des expériences inaugurales. C’est ce qu’Ancelovici et Jenson [61] appellent la phase de « décontextualisation ». Dans les leçons tirées des histoires de réussite de référence, il faut procéder à la division d’une réalité particulièrement complexe en deux parties : d’un côté un « mécanisme », doté d’une efficacité intrinsèque, et de l’autre un « contexte », relégué au statut d’adjuvant. Cette distinction n’est pas spontanément donnée, elle a été construite. La complexité et la diversité des histoires de réussite initiales ont été « réduites » à l’efficacité supposée d’un « mécanisme » explicatif, qui serait donc exportable. On n’a pu « produire » le mécanisme du PBF qu’en l’extrayant de ce qu’on a « produit » simultanément comme étant son contexte d’émergence, que ce soit en Grande-Bretagne ou au Rwanda. Par exemple, dans le cas du Rwanda, de nombreux éléments contextuels ont été décisifs dans le « succès » de la mise en oeuvre du PBF, à savoir la situation politique très spécifique, le très fort contrôle social qui y règne, la capacité du régime à assurer un fonctionnement relativement satisfaisant des services publics, une corruption moins étendue et moins visible qu’ailleurs, l’usage effectif de sanctions au sein de l’administration et la crainte qu’elles suscitent. Ces éléments contrastent fortement avec la situation que connaissent la plupart des systèmes de santé des pays africains, y compris le « chacun-pour-soi-isme » et la faiblesse de l’État, le mauvais fonctionnement des services publics, la corruption ouverte et généralisée, l’impunité, entre autres [62]. La réduction d’une histoire de réussite à un mécanisme efficace implique la mise entre parenthèses de toute une série d’autres co-facteurs potentiellement explicatifs de cette histoire de réussite de référence, qui sont relégués à un statut d’éléments contextuels, qu’il n’est plus nécessaire de prendre en compte.

La réduction à un mécanisme va de pair avec la construction de celui-ci comme étant le coeur du nouveau modèle (comme on parle du coeur d’un réacteur). Un argumentaire de justification et de légitimation se met en place, qui est aussi un argumentaire de vente autour du mécanisme. Les histoires de réussite à l’origine du modèle voyageur doivent être validées, le mécanisme doit être certifié, célébré. Le PBF s’appuie ainsi sur une littérature considérable, sur une masse impressionnante d’évaluations, séminaires, ateliers, formations, voyages d’études, rapports, manuels, ce qui implique une main-d’oeuvre spécialisée importante. Des réseaux de plaidoyer, des réseaux d’experts, des réseaux de financements doivent se mettre en place. Pour le PBF, la Banque mondiale, OMS, Global Fund (GFATM) et diverses agences (en particulier la Norvège) ont collaboré, par exemple au sein de structures telles que Inter-Agency Working Groups on Results-Based Financing ou Health Results Innovation Trust Fund [55]. Dans ce processus de promotion d’un modèle voyageur, on ne prend pas en compte les évaluations négatives ou simplement réservées, mettant en évidence des effets inattendus, comme cette “overview of research on the effects of RBF” dans les LMIC [63] qui aboutissent à trois conclusions significatives, qui seront confirmées par diverses études ultérieures. Tout d’abord, “*There are few rigorous studies of RBF and overall the evidence of its effects is weak”* (especially in the long run); ensuite, *“The use of RBF in LMIC has commonly been as part of a package that may include increased funding, technical support, training, changes in management, and new information systems. It is not possible to disentangle the effects of RBF and there is very limited quantitative evidence of RBF per se having an effect*” et enfin, “*RBF can have undesirable effects, including motivating unintended behaviours, resulting in distortions (ignoring important tasks that are not rewarded with incentives), gaming (improving or cheating on reporting rather than improving performance), corruption, cherry-picking patients that make it easier to reach targets and earn bonuses and selecting out more difficult patients, widening the resource gap between rich and poor, dependency on financial incentives, demoralisation due to feelings of injustice, and bureaucratization.*” ([63], p. 4–5.) Alors que les deux premières conclusions renvoient à la difficulté d’isoler et d’évaluer l’efficacité intrinsèque d’un mécanisme, la dernière souligne les effets inattendus dus aux contextes de mise en oeuvre.

Ces phénomènes ne sont pas spécifiques à la santé : on les retrouve dans tous les domaines du développement, comme l’illustrent les cas du micro-crédit, fondé sur la popularisation de l’expérience de la Grameen Bank au Bengla Desh [64], ou des « transferts monétaires », fondé sur la popularisation des expériences de Bolsa Familia au Brésil et de Oportunidades au Mexique [65]. Ce dernier cas a fourni les arguments de base pour le cadre analytique développé ici.

## À propos du concept de « mécanisme »

La production d’un mécanisme correspond à une certaine « théorisation », mettant en évidence un enchainement causal censé être reproductible. Notre définition du mécanisme correspond pour une part à ce que certains spécialistes de l’évaluation des programmes (« évaluation basé sur une théorie » [66–68]) appellent « programme de théorie » ou « logique de l’intervention », autrement dit un « *set of hypotheses that explains how and why the intervention is expected to produce its effects* » [69]. Mais le mécanisme n’est pas fait que d’hypothèses (ou d’idées, de mots ou de discours), c’est aussi, ou surtout, un montage institutionnel et organisationnel autour de ces hypothèses, autrement dit un ensemble de « dispositifs » (devices). En évaluation, on parle des « composantes essentielles» d’un programme.

C’est l’ensemble « mécanisme + dispositifs » qui définit le coeur d’un modèle voyageur.

Notre définition de « mécanisme » se distingue donc du sens que « mécanisme » a pris récemment en sciences sociales, à la suite de Elster [70] et de Hedström & Swedberg [71, 72], où il désigne un processus causal « réel », mais plus ou moins caché, tel qu’il est déduit/dévoilé/construit par les sciences sociales [73]. Pour notre part, un mécanisme n’est pas un processus causal réel, mais caché, mais plutôt un processus causal présumé explicitement postulé par un modèle d’intervention standardisé.

En associant un mécanisme et un modèle d’intervention, nous nous rapprochons évidemment des approches dites « réalistes » de l’évaluation [74], qui insistent à juste titre sur le fait que les effets (outcomes : O) d’une intervention (d’une politique publique, d’un programme) dépendent des interactions entre le contexte de l’intervention (C) et le mécanisme de l’intervention (M). Elles s’intéressent en conséquence à la configuration « CMO ». Nous partageons leur souci de considérer le contexte comme un élément décisif de toute mise en oeuvre d’un programme ou d’une politique publique (et donc d’un modèle voyageur). Mais il y a un flou important chez les auteurs qui se réclament de cette approche quant au contenu exact du « mécanisme » et quant à la différenciation conceptuelle ou empirique entre le mécanisme et le contexte [16, 42, 69, 75–77]. Selon la définition la plus commune, à la suite de Pawson and Tilley [74], le mécanisme d’une intervention serait constitué des «*idées et des réactions » des acteurs concernés par l’intervention* » [69], proposition qui reste pour le moins énigmatique. Pour nous, cette confusion autour de ce que serait un mécanisme est liée au choix de faire du mécanisme de l’intervention une causalité « réelle », mais cachée, reposant sur les réactions des acteurs à l’intervention, et qui serait, à côté du contexte et à travers lui, à l’origine des effets de l’intervention (outcomes). Lacouture & al. [69], après avoir passé en revue la variété des usages du concept de mécanisme dans les approches réalistes de l’évaluation, proposent une définition qui ne dissipe guère la confusion régnante : « *A mechanism is an element of reasoning and reactions of (an) individual or collective agent(s) in regard of the resources available in a given context to bring about changes through the implementation of an intervention.*» Cette acception oblige à distinguer d’une part l’intervention proprement dite, et d’autre part le mécanisme explicatif qui produirait des effets « réels », mécanisme qu’il incomberait au chercheur de révéler (mais qui reste de fait une « boîte noire »). Elle oblige aussi à mettre les acteurs du côté du mécanisme, et non du côté du contexte, ce qui est difficilement soutenable. Il me semble beaucoup plus simple de ne pas s’aventurer sur ce terrain et de considérer le mécanisme comme étant la « théorie opérationnelle » de l’intervention, mise en pratique à travers les dispositifs. Symétriquement, les perceptions et réactions des acteurs impliqués ou ciblés par la mise en oeuvre d’un modèle voyageur doivent être considérées comme étant des éléments centraux des contextes de mise en oeuvre.

La distinction entre un mécanisme et un contexte n’est donc pas à nos yeux une opération scientifique effectuée par un chercheur a posteriori, c’est une construction sociale délibérée au coeur de la production des modèles voyageurs, selon laquelle des experts font un tri entre ce qui relèverait d’une variable explicative (le mécanisme), qui est leur affaire, et autour duquel ils construisent leurs interventions (grâce aux dispositifs), et ce qui relèverait de variables contextuelles, dont la gestion reste toujours problématique et incertaine. La construction d’un mécanisme est indissociable du langage des variables (et, au sein de ce langage, de la promotion d’une « variable explicative »).

## Les voyages du modèle et la revanche des contextes

Le mécanisme et ses dispositifs sont ensuite déployés dans des contextes nouveaux. Pour cela, ils doivent passer par des réseaux informels et formels qui vont permettre le voyage du modèle, en le justifiant, le légitimant scientifiquement, le promouvant. La sociologie des sciences et techniques promue par Callon et Latour [78], sous le nom de sociologie de la traduction ou de la théorie acteur-réseau s’est particulièrement focalisée sur le rôle de ces réseaux tels que la constitution d’alliances, l’« enrôlement » de soutiens, etc. Cette réplication d’un modèle « loin de la base d’origine », et donc loin de l’histoire de réussite de départ, n’a rien de spontanée. Elle est basée sur une alliance (une « coalition ») d’experts et de décideurs, qui se font les « porteurs sociaux » de ce qu’on pourrait appeler « l’idée » du modèle voyageur (son mécanisme), son récit, sa promotion, jusqu’au point où son importation est mise sur agenda dans divers pays lointains, en général grâce à la conjonction de financements et/ou d’assistances techniques internationales.

Les institutions internationales jouent en effet un rôle central, en particulier dans le domaine de la santé, en tant qu’« agences de voyages » pour les modèles. Mais elles ne sont pas les seules : Bureaux d’études, think tanks, « communautés de pratiques » et ONG médicales interviennent aussi. Toutes ces institutions justifient leurs interventions au nom de l’efficacité intrinsèque du modèle qu’elles promeuvent, et tendent à ignorer ou minimiser les effets inattendus de sa mise en oeuvre dans des contextes peu connus du monde des experts et des décideurs.

## L’épreuve des contextes. Le cas des consultations prénatales

Le concept d’« épreuve » a pris une place importante dans de récents travaux francophones en sciences sociales, entre sociologie pragmatique et sociologie des sciences et techniques [79–81]. Pourtant, l’« épreuve » de la mise en oeuvre d’un modèle voyageur dans des contextes éloignés de l’histoire de réussite d’origine s’avère redoutable. Contrairement à l’idée reçue dans le monde des experts en construction et diffusion de modèles, l’efficacité intrinsèque d’un modèle compte moins que les contextes de mise en oeuvre, et plus particulièrement les routines et contraintes des personnels de santé qui sont au contact des usagers. Ce sont ces travailleurs de première ligne qui vont « tester » le modèle proposé (et, en général, imposé par la hiérarchie sanitaire), l’adapter à leurs conditions de travail et à leur culture professionnelle, et l’ « accommoder » à leur manière, souvent en le contournant, le détournant ou le démantelant, parfois en l’ignorant ou en le boycottant de facto.

Prenons un nouvel exemple. Les consultations pré-natales (antenatal care, ANC) effectuées dans les centres de santé de premier niveau par les sages-femmes dans les pays d’Afrique de l’Ouest, inspirées d’un modèle européen ancien du début du 20 siècle, sont considérées comme un élément clé de la lutte contre la mortalité maternelle. Mais le modèle classique tel qu’il est mis en oeuvre depuis longtemps au Niger et dans les pays voisins souffre de diverses insuffisances et lacunes. Expéditives et bâclées, les ANC ne remplissent pas leur fonction principale, qui est permettre de détecter les grossesses à risques, afin de les prévenir ou de prévoir un accouchement dans une formation sanitaire équipée pour les accouchements dystociques. Par exemple, une étude précédente sur 330 femmes enceintes a conclu qu’«*au Niger, la qualité du dépistage [par les sages-femmes] des facteurs de risque était médiocre* » [82]. Un nouveau modèle voyageur d’ANC a donc été adopté par l’OMS au début des années 2000 [83], et a été diffusé largement en Afrique : la consultation prénatale recentrée (focused antenatal care, FANC). La FANC repose en particulier sur un formulaire pré-imprimé, qui comporte toute une série d’items à renseigner et à cocher scrupuleusement, permettant ainsi à une sage-femme, en suivant à la lettre l’imprimé, de procéder à toute la batterie d’opérations requises pour une FANC efficace et personnalisée (observations, examens, questions, explications). Sur la base des réponses à une première série de questions et d’observations, il y a bifurcation entre deux types de dossiers, l’un pour les grossesses sans risques spécifiques (basic components) qui seront suivies selon un protocole standard, l’autre pour les grossesses à risque (specialized components) qui bénéficieront d’un suivi particulier. C’est donc un mécanisme dont le principe est le même que celui du partogramme (un auto-monitorage guidé par des supports à remplir par écrit), mais qui repose sur des dispositifs plus complexes (items beaucoup plus nombreux, examens biologiques, dialogue soutenu avec les parturientes, deux types de dossiers, etc.). L’argumentaire de promotion est du même type que pour le partogramme – c’est un outil simple adapté aux conditions frustes des LMIC : « *FANG is the best approach for resource-limited countries where health professionals are few and health infrastructures are limited.* » [84] Mais une fois encore les contextes de mise en oeuvre ont démenti le scénario des experts.

Le problème de la gestion du temps s’est posé très vite, dès l’arrivée des FANC, au Niger comme dans d’autres pays africains [85, 86]. En effet, 40 minutes (selon l’OMS) est le temps minimal permettant de remplir tous les items du formulaire lors de la première visite. Or les ANC, qui duraient jusque-là en moyenne moins de 15 minutes, étaient déjà particulièrement suivies par les femmes enceintes, et les queues se sont donc allongées, d’autant plus que, dans la plupart des formations sanitaires nigériennes, seulement deux matinées y sont consacrées, souvent avec une seule sage-femme dédiée à cette activité, ce qui pose évidemment le problème de l’organisation routinière des activités dans les centres de santé de niveau primaire. On peut ajouter la forte répugnance et le malaise des sages-femmes face aux tâches bureaucratiques et à l’écriture. Dans de telles conditions, passer d’une dizaine de minutes de CPN à 45 minutes de FANC est mission impossible. La plupart des sages-femmes ne font donc pas une véritable FANC, sauf lors du passage d’une équipe de supervision. Elles ne documentent pas de nombreux items, et/ou remplissent les fiches de façon standardisée. Certaines constantes sont rarement prises (comme la tension artérielle, la tachycardie ventriculaire ), le spéculum n’est pas utilisé (il est en général absent dans les maternités). Le plan d’accouchement et les complications possibles ne sont pas discutés.

La méconnaissance du problème que pose la durée d’une FANC, mais aussi la méconnaissance de l’organisation du travail dans les CSI et les maternités, la méconnaissance de la culture professionnelle réelle des sages-femmes, sont des causes directes de l’échec (au moins partiel) de la FANC. L’information sur la non délivrance effective de la FANC « remonte » rarement à travers les systèmes de supervision et de rapportage du Ministère de la santé et des programmes verticaux mis en place par les institutions internationales, et ne donne pas lieu à des rectifications. Personne, au sein de la hiérarchie sanitaire, ne pose le problème en public, même s’il est connu en privé. Les statistiques élogieuses qui circulent sur la FANC concernent principalement les formations (nombre de sessions de formation, nombre d’agents de santé formés, nombre de suivis post-formation), les activités de FANC déclarées réalisées (nombre de FANC effectuées, taux de couverture) ou les aspects logistiques et financiers (nombre de formulaires diffusés, taux d’exécution budgétaire). Elles ne concernent pas la qualité des FANC censées avoir été réalisées.

## À propos du concept de « contexte » : les contextes pragmatiques et le rôle des acteurs

Les modèles de santé maternelle introduits dans les LMIC « oublient » donc l’existence massive, au sein des systèmes de santé, de toute une série d’éléments liés aux comportements des acteurs et aux contextes locaux. Cependant, ces éléments ont été soulignés dans certains travaux, notamment les stratégies de captation de l’aide [87], la corruption [88, 89], le clientélisme [90], les routines professionnelles locales, l’absentéisme au travail, les gains informels [91, 92], les types de leadership, de gestion et d’organisation du travail [93], l’interventionnisme en matière de posting and transfer [94, 95], la fabrique opportuniste des chiffres ou des statistiques biaisées [96, 97], entre autres. De manière plus générale, comme le soulignent Greenhalgh et coll. [98] : « *the important principle that the attributes [of an innovation implemented in health services] are neither stable features of the innovation nor sure determinants of their adoption or assimilation. Rather it is the interaction among the innovation, the intended adopters and a particular context that determines the adoption rate.* ».

Il est vrai que, au sein de la santé publique, les contextes ne sont pas inconnus. Mais ils sont réduits à un ensemble de variables dites contextuelles de type socio-démographique ou épidémiologiques (taux, indices et autres indicateurs, relatifs aux revenus, à la couverture sanitaire, à l’accès aux soins, au seuil de pauvreté, à la scolarité, à la fréquentation des services publics, au PIB, à l’IDH, etc…), qui doivent être quantifiées et peuvent être agrégées, pondérées, énumérées dans d’innombrables variations. Par exemple, dans une revue de la littérature consacrée au rôle des contextes dans les programmes d’amélioration de la qualité des soins [99], les auteurs, après avoir distingué quelques familles de variables contextuelles telles que les environnements externes (les environnements économiques, sociaux et politiques), l’environnement organisationnel, les individus et leurs rôles et les réseaux, distinguent soixante-six variables ayant fait l’objet de mesures. Cette logique des variables, sa dérive inflationniste, et l’obsession quantitativiste (aucune étude qualitative n’a été prise en compte dans cette revue) enlèvent dès lors tout son sens au concept de contexte tel que nous l’entendons. Elle aboutit à ignorer ou minimiser ce qui constitue l’élément décisif des contextes, à savoir les acteurs. Comme l’a souligné Abbott [100] : « *In the causal modeling tradition, variables and not actors do the acting* ». (Cité dans Hedström and Swedberg [72]). Il faut au contraire placer l’acteur au centre des contextes (et non en faire une variable parmi d’autres).

Certes, le concept de contexte peut paraître lui-même ambigu, et polysémique. Tout d’abord, dans la perspective des études scientifiques, toute action, à quelque niveau que ce soit, se déroule dans un contexte, chaque contexte étant spécifique. La construction d’un mécanisme ou la diffusion mondiale d’un modèle se font aussi dans des contextes, et les bureaux de la Banque mondiale ou ceux d’un quelconque ministère de la santé sont tout autant des contextes qu’une maternité rurale ou une consultation hospitalière. Les uns et les autres méritent l’attention des chercheurs. Toutefois, on peut admettre qu’il s’agit de contextes très différents : ceux des think tanks ou des réunions d’experts dits « de haut niveau » (contextes de mise en récit, de mise en forme ou de mise en réseau d’une politique publique) ne sont pas ceux des quartiers pauvres de Dakar ou des villages du Niger (contextes de mise en oeuvre). Dans le cadre du présent article, nous réservons le terme de « contexte » aux seuls contextes de mise en oeuvre (nationaux, régionaux, locaux) d’un modèle voyageur.

Nous proposons donc une vision plus différenciée des contextes de mise en oeuvre, en distinguant d’une part les contextes structurels (en arrière-plan, et le plus souvent décrits par le langage des variables) et d’autre part les contextes pragmatiques (au premier plan, et relevant plutôt des approches qualitatives). Au centre des contextes pragmatiques se trouve le rôle des acteurs, dont entend rendre compte le concept, aujourd’hui largement utilisé en sciences sociales, d’agence développé par Giddens [101] et importé dans le champ du développement par Long [102], autrement dit de « marge de manoeuvre » cognitive et stratégique des acteurs (leur capacité à connaître et agir de façon relativement autonome), et les normes et contraintes au sein desquelles elle s’exerce [103]. Les contextes structurels (en général caractérisés comme étant liés à l’environnement économique, politique, social ou culturel) n’agissent sur l’importation d’un modèle voyageur que par l’intermédiaire des contextes pragmatiques, autrement dit du fait du jeu des acteurs et de leurs interactions. Les contextes structurels sont autant de contraintes et de ressources disponibles qui interviennent pour influencer les représentations et pratiques des groupes stratégiques impliqués par les modèles voyageurs en santé publique (experts et décideurs politiques nationaux, professionnels de santé publics et privés, ONG, autorités locales, bénéficiaires, non-bénéficiaires), qui constituent les contextes pragmatiques et sont au coeur de ce que nous appelons la revanche des contextes.

Les contextes structurels sont généralement décrits par le langage des variables, des chiffres et des indicateurs quantitatifs standardisés. Inversement, les contextes pragmatiques sont décrits plus précisément par des approches qualitatives telles que les récits, l’analyse de processus, les études de cas ou les entretiens avec les parties prenantes, et l’utilisation de concepts tels que la logique des acteurs, les perceptions des acteurs, les normes pratiques, les cultures professionnelles, les cultures locales, les groupes stratégiques et bien d’autres encore.

Autrement dit, les contextes de mise en oeuvre, ce sont d’abord et avant tout les acteurs locaux : leurs réseaux et leurs interactions, leurs normes sociales et leurs normes pratiques, leurs cultures organisationnelles ou professionnelles, leurs routines et leurs motivations. Cette perspective s’oppose clairement au langage des variables. Mettre les comportements des acteurs au centre de l’analyse des contextes implique de nouveaux outils conceptuels.

## Écarts et normes pratiques

Ce rôle central des acteurs, de leurs perceptions et de leurs stratégies, toujours éloignées des attentes ou des présupposés des experts, introduit en effet deux types de gaps, soit un écart entre la réalité et les objectifs et un normatif gap.

### Écart entre la réalité et les objectifs

L’écart entre la réalité et les objectifs est un processus inévitable : aussi « bien préparées » que soient les politiques publiques elles connaissent inévitablement au cours de leur mise en oeuvre des écarts entre ce qui a été prévu et ce qui se passe effectivement sur le terrain, autrement dit des « *differences between their formal objectives and goals and those that emerge through the practices and strategies pursued by the actors at different organisational levels*.*.. The relation of policy and practice is not an instrumental or scripted translation of ideas into reality but a messy free-for-all in which processes are often uncontrollable and results uncertain*” [104]. Ce titre éloquent “Good on paper: the gap between programme theory and real-world context”, qui introduit une analyse révélatrice de l’écart entre la réalité et les objectifs d’un programme de sages-femmes communautaires au Pakistan [22], pourrait être appliqué à tous les modèles voyageurs de la santé publique.

### Écart normatif

Nous appelons écart normatif, l’écart entre les règles et procédures incorporées dans un modèle voyageur et les règles et procédures en vigueur dans un contexte professionnel et social spécifique. Toute intervention standardisée en santé publique inclut dans ses dispositifs un ensemble de normes nouvelles, élaborées par des experts internationaux, et parfois aussi nationaux, pour rendre efficient le mécanisme de l’intervention. Mais ces normes importées sont par définition distinctes des normes qui régulent de fait les comportements des acteurs concernés par l’intervention.

En fait, on peut distinguer au moins deux grands types de registres normatifs au sein des contextes de mise en oeuvre des interventions en santé, à savoir les normes sociales des usagers (patients, femmes enceintes, communautés) et les normes pratiques du personnel de santé (infirmières, sages-femmes, docteurs).

**Normes sociales des patients** Les normes sociales des populations bénéficiaires sont très complexes et diverses et ne peuvent en aucun cas être réduites à quelques stéréotypes. La santé publique a en effet parfois la tentation de réduire les cultures locales non occidentales à quelques clichés « traditionnels » plus ou moins exotiques (autrement dit une dérive « culturaliste » [105, 106]. En particulier, les normes sociales sont liées à la maladie, à la souffrance, à la quête de soins, à la pudeur, à la bienséance, à l’entraide, à l’action collective, aux relations de genre, aux relations patron-client, à l’éducation, au surnaturel, à la mort, aux rapports à l’État, etc. Ces normes sociales, ces cultures locales, très variables selon les sites, sont dans les faits sous-estimées par les experts en modèles voyageurs (qui peuvent toutefois, sur un plan rhétorique, en reconnaitre l’existence).

**Normes pratiques des agents de santé**, mais il y a un autre type de normes contextuelles, encore plus ignorées des interventions standardisées, celles qui régulent les écarts entre les normes officielles et les pratiques routinières des agents de santé. Ces derniers, en effet, sont loin de suivre toujours les règles professionnelles et déontologiques auxquelles ils ont été formés. Ces comportements « non observants » ne sont pas pour autant aléatoires, ils sont relativement prévisibles et routiniers, et suivent en fait des normes latentes et implicites que nous appelons « normes pratiques ». Ce concept exploratoire, très pragmatique, se distingue d’autres tentatives intéressantes d’analyse des comportements non observants des personnels de santé face aux interventions bureaucratiques et aux directives cliniques, y compris dans les pays du Nord. Par exemple, en s’appuyant sur les données du Royaume-Uni, Checkland, Harrison & Marshall [107] insistent comme nous sur l’importance des contextes, et l’impasse que représentent l’approche dominante de la médecine fondée sur les données probantes, basée sur le repérage des obstacles aux changements, la diffusion des directives cliniques et les essais randomisés (voir aussi Greenhalgh et coll. [98]), mais, par contre, ils mettent prématurément au premier plan les concepts explicatifs d’identité collective et de raisonnement, importés de la psychologie sociale. Ceux-ci pourtant, selon notre expérience au Niger, sont loin d’être prédominants pour rendre compte des écarts de comportement des agents de santé.

Le concept de « normes pratiques » nous semble particulièrement utile pour analyser empiriquement comment les comportements « non observants » des personnels de santé sont régulés, en particulier dans leurs écarts aux injonctions des modèles voyageurs. En effet, on ne peut se contenter d’invoquer les contextes face aux interventions standardisées, encore faut-il avoir des outils pour analyser ces contextes.

Nous partons de ce premier constat, maintes fois réitéré dans nos enquêtes, à savoir que les comportements des personnels de santé, au Niger comme en Afrique francophone, sont loin de respecter les normes officielles, celles du Ministère de la santé comme celles introduites sans cesse par les modèles voyageurs. Pourtant ils ont été formés à l’observance de ces normes, et régulièrement recyclés via des formations à coûts élevés. Il ne s’agit donc pas d’ignorance.

Un second constat s’impose, à savoir que leurs comportements « non observants » ne sont pas pour autant aléatoires ou anarchiques. Ils sont largement prévisibles pour qui connait le contexte professionnel local (collègues ou usagers). Ils forment la trame de la culture professionnelle locale [108]. Autrement dit ces comportements sont partagés, convergents (au-delà, bien sûr de diverses, exceptions). Ils sont régulés de façon latente, souterraine, tacite, dans leurs écarts aux normes prescrites.

Nous appelons « normes pratiques » ces régulations implicites. Ce concept a déjà été utilisé pour décrire les comportements routiniers des sages-femmes au Sénégal [109] ou au Niger [108], pour étudier le fonctionnement des urgences à Niamey [110] ou d’un service hospitalier à Soweto [111]. Pour une présentation théorique détaillée du concept de normes pratiques et de ses usages possibles, cf. Olivier de Sardan [112] et De Herdt et coll. [113]. S’intéresser aux normes pratiques des agents de la santé, c’est poser autrement la question de leurs cultures professionnelles – une culture professionnelle n’est pas simplement un ensemble de connaissances et de « bonnes pratiques » apprises au cours d’une formation, c’est aussi un ensemble de « trucs » appris sur le terrain pour agir au quotidien en s’écartant de ces connaissances et de ces bonnes pratiques. Il s’agit d’une combinaison de normes officielles et pratiques.

### La culture professionnelle des sages-femmes

Prenons la profession de sage-femme au Niger – toute une série de normes pratiques la caractérise. Voici une liste, sans ordre particulier, de toutes les normes pratiques confirmées par les professionnels de santé réunis à l’occasion de l’École professionnelle du LASDEL en direction du personnel de santé maternelle tenue à Niamey en octobre 2015. Les formulations sont de notre responsabilité (elles ont en effet pour caractéristique de ne jamais être exprimées publiquement comme telles et de rester le plus souvent cantonnée dans l’implicite), mais nous avons rencontré de façon répétée chacune d’entre elles sur nos terrains d’enquête au Niger depuis 15 ans, à travers de multiples observations, études de cas et entretiens privés.

- une sage-femme est plus compétente en matière d’accouchement qu’un médecin de santé publique et sait mieux ce qu’il faut faire
- la gestion d’un accouchement est une question d’expérience et de jugement
- la maternité est un espace qui « appartient » aux sages-femmes, pas aux parturientes
- les accouchements eutociques (sans problèmes) peuvent être délégués aux matrones, stagiaires, aides-sages-femmes ou filles de salles
- les femmes de brousse et les jeunes parturientes sont ignorantes et impatientes
- les femmes qui sont en « faux travail » (dilatation insuffisante) dérangent le service et il faut les renvoyer chez elles si possible
- quand une femme en travail ne « pousse » pas assez, il faut l’obliger à le faire par tous les moyens (insultes, rudoiements, ou menace de référer sont légitimes : c’est pour son bien)
- une parturiente « recommandée » mérite de l’attention et des égards qu’on n’a pas besoin d’accorder aux parturientes anonymes
- les tâches bureaucratiques sont des corvées inutilement chronophages
- sanctionner une sage-femme ne se fait pas, ce serait faire preuve de méchanceté
- il est normal d’arriver vers 9 heures au travail et d’en partir vers 13 heures
- il est normal de ne pas venir au travail pour aller à une cérémonie sociale (baptême, mariage ou décès d’un proche, mais aussi d’une simple connaissance)
- la recherche de gains « informels » aux dépens des parturientes (ventes de produits, tarification d’actes gratuits, etc.) est normale
- il faut partir aussi souvent que possible en formation continue indépendamment du contenu (pour recevoir des per-diem et améliorer son CV)
- pour obtenir des affectations intéressantes il faut recourir à des interventions de connaissances haut placées
- toute tâche supplémentaire doit faire l’objet d’une prime
- tout financement venant des bailleurs de fonds est une occasion de « prendre sa part » au passage
- chacun doit gérer ses activités sans se mêler de celles des autres
- les supports bureaucratiques doivent être remplis avec les données standards, peu importe les données réelles
- les réunions d’équipes (staff) sont des pertes de temps
- dans ces réunions, on ne doit pas pointer les défaillances individuelles
- si du petit matériel manque, on attend que la hiérarchie le remplace
- face aux ruptures récurrentes de certains produits, il faut se « débrouiller » pour fabriquer si possible un ersatz. Par exemple, les sages-femmes utilisent du sérum salé à la place du solvant pour faire le test PTME ou font un collyre pour les nouveau-nés avec un mélange de bétadine et de sérum salé (50 % de chaque solution).

Ces normes pratiques ne sont bien sûr jamais énoncées publiquement en tant que telles, contrairement aux normes officielles (explicites dans les règlements, les procédures et les formations) et aux normes sociales (explicites dans l’éducation familiale ou religieuse et les interactions sociales). Mais elles sont au coeur des routines des maternités nigériennes. Elles constituent le socle, plus ou moins caché, de la culture professionnelle des sages-femmes et des agents obstétricaux. Elles régulent la réaction des sages-femmes face à un modèle voyageur et expliquent le plus souvent l’échec, partiel ou total, de ce dernier.

Seuls quelques écrits en anthropologie de la santé (parce qu’ils ont recours aux méthodes qualitatives, à l’observation prolongée, à l’insertion dans le milieu), ont documenté empiriquement les comportements quotidiens « réels » ou « non observants » des sages-femmes, dans le cas du Niger ou pour d’autres pays d’Afrique de l’Ouest [27, 28, 31–35, 108, 109, 114–119]. Sinon, aucun chiffre, aucune statistique n’en fait état. On n’en trouve très peu ou pas de traces dans les rapports de supervision. Les plans annuels d’activités des districts et les plans de développement sanitaire nationaux n’en parlent jamais dans les parties consacrées à la lutte contre la mortalité maternelle. Et il est rare qu’elles soient évoquées dans des écrits de santé publiques (et, en ce cas, elles le sont sommairement, partiellement et de façon euphémisée). Autrement dit, les maternités dont on parle dans le monde de la santé publique sont pour une grande part des maternités fictives, des maternités de papier, fort éloignées des réalités vécues par les parturientes. Les modèles voyageurs s’adressent à ces maternités de papier, et c’est sur leur image qu’ils sont construits.

## Pourquoi cette récurrence étonnante des modèles voyageurs ? La gouvernance de la santé mondiale

Certes, on trouve jusqu’au sein même des grandes organisations internationales, pourtant grandes productrices de modèles voyageurs, divers propos mettant en garde contre les régulations standardisées et la sous-estimation des contextes, comme dans cette publication de l’OMS “*Considerable caution is needed in scrutinizing formal management structures, rules and regulations, as they may bear no resemblance with actual management practice*” [120]. Plus largement, la standardisation des politiques de développement a déjà, et depuis longtemps, fait l’objet de diverses critiques émanant des sciences sociales, que ce soit dans le champ de l’économie politique, depuis Hirschman [121], qui fait figure de pionnier, jusqu’à Naudet [122] et Easterly [123], ou dans le champ de l’anthropologie du développement avec de nombreux auteurs [124–127].

Mais, loin de perdre en nombre ou en volume, les modèles voyageurs prospèrent de plus belle, sous des noms divers. La forme de gouvernance propre à la santé mondiale (poids des organisations internationales, des fondations privées, des laboratoires et des ONG) privilégie les « interventions à haut facteur d’impact ». Les sciences sociales ont donc bien peu d’influence en la matière. On a même parfois le sentiment que toute critique d’un modèle voyageur ne fait que créer les conditions pour qu’un autre modèle prenne la place, « *Better theory, new paradigms and alternative frameworks are constantly needed..., strangely little attention is given to the relationship between these models and the practices and events that they are expected to generate or legitimise in particular contexts* » [124].

Quels peuvent être les raisons qui expliquent une telle persévérance dans la production incessante de modèles voyageurs ? Nous en évoquerons ici cinq.

### Un marché de l’emploi pour experts

« *En dépit de l’échec de ces transferts systématiques de modèles du Nord vers l’Afrique depuis les indépendances, ceux-ci continuent puisque experts et consultants du Nord et du Sud, professionnels du Sud et leaders du Nord et du Sud en tirent des avantages* » [128]. La production et la circulation des modèles voyageurs sont en quelque sorte le « coeur de métier » des institutions internationales de santé publique et font travailler des cohortes de personnels, depuis la conception (fonctionnaires, chercheurs et experts internationaux) jusqu’à la mise en oeuvre de terrain (consultants internationaux et nationaux, formateurs, agents d’ONG, personnels de santé). Chaque nouveau modèle a son propre marché de spécialistes et de praticiens, à l’échelle nationale comme internationale.

### Des routines institutionnelles

On ne peut non plus sous-estimer le poids de la dépendance au chemin parcouru. Les bureaux d’études, les départements de santé publique, les agences de développement, les organisations internationales, les grandes fondations, les grandes ONG médicales, ont développé depuis plusieurs décennies des savoir-faire et des procédures techniques et comptables qui relèvent pour l’essentiel de la fabrication et de la diffusion de modèles voyageurs : mise en scène et en récit d’histoire de réussite, habillage et légitimation de mécanismes, conception de dispositifs, procédures, séminaires et guides de bonnes pratiques… On peut y ajouter la conception et l’usage d’instruments standardisés de planification (cadre logique) et d’évaluation (essais randomisés) qui sont particulièrement adaptés aux modèles voyageurs [43] en ce qu’ils se focalisent sur l’efficience des mécanismes et qu’ils « neutralisent » systématiquement le rôle des contextes. La fabrication et la diffusion de modèles voyageurs sont devenues des pratiques routinières pour de nombreuses institutions. On rejoint ici ce que Di Maggio et Powell [129] ont appelé l’isomorphisme des organisations, qui, dans un champ donné, tendent à se comporter de façon identique. Les modèles voyageurs ont également une affinité structurelle avec les approches de « commandement et contrôle », les stratégies de mise à l’échelle et les politiques (statistiques) fondées sur des données probantes, qui sont au coeur des cultures organisationnelles et des compétences professionnelles des institutions de santé publique.

### Le pilotage par l’offre des interventions en santé

Les politiques publiques dans le domaine du développement répondent assez généralement à l’idéal-type du type « corbeille » [130]. Comme noté par Naudet [122] à propos des politiques et programmes de développement, il s’agit de « trouver des problèmes pour les solutions ». Cette forte « politique de l’offre », qui s’appuie sur les modèles mis au point par les institutions internationales, fait face à une faible (et parfois inexistante) « politique de la demande » émanant des autorités du Sud, le plus souvent « suivistes » et conformistes, préoccupées avant tout par les ressources (la rente) que les intervenants extérieurs lui fourniront, et peu soucieuses de mettre au premier plan les préoccupations contextuelles qu’elles devraient théoriquement porter. La rhétorique des « bonnes pratiques » l’illustre : il s’agit pour l’essentiel des pratiques conformes aux normes promues par les modèles voyageurs, dans une perspective de « bon élève ». C’est un des effets pervers de la dépendance à l’aide (ou « rente du développement » [131]. Aucune pression, ou presque, du côté de la demande ne vient contrebalancer ou modifier l’offre incessante et pressante de modèles voyageurs. A cet égard, on ne peut faire porter la responsabilité de l’hégémonie des modèles voyageurs aux seuls experts et bailleurs de fonds internationaux. Les gouvernements et les experts des pays du Sud en sont largement co-responsables.

### Les logiques de financement

Il est beaucoup plus difficile d’obtenir des financements modestes pour des expériences innovantes à partir des contextes locaux, que de gros financements pour des modèles voyageurs : tous les professionnels de santé publique (et tous les professionnels du développement) le disent. Le montage et le format des dossiers, les mécanismes de financement, les procédures de décaissement, les modes de gestion, les pratiques d’audit : tout concourt à privilégier les mécanismes et dispositifs exportables, qui permettent de réaliser d’importantes économies et sont évaluables par des critères quantitatifs.

### Un logiciel bio-médical enraciné dans les mentalités

La croyance – car après tout elle en est une – en l’efficacité intrinsèque d’un mécanisme placé au centre d’une politique publique de santé repose sur une base expérimentaliste et techniciste qui, dans certains domaines précis, a fait ses preuves : en effet, une nouvelle molécule est porteuse d’une telle efficacité intrinsèque, à vocation universelle, et – d’un point de vue purement thérapeutique – peut s’appliquer dans les contextes les plus divers. Il est donc tentant de croire qu’il en est de même des mécanismes placés au centre des technologies sociales ou institutionnelles, et de créditer ces dernières de la même efficacité potentielle que celles des technologies bio-médicales (ou industrielles). Les essais randomisés reposent sur une telle extrapolation.

C’est un présupposé très répandu que de croire que ce qui est vrai de l’ingénierie biomédicale serait aussi vrai de l’ingénierie sociale. Rien n’est plus faux. Le passage de l’ingénierie biomédicale à l’ingénierie sociale est un changement radical de « monde de référence ». Dans le monde social, l’énoncé expérimentaliste classique « toutes choses égales par ailleurs » n’a pas de sens; sa mise en oeuvre est impossible [132, 133]. La multiplicité, la complexité et l’imbrication des variables impliquées par toute intervention sociale, aussi minime soit-elle, rend illusoire toute tentative de réduction à une variable explicative unique. Néanmoins cette illusion persiste encore dans certains secteurs des sciences sociales régis par une vision positiviste du monde. Elle est surtout dominante au sein des instances d’élaboration et de mise en oeuvre des politiques de santé.

Le cas des campagnes de vaccination montre bien ce fossé qui existe entre l’ingénierie biomédicale et l’ingénierie sociale, car elles associent les deux dimensions, et l’efficacité de l’une contraste avec les déboires de l’autre. Alors qu’un vaccin témoigne de propriétés intrinsèques qui sont pour l’essentiel contexte indépendant (le même vaccin contre la polio protège un cadre de Wall Street ou un paysan de la vallée du Mékong), son administration repose au contraire sur des architectures institutionnelles et des montages organisationnels qui sont de part en part « sociaux », et profondément dépendant du contexte. C’est ainsi que les Journées nationales de vaccination, qui sont un modèle voyageur largement célébré, organisées et financées à grands frais chaque année par le Fonds Gavi dans tous les pays d’Afrique, présentées mondialement comme de grands succès sur la base de statistiques faisant état d’une augmentation spectaculaire de la couverture vaccinale, ont de très nombreux effets pervers [97], entre autres dus au fait qu’elles fonctionnent depuis des années sur un mécanisme de type PBF (rémunérer les vaccinations à la performance) : désorganisation des services de santé (les agents de santé délaissent les activités de routine pour courir derrière les primes), multi-vaccinations systématiques et falsification des chiffres (pour « gonfler » les résultats), fausses missions des cadres (liées aux stratégies d’appropriation de la rente par la hiérarchie sanitaire); pour le Niger, cet aspect a été mis en évidence par un audit international qui a abouti à l’incarcération (provisoire) de plusieurs douzaines de médecins [134]). Diverses enquêtes qualitatives du LASDEL non encore publiées attestent de l’importance des multi-vaccinations et de la falsification des chiffres. Mais plusieurs études de santé publique plus classiques montrent aussi les limites de ces stratégies vaccinales, y compris au Burkina Faso [135], et évoquent « les inexactitudes de la couverture » [136].

## Partir des normes pratiques, pour induire des changements : à la quête des innovations et des réformateurs de l’intérieur

Quelles alternatives se présentent face aux modèles voyageurs ? Ce domaine reste très mal connu et reste à explorer systématiquement de façon empirique.

Une des pistes possibles consiste à partir des normes pratiques, pour induire un changement progressif de celles-ci, plutôt que d’importer encore et toujours de nouvelles normes officielles standardisées, au fil des interventions qui se succèdent et s’empilent sans guère modifier les comportements routiniers et sans stratégies crédibles d’appropriation et de pérennisation. Parmi les normes pratiques en cours dans des maternités, lesquelles peuvent être amendées, modifiées, améliorées, ou même valorisées ? Lesquelles doivent-elles et peuvent-elles abandonner, et comment, au profit de quelles autres? Comment peut-on introduire de meilleures normes pratiques, et les pérenniser ou les institutionnaliser? Cette perspective privilégie le « sur mesure » plutôt que le « prêt-à-porter ». Mais en outre, les normes pratiques peuvent aussi être parfois positives, innovantes. Elles ne sont pas toujours au service de stratégies opportunistes, conformistes ou égoïstes, et peuvent au contraire permettre de se « débrouiller », de « bricoler » des solutions, d’inventer des pratiques « palliatives », de développer des stratégies de mise en charge dans l’intérêt des patientes, de gérer la pénurie ou la démotivation des collègues, de tenter des formes adaptées de leadership…

Nous avons en effet rencontré des sages-femmes ou des gynécologues « réformatrices », qui tentent d’améliorer la qualité des services de santé, à partir des contextes réels, à partir des problèmes du terrain, sans appliquer pour autant intégralement les interventions standardisées, loin d’une logique de l’observance stricte des modèles voyageurs. Leurs initiatives sont discrètes, éparpillées, en général quasi invisibles, et méritent d’être connues et documentées.

### Le cas d’une sage-femme réformatrice

Pour proposer un exemple allant dans ce sens, nous évoquerons une étude de cas, qui porte sur une directrice de maternité « réformatrice » au Niger [119]. Cette enquête a été menée dans le cadre d’un mémoire d’une maîtrise d’anthropologie de la santé de l’Université Abdou Moumouni et du programme de recherche sur les sages-femmes financé par le CRDI et dirigé par Aïssa Diarra. Sur trois points, la directrice a réussi à modifier les normes pratiques des personnels dans sa maternité, sans toutefois recourir aux normes officielles (et en restant toujours dans un écart avec celles-ci)

**Retard au travail** L’horaire officiel de travail des agents de santé est de 7 h 30 à 16 h 30 avec une heure de pause déjeuner. Mais les sages-femmes arrivent vers 9 heures et partent vers 13 heures (sauf lorsqu’elles sont de garde). Pour tenter de faire respecter les horaires, le Ministère a récemment tenté une intervention : il oblige les formations sanitaires à tenir un cahier de présence, avec une ligne rouge à 8 heures. On ne s’étonnera pas de ce que, partout, les personnels continuent à arriver comme d’habitude vers 9 heures, mais reportent sur le cahier une heure d’arrivée fictive antérieure à 8 heures. La directrice de la maternité Salam n’utilise pas le cahier officiel, elle sait qu’il est inutile. Par contre, elle arrive elle-même à 8 heures, et fait alors le tour de tous les services, pour saluer les personnels, qui se sentent donc obligés d’être présents. La directrice, parce qu’elle donne elle-même l’exemple, et grâce à un contrôle « doux », fait mieux que la mesure bureaucratique imposée officiellement par le Ministère, mais sans respecter les normes officielles (début à 7 h 30, tenue du cahier).

**Les hémorragies** L’hémorragie post-partum est le plus souvent liée à une faute de la sage-femme en charge de l’accouchement. Mais, au Niger, personne n’utilise les sanctions légales prévues pour les fautes professionnelles. La directrice de la maternité Salam n’utilise pas non plus les sanctions légales. Mais elle a « inventé » une sanction locale (une norme pratique qu’elle a imposée) : en cas d’hémorragie, la sage-femme doit accompagner elle-même la parturiente jusqu’au centre de référence. Pour la sage-femme en cause ce sera une perte de temps (et d’argent) et, pire, une honte – les agents du centre de référence savent qu’il y a eu faute si on leur amène une hémorragie, et voient la fautive face à eux.

**Les paiements informels** On peut estimer à environ 10 dollars par accouchement l’argent que les sages-femmes (et les autres personnels des maternités) gagnent en moyenne avec les paiements « informels » (et illégaux) qu’elles exigent des parturientes (vente d’ocytocine, de sérum, de fil de suture, de gants, etc.). La directrice de la maternité Salam n’a pas tenté de supprimer ces « prélèvements » (ce qui serait peu réaliste), mais de les réduire. Elle a réuni son personnel, en donnant un plafond de 5 dollars à ne pas dépasser.

On a donc ici un exemple de réformes locales, partant des normes pratiques pour en modifier certaines, sans pour autant tenter de faire appliquer les normes officielles, qui actuellement non seulement sont inappliquées (revanche des contextes), mais inapplicables (en l’absence d’une réforme radicale de la fonction publique et de l’Etat).

# Conclusion

Il existe bien sûr au Niger – comme dans tous les pays – d’autres sages-femmes réformatrices (ou, plus généralement, personnel de santé réformateur). Certes, elles constituent une minorité invisible, mais pourquoi ne pas tenter de les mettre en réseau, et de s’appuyer sur elles pour définir diverses pistes d’amélioration des maternités? C’est ce que tente un programme de recherche-action du LASDEL. Ce programme, intitulé « Santé maternelle et adolescente en Afrique de l’Ouest. Pour des réformes à faibles coûts enracinées dans la réalité », concerne le Bénin et le Niger, et est financé par le CRDI. Le volet central porte sur la recherche et la documentation de réformes locales, et de regroupement d’efficacité [137–139] internes au système de santé maternelle, autrement dit d’innovations formelles ou informelles ayant permis des changements de comportement en faveur d’une meilleure qualité des soins. Autour d’une douzaine de sages-femmes « réformatrices », il s’agira, sur la base de leur expérience dans le « monde réel », et de leur connaissance des normes pratiques en usage, de définir des actions expérimentales qu’elles mettront elles-mêmes en oeuvre dans quelques maternités, avec pour objectif ultérieur de servir de points d’appui à des « coalitions réformatrices » locales et régionales dans le domaine de la santé maternelle, afin de modifier et d’améliorer, étape par étape, la culture professionnelle des sages-femmes nigériennes (sur la base de normes pratiques). C’est cette culture professionnelle (à base de normes pratiques) qu’il faut tenter de modifier, d’améliorer, pas à pas. C’est ce que ne peuvent pas faire les modèles voyageurs, aussi valables techniquement soient-ils. Les cultures professionnelles en place sont en général gagnantes dans leur confrontation avec les modèles voyageurs. Pour transformer ces cultures professionnelles, il y a certainement d’autres pistes que celle que nous suggérons ici. Il y a certainement d’autres façons de partir des contextes. Par exemple, à côté de ces « réformateurs de l’intérieur », au niveau individuel, que nous venons d’évoquer, certaines institutions, parfois d’origine extérieure, en générale de petite taille, tentent de leur côté de développer aussi des approches innovantes s’appuyant sur les réalités locales. On peut ainsi citer le cas de l’approche relative à la sécurité du réseau en santé maternelle, menée par une ONG (Organization of Human Welfare) qui repose sur la préoccupation suivante : « *Our argument is that cultural specificity is irreducible and not standardisable, but rather a basis for creating plural and complex models for intervention. How, then, might we make use of cultural specificity as a starting point for designing interventions, rather than as a reproducible and reducible set of variables that would look structurally the same in any target community?* » [140].

Ces tentatives diverses de réformes ancrées dans les contextes ou de modification des comportements à partir des normes pratiques révèlent un important manque de connaissances. Identifier et documenter empiriquement toutes les innovations à « faible bruit » (ou même silencieuses) qui prennent place au sein même des systèmes de santé et du « monde réel » est une des tâches prioritaires qu’on peut assigner à la socio-anthropologie de la santé. On doit déplorer qu’il y ait bien peu d’intérêt dans les revues professionnelles de santé publique pour des démarches allant dans ce sens, tant l’hégémonie des modèles voyageurs est grande, et tant le poids des méthodes quantitatives qui leur sont associées est lourd [43, 141]. Il y a aussi bien peu de crédits disponibles pour de telles recherches et de telles expériences, malgré leur coût dérisoire comparé aux coûts des modèles voyageurs ou aux coûts des études d’impact randomisées. Il y a enfin bien peu de place et d’audience pour elles dans les colloques de santé publique, de santé globale et même sur les systèmes et politiques de santé. Il y a heureusement au sein des institutions de santé publique (y compris dans le cadre de l’initiative pour une maternité sans risques), des professionnels qui souhaitent aller dans ce sens et appellent à la « diversité épistémologique » [43]. Des collaborations croissantes se sont nouées dans cette perspective avec l’anthropologie de la santé depuis une vingtaine d’années.

Nous savons qu’il serait, d’un point de vue pragmatique, totalement illusoire de vouloir mettre fin aux modèles voyageurs (qui, par ailleurs, quelles que soient leurs limites, ont aussi leur utilité et leur efficacité). Il faut aussi oeuvrer pour que les modèles voyageurs soient plus sensibles au contexte et prennent mieux en compte les contraintes, les ressources, les aspirations, les normes et les stratégies des acteurs locaux ou régionaux. Cependant, n’est-il pas possible d’allouer un peu plus d’espace, de temps, d’attention et d’argent, dans le monde de la santé publique en général et de la santé maternelle en particulier, pour des recherches alternatives (qualitatives) centrées sur des contextes pragmatiques, et pour des expériences « de l’intérieur » considérant la réalité quotidienne des normes sociales et des normes pratiques? Est-ce trop demander?

#### Abréviations

ANC: Antenatal care; FANC: Focused antenatal care; GFATM: Global Fund against Aids, Tuberculosis and Malaria; CRDI: Centre de recherches pour le développement international; LASDEL: Laboratoire d’études et Recherches sur les Dynamiques Sociales et le Développement Local (Social dynamics and local development research and study laboratory); LMICs: Low- and middle-income countries; NGO: Non-governmental organisation; PBF: Paiement basé sur la performance; PMTCT: Prevention of mother to child transmission of HIV; RBF: Result-based financing; RCT: Randomised control trials; UNICEF: United Nations International Children’s Emergency Fund

#### Remerciements

Les auteurs tiennent à remercier Susan Godt, Marie-Gloriose Ingabire et Valéry Ridde pour leurs commentaires sur une première ébauche du texte. Cet article est tiré d’un programme de recherche exécuté par le LASDEL (Niger).

#### Financement

Le programme de recherche et la publication ont été financés par le Centre de recherches pour le développement international du Canada.

#### Disponibilité des données et du matériel

Les ensembles de données acquis et/ou analysés au cours de la présente étude sont disponibles auprès de l’auteur correspondant sur demande raisonnable.

#### Contribution des auteurs

JPOS a conçu l’idée et a écrit l’ébauche et la version finale du manuscrit. AD et MM ont fourni des données et des idées issues d’années de recherche sur les questions de santé maternelle. Tous les auteurs ont lu et approuvé la version définitive du texte.

#### Intérêts conflictuels

Les auteurs déclarent n’avoir pas d’intérêts conflictuels.

#### Consentement à la publication

Ne s’applique pas.

#### Approbation éthique et consentement à la participation

Ne s’applique pas.

Date de publication : 12 juillet 2017

#### Références

1. Pressman, J. et A. Wildavsky, *Implementation: How Great Expectations in Washington Are Dashed in Oakland: Or, Why It’s Amazing that Federal Programs Work at All, This Being a Saga of the Economic Development Administration as Told by Two Sympathetic Observers Who Seek to Build Morals on a Foundation of Ruined Hopes*, Berkeley, Los Angeles et Londres, University of California Press, 1973.
2. Fixsen, D.L., S.F. Naoom, K.A. Blase, R.M. Friedman et F. Wallace, *Implementation Research: A Synthesis of the Literature*, Tampa, University of South Florida, The National Implementation Research Network, publication FMHI n^o^ 231, 2005.
3. Thomas, J. et M. Grindle, « After the decision: implementing policy reforms in developing countries », *World Dev.*, vol. 18, n^o^ 8, 1990, p. 1163-81.
4. Barrett, S, « Implementation studies: time for a revival? Personal reflections on 20 years of implementation studies », *Public Adm.*, vol. 87, 2004, p. 938-54.
5. Nilsen, P., C. Stahl, K. Roback et P. Cairney, « Never the twain shall meet? A comparison of implementation science and policy implementation research », *Implement Sci.*, vol. 8, 2013, p. 63.
6. Elwert, G. et T. Bierschenk, « Development aid as an intervention in dynamic systems. An introduction », *Sociol Rural.*, vol. 28, n^os^ 2-3, 1988, p. 99-112.
7. Olivier de Sardan, J.P., « Peasant logics and development projects logics », *Sociol Rural*, vol. 28, n^os^ 2-3, 1988, p. 216-26.
8. Walt, G. et L. Gilson, « Reforming the health sector in developing countries. The central role of policy analysis », *Health Policy Plan*, vol. 9, 1994, p. 353-70.
9. Erasmus, E. et L. Gilson, « How to start thinking about investigating power in the organizational settings of policy implementation », *Health Policy Plan*, vol. 23, 2008, p. 361-8.
10. Ridde, V., « Policy Implementation in an African state: an extension of the Kingdon’s multiple-streams approach », *Public Adm.*, vol. 87, 2009, p. 938-54.
11. Gilson, L., « Building the field of health policy and systems research: social science matters », *PLoS Med.*, vol. 8, n^o^ 8, 2011, p. 1-6.
12. Bunce, A., *et al.* « Ethnographic process evaluation in primary care: explaining the complexity of implementation », *BMC Health Serv Res.*, vol. 14, 2014, p. 607.
13. Pérez, D., P. Van der Stuyft, Z.M. del Carmen, M. Castro et P. Lefèvre, « A modified theoretical framework to assess implementation fidelity of adaptive public health interventions », *Implement Sci.*, vol. 11, 2016, p. 91.
14. Miller, S., N.L. Sloan, B. Winikoff, A. Langer et F.F. Fikree, « Where is the “E in MCH? The need for an evidence-based approach in safe motherhood », *J Midwifery Womens Health.*, vol. 48, n^o^ 1, 2003, p. 10-8.
15. Pawson, R., *Evidence-Based Policy: A Realist Perspective*, Thousand Oaks, Sage, 2006.
16. Marchal, B., *et al.* « Is realist evaluation keeping its promise? A review of published empirical studies in the field of health systems research », *Evaluation.*, vol. 1, n^o^ 2, 2012, p. 192-212.
17. Huby, G. « Addressing the complexity of health care: the practical potential of ethnography », *J Health Serv Res Policy.*, vol. 12, 2007, p. 193-94.
18. Sarriot, E., E. Sacks et L. Larson, « Self-organization and emergence in global health. Insights from practice, blind spots and possible contributions from complexity », *Sci Int J Design Nat Ecodynamics.*, vol. 11, n^o^ 4, 2016, p. 644-53.
19. Dopson, S., L. Locock, J. Gabbay, E. Ferlie et L. Fitzgerald, « Evidence-based medicine and the implementation gap », *Health.*, vol. 7, n^o^ 3, 2003, p. 311-30.
20. Peters, D., N. Tran et T. Adam, *La recherche sur la mise en oeuvre en santé : Guide pratique*, Genève, OMS, 2013.
21. Robson, C. *Real World Research: A Resource for Social Scientists and Practitioner-Researchers*, Oxford, Blackwell, 2002.
22. Mumtaz, Z., A. Levay, A. Bhatti et S. Salway, « Good on paper: the gap between programme theory and real world context in Pakistan’s community midwife programme », *BJOG.*, vol. 122, n^o^ 2, 2015, p. 249-58.
23. Glaser, B. et A. Strauss, *The Discovery of Grounded Theory. Strategies for Qualitative Research*, Chicago, Eldin, 1973.
24. Jaffré, Y. et J.P. Olivier de Sardan, *Une médecine inhospitalière : Les difficiles relations entre soignants et soignés dans cinq capitales d’Afrique de l’Ouest*, Paris, Karthala, 2003.
25. Olivier de Sardan, J.P., A. Moumouni et A. Souley, « L’accouchement c’est la guerre. Accoucher en milieu rural nigérien », *Afrique Contemporaine*, vol. 195, 2000, p. 136-54.
26. Moumouni, A. et A. Souley, *La Maternité Issaka Gazoby et l’Hôpital National de Niamey*, Niamey, Etudes et Travaux n^o^ 19, LASDEL, 2004.
27. Olivier de Sardan, J.P. et N. Bako Arifari, *La référence obstétricale au Bénin, étude socio-anthropologique*, Niamey, Etudes et Travaux n^o^ 97, LASDEL, 2011.
28. Diarra, A., *La prise en charge de l’accouchement dans trois communes au Niger. Say, Balleyara et Guidan Roumji*, Niamey, Etudes et Travaux n^o^ 101, LASDEL, 2012.
29. Olivier de Sardan, J.P. et V. Ridde, *Une politique publique de santé et ses contradictions. La gratuité des soins au Burkina Faso, au Mali et au Niger*, Paris, Karthala, 2014.
30. Olivier de Sardan JP, Ridde V., « Diagnosis of a public policy: an introduction to user fee exemptions for healthcare in the Sahel », *BMC Health Serv Res.*, vol. 15, suppl. 3, 2015, p. S2.
31. Jaffré, Y., Y. Diallo, P. Vasseur, C. et Grenier-Torrès, *La bataille des femmes. Analyse anthropologique de la mortalité maternelle dans quelques services d’obstétrique d’Afrique de l’Ouest*, Descartes, Éditions Faustroll, 2009.
32. Jaffré, Y., « Towards an anthropology of public health priorities: maternal mortality in four obstetric emergency services », *Soc Anthropol.*, vol. 20, n^o^ 1, 2012, p. 3-18.
33. Souley, A., *Interactions entre personnels de santé et usagers à Niamey*, Niamey, Etudes et Travaux n^o^ 2, LASDEL, 2001.
34. Souley, A., « Un environnement inhospitalier », dans Jaffré, Y., Olivier de Sardan, J.P. (dir.), *Une médecine inhospitalière. Les difficiles relations entre soignants et soignés dans cinq capitales d’Afrique de l’Ouest*, Paris, Karthala, 2003.
35. Moussa, H., « Niamey: le complexe sanitaire de Boukoki », dans Jaffré, Y., J.P. Olivier de Sardan (dir.), *Une médecine inhospitalière. Les difficiles relations entre soignants et soignés dans cinq capitales d’Afrique de l’Ouest*, Paris, Karthala, 2003.
36. Moussa, H., *La pratique de la planification familiale en milieu rural : Cas du district de Kollo*, Niamey, Etudes et Travaux n^o^ 23, LASDEL, 2004.
37. Touré, L. « User-fee exemption policies in Mali: sustainability jeopardized by the malfunctioning of the health system », *BMC Health Serv Res.*, vol. 15, suppl. 3, 2015, p. S8.
38. Diarra, A. et Ousseini, A., « The strategies of front-line health workers in the context of user fee exemption in Niger », *BMC Health Serv Res.*, vol. 15, suppl 3, 2015, p. S1.
39. Paul, E., N. Sossouhounto et D. Eclou, « Local stakeholders’ perceptions about the introduction of performance-based financing in Benin: a case study in two health districts », *Int J Health Policy Manag.*, vol. 3, n^o^ 4, 2014, p. 207-14.
40. Hibou, B., *La bureaucratisation du monde à l’ère néolibérale*, Paris, La Découverte, 2012.
41. Timmermans, S. et Berg, M., *The Gold Standard. The Challenge of Evidence-Based Medicine and Standardization in Health Care*, Philadelphie, Temple University Press, 1997.
42. MacDonald, M., *et al.* « Supporting successful implementation of public health interventions: protocol for a realist synthesis », *Syst Rev.*, vol. 5, 2016, p. 54.
43. Behague, D.T. et K.T. Storeng, « Pragmatic politics and epistemological diversity: the contested and authoritative use of historical evidence in the safe motherhood initiative », *Evidence Policy.*, vol. 9, n^o^ 1, 2013, p. 65-85.
44. Rottenburg, R., *Far-Fetched Facts. A Parable of Development Aid*, Cambridge, MIT Press, 2007.
45. Behrends, A., S.J. Park et R. Rottenburg, *Travelling Models in African Conflict Management. Translating Technologies of Social Ordering*, Leiden, Brill, 2014.
46. Sabatier, P. (dir.), *Theories of the Policy Process. Theoretical Lenses on Public Policy*, Boulder, Westview Press, 1992.
47. Lemieux, V. *L’étude des politiques publiques : Les acteurs et leur pouvoir*, Québec, Presses de l’Université Laval, 2002.
48. Kingdon, J., *Agendas, Alternatives and Public Policies*, New York, Harper Collins, 1995.
49. Fritsche, G.B., R. Soeters et B. Meessen, *Performance-Based Financing Toolkit*, Washington, World Bank Publications, 2014.
50. Turcotte-Tremblay, A.M., J. Spagnolo, M. De Allegri et V. Ridde, « Does performance-based financing increase value for money in low- and middle-income countries? A systematic review », *Heal Econ Rev.*, vol. 6, n^o^ 1, 2016, p. 30.
51. Maynard, A., « The powers and pitfalls of payment for performance », *Health Econ.*, vol. 21, 2012, p. 3-12.
52. Turcotte-Tremblay, A., L. Gautier, O. Bodson, N. Sambieni et V. Ridde, « Dans les coulisses du pouvoir décisionnel : Le rôle des organisations internationales dans l’expansion du financement basé sur les résultats dans les pays à faible et à moyen revenu », dans Matyjasik, N., directeur, *En finir avec le New Public Management? De nouvelles perspectives pour l’action publique*, Paris, Éditions du Comité pour l’Histoire Économique et Financière de la France, 2016.
53. Meessen, B., L. Musango, J.P.I. Kashala et J. Lemlin, « Reviewing institutions of rural health centres: the performance initiative in Butare, Rwanda », *Trop Med Int Health.*, vol. 11, n^o^ 8, 2006, p. 1303-17.
54. Soeters, R., Habineza, C. et P.B. Peerenboom, « Performance-based financing and changing the district health system: experience from Rwanda », *Bull World Health Organ.*, vol. 84, n^o^ 11, 2006, p. 884-9.
55. Kalk, A., Paul F. Amani et E. Grabosch, « ’Paying for performance’ in Rwanda: does it pay off? », *Tropical Med Int Health.*, vol. 15, n^o^ 2, 2009, p. 181-90.
56. Basinga, P., P.J. Gertler, A. Binagwaho, A.L. Soucat, J. Sturdy et C.M. Vermeersch, « Effect on maternal and child health services in Rwanda of payment to primary health-care providers for performance: an impact evaluation », *Lancet*, vol. 377, n^o^ 9775, 2011, p. 1421-8.
57. Lannes, L., B. Meesen, A. Soucat et P. Basinga, « Can performance-based financing help reaching the poor with maternal and child health services? The experience of rural Rwanda », *Int J Health Plann Manage.*, vol. 31, n^o^ 3, 2016, p. 309-48.
58. Logie, D.E., M. Rowson et F. Ndagije, « Innovations in Rwanda’s health system: looking to the future », *Lancet*, vol. 372, n^o^ 9634, 2008, p. 256-61.
59. Lascoumes, P., P. Le Galès, « Introduction: understanding public policy through its instruments. From the nature of instruments to the sociology of public policy instrumentation », *Governance Int J Policy Adm Inst.*, vol. 20, 2007, p. 1-21.
60. Lascoumes, P., P. Le Galès (dir.), *Gouverner par les Instruments*, Paris, Presses de la Fondation Nationale des Sciences Politiques, 2004.
61. Ancelovici, M. et J. Jenson, « La standardisation et les mécanismes du transfert transnational », *Gouvernement et Action Publique*, vol. 1, n^o^ 1, 2012, p. 37-58.
62. Olivier de Sardan, J.P., « State bureaucracy and governance in West Francophone Africa. Empirical diagnosis, historical perspective », dans Blundo, G., P.Y. Le Meur (dir.), *The Governance of Daily Life in Africa. Ethnographic Explorations of Public and Collective Service*, Leiden, Brill, 2009.
63. Oxman, A.D. et A. Fretheim, *An Overview of Research on the Effects of Results-Based Financing*, Oslo, Norwegian Knowledge Center for Health Services, 2008.
64. Bédécarrats, F., *La Microfinance : Entre utilité sociale et rentabilité financière*, Paris, L’Harmattan, 2013.
65. Olivier de Sardan, J.P. et E. Piccoli, *Cash Transfers: The Revenge of Contexts. An Anthropological Approach*, New York, Berghahn Books, 2017.
66. Chen, H., *Theory-Driven Evaluation*, Newbury Park, Sage Publications, 1990.
67. Weiss, C.H., *Theory-Based Evaluation: Past, Present and Future. New Directions for Evaluation*, San Francisco, Jossey-Bass; 1997.
68. Coryn, C., L. Noakes, C. Westine et D. Schröter, « A systematic review of theory-driven evaluation practice from 1990 to 2009 », *Am J Eval.*, vol. 32, n^o^ 2, 2011, p. 199-226.
69. Lacouture, A., E. Breton, A. Guichard et V. Ridde, « The concept of mechanism from a realist approach: a scoping review to facilitate its operationalization in public health program evaluation », *Implement Sci.*, vol. 10, 2015, p. 153.
70. Elster, J., *Nuts and Bolts for the Social Sciences*, Cambridge, Cambridge University Press, 1989.
71. Hedström, P. et Swedberg, R. *Social Mechanisms. An Analytical Approach to Social Theory*, Cambridge, Cambridge University Press, 1998.
72. Hedström, P. et R. Swedberg, « Social mechanisms », *Acta Sociologica*, vol. 39, 1996, p. 281-308.
73. Gerring, J., « The mechanistic world view: thinking inside the box », *Br J Polit Sci.*, vol. 38, n^o^ 1, 2007, p. 161-79.
74. Pawson, R. et N. Tilley, *Realistic Evaluation*, Londres, Sage, 1997.
75. Ridde, V., E. Robert, A. Guichard, P. Blaise et J. Van Olmen, « L’approche Realist à l’épreuve du réel de l’évaluation des programmes », *Revue canadienne d’évaluation de programme*, vol. 26, n^o^ 3, 2012, p. 14.
76. Marchal, B., *et al.* *Methodological Reflections on Using Realist Evaluation in a Study of Fee Exemption Policies in West Africa and Morocco*, Cotonou et Bruxelles, Centre de recherche en reproduction humaine et en démographie et Institute of Tropical Medicine of Antwerp, 2013.
77. Dalkin, S., *et al.* « What’s in a mechanism? Development of a key concept in realist evaluation », *Implement Sci.*, vol. 10, 2015, p. 49.
78. Callon, M. et B. Latour, « Unscrewing the big Leviathan: how actors macrostructure reality and how sociologists help them to do so», dans Cicourel, A.V. et K.D. Knorr-Cetina (dir.), *Advances in Social Theory: Towards an Integration of Micro- and Macro-Sociologies*, Boston, Londres, Henley, Routledge and Kegan Paul, 1981.
79. Latour, B., *Pasteur : Guerre et Paix des Microbes*, Paris, La Découverte, 2001.
80. Nachi, M., *Introduction à la sociologie pragmatique*, Paris, Armand Colin, 2006.
81. Martuccelli, D. *Forgé par l’épreuve. L’individu dans la France contemporaine*, Paris, Armand Colin, 2006.
82. Prual, A., A. Toure, D. Huguet et Y. Laurent. « The quality of risk factor screening during antenatal consultations in Niger », *Health Policy Plan*, vol. 15, n^o^ 1, 2000, p. 11–6.
83. Organisation mondiale de la Santé, *WHO Antenatal Care Randomized Trial: Manual for the Implementation of the New Model*, Genève, Organisation mondiale de la Santé, 2002, p. 37.
84. Health Education and Training Team for Africa, *Antenatal Care Module. Chapter 13: Providing Focused Antenatal Care*, Ethiopian Health Extension Programme, Londres, The Open University Press, 2011.
85. von Both, C., S. Flessa, A. Makuwani, R. Mpembeni et A. Jahn, « How much time do health services spend on antenatal care? Implications for the introduction of the focused antenatal care model in Tanzania », *BMC Pregnancy Childbirth*, 2006, vol. 23, n^o^ 6, p. 22.
86. Conrad, P., G. Schmid, J. Tientrebeogo, A., Moses, S. Kirenga, F. Neuhann et M. Sarker, « Compliance with focused antenatal care services: do health workers in rural Burkina Faso, Uganda and Tanzania perform all ANC procedures? », *Tropical Med Int Health.*, vol. 17, n^o^ 3, 2012, p. 300-7.
87. Olivier de Sardan, J.P., A. Diarra, F.Y. Koné, M. Yaogo et R. Zerbo, « Local sustainability and scaling up for user fee exemptions: medical NGOs vis-à-vis health systems », *BMC Health Serv Res.*, vol.15, suppl. 3, 2015, p. S5.
88. Jewkes, R., A. Naeemah et M. Zodumo, « Why do nurses abuse patients? Reflections from South African obstetric services », *Soc Sci Med.*, vol. 47, n^o^ 11,1998, p. 1781-95.
89. Olivier de Sardan, J.P., N. Bako Arifari, A. Moumouni, « La corruption dans le domaine de la santé », dans Blundo G., Olivier de Sardan, J.P. (dir.), *État et Corruption en Afrique. Une anthropologie comparative des relations entre fonctionnaires et usagers (Bénin, Niger, Sénégal)*, Paris, Karthala, 2007.
90. Jordan Smith, D. « Patronage, per diems and the “workshop mentality’: the practice of family planning programs in southeastern Nigeria », *World Dev.*, vol. 31, n^o^4, 2003, p. 703-15.
91. Fassin, D., « La vente illicite de médicaments au Sénégal. Économies “parallèles”, état et société », *Politique Africaine*, vol. 23, 1986, p. 123-30.
92. Koné, M., « Contexte’ et ‘gombo’ dans les formations sanitaires », dans Jaffré, Y., Olivier de Sardan, J.P. (dir.), *Une Médecine Inhospitalière. Les difficiles relations entre soignants et soignés dans cinq capitales d’Afrique de l’Ouest*, Paris, Karthala, 2003.
93. Belaid, L. et Ridde, V. « Contextual factors as a key to understanding the heterogeneity of effects of a maternal health policy in Burkina Faso? », *Health Policy Plann.*, vol. 30, n^o^ 3, 2015, p. 309-21.
94. Schaaf, M. et L. Freedman, « Unmasking the open secret of posting and transfer practices in the health sector », *Health Policy Plann.*, vol. 30, n^o^ 1, 2015, p. 121-30.
95. Sheikh, K., L. Freedman, A. Ghaffar, B. Marchal, F. el-Jardali, J. McCaffery, J.P. Olivier de Sardan, M. Dal Poz, W. Flores, S. Garimella et M. Schaaf, « Posting and transfer: key to fostering trust in government health services », *Hum Resour Health.*, vol. 13, 2015, p. 82. DOI : 10.1186/s12960-015-0080-9.
96. Bonnecase, V., « A quoi servent les indicateurs nutritionnels? », *Cahiers Agriculture*, vol. 21, 2012, p. 311-7.
97. Sandefur, J. et A. Glassman, « The political economy of bad data: evidence from African survey and administrative statistics », vol. 51, n^o^ 2, 2015, p. 116-32. DOI : 10.1080/ 00220388.2014.968138.
98. Greenhalgh, T., G. Robert, F. Macfarlane, P. Bate et O. Kyriakidou. « Diffusion of innovations in service organizations: systematic review and recommendations », *Milbank Q.*, vol. 82, n^o^ 4, 2004, p. 581-629.
99. Kaplan, H.C., P.W. Brady, M.C. Dritz, D.K. Hooper, W. Linam, C.M. Froehle et P. Margolis, « The influence of context on quality improvement success in health care: a systematic review of the literature », *Milbank Q.*, vol. 88, n^o^ 4, 2010, p. 500-59.
100. Abbott, A, « What do Cases do? Some Notes on Activity in Sociological Analysis », dans Ragin, C., Becker, H. (dir.), *What is a Case? Exploring the Foundations of Social Enquiry*, Cambridge, Cambridge University Press, 1992.
101. Giddens, A., *The Constitution of Society: An Outline of the Theory of Structuration*, Cambridge, Polity Press, 1984.
102. Long, N., « From paradigm lost to paradigm regained? The case for an actor-oriented sociology of development », dans Long, N., A. Long (dir.), *Battlefields of Knowledge. The Interlocking of Theory and Practice in Social Research and Development*, Londres, Routledge, 1992.
103. Mahoney, J. et K. Thelen, *Explaining Institutional Change: Ambiguity, Agency and Power*, Cambridge, Cambridge University Press, 2010.
104. Lewis, D. et Mosse, D., « Encountering order and disjuncture: contemporary anthropological perspectives on the organisations of development », *Oxford Contemp Stud.*, vol. 34, n^o^ 1, 2006, p. 1-14.
105. Fassin, D. « Le culturalisme pratique de la santé publique. Critique d’un sens commun », dans Dozon, J.P., D. Fassin (dir.), *Critique de la Santé Publique. Une Approche Anthropologique*, Paris, Balland, 2001.
106. Olivier de Sardan, J.P. « Health fee exemptions: controversies and misunderstandings around a research programme. Researchers and the public debate », *BMC Health Serv Res.*, vol. 15, suppl. 3, 2015, p. S4.
107. Checkland, K., Harrison, S. et M. Marshall, « Is the metaphor of “barriers to change’ useful in understanding implementation? Evidence from general medical practice », *J Health Serv Res Policy*, vol. 12, 2007, p. 95-100.
108. Olivier de Sardan, J.P. « La sage-femme et le douanier. Cultures professionnelles locales et culture bureaucratique privatisée en Afrique de l’Ouest », *Autrepart.*, vol. 20, 2001, p. 61-73.
109. Vasseur, P. « Le travail des sages-femmes, entre savoir technique et normes pratiques », dans Jaffré, Y., Y. Diallo, P. Vasseur, C. Grenier-Torrès (dir.), *La bataille des femmes. Analyse anthropologique de la mortalité maternelle dans quelques services d’obstétrique d’Afrique de l’Ouest*, Descartes, Les Éditions Faustroll, 2009.
110. Hahonou, E., « Juggling with the norms. Everyday practice in an emergency service in Niger », dans De Herdt, T., J.P. Olivier de Sardan (dir.), *Real Governance and Practical Norms in Sub-Saharan Africa. The Game of the Rules*, Londres, Routledge, 2015.
111. Lemarcis, F. et J. Grard, « Ethnography of everyday ethics in a South African medical ward », dans De Herdt, T., J.P. Olivier de Sardan (dir.), *Real Governance and Practical Norms in Sub-Saharan Africa. The Game of the Rules*, Londres, Routledge, 2015.
112. Olivier de Sardan, J.P., « Africanist traditionalist culturalism: analysis of a scientific ideology and a plea for an empirically grounded concept of culture encompassing practical norms », dans De Herdt, T., J.P. Olivier de Sardan (dir.), *Real Governance and Practical Norms in Sub-Saharan Africa. The Game of the Rules*, Londres, Routledge, 2015.
113. De Herdt, T., J.P. Olivier de Sardan, *Real Governance and Practical Norms in Sub-Saharan Africa. The Game of the Rules*, Londres, Routledge, 2015.
114. Jaffré, Y. et A. Prual, « Midwives in Niger: an uncomfortable position between social behaviours and health care constraints », *Soc Sci Med.*, vol. 38, n^o^ 8, 1994, p. 1069-73.
115. Vasseur, P., *Socialisation des sages-semmes jeunes diplômées dans la vie active au Sénégal*, Marseille, 2004. Mémoire de l’EHESS.
116. Diarra, A. *Socio-anthropologie de la prise en charge de l’accouchement au Mali*, Marseille, EHESS, 2010. Thèse de doctorat en anthropologie.
117. Diop, N., B. Mané, F.B. Mbow et A. Thiam, *Etude exploratoire des soins respectueux aux femmes fréquentant les services de santé de la reproduction dans deux centres urbains principaux du Sénégal – Investigation des données qualitatives*, Dakar, Population Council, 2013.
118. Moha, M., V. Ridde et C. Dagenais, *Personnels d’obstétrique nigériens et conditions pour exercer en milieu rural*, Niamey-Montréal, LASDEL-REALISME, 2015.
119. Souley Issoufou, M.S., *Ecarts aux normes officielles dans deux maternités du Niger*, Niamey, Etudes et Travaux n^o^ 115, du LASDEL, 2017.
120. Pavignani, E. et Colombo, S., *Analysing Disrupted Health Sectors: A Modular Manual*, Genève, OMS, 2009.
121. Hirschman, A., *Development Projects Observed*, Washington, The Brooking Institution, 1967.
122. Naudet, J.D., *Trouver des problèmes aux solutions : Vingt ans d’aide au Sahel*, Paris, OCDE, 1999.
123. Easterly, W., *The White Man’s Burden. Why the West’s Efforts to Aid the Rest Have done so Much Ill and so Little Good*, New York, Penguin, 2006.
124. Mosse, D., *Cultivating Development: An Ethnography of Aid Policy and Practice*, Londres, Pluto Press, 2005.
125. Olivier de Sardan, J.P., *Anthropology and Development. Understanding Contemporary Social Change*, Londres, Zed Books, 2005.
126. Gardner, K. et D. Lewis, *Anthropology and Development: Challenges for the Twenty-First Century*, Londres, Pluto Press, 2015.
127. Li, T.M., *The Will to Improve: Governmentality, Development and the Practice of Politics*, Londres, Duke University Press, 2007.
128. Darbon, D., « Modèles et transferts institutionnels vus des Afriques: les nouveaux villages Potemkine de la modernité? », dans Darbon, D. (dir.), *La Politique des Modèles en Afrique. Simulation, Dépolitisation et Appropriation*, Paris, Karthala, 2009.
129. DiMaggio, P. et Powell, W.W., « The iron cage revisited: collective rationality and institutional isomorphism in organizational fields », *Am Sociol Rev.*, vol. 48, n^o^2, 1983, p. 147-60.
130. Cohen, M., J. March et J.P. Olsen, « A garbage can model of organisational choice », *Adm Sci Quaterly*, vol. 17, 1972, p. 1-15.
131. Olivier de Sardan, J.P., « The bureaucratic mode of governance and practical norms in West Africa and beyond », dans Bouziane, M., C. Harders et A. Hofmann (dir.), *Local Politics and Contemporary Transformations in the Arab World. Governance Beyond the Center*, Basingstoke, Palgrave Macmillan, 2013.
132. Passeron, J.C., « Le raisonnement sociologique. La preuve et le contexte », dans Michaud, Y. (dir.), *L’Histoire, la Sociologie et l’Anthropologie*, Paris, Odile Jacob, 2002.
133. Passeron, J.C., *Le Raisonnement Sociologique. Un Espace Non-Poppérien de l’Argumentation*, Paris, Albin Michel, 2006.
134. Gavi Investigation Mission, *Investigation Report*, Niamey, 2012. Sur Internet : [http/www.gavi.org/librairie/documents-gavi/legal/rapport-d-investigation-(niger)- f%C3%A9vrier-2012/](http://gavi.org/librairie/documents-gavi/legal/rapport-d-investigation-(niger)-). Consulté le 28 juin 2017.
135. Haddad, S., A. Bicaba, *et al.*, « System-level determinants of immunization coverage disparities among health districts in Burkina Faso: a multiple case study », *BMC Int Health Hum Rights*, vol. 9, suppl. 1, 2009, p. S15.
136. Mhatre, S. et A.M. Schryer-Roy, « The fallacy of coverage: uncovering disparities to improve immunization rates through evidence. Results from the Canadian International Immunization Initiative Phase 2 », *BMC Int Health Human Rights*, vol. 9, suppl. 1, 2009, p. 51.
137. Crook, R., « Rethinking civil service reform in Africa: ‘Islands of effectiveness’ and organisational commitment », *Commonwealth Comp Polit.*, vol. 48, n^o^ 4, 2010, p. 479-504.
138. Leonard. D., « Pockets of effective agencies in weak governance states: where are they likely and why does it matter? », *Public Adm Dev.*, vol. 30, n^o^ 2, 2010, p. 91-101.
139. Roll, M. (dir.), *The Politics of Public Sector Performance: Pockets of Effectiveness in Developing Countries*, Londres, Routledge, 2014.
140. Adams, V., Craig, S.R. et A. Samen, « Alternative accounting in maternal and infant global health », *Global Public Health*, vol. 11, n^o^ 3, 2016, p. 276-94.
141. Social Science Approaches for Research and Engagement in Health Policy & Systems (SHaPeS) Thematic Working Group of Health Systems Global, Regional Network for Equity in Health in East and Southern Africa (EQUINET), Emerging Voices for Global Health et Daniels, K., *et al.*, « Fair publication of qualitative research in health systems: a call by health policy and systems researchers », *Int J Equity Health*, vol. 15, 2016, p. 98.
