# Supplementary material for: People and research: improved health systems for West Africans, by West Africans - report on special supplement
Source: BMC Proc. 2019 Feb 7;13(Suppl 1):1. doi: 10.1186/s12919-019-0162-0 (PMC6366023; doi:10.1186/s12919-019-0162-0)
Supplement: Supplementary file 8 — Évaluation d’un projet régional visant à renforcer les systèmes nationaux de recherche en santé dans quatre pays d’Afrique de l’Ouest: leçons apprises, Sombie, I., Aidam, J., Montorzi, G. [file 12919_2019_162_MOESM8_ESM.docx]

***Évaluation d’un projet régional visant à renforcer les systèmes nationaux de recherche en santé dans quatre pays d’Afrique de l’Ouest: leçons apprises***

**Issiaka Sombié^*^, Jude Aidam^1^ et Gabriela Montorzi^2^**

* Correspondance : [isombie@wahooas.org](mailto:isombie@wahooas.org)

^1^Organisation ouest-africaine de la Santé, BP 153 Bobo-Dioulasso 01, Burkina Faso

^2^La liste complète des auteurs est disponible à la fin de la section.

# Résumé

**Contexte** : Depuis la publication du rapport phare du Conseil de la recherche en santé pour le développement (COHRED), plus d’attention a été portée au renforcement des systèmes nationaux de recherche en santé (SNRS). Le présent article évalue l’apport d’un projet régional ayant utilisé une approche participative pour renforcer les SNRS dans quatre pays post-conflit en Afrique de l’Ouest : Guinée-Bissau, Libéria, Sierra Leone et Mali.

**Méthodologie** : Une comparaison a été réalisée entre les données de l’analyse de la situation réalisée au début du projet et les données de l’évaluation finale du projet, au moyen d’un cadre conceptuel hybride construit autour de quatre domaines clés identifiés à travers l’analyse de cadres existants. Ces quatre domaines sont la gouvernance et la gestion, les capacités, le financement et la diffusion/utilisation des résultats de la recherche.

**Résultats** : Le projet a contribué à une amélioration des mécanismes de gouvernance et de gestion dans les pays sans pour autant obtenir un renforcement global des SNRS. Ainsi, dans les quatre pays au moins une politique, un plan ou un programme de recherche ont été élaborés. Un pays a mis en place un comité national d’éthique en recherche pour la santé, tandis que les quatre pays ont pu adopter un système de gestion de l’information sur la recherche. L’approche participative du projet, l’accompagnement par l’équipe de l’Organisation ouest-africaine de la santé et du COHRED ont tous été des facteurs déterminants.

**Conclusion** : Les leçons tirées montrent que le contexte fragile de ces pays nécessite un projet sur le long terme et que l’accompagnement des pays par une institution régionale est nécessaire pour relever les défis et aboutir à un renforcement global des SNRS.

**Mots-clés** : Guinée-Bissau, Libéria, Sierra Leone, Mali, système national de recherche en santé, post-conflit, renforcement des systèmes de santé, Afrique de l’Ouest

# Contexte

En 1990, le rapport du Conseil de la recherche en santé pour le développement affirmait que « le renforcement des capacités de recherche dans les pays en développement est l’un des moyens les plus puissants, les plus rentables et les plus durables pour faire progresser la santé et le développement [1]. » Depuis lors, une plus grande attention a été accordée aux systèmes nationaux de recherche en santé dans les pays en développement [2-18], et plusieurs initiatives visant à renforcer ces systèmes ont eu lieu en Afrique [10, 13-15]. Un système national de recherche est une composante importante d’un système moderne de santé qui permet de définir l’environnement dans lequel la recherche est encouragée et réalisée. Il est défini par l’Organisation mondiale de la Santé (OMS) comme un système qui fournit la gouvernance, le développement des capacités, la génération des connaissances et les mécanismes d’utilisation des données probantes, soutenu par des mécanismes de financement durables des systèmes de santé d’un pays pour conduire les activités de recherche [6]. Enfin, il regroupe l’ensemble des parties prenantes de la recherche depuis les chercheurs, les gestionnaires de la recherche, les bailleurs de fonds jusqu’aux utilisateurs potentiels de la recherche (décideurs, société civile, associations professionnelles).

L’Organisation ouest-africaine de la santé (OOAS) est l’institution spécialisée de la Commission économique des États de l’Afrique de l’Ouest (CEDEAO) dans le domaine de la santé. Sa mission est d’aider les pays de l’OOAS à offrir à leur population la meilleure qualité de soins. Pour ce faire, elle doit promouvoir la recherche en santé afin de s’assurer que les décisions sont fondées sur des données probantes. Le renforcement des systèmes nationaux de la recherche dans les pays de la région constitue une étape clé de cette promotion de la recherche. En 2009, avec l’appui des partenaires techniques, l’OOAS a organisé un atelier régional d’analyse des systèmes nationaux de la recherche des 15 pays de la CEDEAO [19].

Il en est ressorti une faiblesse de ces systèmes, qui se caractérisaient par : l’absence de politiques, plans et agendas de recherche; l’absence de cadre ou de mécanisme de coordination des parties prenantes; l’inexistence ou le faible fonctionnement des comités nationaux d’éthique; l’absence de mécanismes de suivi et d’évaluation; de faibles capacités individuelles et institutionnelles pour gérer et conduire la recherche; une faible collaboration entre les chercheurs; une faible diffusion des résultats; et une faible utilisation de ces résultats dans les prises de décision. L’analyse a aussi montré que trois pays (Guinée-Bissau, Libéria et Sierra Leone) étaient très en retard par rapport aux autres. Leur caractéristique commune était qu’il s’agissait de pays post-conflit caractérisés par de rares ressources humaines qualifiées, un nombre important de priorités concurrentes, un nombre élevé de parties prenantes participant à la reconstruction [20-25], une attention plus marquée portée à l’offre de soins qu’à la recherche, un environnement d’insécurité et un financement limité de la recherche [26-28]. Pour répondre à ces besoins, un projet régional axé sur le renforcement des systèmes de recherche nationaux a été élaboré dans ces trois pays. Le Mali a également été ajouté parce que, bien qu’il dispose de nombreuses institutions de recherche, il souffrait d’une mauvaise coordination entre les parties prenantes [29].

Déployé de 2011 à 2015 avec l’appui technique du COHRED, ce projet a été mis en oeuvre par l’OOAS en quatre étapes : l’analyse de la situation pour mieux comprendre les systèmes nationaux de la recherche des différents pays; l’identification des priorités et des activités à mettre en place sur le plan national; la mise en oeuvre des activités choisies; et l’évaluation du projet. Le projet a adopté un processus participatif, incluant des parties prenantes des pays bénéficiaires ainsi que d’autres partenaires sur le terrain. Il a débuté avec un atelier de lancement auquel furent invités trois représentants de chaque pays. Ces représentants incluaient le responsable de la recherche du ministère de la Santé et du ministère de l’Éducation et des Sciences et Technologies, et un directeur d’un institut de recherche en santé publique. Au cours de cette rencontre, la description des SNRS dans les quatre pays a été livrée à partir des données de la rencontre de 2009, et des informations complémentaires ont été recueillies. Un exercice de cartographie des différentes parties prenantes de la recherche en santé a été entrepris par les équipes des pays, avec pour but d’identifier le potentiel existant. Les équipes ont aussi identifié les priorités pour améliorer le SNRS dans chaque pays. Enfin, la plateforme HRWeb [30,31], qui avait été développée par le COHRED, a été présentée comme outil d’aide à la gestion de l’information sur la recherche.

Les exercices de cartographie des parties prenantes et d’identification des priorités et activités à mettre en oeuvre ont été validés lors d’un atelier d’information sur le projet dans chacun des quatre pays en 2011, et un appui technique et financier a été apporté aux équipes.Le présent article tente d’évaluer si les activités de ce projet régional ont renforcé les systèmes nationaux de recherche en santé dans ces quatre pays. Nous utilisons ici un cadre conceptuel hybride construit autour de quatre domaines clés, identifiés à travers l’analyse de cadres existants [2, 16-18] et les priorités déterminées lors de l’atelier de 2009. Étant donné que ce projet a été développé par l’OOAS, une institution régionale, les leçons qui en ont été tirées ont aussi été utilisées pour formuler des propositions relatives au rôle d’une institution régionale et des autres partenaires techniques et financiers dans le renforcement des systèmes nationaux de recherche en santé.

# Méthodologie

L’étude se fonde sur une évaluation comparative avant et après l’intervention. Les données « préintervention » proviennent de l’analyse de la situation réalisée au début du projet pour mieux caractériser les systèmes nationaux d’intervention. Les données de fin du projet proviennent du rapport final d’évaluation du projet, tandis que les informations du projet sont tirées du protocole et des rapports de mise en oeuvre du projet.

## Analyse de la situation du système national de recherche dans les quatre pays

L’analyse de la situation au début du projet a été réalisée à l’aide de trois sources d’information. La première a été un examen de la documentation associée aux entretiens avec des participants des quatre pays à l’atelier de lancement du projet sur le plan régional en 2011 [27]. Les autres sources sont deux études réalisées dans les différents pays lors d’ateliers pour valider les plans formulés pendant l’atelier de lancement. Elles ont été utilisées pour analyser la gestion de l’information sur la recherche et la formation au renforcement des capacités dans les quatre pays [32, 33].

## Évaluation externe du projet

Pour analyser la situation à la fin de l’intervention, nous avons utilisé le rapport de l’évaluation externe [34] menée à la fin du projet par un expert indépendant, qui a réalisé une revue documentaire et une analyse qualitative. La revue documentaire a consisté en une analyse du protocole du projet, des plans de mise en oeuvre de l’activité ainsi que des rapports techniques et financiers d’activité et de mise en oeuvre du projet. Cette revue documentaire a permis d’analyser la planification du projet, les stratégies d’intervention, les activités planifiées et leur mise en oeuvre, les ressources utilisées, les résultats obtenus, les observations émises afin de dégager les forces, les faiblesses, les possibilités et les menaces ou défis qui ont été notés durant la réalisation du projet.

L’étude qualitative s’est fondée sur 50 entretiens directs, par Skype ou par téléphone à l’aide d’un guide d’entretien. Les personnes interviewées étaient l’équipe de la direction générale de l’OOAS, les responsables des départements, services et programmes de l’OOAS, les responsables de la recherche en santé des ministères de la Santé des quatre pays du projet, les responsables des institutions de recherche des pays, les agents de liaison et responsables des sites HRWEB-pays, les correspondants OOAS-pays, les responsables des réseaux de chercheurs dans les pays, les personnes-ressources des institutions internationales partenaires de l’OOAS dans la mise en oeuvre du projet, à savoir le Centre de recherche pour le développement international et le COHRED. Les entretiens ont abordé ce qui a fonctionné et ce qui n’a pas fonctionné dans la mise en oeuvre, ce qui a été bien réalisé et ce qui ne l’a pas été, ce qu’il faudrait améliorer dans le futur et les leçons tirées.

Une triangulation de l’ensemble des données de la revue documentaire et de l’analyse qualitative a permis d’identifier les principaux résultats du projet, les résultats indirects, les défis et les leçons apprises qui devraient permettre un examen du rôle de l’OOAS et des autres acteurs pour améliorer des systèmes nationaux de recherche dans les pays post-conflit.

## Cadre conceptuel pour l’analyse du projet

Pour renforcer les capacités des systèmes nationaux de recherche en santé, plusieurs cadres conceptuels ont été proposés. Le cadre conceptuel de la capacité de la recherche élaboré par McIntyre [16] décrit les dimensions selon lesquelles le renforcement des capacités en matière de recherche en santé peut être réalisé sur le plan national. Il s’agit des dimensions individuelles et organisationnelles, incluant le gouvernement (plateformes de recherche et technologies), les ministères clés, les universités, les hôpitaux universitaires, les organisations non gouvernementales (ONG), les agences du système des Nations Unies, les structures de gestion de la recherche, les instituts publics de santé, l’entreprise privée et les structures de régulation (comité de contrôle de la qualité des médicaments et comités d’éthique). Le cadre conceptuel de Pang et col [2], ou cadre de l’OMS, a pour sa part été élaboré à partir des quatre fonctions essentielles que doit remplir un SNRS à savoir : le financement, la création et le soutien aux ressources, la production et la diffusion des résultats de la recherche. Enfin, le cadre conceptuel du COHRED a été élaboré pour aider les pays à construire un système solide avec des capacités lui permettant de produire des résultats de recherche optimaux [17]. Le cadre conceptuel du COHRED comprend quatre éléments : la fondation (développement des politiques, définition des priorités et gestion), les ressources (capacités, financement et coordination), l’optimisation (régulation éthique, engagement de la société civile, attribution équitable des contrats de recherche, innovation pharmaceutique, communication de la recherche, système d’information de la recherche et suivi de l’évaluation) et un environnement politique favorable (leadership) (tableau 1).

L’analyse de ces cadres montre la nécessité de travailler dans quatre domaines (gouvernance et gestion, capacités, financement et diffusion/utilisation des résultats de la recherche) pour réussir le renforcement d’un système national de la recherche. Aux fins du présent projet, nous avons construit un cadre avec ces quatre domaines. Pour mesurer les progrès par rapport à chacun de ces quatre domaines, des indicateurs ont été proposés par d’autres [2,18].

La gouvernance est une fonction du gouvernement de surveillance, de construction d’une coalition, d’un système de reddition de comptes et de régulation de toutes les recherches pour la santé qui prennent place aussi bien dans les secteurs privés que public [18]. Ainsi, il suppose l’existence de politiques, de plans, de priorités, de lois, de textes et de mécanismes de régulation comme les comités d’éthique. La gestion permet la mise en oeuvre de lignes directrices nationales fondées sur un cadre réglementaire et à des fins de suivi des processus. Cela nécessitera un système de gestion de l’information de recherche qui recueille des données pour l’élaboration d’indicateurs prédéfinis. Ainsi, les indicateurs du domaine de la gouvernance et de la gestion étaient la disponibilité et la mise en oeuvre de politiques, de plans stratégiques, d’agendas de recherche, l’existence d’un comité national d’éthique en santé, l’existence d’un mécanisme de coordination de la recherche et l’existence d’un système de gestion de l’information sur la recherche.

Le renforcement des capacités se définit comme un processus de transfert des compétences et des capacités à des individus, des institutions, des organisations ou à un pays qui va leur permettre de définir et prioriser les problèmes systématiquement, de développer et évaluer scientifiquement les solutions ainsi que de partager et d’appliquer les connaissances générées [16,35]. Selon Lansang et al [35], d’autres stratégies individuelles, institutionnelles et nationales à court et à long terme peuvent être appliquées. Celles-ci comprennent les formations de base ou supérieures, l’apprentissage sur le tas, la promotion du partenariat entre institutions de pays développés et de pays en développement et la création de centres d’excellence. Ainsi, les indicateurs sélectionnés dans ce domaine incluaient l’existence de programmes de renforcement des capacités individuelles (méthodologie de la recherche, éthique, gestion de la recherche, diffusion et utilisation de la recherche) pour gérer, conduire et partager les résultats de la recherche, le nombre de personnes ayant bénéficié de ces programmes de renforcement des capacités et le nombre d’activités de renforcement de capacité institutionnelle.

La fonction de financement comprend l’estimation des besoins financiers, la mobilisation, la mise à disposition et la gestion des fonds venant du gouvernement, des bailleurs de fonds internes et externes pour les individus, les institutions et les organisations afin de faire fonctionner le système national de recherche en santé [18]. La disponibilité d’une ligne budgétaire pour appuyer la recherche d’un ministère de la santé est une reconnaissance de l’importance de la recherche et l’engagement d’un gouvernement à soutenir la recherche. Ainsi, dans le cadre du projet, à la suite des différents engagements internationaux - par exemple consacrer 2 % des budgets de la santé à la recherche -, l’indicateur retenu a été la disponibilité d’une ligne budgétaire du ministère de la Santé dédiée à la recherche.

**Tableau 1** Analyse comparative des différents cadres conceptuels pour le renforcement des systèmes nationaux de recherche en santé.

| Domaine | Cadre de Pang et al. [2] | Cadre de McIntyre [16] | Cadre du COHRED [17] |
| --- | --- | --- | --- |
| Gouvernance et gestion | Intendance (définir et articuler une vision pour le système national de recherche en santé; déterminer les priorités de recherche en santé appropriées et coordonner leur respect; établir et surveiller les normes éthiques pour la recherche en santé et les partenariats de recherche en santé; surveiller et évaluer le système de recherche en santé). | Environnement national de recherche (gouvernance et coordination, directives/normes éthiques) Réseau de travail (ministère de la Santé, bailleurs de fonds internationaux, ONG du Sud, universités du Nord) | Fondations (élaboration des politiques, définition des priorités et gestion) Ressources (coordination) Optimisation (réglementation éthique, engagement de la société civile, système d’information de recherche, suivi et évaluation) Environnement politique favorable (leadership) |
| Capacités | Créer et maintenir des ressources (développer, renforcer et maintenir les capacités humaines et institutionnelles pour mener, absorber et utiliser la recherche en santé). | Individuel (formation et expériences, disciplines, recherche, compétences en gestion et en leadership) Institutionnel (masse critique, environnement externe/infrastructure, environnement collégial, leadership, financement) | Ressources (capacités) |
| Financement | Financement (obtenir des fonds de recherche et les répartir de façon responsable) | Environnement de recherche national (demande de financement des extrants de recherche) Environnement de recherche externe (bailleurs de fonds internationaux, organisations internationales) | Ressources (financement)  Optimisation (attribution équitable des contrats de recherche) |
| Diffusion et utilisation des résultats de la recherche | Produire et utiliser des ressources (produire des recherches scientifiquement valables; traduire et communiquer la recherche pour éclairer les politiques, les stratégies, les pratiques et l’opinion publique; promouvoir l’utilisation de la recherche pour développer de nouveaux outils - par exemple des médicaments, des vaccins, des dispositifs - pour améliorer la santé) |  | Optimisation (communication de la recherche) |

La fonction de diffusion et d’utilisation de la recherche comprend la dissémination et l’application des résultats de la recherche à la défense, à la planification, à l’élaboration et à la mise en oeuvre des politiques, des plans et au suivi des évaluations. Pour réussir la diffusion et l’application, il faut des plateformes de connaissances qui permettent la traduction, la synthèse, la communication des résultats de recherche pour informer les politiques et les pratiques [18]. Il peut s’agir de plateformes de transfert de connaissances, un forum d’échanges entre chercheurs et décideurs et une base des publications nationales. Ici, on a choisi comme indicateur l’existence de cadre d’échanges entre chercheurs et décideurs permettant de partager et d’échanger sur les résultats de recherche et leur utilisation, ce qui va créer un espace de dialogue entre les chercheurs et les décideurs.

# Résultats Gouvernance et gestion de la recherche

Durant la phase d’analyse de la situation, les quatre pays étaient signataires des déclarations d’Alger sur la recherche pour la santé [36] et de Libreville sur la santé et l’environnement [37], et disposaient de politiques ou de plans de santé accordant une place importante au développement de la recherche, démontrant une volonté de développer la recherche en santé [27].

Cette volonté était plus marquée en Guinée-Bissau, qui avait déposé une demande d’appui au COHRED en 2008 [28]. Alors que la structure de gouvernance de la recherche pour la santé était basée au ministère de la Santé dans trois pays, au Mali elle l’était au ministère de l’Enseignement supérieur et de la Recherche scientifique. Dans trois pays, ces structures étaient au niveau de la direction, tandis qu’au Libéria elle était au niveau de l’unité [11]. Ces structures étaient en général dirigées par des personnes non formées à la gestion de la recherche et elles ne disposaient pas de plus de trois agents. Seul le Mali disposait d’un document de politique et d’un plan de développement de la recherche. Il n’existait dans aucun pays un mécanisme de coordination des différentes parties prenantes de la recherche. Le Libéria était le seul pays qui ne disposait pas d’un comité national d’éthique, mais il disposait de deux comités institutionnels d’éthique, dont un jouait le rôle de comité national d’éthique, sans coordination efficace entre eux toutefois. Il existait dans tous les pays des documents pouvant être utilisés pour la réglementation de la recherche. Aucun des quatre pays ne disposait d’un mécanisme centralisé de gestion de l’information sur la recherche ni d’une cartographie des parties prenantes de la recherche en santé.

Les priorités retenues variaient d’un pays à l’autre. L’équipe du Libéria a donné la priorité à la promotion d’une culture de l’utilisation des résultats de la recherche parmi les décideurs et les responsables politiques, à la mobilisation de fonds pour la recherche en santé, à la mise en place d’un comité national d’éthique, à l’établissement d’un programme national de gouvernance et au développement d’un système de gestion de l’information sur la recherche qui sera soutenu par HRWeb. Les priorités de la Sierra Leone visaient à élaborer une politique et un plan de recherche en santé, à renforcer l’évaluation éthique et les ressources humaines pour la recherche en santé et à mobiliser des ressources financières pour la recherche en santé. L’équipe de la Guinée-Bissau a présenté un plan de travail fixant des objectifs et des activités, en commençant par une recherche sur la cartographie du système de santé et en jetant les bases d’une bonne gouvernance, l’établissement des priorités, la création de comités d’éthique, ainsi que le plaidoyer pour des fonds durables. Elle a également proposé un processus de renforcement des capacités, y compris la gestion financière et la responsabilité, la communication, la mise en réseau et le suivi de l’utilisation des résultats de la recherche. Juste avant l’atelier de lancement, le Mali a adopté une politique nationale de recherche en santé et un plan d’action, une vision à long terme pour améliorer le système de recherche en santé et, en fin de compte, pour réduire l’incidence de la maladie et de la mortalité. L’équipe a proposé un plan de travail lié au document ci-dessus, y compris la création d’un comité de coordination de la recherche en santé, l’amélioration des comités éthiques, le renforcement de la mobilisation de fonds, et finalement une meilleure diffusion et utilisation des résultats de la recherche, la formation des ressources humaines, la construction ou la rénovation d’infrastructures, le développement de systèmes informatiques et la mise en réseau, ainsi qu’un plaidoyer pour consacrer 2 % du budget national de la santé et 5 % des fonds des bailleurs de fonds pour les programmes de santé à la recherche pour la santé.

Au Libéria, le projet a donné l’impulsion pour la création d’une unité de recherche en santé au sein du ministère de la Santé et de la Protection sociale qui gérera et coordonnera la recherche en santé dans le pays. Le projet de carte du système, le plan de travail et le programme de recherche du Libéria ont été validés lors d’un atelier qui s’est tenu en décembre 2011. Des suggestions ont été émises concernant les rôles des individus et des institutions et, par conséquent, les rôles de certaines institutions ont été élargis. Une feuille de route a été rédigée pour l’élaboration d’un programme national de recherche sur la politique de santé qui fournira au pays un cadre politique et des lignes directrices pour la conduite de la recherche.

En Sierra Leone, le plan de travail a été revu et validé par les parties intéressées locales lors d’une réunion convoquée par le ministère de la Santé en 2011. Un consultant soutenu par le projet de renforcement des capacités du Partenariat irlando-africain pour la recherche en santé (ChRAIC) a coordonné l’élaboration et l’achèvement du programme national de recherche sur les politiques de santé. Grâce à l’appui de ce projet, un groupe intersectoriel a élaboré et examiné les deux documents. L’équipe était composée des mêmes groupes de membres représentés au sein de l’organe directeur. Le projet de carte du système, le plan de travail et le programme de recherche de Guinée-Bissau ont été validés lors d’un atelier qui s’est tenu en novembre 2011. Le projet a soutenu la finalisation d’un programme de recherche et sa traduction en anglais. Au Mali, lors d’une réunion des parties prenantes, le plan d’action et la carte du système de recherche en santé ont été validés avec des changements mineurs; quant aux termes de référence du comité national de coordination, ils ont été élaborés et soumis au ministre de la Santé pour la création du comité.

Le tableau 2 montre les changements liés aux mécanismes de gouvernance et de gestion de la recherche dans les quatre pays, à la fin du projet. Chacun des pays dispose d’une cartographie des différentes parties prenantes de la recherche pour la santé qui permettait d’avoir une connaissance de ces acteurs et de leurs rôles. Ainsi, on a noté que les différentes parties prenantes de la recherche pour la santé provenaient des ministères de la Santé, de l’Éducation, des Sciences et des Technologies, de l’Environnement, de l’Agriculture et de l’Élevage, des ONG, du système des Nations Unies, des universités, des bailleurs de fonds et d’importantes initiatives de recherche. Ces parties prenantes dans chaque pays ont été classées en tant que producteurs de l’information, régulateurs et consommateurs de la recherche. Ensuite, une politique, un plan stratégique et deux agendas ou priorités de recherche ont été élaborés. En Guinée-Bissau et au Libéria, les priorités de recherche avaient été finalisées, adoptées et partagées avec toutes les parties prenantes. Toutefois, la politique et le plan de renforcement du système national de la recherche élaborés en Sierra Leone n’ont pas été adoptés. L’élaboration d’une politique de santé au Libéria a été entamée avec la contribution technique et financière du bureau national de l’OMS, mais le processus a été interrompu avec la survenue de l’épidémie du virus Ebola. Actuellement, l’équipe du ministère de la Santé a exprimé le besoin d’un appui pour finaliser ce document. Au Mali, malheureusement, les termes de référence du comité de concertation de la recherche n’avaient pas été entérinés et le comité n’avait pas été mis en place. Un comité national d’éthique de la recherche en santé a été mis en place au Libéria, dont les membres ont bénéficié de la formation en éthique. Au chapitre de la gestion de l’information sur la recherche, chacun des quatre pays avait sa page sur la plateforme HRWeb, et une page régionale avait aussi été créée. Un lien a été établi entre les sites des ministères de la Santé, l’OOAS et la plateforme HRWeb. Dans chacun des quatre pays, et pour l’OOAS, des personnes ont reçu des autorisations leur permettant d’entrer des informations sur la plateforme pour le compte des pays et de l’OOAS. Ensuite, une liste de 24 indicateurs a été développée et générée directement par la plateforme sur la page de l’OOAS permettant d’établir la comparaison des pays en termes de gouvernance, d’institutions de régulation, d’institutions de recherche, d’organisations de la société civile et de projets de recherche [38].

**Tableau 2** Situation des pays au chapitre des SNRS avant et après la mise en oeuvre du projet régional selon les domaines du cadre.

| Domaines | Indicateurs | Guinée-Bissau | | Libéria |  | Mali |  | Sierra Leone | |
| --- | --- | --- | --- | --- | --- | --- | --- | --- | --- |
|  |  | Avant | Après | Avant | Après | Avant | Après | Avant | Après |
| Gouvernance et gestion | Existence d’une carte de la recherche pour les acteurs de la santé | Non | Oui | Non | Oui | Non | Oui | Non | Oui |
|  | Existence d’une politique, d’un plan et d’un programme de recherche | Non | Oui | Non | Oui | Oui | Oui | Non | Oui |
|  | Existence d’un système de coordination de la recherche | Non | Non | Non | Non | Non | Non | Non | Non |
|  | Existence d’un comité national d’éthique | Oui | Oui | Non | Oui | Oui | Oui | Oui | Oui |
|  | Existence d’un système de surveillance de la recherche en santé et d’indicateurs | Non | Oui | Non | Oui | Non | Oui | Non | Oui |
| Capacités | Existence d’un programme de formation à la recherche en santé | Oui | Oui | Oui | Oui | Oui | Oui | Oui | Oui |
|  | Nombre d’activités de renforcement des capacités institutionnelles |  |  |  | 1 |  |  |  |  |
|  | Nombre de personnes formées à l’examen éthique |  | 1 |  | 26 |  | 1 |  |  |
|  | Nombre de personnes formées à la gestion de la recherche au ministère de la Santé |  | 1 |  | 2 |  |  |  |  |
|  | Nombre de personnes formées en méthodologie de recherche |  | 18 |  | 32 |  | 2 |  | 8 |
|  | Nombre de personnes formées au niveau Master en recherche |  | 0 |  | 1 |  | 1 |  | 0 |
|  | Nombre de personnes formées à l’utilisation de la plateforme HRWeb |  | 20 |  | 24 |  | 28 |  | 28 |
| Financement | Existence de lignes budgétaires au sein du ministère de la Santé pour financer la recherche | Non | Non | Non | Non | Oui | Oui | Non | Non |
| Diffusion et utilisation des résultats de la recherche | Existence de mécanismes de diffusion des résultats de la recherche | Non | Non | Oui | Oui | Non | Non | Oui | Oui |

Ministère de la Santé

## Capacités

D’après l’analyse de la situation, les ressources humaines disponibles pour la santé dans les pays sortant d’un conflit étaient en général limitées. Cette disponibilité limitée était encore plus marquée dans le domaine de la recherche, où le nombre de doctorants était faible, sauf au Mali. En ce qui concerne les comités d’éthique, certains membres avaient été formés et d’autres non dans les quatre pays. L’analyse de situation a révélé qu’il existait dans tous les pays des formations en méthodologie de la recherche avec une grande variabilité en matière de durée, de contenu et de formateurs. De plus, sur le plan institutionnel, on notait que les structures responsables de la recherche n’avaient pas toutes les capacités pour être fonctionnelles. Dans tous les pays, des besoins de formation avaient été exprimés dans le domaine de l’éthique, de la gestion de la recherche, en méthodologie de la recherche et à l’utilisation de la plateforme HRWeb de gestion de l’information et de renforcement des capacités institutionnelles.

En fonction des différents besoins de formation, le projet a examiné les formations de courte durée existantes en méthodologie de la recherche et en éthique. Cet examen a révélé l’existence de plusieurs formations peu standardisées avec une difficulté de pérennisation. Dans le cadre du projet, trois approches de formation ont été utilisées. La première consistait en des formations de courte durée et permettait aux agents de ne pas s’éloigner trop longtemps de leur poste de travail. La deuxième était le soutien à des formations avec des diplômes universitaires. Des bourses étaient offertes par l’OOAS, selon un appel à soumission de candidature, et ouvertes aux ressortissants de tous les pays de l’espace CEDEAO ou à la suite d’une demande des pays. La troisième approche de formation était la promotion d’une formation en ligne en gestion de la recherche qui se déroulait en Amérique latine, en espagnol. Des formations de courte durée ont été organisées en méthodologie de la recherche, en éthique et dans l’utilisation de la plateforme HRWeb. Les formateurs en méthodologie de recherche étaient des formateurs régionaux et nationaux expérimentés en recherche, tandis que ceux dans le domaine de l’éthique et de l’utilisation de la plateforme HRWeb étaient des experts du COHRED. Les formations en méthodologie de la recherche et dans l’utilisation de la plateforme HRWeb ont été ouvertes à tous les autres pays de l’espace CEDEAO.

Sur le plan institutionnel, les équipes de l’OOAS et du COHRED ont aidé les responsables chargés de la gestion de la recherche à mieux comprendre leur rôle à travers les échanges et le partage de documents sur la gestion du SNRS. Au Libéria, ces équipes ont aussi contribué à l’élaboration des termes de référence de l’unité chargée de la recherche.

À la fin du projet, les formations de courte durée avaient permis de former au total 245 personnes (39 en Guinée-Bissau, 29 au Mali, 81 au Libéria et 37 en Sierra Leone) en éthique de la recherche, en gestion des programmes de recherche, en méthodologie de la recherche et dans l’utilisation de la plateforme HRWeb (tableau 2). Les personnes formées étaient des responsables de la gestion de la recherche au sein du ministère de la Santé et des institutions de recherche, des chercheurs, des responsables de programmes de santé et des informaticiens. Trois personnes (deux au Mali et une au Libéria) ont obtenu leur diplôme de Master en épidémiologie (tableau 2). Au Libéria, la personne formée était un agent au service de gestion de la recherche et au Mali les deux personnes travaillaient dans un hôpital et au ministère de la Santé. Au sujet de la formation en ligne, deux participants s’étaient inscrits mais, du fait de problèmes de connexion Internet, ont dû abandonner leur formation sans la terminer.

Parmi les impacts directs de ces formations, citons la mise en place du comité d’éthique national de la recherche du Libéria, dont les membres ont bénéficié de la formation en éthique organisée dans le cadre du projet, ainsi que l’existence d’informations disponibles sur la plateforme HRWeb. En septembre 2014, 279 documents de recherche étaient disponibles sur la plateforme HRWeb (y compris des documents de politiques, des lois, des plans et des agendas de recherche des différents pays), 118 projets de recherche examinés par les comités d’éthique, 226 institutions de recherche (y compris 5 en Guinée-Bissau, 24 au Mali, 8 au Libéria et 5 en Sierra Leone) et une liste d’une quarantaine de gestionnaires autorisés à saisir les informations, témoignant d’une utilisation de la plateforme. Un résultat indirect du projet a été l’affectation et l’utilisation de la plateforme au Bénin et au Niger, qui continue aujourd’hui. Après la formation en méthodologie de la recherche, on a noté une faiblesse des bénéficiaires des pays post-conflit qui, à leur retour, n’ont souvent pas finalisé leur protocole de recherche, contrairement aux participants des autres pays de la région.

En revanche, il faut souligner que les formations promues par le projet n’ont pas couvert tous les besoins des pays. De plus, seulement trois personnes ont pu bénéficier d’une formation diplômante, ce qui est insuffisant par rapport aux besoins. Par ailleurs, le choix des personnes à former ne relevait pas du projet. Enfin, la formation seule ne fournit pas tous les outils nécessaires pour garantir un travail efficace. Les besoins en matériel et en équipement ainsi que la nécessité de créer un environnement institutionnel n’étaient pas sous le contrôle du projet.

# Financement

Au début du projet, seul le Mali possédait une ligne budgétaire dédiée à la recherche au ministère de la Santé. Cependant, même au Mali, l’acquisition des fonds par les chercheurs restait difficile du fait de nombreuses lourdeurs administratives.

La priorité principale de chacun des pays était d’améliorer la mobilisation du financement du ministère de la Santé, et la stratégie adoptée au cours du projet pour atteindre cette priorité a été le plaidoyer auprès des autorités. Les équipes de l’OOAS et du COHRED sont intervenues, à chacune de leurs rencontres, auprès des autorités du ministère de la Santé durant leurs séjours dans les quatre pays. Il en a été de même pour les responsables des services de la recherche auprès de leurs responsables hiérarchiques.

À la fin du projet, aucune amélioration n’a été notée à cet égard, et le Mali était toujours le seul pays où il existait une ligne budgétaire dédiée à la recherche au sein du ministère de la Santé. Cependant, au Libéria et en Sierra Leone, des financements complémentaires ont pu être mobilisés auprès du bureau de l’OMS et auprès d’une organisation non gouvernementale, respectivement, pour couvrir les frais des consultants qui ont aidé à élaborer la politique et le plan de recherche.

## Diffusion et utilisation de la recherche

Au début du projet, seule la Sierra Leone possédait un cadre d’échanges entre chercheurs et décideurs qui pouvait faciliter le transfert des connaissances et l’utilisation des résultats de recherche. Il s’agissait de l’organisation par un groupe de chercheurs en collaboration avec le ministère de la Santé d’un symposium de la recherche biomédicale au cours duquel des résultats de recherche étaient présentés et discutés, et des formations en recherche réalisées. Au Libéria, un temps a été réservé lors de la réunion mensuelle du comité de coordination du secteur de la santé pour discuter de résultats de recherche, et un répertoire des recherches réalisées dans le pays a été constitué. La stratégie du projet a consisté à motiver les parties prenantes des pays à créer ces cadres de dialogue ou à soutenir les cadres existants. Ainsi, le projet a soutenu pendant deux années la tenue du symposium de la recherche biomédicale en Sierra Leone. Au cours de ces deux forums, des formations en rédaction de protocole et en rédaction de sections avaient été organisées au profit des chercheurs. Au Libéria, l’unité chargée de la recherche a créé une base des recherches effectuées dans le pays. Aucun cadre d’échanges entre chercheurs et décideurs n’a été créé.

Au chapitre des résultats en fin de projet, des différences ont été constatées entre les pays et seraient liées au contexte local, au leadership, à la volonté et à l’engagement des responsables de la mise en oeuvre du projet dans les différents pays. Le tableau 2 montre que le plus grand nombre de résultats a été obtenu au Libéria, où le contexte était plus favorable et où les personnes chargées de la mise en oeuvre du projet étaient motivées. Le projet a commencé sensiblement au même moment que la création de l’unité de la recherche qui était composée de jeunes cadres motivés et désireux d’obtenir des résultats. Ils ont donc saisi l’occasion. De plus, le bureau de l’OMS dans le pays était engagé dans le développement de la recherche. Il a apporté un appui matériel à l’unité de recherche, les cadres ont pris part aux réunions sur le plan national et un consultant a été appuyé dans le développement de documents de politique et de plans de recherche. Enfin, il n’y a pas eu de roulement dans l’unité de recherche, ce qui a permis la réalisation de la quasi-totalité des activités planifiées. Par contre, en Sierra Leone et en Guinée-Bissau, durant les quatre années de mise en oeuvre du projet, les organismes chargés de la recherche au sein du ministère de la Santé, qui était responsable de la mise en oeuvre du projet, ont connu respectivement deux et trois responsables. Par ailleurs, en 2012, le Mali et la Guinée-Bissau ont connu des coups d’État avec comme conséquence la suspension des voyages et du financement au Mali. Dans ces trois pays, ces différents événements ont eu engendré le ralentissement de la mise en oeuvre des activités. Au Libéria, malgré l’appui de l’OMS et du projet, le pays n’est pas parvenu à terminer l’élaboration et la validation de sa politique et de son plan de recherche. L’élaboration du document a commencé en 2013 et, malgré les différents appuis techniques et financiers, le problème de capacités techniques a été le facteur limitant aussi bien chez le consultant national qu’au sein de l’unité de recherche. En Sierra Leone et en Guinée-Bissau, les capacités techniques ont aussi joué un rôle déterminant. En effet, en Sierra Leone, deux personnes ont supervisé la recherche et la mise en oeuvre du projet. En Guinée-Bissau, le départ du premier responsable, qui avait une bonne capacité en gestion de la recherche, a nui à la réalisation du projet. Parmi les responsables du HRWeb, certaines personnes formées avaient besoin d’être motivées avant de continuer à saisir les informations sur la plateforme en ligne. Enfin, les faibles taux d’accessibilité et de connectivité à Internet dans les pays post-conflit (Guinée-Bissau, Libéria, Sierra Leone) ont influé sur l’utilisation de la plateforme HRWeb pour la gestion de l’information sur la recherche. En conclusion, le leadership, l’engagement, les compétences individuelles et organisationnelles, les ressources financières et l’engagement des parties prenantes sont essentiels pour créer un contexte favorable.

Les succès obtenus sont aussi dus en partie au projet et à la présence des équipes de l’OOAS et du COHRED, dont la contribution a été reconnue et bien reçue par les parties prenantes dans les pays, comme le démontre cette déclaration : «*L’équipe de l’OOAS a fait tout ce qu’elle a pu. Ses membres ont toujours été à nos côtés. La balle est dans le camp du pays pour faire bouger les choses. »* Les équipes des pays étaient satisfaites de cette présence, comme en témoigne cette citation du rapport final du projet : « *Les équipes de recherche des quatre pays enquêtés ont unanimement exprimé leur satisfaction quant à l’engagement et à la compétence dont ont fait preuve les membres de l’unité de recherche de l’OOAS dans le processus d’accompagnement des pays dans la mise en place ou le renforcement du système de recherche en santé. On a particulièrement souligné leur disponibilité et leur professionnalisme. L’observation de la dynamique du fonctionnement de l’équipe lors de l’enquête à l’OOAS n’a fait que confirmer cette appréciation [32].* »

# Discussion

Le projet a démontré qu’une approche systématique et participative pour développer les mécanismes nationaux de gouvernance et de gestion de la recherche était possible, même dans les pays post-conflit. En même temps, le renforcement de l’ensemble du système national de recherche est une tâche considérable qui nécessite un engagement à long terme. Le projet a contribué à améliorer l’environnement national dans quatre pays, sans pour autant relever tous les défis locaux qui ont un impact sur le renforcement global du système national de recherche en santé. Les leçons apprises indiquent que le rôle d’accompagnement de l’OOAS a été nécessaire et bien apprécié par les pays.

Les faiblesses identifiées lors de l’analyse de la situation des SNRS des quatre pays peuvent être regroupées sous les rubriques de la gouvernance, de la gestion, du financement, des capacités individuelles et institutionnelles, du leadership, de la collaboration, de la coordination, de la diffusion et de l’utilisation des résultats de recherche. Ces rubriques ont été ciblées dans le cadre conceptuel et corroborent celles identifiées dans d’autres études en Afrique [6, 8, 11, 12, 14].

Le choix des priorités pour le renforcement des SNRS diffère d’un pays à l’autre. Les expériences de renforcement des SNRS dans d’autres pays d’Afrique [10, 14, 35] ont montré que les besoins et les questions prioritaires à cet égard peuvent différer d’un pays à un autre, soulignant l’importance de fonder tout projet sur les priorités des parties prenantes locales. Cette approche présente l’avantage de mieux engager les acteurs locaux, comme nous l’avons constaté dans la mise en oeuvre du projet. En effet, c’est l’engagement des acteurs des quatre pays qui a permis la réalisation de la cartographie des différentes parties prenantes de la recherche, l’identification de leurs priorités et de leurs activités, et leur mise en oeuvre.

Il est ressorti que le projet a permis le développement de mécanismes de gouvernance et de gestion comme les politiques, les plans, les agendas de recherche, la mise en place d’un comité national d’éthique et l’adoption d’un système de gestion de l’information sur la recherche (la plateforme HRWeb). De tels succès ont été notés dans d’autres pays [10, 14, 35] et démontrent qu’il est possible d’apporter quelques changements qualitatifs dans les systèmes nationaux de recherche en Afrique, particulièrement dans les pays post-conflit où la recherche peut aider à une prise de décision éclairée dans le domaine de la planification. Kirigia et al [14] ont montré une tendance à l’amélioration dans les systèmes nationaux de recherche en Afrique de façon générale en comparant les résultats de trois études réalisées respectivement en 2003, 2009 et 2014. Malgré ces améliorations, les pays post-conflit restaient encore mal classés en 2014 en Afrique de l’Ouest [18]. En effet, dans ce classement parmi les 15 pays de la CEDEAO, le Mali était classé 4^e^, le Libéria 12^e^, la Guinée-Bissau 13^e^ et la Sierra Leone 14^e^, ce qui révèle la nécessité d’efforts sur le long terme dans ces pays.

Dans le cadre du projet, les facteurs suivants peuvent avoir contribué à l’obtention de certains résultats. Les activités du projet ont contribué à la création d’une fondation et à une meilleure connaissance de l’ensemble des parties prenantes de la recherche à travers la cartographie, une meilleure connaissance de l’état du SNRS à l’issue des analyses de la situation, la sensibilisation et la mobilisation des différents acteurs lors des ateliers sur les plans régional et national, la motivation des acteurs au changement en les engageant dans les activités d’identification des priorités et activités à mettre en oeuvre et en les exposant aux expériences d’autres pays, la création d’un dialogue entre les différentes parties prenantes au cours des différentes rencontres créant un lien de confiance, le soutien pour le changement à travers les appuis techniques et financiers qui ont permis aux équipes de chaque pays de réaliser les activités et le renforcement des capacités. Toutes ces actions ont permis d’obtenir une bonne compréhension de l’état actuel du SNRS, la mise en place d’une structure chargée du pilotage, de la gestion et de la coordination, la définition du rôle de tous les acteurs, la mise en place d’un réseau regroupant l’ensemble des parties prenantes assurant une harmonisation et un renforcement de leurs capacités. Ce sont ces éléments qui avaient été identifiés comme déterminants lors des expériences décrites en Afrique du Sud, au Kenya, au Malawi, en Tanzanie et en Zambie [10, 15, 36]. De plus, la présence de l’équipe de l’OOAS et de COHRED a été bien appréciée par les pays, et la contribution des partenaires sur le plan national a aidé à aboutir à des changements. Ceci montre l’importance de l’accompagnement des équipes des pays, qui ont souvent des faiblesses réelles en termes de capacités et qui sont confrontés à de nombreux défis. Cet accompagnement aide à surmonter les difficultés, motive les équipes à aller de l’avant et les aide à négocier avec les partenaires. De façon générale, le projet a contribué à informer, sensibiliser et mobiliser les parties prenantes locales sur la situation réelle de leur SNRS à travers les analyses de la situation, à motiver les acteurs à s’engager pour obtenir des changements à travers le choix des priorités et enfin en aidant les acteurs locaux à réaliser le changement en mettant à leur disposition des appuis techniques et financiers et en les accompagnant lors de la réalisation des activités. Ces actions du projet ont permis de créer un environnement qui a favorisé l’obtention de politiques, de plans, d’agendas de recherche et de comités éthiques. Toutefois, elles n’ont pas apporté tous les fondements pour un renfoncement d’un système national de recherche plus large, à savoir le leadership, les capacités individuelles suffisantes et organisationnelles, ce qui requiert des mesures à long terme. Cette réalité montre l’importance de la place des partenaires techniques auprès des parties prenantes dans les différents pays.

Malgré l’apport du projet, un renforcement global des SNRS n’a pas été obtenu. L’analyse de la mise en oeuvre du projet entre les différents pays a montré qu’en plus de l’apport du projet, le manque de leadership, l’appropriation du projet par les acteurs, l’existence d’un environnement de soutien, l’existence de capacités individuelles et institutionnelles, l’existence de moyens financiers et l’environnement sociopolitique difficile avaient tous joué un rôle, démontrant l’importance du contexte local. En effet, le contexte était caractérisé dans la plupart de ces pays par des ressources humaines limitées en nombre et en qualité, un roulement élevé, des problèmes de motivation et la bureaucratie. On sait que ces facteurs entraînent de mauvais rendements dans la plupart des pays en développement [39-45] et apparaissent plus marqués dans les pays post-conflit [46-53]. L’existence de ces facteurs exerce une influence dans la mise en oeuvre des projets et suggère que les projets de courte durée ne pourront pas renforcer tous les SNRS, surtout dans des pays post-conflit. Ces pays auront besoin d’accompagnement de longue durée afin de leur permettre de trouver des solutions à ces problèmes complexes.

Si la délégation des tâches à du personnel peu qualifié a été proposée pour améliorer l’offre de soins dans ces pays post-conflit, cette stratégie est impossible en termes de recherche, qui exige un personnel qualifié. La collaboration intersectorielle avec les ministères de l’Éducation, des Sciences et des Technologies et de la Recherche, les établissements d’enseignement, les institutions de recherche et les ONG devrait être une piste potentielle pour les pays post-conflit, ce qui permettra de combler une partie du problème des capacités. Une autre stratégie serait de réfléchir à un programme de renforcement des capacités avec l’apprentissage sur le tas, qui présente l’avantage de maintenir les acteurs sur le terrain et de joindre de nombreuses personnes.

Au cours de la mise en oeuvre du projet, le partage d’expériences entre pays a été promu et a été identifié comme un élément déterminant en Afrique de l’Est, en Afrique australe et en Afrique du Sud [10]. La pérennisation d’une telle activité peut être assurée par une institution régionale comme l’OOAS. Cette organisation pourrait aussi aider à négocier avec les bailleurs de fonds pour obtenir des projets sur le long terme et accompagner leur mise en oeuvre. L’OOAS, à travers ce projet régional, a montré qu’elle peut jouer un rôle d’appui aux pays post-conflit en Afrique de l’Ouest. Son rôle de facilitateur [54] comprend la promotion du leadership, le marketing, la communication stratégique, le plaidoyer politique, la coordination, la mise en réseau, le soutien à la mobilisation des ressources, le soutien à l’harmonisation et le développement de partenariats. Ces formes d’appui aident les pays à élaborer et à mettre en oeuvre des programmes de renforcement des capacités adaptés à leur situation. Actuellement, l’OOAS joue une partie de ce rôle à travers des projets régionaux financés par la Banque mondiale (WARDS [55], SWEDD [56] et REDISSE [57]), dans les domaines de la surveillance des maladies et de la promotion de la planification familiale renforçant la fonction d’appui de l’organisation auprès des pays. Ce rôle d’appui de l’OOAS a été bien perçu par une partie des pays post-conflit dans le domaine de la recherche. En effet, à la fin de l’épidémie du virus Ebola, les trois pays les plus touchés de la région ouest-africaine ont décidé de collaborer en matière de recherche et ont demandé un appui à l’OOAS [58]. Cet appel devrait être saisi par l’OOAS pour renforcer son rôle auprès des pays. L’OOAS devrait aussi améliorer son rôle en ajoutant la négociation et la médiation pour aider les pays à obtenir auprès des bailleurs de fonds des programmes de renforcement des capacités sur le long terme. Elle pourrait aussi utiliser une partie des fonds de ces projets pour assurer une collaboration régionale, un suivi d’évaluation et un accompagnement politique et technique. Elle dispose de mécanismes qui pourraient l’aider à jouer son rôle comme le forum CEDEAO des bonnes pratiques en santé, l’Assemblée annuelle des ministres de la Santé, les réunions techniques régionales entre pays, les cadres d’échanges entre parties prenantes de la recherche sur les plans national et régional. Les possibilités de renforcement de la recherche post-Ebola dans certains de ces pays post-conflit devraient être saisies pour impliquer tous les acteurs dans l’élaboration des programmes de renforcement des SNRS adaptés, qui pourraient être soutenus par toutes les parties prenantes dans ces pays.

En plus d’un rôle de l’OOAS, la participation d’autres parties prenantes comme les organismes des Nations Unies avec l’OMS en tant que chef de file est cruciale. En effet, de par leur présence permanente dans les pays et leurs activités, ces institutions contribuent déjà au renforcement des systèmes nationaux de la recherche à travers des appuis, comme cela a été constaté dans la mise en oeuvre du projet au Libéria et le financement des activités de recherche. Elles pourront aider au plaidoyer, à la mobilisation des fonds et à la mise en oeuvre technique des activités. La Banque mondiale, la Banque africaine de développement et les autres bailleurs de fonds devront être aussi des acteurs clés en apportant les fonds nécessaires pour permettre la mise en oeuvre de ces projets de renforcement des systèmes nationaux de recherche en santé.

# Conclusion

La présente évaluation a montré qu’un projet régional avec une approche participative peut contribuer à un changement positif sur les mécanismes de gouvernance et de gestion des SNRS. Cependant, un renforcement durable des capacités ne peut être obtenu que par un engagement à long terme.

#### Abréviations

COHRED : Conseil de la recherche en santé pour le développement

CEDEAO : Communauté économique des États de l’Afrique de l’Ouest

ONG : organisation non gouvernementale

SNRS : systèmes nationaux de recherche en santé

OOAS : Organisation ouest-africaine de la santé

#### Remerciements

Les auteurs aimeraient remercier tous leurs collègues et partenaires pour avoir travaillé avec eux et les avoir soutenus dans ce projet, situé dans la région de l’Afrique de l’Ouest.

#### Financement

La publication de cette section a été appuyée par le Centre de recherches pour le développement international dans le cadre du projet 106498-001 du CRDI.

Disponibilité des données et du matériel : toutes les données sont disponibles auprès des auteurs.

#### Contribution des auteurs

IS, JA et GM ont tous conçu l’examen, la rédaction du manuscrit et l’examen du présent document. Tous les auteurs ont lu et approuvé la version définitive du manuscrit.

#### Intérêts conflictuels

Les auteurs déclarent n’avoir pas d’intérêts conflictuels.

#### Consentement à la publication

Sans objet.

#### Approbation éthique et consentement à la participation

Sans objet.

#### Détails à propos des auteurs

^1^ Organisation ouest-africaine de la santé, BP 153, Bobo-Dioulasso, Burkina Faso. ^2^ Conseil de la recherche en santé pour le développement (COHRED), Genève, Suisse.

Date de publication : 12 juillet 2017

#### Références

1. Conseil de la recherche en santé pour le développement Health Research: Essential Link to Equity in Development. New York : Oxford University Press; 1990.
2. Pang T, Sadana R, Hanney S, Bhutta ZA, Hyder AA, Simon J. Knowledge for better health: a conceptual framework and foundation for health research systems. Bull World Health Organ. 2003;81(11):815-20.
3. Sadana R, Pang T. Current approaches to national health research systems analysis: a brief overview of the WHO health research system analysis initiative. Ciência & Saúde Coletiva 2004;9(2):351–62.
4. Sadana R, D’Souza C. Why do case studies on national health research systems matter? Identifying common challenges in low- and middle-income countries. Soc Sci Med. 2006;62:2072–8.
5. Hanney SR, González BM. Building health research systems to achieve better health. Health Res Policy Syst. 2006;4:10.
6. Kirigia JM, Wambebe C. Status of national health research systems in ten countries of the WHO African Region. BMC Health Serv Res. 2006;6:135.
7. Hanney SR, Gonzalez Block MA. Why national health research systems matter. Health Res Policy Syst. 2008;6:1. doi:10.1186/1478-4505-6-1.
8. Kennedy A, Khoja TA, Abou-Zeid AH, Ghannem H, IJsselmuiden C. WHOEMRO/COHRED/GCC NHRS Collaborative Group. National health research system mapping in 10 Eastern Mediterranean countries. East Mediterr Health J. 2008;14(3):502–17.
9. Palmer A, Anya SE, Bloch P. The political undertones of building national health research systems – reflections from The Gambia. Health Res Policy Syst. 2009;7:13.
10. Chanda-Kapata P, Sandy Campbell S, Zarowsky C. Developing a national health research system: participatory approaches to legislative, institutional and networking dimensions in Zambia. Health Res Policy Syst. 2012;10:17.
11. Sombié I, Aidam J, Konaté B, Somé TD, Kambou SS. The state of the research for health environment in the ministries of health of the Economic Community of the West African States (ECOWAS). Health Res Policy Syst. 2013;11:35. doi:10.1186/1478-4505-11-35.
12. Mbondji PE, Kebede D, Zielinski C, Kouvividila W, Sanou I, Lusamba-Dikassa P-S. Overview of national health research systems in sub-Saharan Africa: results of a questionnaire-based survey. J R Soc Med. 2014;107 (IS) : 46–54.
13. Aidam J, Sombié I. The West African Health Organization’s experience in improving the health research environment in the ECOWAS region. Health Res Policy Syst. 2016;14:30. doi:10.1186/s12961-016-0102-7.
14. Kirigia JM, Ota MO, Motari M, Bataringaya JE, Mouhouelo P. National health research systems in the WHO African Region: current status and the way forward. Health Res Policy Syst. 2015;13:61. doi:10.1186/s12961-015-0054-3.
15. Kirigia JM, Kathyola DD, Muula AS, Ota MMO. National health research system in Malawi: dead, moribund, tepid or flourishing? BMC Health Serv Res. 2015;15:126. doi:10.1186/s12913-015-0796-1.
16. Changing Mindsets. Research Capacity Strengthening in Low and Middle Income Countries. Genève : COHRED, Forum mondial de la recherche et Programme spécial de recherche et de formation concernant les maladies tropicales UNICEF/PNUD/Banque mondiale/OMS; 2008.
17. COHRED. Frameowrk & Guides for System Strengthening. How the System Strengthening Approach Works. [http://www.cohred.org/research](http://www.cohred.org/research-innovation-system-strengthening-approach/)innovation-system-strengthening-approach/. Consulté le 27 décembre 2016.
18. Kirigia JM, Ota MO, Senkubuge F, Wiysonge CS, Mayosi BM. Developing the African national health research systems barometer. Health Res Policy Syst. 2016;14(1):53. doi:10.1186/s12961-016-0121-4.
19. COHRED. Partnership to Strengthen Health Research in 15 West African Countries. [http://www.cohred.org/2010/02/partnership-to-strengthen](http://www.cohred.org/2010/02/partnership-to-strengthen-health-research-in-15-west-african-countries/)health-research-in-15-west-african-countries/. Consulté le 27 décembre 2016.
20. Lanjouw S, Macrae J, Zwi AB. Rehabilitating health services in Cambodia: the challenge of coordination in chronic political emergencies. Health Policy Plan. 1999;14:229–42.
21. Fujita N, Zwi AB, Nagai M, Akashi H. A comprehensive framework for human resources for health system development in fragile and post-conflict states. PLoS Med. 2011;8(12):e1001146. doi:10.1371/journal.pmed.1001146.
22. Lee PT, Kruse GR, Chan BT, Massaquoi MB, Panjabi RR, Dahn BT, Gwenigale WT. An analysis of Liberia’s 2007 national health policy: lessons for health systems strengthening and chronic disease care in poor, post-conflict countries. Global Health. 2011;7:37. doi:10.1186/1744-8603-7-37.
23. Bertone MP, Samai M, Edem-Hotah J, Witter S. A window of opportunity for reform in post-conflict settings? The case of human resources for health policies in Sierra Leone, 2002-2012. Confl Health. 2014;8:11. doi:10.1186/ 1752-1505-8-11.
24. Wurie HR, Samai M, Witter S. Retention of health workers in rural Sierra Leone: findings from life histories. Hum Resour Health. 2016;14:3. doi:10. 1186/s12960-016-0099-6.
25. Gberie L. A Dirty War in West Africa. The RUF and the Destruction of Sierra Leone. Bloomington: Indiana University Press; 2005.
26. Kruk ME, Freedman LP, Anglin GA, Waldman RJ. Rebuilding health systems to improve health and promote state building in post-conflict countries: a theoretical framework and research agenda. Soc Sci Med. 2010;70:89–97.
27. Marais D, Sombié I, Becerra-Posada F, Montorzi G, de Haan S. Gouvernance, priorités, politiques des systèmes nationaux de recherche en santé de l’Ouest (Guinée-Bissau, Libéria, Mali, Sierra Leone). Genève : COHRED; 2011.
28. Kok MO, Rodrigues A, Silva AP, de Haan S. The emergence and current performance of a health research system: lessons from Guinea Bissau. Health Res Policy Syst. 2012;10:5. doi:10.1186/1478-4505-10-5.
29. CRDI. Renforcement de la recherche en santé en Afrique de l’Ouest. [https://www.idrc.ca/fr/project/renforcement-de-la-recherche-en-sante-en](https://www.crdi.ca/fr/project/renforcement-de-la-recherche-en-sante-en-afrique-de-louest)afrique-de-louest. Consulté le 27 janvier 2016.
30. Villanueva EC, Abreu DR, Cuervo LG, Becerra-Posada F, Reveiz L, Ijsselmuiden C. HRWeb Americas: a tool to facilitate better research governance in Latin America and the Caribbean. Cad Saude Publica. 2012;28(10):2003–8.
31. IJsselmuiden C, Marais D, Wassenaar D, Mokgatla-Moipolai B. Mapping African ethical review committee activity onto capacity needs: the MARC initiative and HRWeb’s interactive database of RECs in Africa. Dev World Bioeth. 2012;12(2):74–86. doi:10.1111/j.1471-8847.2012.00325.x.
32. OOAS/COHRED Renforcement de la recherche en santé en Afrique de l’Ouest. Évaluation du système de gestion de l’information sur la recherche. Premier rapport technique. Annexe 10. Bobo-Dioulasso : OOAS; 2011.
33. OOAS/COHRED Renforcement de la recherche en santé en Afrique de l’Ouest. Évaluation de la capacité du système de santé. Premier rapport technique. Annexe 11. Bobo-Dioulasso : OOAS; 2011.
34. OOAS Évaluation du Projet ouest-africain de Recherche pour la Santé OOAS-CRDI (2011-2014) et du Programme « Recherche » du Plan stratégique 2009-2013 de l’OOAS. Rapport final. Bobo-Dioulasso : OOAS; 2015.
35. Lansang MA, Dennis R. Building capacity in health research in developing world. Bull World Health Organ. 2004;82(10):764–70.
36. Organisation mondiale de la Santé – Bureau régional de l’Afrique Conférence des ministres sur la recherche sur la santé dans la région Afrique. La déclaration d’Alger. Brazzaville : OMS/AFRO; 2008.
37. Organisation mondiale de la Santé – Bureau régional de l’Afrique Déclaration de Libreville sur la Santé et l’Environnement en Afrique. Libreville, le 29 août 2008 Brazzaville : OMS/AFRO; 2008.
38. HRWeb de l’Afrique de l’Ouest <https://www.healthresearchweb.org/en/west_african_> project/west_africa. Consulté le 27 janvier 2016.
39. de Sardan JPO, Diarra A, Koné FY, Yaogo M, Zerbo R. Local sustainability and scaling up for user fee exemptions: medical NGOs vis-à-vis health system. BMC Health Serv Res. 2015;15 Suppl 3:S5.
40. Smith DJ. Patronage, per diems and the Workshop mentality”: the practice of family planning programs in Southeastern Nigeria. World Dev. 2003;31(4): 703–15.
41. Ridde V. Per diems undermine health interventions, systems and research in Africa: burying our heads in the sand. Trop Med Int Health. 2010. doi:10. 1111/j.1365-3156.2010.02607.x.
42. Conteh L, Kingori P. Per diems in Africa: a counter-argument. Trop Med Int Health. 2010;15 (12) : 1553–5.
43. Nkamleu G, Kamgnia B. Uses and abuses of per-diems in Africa: a political economy of travel allowances. Working Paper Series 196. Tunis : Banque de développement africaine; 2014.
44. Prytherch H, Kagone M, Aninanya GA, Williams JE, Kakoko DC, Leshabari MT, Ye M, Marx M, Sauerborn R. Motivation and incentives of rural maternal and neonatal health care providers: a comparison of qualitative findings from Burkina Faso, Ghana and Tanzania. BMC Health Serv Res. 2013;13:149.
45. Remme JHF, Adam T, Becerra-Posada F, D’Arcangues C, Devlin M, Gardner C, Ghaffar A, Hombach J, Kengeya JFK, Mbewu A, Mbizvo MT, Mirza Z, Pang T, Ridley RG, Zicker F, Terry RF. « Defining research to improve health systems ». PLoS Med. 2010;7:e1001000.
46. Tamura M, Hinderaker SG, Manzi M, Van Den Bergh R, Zachariah R. Severe acute maternal morbidity and associated deaths in conflict and post-conflict settings in Africa. Public Health Action. 2012;2(4):122–5. doi: 10.5588/pha.12.0036.
47. Warsame A. Opportunity for health systems strengthening in Somalia. Lancet Glob Health. 2014;2:e197–8.
48. Swanson RC, Atun R, Best A, Betigeri A, de Campos F, Chunharas S, Collins T, Currie G, Jan S, McCoy D, Omaswa F, Sanders D, Sundararaman T, Van Damme W. Strengthening health systems in low-income countries by enhancing organizational capacities and improving institutions. Global Health. 2015;11:5.
49. Percival V, Sondorp E. A case study of health sector reform in Kosovo. Confl Heal. 2010;4:7.
50. Cometto G, Fritsche G, Sondorp E. Health sector recovery in early post-conflict environments: experience from southern Sudan. Disasters. 2010;34:885–909.
51. Bertone MP, Witter S. The complex remuneration of human resources for health in low-income settings: policy implications and a research agenda for designing effective financial incentives. Hum Resour Health. 2015;13:62. doi:10.1186/s12960-015-0058-7.
52. O’Hanlon KP, Budosan B. Post-disaster recovery: a case study of human resource deployment in the health sector in post-conflict Kosovo. Prehosp Disaster Med. 2011;26(1):7–14.
53. Homan FF, Hammond CS, Thompson EF, Kollisch DO, Strickler JC. Post-conflict transition and sustainability in Kosovo: establishing primary healthcare-based antenatal care. Prehosp Disaster Med. 2010;25(1):28–33.
54. CATECH. Évaluation à mi-parcours du plan stratégique 2009-2013 de l’Organisation ouest-africaine de la santé (OOAS). Bobo-Dioulasso : OOAS; 2012.
55. West African Regional Diseases Surveillance Capacity Strengthening. http:// [www.worldbank.org/projects/P125018?lang=en](http://www.worldbank.org/projects/P125018?lang=en). Consulté le 27 décembre 2016.
56. Sahel Women’s Empowerment and Demographic Project. <http://projects>. [worldbank.org/P150080?lang=en](http://worldbank.org/P150080?lang=en). Consulté le 27 décembre 2016.
57. Regional Disease Surveillance Systems Enhancement (REDISSE) Project. <http://projects.worldbank.org/P154807?lang=en>. Consulté le 27 décembre 2016.
58. Joint communique final of a the Sub regional Ebola Virus Disease Collaboration Meeting among representatives researchers and scientists from the most affected countries - Guinea Liberia and Sierra Leone held in the city of Conakry, the Republic of Guinea, from 11-12 June 2015.
