# Supplementary material for: People and research: improved health systems for West Africans, by West Africans - report on special supplement
Source: BMC Proc. 2019 Feb 7;13(Suppl 1):1. doi: 10.1186/s12919-019-0162-0 (PMC6366023; doi:10.1186/s12919-019-0162-0)
Supplement: Supplementary file 9 — Comprendre l’état de la recherche sur les politiques et les systèmes de santé en Afrique occidentale et les besoins de renforcement des capacités: portée des tendances et des caractéristiques des publications à comité de lecture de 1990 à 2015, Defor, S., Kwamie, A., Agyepong, I. [file 12919_2019_162_MOESM9_ESM.docx]

***Comprendre l’état de la recherche sur les politiques et les systèmes de santé en Afrique occidentale et les besoins de renforcement des capacités : portée des tendances et des caractéristiques des publications à comité de lecture de 1990 à 2015***

**Selina Defor (1)*, Aku Kwamie (1), Irene Akua Agyepong (1)**

*Auteure-ressource

(1) Service de santé du Ghana, Division de la recherche et du développement

**Résumé**

**Contexte :**

Le besoin de données probantes gérées et produites localement pour orienter la prise de décision sur les politiques et les systèmes de santé et leur mise en oeuvre en Afrique occidentale reste urgent et la recherche sur les politiques et les systèmes de santé (RPSS) est un domaine ayant un grand potentiel pour relever les nombreux défis intransigeants en matière de santé de la sous-région. Cet article présente une analyse des tendances et des caractéristiques des publications à comité de lecture portant sur la RPSS à l’échelle de la Communauté économique des États de l’Afrique de l’Ouest (CEDEAO), afin d’aider à comprendre les tendances et les caractéristiques des publications de RPSS et le degré d’implication de chercheurs ouest-africains dans la production de données probantes de RPSS dans la sous-région Notre objectif était d’utiliser les résultats pour façonner l’élaboration d’une stratégie sous-régionale afin de renforcer la RPSS et son utilisation pour orienter l’élaboration et l’amélioration des résultats sur la santé.

**Méthodologies :**

Un examen de la portée a été effectué sur une période de 25 ans de janvier 1990 à septembre 2015. Des recherches dans la documentation ont été menées en anglais et en français en utilisant Google Scholar, PubMed Central et CAIRN.INFO.

**Résultats :**

Au total, 246 articles ont fait l’objet d’une analyse statistique sur les 258 qui ont été téléchargés. Cinquante-quatre pour cent de ces articles étaient principalement rédigés par des auteurs ouest-africains. Les deux tiers des articles provenaient de trois des quinze pays de la CEDEAO, spécifiquement le Nigéria (28,86 %), le Burkina Faso (21,54 %) et le Ghana (17,07 %). La plupart des auteurs étaient basés dans des établissements universitaires et la participation d’auteurs de ministères de la Santé, d’hôpitaux et d’ONG était limitée. L’anglais était la langue prédominante des publications même pour les articles provenant de l’Afrique occidentale francophone. Le nombre de publications a augmenté progressivement au cours de la période examinée.

**Conclusion :**

Malgré des améliorations régulières, l’Afrique occidentale reste une région pauvre en matière de publications à comité de lecture de RPSS. Dans cette faiblesse générale, il existe des écarts entre les pays. Le fait que seule une poignée de pays représente près de 70 % du nombre total de publications en Afrique occidentale témoigne des grandes disparités en capacités individuelles, institutionnelles et contextuelles pour la production de données probantes de RPSS. Combler l’écart entre les établissements principaux (universités et centres de recherche) et la communauté de praticiens (ministères, hôpitaux, ONG) est indispensable pour assurer une utilisation pratique des données probantes de la RPSS. Des investissements sont toujours nécessaires en matière de renforcement des capacités de RPSS en Afrique occidentale.

**Introduction**

Le besoin urgent de s’occuper des systèmes de santé faibles et fragiles pour atteindre de meilleurs résultats en santé a été au centre du programme mondial en matière de santé depuis plus de dix ans maintenant. Pourtant, la réussite des objectifs du Millénaire pour le développement (OMD) en Afrique occidentale a été sérieusement entravée par la faiblesse des systèmes de santé et qui fonctionnent mal, entre autres défis[^[[1]](#endnote-1)^]. Les systèmes de santé ont été définis comme étant composés de tous les acteurs, organismes, établissements et ressources dont l’objectif principal est d’améliorer la santé [^[[2]](#endnote-2),^^[[3]](#endnote-3)^]. Excepté cet objectif défini, le système de santé a d’autres buts intrinsèques : 1) être attentif à la population qu’il sert (cette attention est déterminée par la manière dont les personnes sont traitées et l’environnement dans lequel elles le sont); 2) assurer une répartition juste du fardeau financier du paiement en matière de santé.

Les systèmes de santé ont été décrits comme étant des systèmes adaptatifs complexes (SAC) étant donné qu’ils s’ajustent constamment de manière dynamique et imprévisible aux changements dans le système lui-même ou dans le contexte plus large dans lequel ils fonctionnent [^[[4]](#endnote-4)^]. Ce dynamisme souligne le fait qu’un système de santé prédéfini qui renforce les plans risque de devenir inefficace après un certain temps, et nécessite ainsi la production régulière de données probantes pour orienter l’élaboration de systèmes de santé nationaux et locaux. De plus, plusieurs études ont également souligné le fait que les données probantes émanant de la recherche peuvent améliorer l’élaboration de politiques en matière de santé en ce qui concerne l’identification des enjeux pour le programme de politique, la prise de décision relative aux politiques, l’évaluation des résultats des politiques, et ultimement l’orientation des efforts de renforcement des systèmes de santé [^[[5]](#endnote-5),^^[[6]](#endnote-6),^^[[7]](#endnote-7),^^[[8]](#endnote-8)^]. Le renforcement des systèmes de santé a été reconnu mondialement comme étant essentiel pour améliorer les résultats sur la santé, mais la base de connaissances pour appuyer cet effort dans les pays à faible revenu et les pays à revenu intermédiaire (PFR-PRI) a été plutôt restreinte.

La recherche sur les politiques et les systèmes de santé (RPSS) est définie comme un domaine visant à comprendre et à améliorer la façon dont les sociétés s’organisent pour réussir leurs objectifs collectifs en matière de santé, et la façon dont différents acteurs interagissent dans le processus de politiques et de mise en oeuvre pour contribuer aux résultats de politique [^[[9]](#endnote-9)^]. La RPSS répond aux questions sur les politiques et les systèmes de santé qui ne sont pas propres à une maladie, mais qui concernent des problèmes de systèmes qui ont des répercussions sur le rendement de tout le système de santé. La RPSS répond à un large éventail de questions, du financement de la santé, à la gouvernance, et aux politiques, jusqu’aux problèmes de structuration, de planification, de gestion, de ressources humaines, de prestation de services, de référencement, et de qualité des soins dans les secteurs public et privé. Naturellement, la RPSS est un domaine multidisciplinaire qui allie l’économie, la sociologie, l’anthropologie, les sciences politiques, la santé publique et l’épidémiologie pour dresser un portrait complet de la manière dont les systèmes de santé réagissent et s’adaptent aux politiques en matière de santé et de la manière dont ces politiques sont influencées par des systèmes de santé et des facteurs déterminants plus vastes de la santé. La RPSS se concentre sur la politique et favorise donc le travail qui cherche explicitement à influencer les politiques [^[[10]](#endnote-10),^^[[11]](#endnote-11)^]. Il convient de noter que l’intérêt mondial et la reconnaissance de l’importance de la RPSS ont été soulignés dans plusieurs rapports et événements des dernières années axés sur l’action [^[[12]](#endnote-12),^^[[13]](#endnote-13),^^[[14]](#endnote-14),^^[[15]](#endnote-15),^^[[16]](#endnote-16),^^[[17]](#endnote-17),^^[[18]](#endnote-18),^^[[19]](#endnote-19),^^[[20]](#endnote-20),^^[[21]](#endnote-21),^^[[22]](#endnote-22),^^[[23]](#endnote-23),^^[[24]](#endnote-24)^]. Selon certains auteurs, l’engagement envers la RPSS et son application se reflètent dans la montée récente de l’investissement international dans le domaine [^[[25]](#endnote-25)^]. Pendant ce temps, plusieurs autres études ont souligné à maintes reprises l’écart persistant entre les pays à revenu élevé (PRE) et les PFR-PRI en matière de production et d’utilisation de RPSS [^[[26]](#endnote-26),^^[[27]](#endnote-27),^^[[28]](#endnote-28)^]. Cette situation a incité certains auteurs à remettre en question l’effet d’entraînement de toutes les mesures mondiales à l’appui du développement de RPSS dans les PFR-PRI [^[[29]](#endnote-29),^^[[30]](#endnote-30),^^[[31]](#endnote-31)^].

L’Afrique occidentale, dont la population est estimée à environ 350 millions de personnes, comprend 15 pays (le Bénin, le Burkina Faso, le Cabo Verde, la Côte d’Ivoire, la Gambie, le Ghana, la Guinée, la Guinée-Bissau, le Libéria, le Mali, le Nigéria, le Niger, la Sierra Leone, le Sénégal, le Togo) qui sont tous classés comme étant des pays à faible revenu ou à revenu intermédiaire [^[[32]](#endnote-32)^]. La sous-région accueille une immense diversité de personnes, du point de vue des cultures, des langues et des religions. Les complexités de la sous-région sont superposées à la diversité traditionnelle ethnique, religieuse et linguistique qui sont exacerbées davantage par l’héritage colonial de la fragmentation de la sous-région selon la langue officielle en pays anglophone, francophone et lusophone.

Comparées à d’autres régions, les publications de RPSS en Afrique occidentale ont été nettement insuffisantes[^[[33]](#endnote-33)^]. La faible production de recherches en santé dans la sous-région a été attribuée non seulement à des capacités limitées en recherche et aux faibles capacités de formation [^[[34]](#endnote-34),^^[[35]](#endnote-35)^], mais également aux faibles capacités quant à la mobilisation des ressources, de même que la capacité limitée de développer des collaborations de recherche parmi les pays de la sous-région [^[[36]](#endnote-36),^^[[37]](#endnote-37)^]. Même si la RPSS a le potentiel de relever la plupart des défis intransigeants de la région, son état en Afrique occidentale n’a pas été systématiquement étudié.

Cette recherche visait donc à parcourir le paysage et à décrire les caractéristiques et les tendances des publications anglophones et francophones de RPSS à l’échelle des pays de la CEDEAO de 1990 à 2015. Nos objectifs précis étaient de décrire les tendances, les établissements, les personnes et les réseaux qui effectuent de la RPSS, et le degré d’implication de chercheurs sous conduite ouest-africaine dans la production de données probantes en RPSS dans la sous-région. Le but était d’utiliser les résultats afin d’orienter l’élaboration de stratégies pour renforcer la capacité de RPSS, menée et utilisée pour le renforcement des systèmes de santé et l’amélioration des résultats sur la santé au sein de la CEDEAO, y compris la mise en place de réseaux de RPSS. Cet article met l’accent sur l’analyse des établissements et des tendances, et nous ne présentons pas l’analyse des personnes et des réseaux.

**Méthodologies**

Notre méthodologie était un examen de la portée des publications à comité de lecture sur une période de près de 25 ans allant de janvier 1990 à septembre 2015. Les fondements théoriques sous-jacents à cette approche étaient fondés sur le cadre méthodologique en six étapes élaboré par Hilary Arksey et Lisa O’Malley qui définit un examen de la portée comme une « technique pour caractériser la documentation pertinente dans le domaine d’intérêt ». Selon ces auteures, l’examen de la portée est effectué par : 1) l’identification de la question de recherche, 2) la recherche des études pertinentes, 3) la sélection des études, 4) le déchiffrement des données, 5) la collecte, la synthèse et le compte rendu des résultats, 6) la consultation avec les intervenants pour éclairer ou valider les résultats [^[[38]](#endnote-38)^]. La méthode est décrite en tant qu’approche de synthèse des connaissances qui répond à une question exploratoire de recherche visant à schématiser les données probantes et les écarts en matière de recherche liée à un domaine donné par une recherche, une sélection et une synthèse systématiques des connaissances existantes [^[[39]](#endnote-39)^]. L’examen visait à déterminer et à recueillir les publications à comité de lecture de RPSS sur l’Afrique occidentale pour : comprendre les caractéristiques des publications de RPSS anglophones et francophones afin de cibler les personnes et les établissements qui effectuent de la RPSS, et pour vérifier le degré d’implication de chercheurs sous conduite ouest-africaine dans la production régionale de données probantes en RPSS. Par conséquent, l’examen de la portée a été considéré comme pertinent pour une étude dont le but est de caractériser la portée [^[[40]](#endnote-40)^] plutôt que la profondeur des données probantes dans le domaine de la RPSS en Afrique occidentale.

**Chercher et sélectionner les études pertinentes**

Des recherches dans la documentation ont été menées en anglais et en français en utilisant Google Scholar, PubMed Central et CAIRN.INFO. Les critères d’inclusion utilisés étaient des publications à comité de lecture de travaux effectués dans n’importe lequel des quinze États membres de la CEDEAO, publiées en anglais ou en français, dont les objectifs ou les mots clés comprenaient explicitement la mention de systèmes de santé d’un ou de plus d’un élément constitutif des systèmes de santé établis de l’OMS. À l’exception de Cabo Verde et de la Guinée-Bissau, qui sont lusophones, les treize autres pays de la CEDEAO sont anglophones ou francophones. Cependant, nous n’avions pas l’expertise pour examiner la documentation en portugais.

En anglais, les termes de recherche utilisés étaient initialement limités à « health systems » ET « nom du pays de la CEDEAO », à « Health Policy Systems Research » ET « West Africa » ou « nom du pays de la CEDEAO » et à « health policy » ET « nom du pays de la CEDEAO » Plus tard, ils ont été élargis pour comprendre « Health Care Financing » ET « West African country », « Health Service delivery » ET « West African country », « Health Leadership » OU « Governance » ET « West African country », « Health Information Systems » ET « West African country », « Health Care » ET « Human resource » ET « West African country ». En français, les termes de recherche utilisés étaient : Prestations services + soin de santé + Afrique de l’Ouest/un pays de l’Afrique de l’Ouest, Accès +soin + Afrique de l’Ouest, Accès +soin + un pays de l’Afrique de l’Ouest, Personnels de santé + Afrique de l’Ouest/un pays de l’Afrique de l’Ouest, Politiques pharmaceutiques +Afrique de l’Ouest /un pays de l’Afrique de l’Ouest, système d’information santé +Afrique de l’Ouest/un pays de l’Afrique de l’Ouest.

**Figure 1 : Organigramme de l’inclusion**

La figure ci-dessous illustre l’organigramme de l’inclusion des articles de l’examen de la portée.

Nombre total d’articles récupérés de juillet 2014 à septembre 2015 = 262

Nombre d’articles exclus = 4

Nombre d’articles téléchargés = 258

Nombre d’articles analysés d’un point de vue statistique = 246

Articles publiés avant 1990 (1977) = 1

Articles de nature plus clinique = 3

12 articles rejetés pour des raisons d’erreur de saisie de données

Articles en français = 37

Articles en anglais = 209

**Déchiffrer les données, recueillir, résumer et faire un compte rendu des résultats**

Tous les articles admissibles ont été téléchargés et sauvegardés dans EndNote. Les données ont été compilées et importées dans Microsoft Excel pour la validation et le chiffrage. Pour chaque publication comprise dans l’étude, nous avons extrait les variables suivantes : la langue de la publication, l’année de la publication, l’endroit de l’étude, le pays dans lequel l’établissement principal de l’auteur principal et des coauteurs était basé, le type d’établissement de l’auteur principal et des coauteurs (université, institut ou centre de recherche, hôpital, organisation non gouvernementale), et le nombre d’auteurs principaux ouest-africains. Nous avons effectué certaines des analyses dans Excel et exporté les données dans la version 12 de Stata pour une analyse supplémentaire.

**Consulter les intervenants pour éclairer ou valider les résultats**

La consultation avec les intervenants pour valider les résultats a été menée lors de deux réunions sous-régionales. La première était une réunion de consultation avec des chercheurs principaux en santé de la CEDEAO concernant la validation de plan stratégique de recherche en santé organisée par l’Organisation ouest-africaine de la santé (OOAS) à Abidjan en février 2015. Des chercheurs principaux d’universités, d’instituts de recherche en santé et de ministères de la Santé à l’échelle de la sous-région de la CEDEAO étaient présents à la réunion. La seconde réunion de validation était la réunion de lancement des jeunes chercheurs ouest-africains sur les politiques et les systèmes de santé et du réseau de soutien des exécutants connu sous le nom de West African Network of Emerging Leaders (WANEL) organisé à Accra en juin 2015.

De juin à septembre 2015, la recherche s’est poursuivie après la seconde réunion de validation. La prolongation de l’examen et l’ajout des six éléments constitutifs des systèmes de santé établis par l’OMS et leurs sous-éléments pour définir la portée de l’examen sont attribuables aux commentaires recueillis lors des deux réunions de validation avec les intervenants. La recherche initiale, sauf le fait qu’elle s’arrête en 2014, avait été limitée de près à « health systems » OU « health policy » ET « nom du pays de la CEDEAO ». Cette approche limitée a permis de trouver seulement 65 publications. Dans les deux ateliers de validation, les répondants ont fait remarquer des articles manquants. Afin d’être impartial, puisque tous les chercheurs possibles n’étaient pas membres de ces réunions de validation, il n’a pas été jugé souhaitable d’ajouter les articles signalés comme manquants, sans s’assurer qu’ils répondaient à un critère de recherche inclusif et défini objectivement. L’élargissement des termes de recherche a fait augmenter le nombre d’articles à 262. Cela a permis de traiter pour la plupart des enjeux concernant des articles manquants. Cependant, aux fins d’impartialité dans l’analyse comparative et des tendances, nous avons retenu 246 articles, tout en reconnaissant qu’il ne s’agit pas d’une liste exhaustive.

**Considérations éthiques**

Il s’agissait ici d’une étude documentaire de documents déjà publiés dans le domaine public. Aucune collecte ou analyse de données primaires n’a été effectuée. Il n’y a donc pas eu lieu de solliciter l’approbation sur le plan de l’éthique ou le consentement éclairé.

**RÉSULTATS**

Après avoir vérifié le titre, les mots clés et le résumé des articles extraits par notre recherche, nous avons compris dans l’analyse 246 articles provenant de 14 des 15 pays d’Afrique de l’Ouest.

**Tendances dans les publications**

La figure 2 illustre le nombre total de publications par année pour la période examinée. Le nombre presque fixe de publications par année commence à afficher une augmentation lente du nombre de publications par année à partir de 2001, qui s’accélère à partir de 2006.

**Figure 2 Tendances dans les publications à comité de lecture qui mettent l’accent sur les systèmes de santé d’Afrique de l’Ouest**


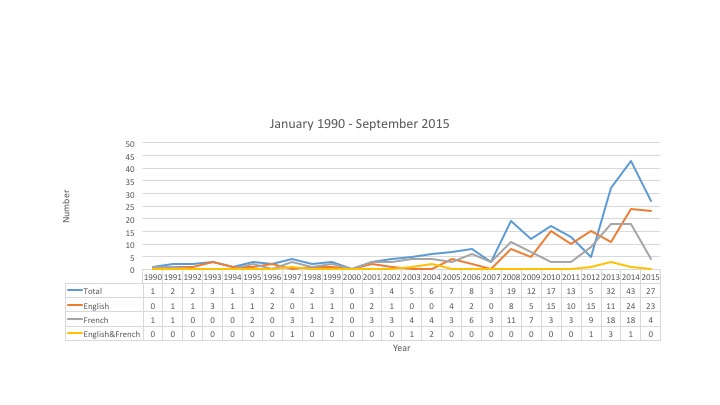


**Publication par emplacement des pays de l’étude**

Cette augmentation des publications est cependant due à un nombre élevé de publications de quelques pays uniquement. Pendant la période examinée, tous les pays d’Afrique occidentale ont présenté au moins un article lié à la RPSS. À l’exception du Burkina Faso, qui se démarque considérablement, les cinq pays anglophones d’Afrique de l’Ouest semblent publier davantage que la majorité des pays francophones d’Afrique de l’Ouest. Le Nigéria était le pays produisant le plus grand nombre de publications avec une quantité équivalant à 28,86 % (n = 71), suivi du Burkina Faso avec 21,54 % (n = 53) et du Ghana avec 17,07 % (n = 42). À eux seuls, le Nigéria, le Ghana et le Burkina Faso représentent plus des deux tiers des articles extraits.

Lorsque les publications de la période examinée sont liées à la population en milieu d’année de 2015, la différence est marquée entre les pays anglophones et francophones en matière de quantité de publications par rapport à la population. Le Burkina Faso a le plus grand nombre de publications par million de personnes. À cet égard, le Nigéria se situe presque en fin de liste. Certains des plus petits pays comme la Sierra Leone et la Gambie affichent une quantité relativement élevée. (Voir le tableau 1)

**Tableau 1 : Publications par pays sur 25 ans par 1 000 000 de la population estimée de 2015**

| Population de 2015 estimée | en milieu d’année | % de la population de 2015 | Publications | Par 1 000 000  de la population estimée de 2015 |
| --- | --- | --- | --- | --- |
| Nigéria | 183 523 432 | 53 % | 70 | 0,381 |
| Ghana | 26 984 328 | 8 % | 41 | 1,519 |
| Côte d’Ivoire | 21 295 284 | 6 % | 6 | 0,282 |
| Niger | 19 268 380 | 6 % | 6 | 0,311 |
| Burkina Faso | 17 914 625 | 5 % | 53 | 2,958 |
| Mali | 16 258 587 | 5 % | 11 | 0,677 |
| Sénégal | 14 967 446 | 4 % | 9 | 0,601 |
| Guinée | 12 347 766 | 4 % | 3 | 0,243 |
| Bénin | 10 879 828 | 3 % | 10 | 0,919 |
| Togo | 7 170 797 | 2 % | 1 | 0,139 |
| Sierra Leone | 6 318 575 | 2 % | 11 | 1,741 |
| Libéria | 4 503 439 | 1 % | 3 | 0,666 |
| Gambie | 1 970 081 | 1 % | 3 | 1,523 |
| Guinée-Bissau | 1 787 793 | 1 % | 1 | 0,559 |
| Cabo Verde | 508 315 | 0 % | 0 | 0,000 |
| Population totale en AO | 345 698 676 | 100 % |  | 0,000 |
| Plusieurs d’Afrique de l’Ouest |  |  | 13 | 0,038 |
| Plusieurs d’Afrique |  |  | 4 | 0,012 |
| Plusieurs à l’international |  |  | 1 | 0,003 |
|  |  |  | 246 | 0,712 |

**Auteur principal et pays de rattachement**

L’auteur principal de 54 % (n = 132) des articles était ouest-africain. Le tableau 2 résume les auteurs principaux ouest-africains par pays. Cependant, il y avait de nombreux cas où même si l’auteur principal était ouest-africain, le rattachement principal était un établissement non ouest-africain. Cela explique le nombre élevé d’établissements principaux non ouest-africains comparé aux auteurs principaux ouest-africains. Cette situation semble être généralement causée par le fait qu’ils étaient inscrits en tant que chercheur au doctorat dans les établissements du Nord.

**Tableau 2 : Auteurs principaux ouest-africains par pays dans lequel la recherche a été menée**

| **Pays** | **N^o^ d’articles** | **N^o^ comportant un**  **auteur principal ouest-africain** | **% comportant un**  **auteur principal ouest-africain** |
| --- | --- | --- | --- |
| Bénin | 10 | 8 | 80 % |
| Burkina Faso | 53 | 14 | 26 % |
| Côte d’Ivoire | 6 | 5 | 83 % |
| Gambie | 3 | 1 | 33 % |
| Ghana | 42 | 31 | 74 % |
| Guinée | 3 | 0 | 0 % |
| Guinée-Bissau | 1 | 0 | 0 % |
| Libéria | 2 | 0 | 0 % |
| Mali | 12 | 1 | 8 % |
| Niger | 5 | 3 | 60 % |
| Nigéria | 71 | 59 | 83 % |
| Sénégal | 9 | 5 | 56 % |
| Sierra Leone | 11 | 0 | 0 % |
| Togo | 1 | 0 | 0 % |
| Plusieurs pays d’Afrique de l’Ouest | 14 | 5 | 36 % |
| Plusieurs pays d’Afrique | 2 | 0 | 0 % |
| Plusieurs pays à l’international | 1 | 0 | 0 % |
| Total | 246 | 132 | 54 % |

**Établissement ou rattachement de l’auteur principal**

Le tableau 3 résume l’établissement ou le rattachement de l’auteur principal de même que le pays dans lequel l’établissement est situé. Les cinq établissements ou rattachements les plus courants des auteurs principaux étaient l’Université de Montréal (8,5 %), l’Université de Heidelberg (7,3 %), l’Université du Ghana et l’Université du Nigéria (4,1 % chacune), l’Université John Hopkins (2,8 %) et l’Université Columbia (2 %).

La plupart des auteurs principaux (162/246) étaient rattachés à une université. Parmi les universités, 62 % d’entre elles (100/162) se trouvaient dans les pays du Nord et les autres, 38 %, en Afrique de l’Ouest. Les universités du Nord étaient dominées par l’Université de Montréal, qui menait dans 21 des 100 (21 %) principaux auteurs d’établissements du Nord, suivie de l’Université de Heidelberg (18/100). Les universités d’Afrique de l’Ouest étaient dominées par l’Université du Ghana (10/63) et l’université du Nigéria (10/63).

Lorsque les universités du Nord et celles de l’Afrique de l’Ouest sont unies dans l’analyse, les cinq universités qui se trouvent au sommet de la liste en tant que rattachement des auteurs principaux étaient l’Université de Montréal (14 %), l’Université de Heidelberg (11 %), l’Université du Ghana (6 %), l’Université du Nigéria (6 %) et l’École de santé publique John Hopkins (4 %).

Le deuxième plus grand groupe en importance était les instituts de recherche (37/246). La plupart de ces instituts de recherche (73 % ou 27/37) étaient en Afrique de l’Ouest tandis que les autres se trouvaient dans le Nord. Cette situation est l’inverse de celle des universités. L’Institut de médecine tropicale à Anvers était l’institut d’auteurs principaux pour quatre des dix auteurs principaux d’instituts de recherche du Nord. Parmi les instituts de recherche d’Afrique occidentale, le Centre de recherche en santé de Navrongo au Ghana et le Centre de Recherche en Santé de Nouna au Burkina Faso menaient en ce qui concerne les publications avec six publications chacun. L’Institut régional de santé publique au Bénin suivait avec quatre publications. La culture des instituts de recherche semble être particulièrement forte au Ghana et au Burkina Faso.

À elles seules, les universités représentent plus des deux tiers du nombre total de publications, quoique la pertinence de publication de RPSS dépend de sa capacité à influencer les politiques et la pratique. Les praticiens semblent moins impliqués dans la production de connaissances de RPSS en Afrique de l’Ouest. Une plus grande implication de dirigeants de ministères de la Santé et d’organismes gouvernementaux et même d’ONG dans la production de données probantes en RPSS doit être encouragée à l’échelle de la sous-région. Les autres pays de la sous-région pourront en apprendre beaucoup du Ghana à cet égard (dix publications des services de santé du Ghana, dont six du Centre de recherche en santé de Navrongo, et quatre provenant d’autres sections du service). Les publications du Ghana du service de santé étaient généralement menées par des chercheurs ghanéens. Le Burkina Faso mène également un peu dans ce domaine malgré la forte dominance de chercheurs du Nord dans les articles venant du Burkina Faso.

**Table 3 – Détails des établissements des auteurs principaux**

| **Institution of lead author** | **Country of institution** | **Number** | **% of all institutions** |
| --- | --- | --- | --- |
| SOUTHERN (WEST AFRICAN) UNIVERSITIES |  |  |  |
| University of Ghana | Ghana | 10 | 4.1% |
| University of Nigeria | Nigeria | 10 | 4.1% |
| University of Calabar | Nigeria | 4 | 1.6% |
| Nnamdi Azikiwe University | Nigeria | 4 | 1.6% |
| University of Lagos | Nigeria | 3 | 1.2% |
| University of Ibadan | Nigeria | 3 | 1.2% |
| Ahmadu Bello University | Nigeria | 2 | 0.8% |
| Cheikh Anta Diop University | Senegal | 2 | 0.8% |
| Ebonyi State University | Nigeria | 2 | 0.8% |
| L'Universite de Ouagadougou | Burkina Faso | 1 | 0.4% |
| Olabisi Onabanjo University | Nigeria | 2 | 0.8% |
| Nnamdi Azikiwe University | Nigeria | 3 | 1.2% |
| Bayero University | Nigeria | 1 | 0.4% |
| Ekiti State University | Nigeria | 1 | 0.4% |
| Kwame Nkrumah University of Science and Technology | Ghana | 1 | 0.4% |
| Niger Delta University | Nigeria | 1 | 0.4% |
| ObafemiAwolowo University | Nigeria | 1 | 0.4% |
| University of Abomey-Calavi | Benin | 1 | 0.4% |
| Federal University of Agriculture | Nigeria | 1 | 0.4% |
| University of Benin Teaching Hospital | Benin | 1 | 0.4% |
| University of Cape Coast | Ghana | 1 | 0.4% |
| University of Cocody | Cote d'Ivoire | 2 | 0.8% |
| Delta State University | Nigeria | 1 | 0.4% |
| University of Development studies | Ghana | 1 | 0.4% |
| University of Ilorin | Nigeria | 1 | 0.4% |
| University of Jos | Nigeria | 1 | 0.4% |
| University of Sokoto, Nigeria | Nigeria | 1 | 0.4% |
|  |  | 62 | 25.2% |
| NOTHERN UNIVERSITIES |  |  |  |
| Universite de Montreal/University of Montreal | Canada | 21 | 8.5% |
| University of Heidelberg | Germany | 18 | 7.3% |
| John Hopkins University | USA | 7 | 2.8% |
| Colombia University | USA | 5 | 2.0% |
| London School of Hygiene & Tropical Medicine | UK | 4 | 1.6% |
| The University of Western Ontario | Canada | 4 | 1.6% |
| Norwegian University of Science and Technology | Norway | 3 | 1.2% |
| University of Aberdeen | UK | 3 | 1.2% |
| University of California | USA | 3 | 1.2% |
| London School of Economics and Political Science | UK | 2 | 0.8% |
| University of Ottawa | Canada | 2 | 0.8% |
| University of Washington | USA | 2 | 0.8% |
| King's College London | UK | 2 | 0.8% |
| Radboud University Nijmegen | Netherlands | 2 | 0.8% |
| Université Claude Bernard | France | 2 | 0.8% |
| Liverpool School of Tropical Medicine | UK | 2 | 0.8% |
| Ernst-Moritz-Arndt-Universität Greifswald (University of Greifswald) | Germany | 2 | 0.8% |
| University of Illinois | USA | 1 | 0.4% |
| Aarhus University | Denmark | 1 | 0.4% |
| Institut d'etudes politiques de Grenoble | France | 1 | 0.4% |
| Graduate Institute of International and Development Studies | Switzerland | 1 | 0.4% |
| McGill University | Canada | 1 | 0.4% |
| Université Catholique de Louvain | Belgium | 1 | 0.4% |
| University of Alabama | USA | 1 | 0.4% |
| University of Birmingham | UK | 1 | 0.4% |
| University of North Carolina | USA | 1 | 0.4% |
| University of Sydney | Australia | 1 | 0.4% |
| University of Toronto | Canada | 1 | 0.4% |
| University of Western Australia | Australia | 1 | 0.4% |
| Hebrew University, Jerusalem | North | 1 | 0.4% |
| Vrije Universitat Amsterdam | Netherlands | 1 | 0.4% |
| Concordia University | Canada | 1 | 0.4% |
| Yale School of Public Health | USA | 1 | 0.4% |
|  |  | 100 | 40.7% |
| NORTHERN RESEARCH INSTITUTES AND GROUPS (NON UNIVERSITY) |  |  |  |
| Institut français de recherche scientifique pour le développement en coopération | North | 1 | 0.4% |
| Centre de Recherche Warocqué | Belgium | 1 | 0.4% |
| Institute for Medical Technology Assessment | Netherlands | 1 | 0.4% |
| Institute of Development Studies | UK | 1 | 0.4% |
| Institute of Tropical Medicine-Antwerp | Belgium | 4 | 1.6% |
| Memorial Sloan-Kettering Cancer Center | USA | 1 | 0.4% |
| Royal Tropical Institute | Netherlands | 1 | 0.4% |
|  |  | 10 | 4.1% |
| SOUTHERN RESEARCH INSTITUTES AND GROUPS (NON UNIVERSITY) |  |  |  |
| Centre Muraz | Burkina Faso | 6 | 2.4% |
| Navrongo Health Research Centre | Ghana | 6 | 2.4% |
| Institut régional de santé publique | Benin | 4 | 1.6% |
| Centre de Recherche en Santé de Nouna | Burkina Faso | 2 | 0.8% |
| **Laboratoire d'Etudes et de Recherche sur les Dynamiques Sociales et le Développement Local** | Niger | 2 | 0.8% |
| African Population and Health Research institute | Kenya | 1 | 0.4% |
| Consortium de Recherche en Economie Sociale | Senegal | 1 | 0.4% |
| Ecole Nationale Supérieure de Statistiques et d'Economie Appliquée | Cote d'Ivoire | 1 | 0.4% |
| Institut de recherche en sciences de la santé | Burkina Faso | 1 | 0.4% |
| Institut National de Recherches en Santé Publique | Mali | 1 | 0.4% |
| Institut de Recherche en Science de la Santé | Burkina Faso | 1 | 0.4% |
| Public Health Research & Development Centre | Gambia | 1 | 0.4% |
|  |  | 27 | 11.0% |
| NORTHERN INSTITUTES - ALL OTHERS |  |  |  |
| Center for Disease Control , Atlanta | USA | 4 | 1.6% |
| Africa Development Bank | Multilateral | 3 | 1.2% |
| Abt Associates | USA | 3 | 1.2% |
| Astellas Pharma UK Ltd | UK | 2 | 0.8% |
| ICF International | USA | 2 | 0.8% |
| United Nations Children Emergency Fund | Multilateral | 2 | 0.8% |
| Massachusetts General Hospital | USA | 1 | 0.4% |
| German Development Cooperation | Germany | 1 | 0.4% |
| United States Peace Corps | USA | 1 | 0.4% |
| World Bank | Multilateral | 1 | 0.4% |
| Institut de Veille sanitaire | France | 1 | 0.4% |
| Family Care International | USA | 1 | 0.4% |
| Helen Keller International | USA | 1 | 0.4% |
| Save the Children | USA | 1 | 0.4% |
| Surgeons OverSeas | USA | 1 | 0.4% |
| University Research Co. | USA | 1 | 0.4% |
|  |  | 26 | 10.6% |
| SOUTHERN INSTITUTES - ALL OTHERS |  |  |  |
| Ghana Health Service | Ghana | 4 | 1.6% |
| National Primary Health Care Development agency | Nigeria | 2 | 0.8% |
| Agogo Presbyterian Hospital | Ghana | 1 | 0.4% |
| Aminu Kano Teaching Hospital | Nigeria | 1 | 0.4% |
| Federal Medical Centre | Nigeria | 1 | 0.4% |
| Institut National de Santé Publique | Cote d'Ivoire | 1 | 0.4% |
| Irrua Specialist Teaching Hospital | Nigeria | 1 | 0.4% |
| Komfo Anokye Teaching Hospital | Ghana | 1 | 0.4% |
| National Hospital | Nigeria | 1 | 0.4% |
| Federal Ministry of Health, Abuja | Nigeria | 1 | 0.4% |
| Ministère de la Santé et de l’Hygiène Publique | Cote d'Ivoire | 1 | 0.4% |
| Ministère de la santé publique/Mission de coopération | Niger | 1 | 0.4% |
| Burkinabe Public Health Association | Burkina Faso | 1 | 0.4% |
| Cellule d’Analyse et de Prospective en Développement | Niger | 1 | 0.4% |
| Partnership for Reviving Routine Immunisation in Northern-Maternal Newborn and Child Health | Nigeria | 1 | 0.4% |
| Mediplan healthcare limited | Nigeria | 1 | 0.4% |
| Nigeria Reinsurance Corporation | Nigeria | 1 | 0.4% |
|  |  | 21 | 8.5% |
|  |  |  |  |
| Total |  | 246 |  |

**Tableau 4 : Résumé des établissements des auteurs principaux**

| Type d’établissement | Lieu | | |
| --- | --- | --- | --- |
|  | Nord  (% des types d’établissements) | Sud  (% des types d’établissements) | Total  (% de tous les établissements) |
| Organisme gouvernemental | 6 (40 %) | 9 (60 %) | 15 (6 %) |
| Hôpital | 1 (14 %) | 6 (86 %) | 7 (3 %) |
| International bilatéral | 1 (100 %) | 0 (0 %) | 1 (0,4 %) |
| International multilatéral | 6 (100 %) | 0 (0 %) | 6 (2 %) |
| ONG/Privé | 11 (73 %) | 4 (27 %) | 15 (6 %) |
| Privé (autre) | 1 (33 %) | 2 (67 %) | 3 (1 %) |
| Institut de recherche | 10 (27 %) | 27 (73 %) | 37 (15 %) |
| Université | 100 (62 %) | 62 (38 %) | 162 (66 %) |
| Total | 136 (55 %) | 110 (45 %) | 246 (100 %) |

**Langue de publication**

Même si le français est largement parlé dans la sous-région, la plupart des publications (84,96 %) étaient en anglais. Ce nombre comprend les publications de travaux effectués dans les pays francophones, et la seule publication de la Guinée-Bissau.

**Discussion**

Cette étude confirme l’observation d’un intérêt accru mondialement pour la RPSS à compter de 2008[^[[41]](#endnote-41)^]. De façon similaire, le taux de publication au sein de la CEDEAO a commencé à augmenter considérablement à partir de 2008. Cette augmentation de publications à comité de lecture peut être attribuée à plusieurs facteurs. Nous émettons l’hypothèse que le Sommet de Mexico de 2004, la première discussion ministérielle de haut niveau qui a remis la santé mondiale à l’ordre du jour, pourrait avoir contribué à cette augmentation. Les séries de forums mondiaux sur la recherche en santé, y compris leurs documents de résultats axés sur l’action et les activités qui ont menés au deuxième Forum ministériel en 2008, suivi peu de temps après par le premier Symposium mondial sur la recherche sur les systèmes de santé en 2010, pourraient toutes avoir accru l’intérêt et la reconnaissance de la valeur de la RPSS. Un total de 33 articles ont été publiés entre 1991 et 2003, les 12 années précédant le Sommet de Mexico de 2004. La situation s’est améliorée grâce à une augmentation par six des publications au cours de la période de 12 ans qui a suivi, de 2004 à 2015, avec un total de 212 publications.

De multiples actions et événements mondiaux qui ont mis l’accent sur l’importance de la recherche sur la santé pour le renforcement des systèmes de santé afin d’améliorer les résultats sur la santé peuvent également avoir contribué à la situation. Ce sont surtout les Forums mondiaux pour la recherche en santé et les appels à l’action [^[[42]](#endnote-42),^^[[43]](#endnote-43),^^[[44]](#endnote-44)^] la Stratégie OMS de recherche pour la santé [^[[45]](#endnote-45)^], et la publication de la stratégie de recherche 2008-2013 du DFID du Royaume-Uni, qui mettaient l’accent sur le besoin de capacité en recherche pour que les pays africains puissent déterminer leurs priorités en matière de besoins en santé et s’en occuper en conséquence [^[[46]](#endnote-46)^]. En reconnaissant le besoin de développer la capacité de recherche multidisciplinaire au niveau national, l’Union européenne a procédé à des investissements importants dans la recherche menée par les pays et dans les efforts de production de connaissances. Grâce à un budget de 6 milliards d’euros en vertu du septième programme-cadre, l’Union européenne a encouragé les collaborations de recherche avec les PFR-PRI pour des programmes coopératifs en recherche sur la santé et a défini la recherche sur l’optimisation de la prestation des soins de santé comme un des trois domaines prioritaires [^[[47]](#endnote-47)^].

Bien que le nombre de publications de RPSS ait régulièrement augmenté en Afrique de l’Ouest pour la période examinée, elle demeure minime, et l’écart de longue date persiste entre les PFR-PRI et les PRE. En raison d’une quantité de publications équivalant à 212 de 2004 à 2015, l’Afrique de l’Ouest traîne encore loin derrière d’autres régions. Un exercice semblable de constitution d’une liste de RPSS effectué dans la région de la Méditerranée orientale sur une période de huit ans (2000 à 2008) a montré un nombre de publications correspondant à 1 489. Dans cette région, le pays ayant produit le moins de publications (Yémen) a présenté la même quantité (n = 71) que le pays ayant produit le plus de publications (Nigéria; n = 71) en Afrique de l’Ouest. Une étude qui a examiné les tendances dans les publications internationales en RPSS a conclu qu’aux taux actuels de publications, il faudra plus de 42 ans pour que les publications de RPSS du Sud atteignent le taux actuel du Nord [^[[48]](#endnote-48)^]. Plusieurs facteurs pourraient avoir contribué à cet écart persistant en quantité de RPSS dans les PFR-PRI.

En premier lieu, les contraintes en matière de ressources financières, particulièrement le manque de financement national pour la recherche sur la santé, ont généralement été perçues comme étant un des facteurs importants qui ralentissait la production de RPSS dans les PFR-PRI [^[[49]](#endnote-49)^] et cela est vrai aussi pour l’Afrique de l’Ouest. Malgré tous les engagements internationaux visant à augmenter les ressources nationales pour la recherche en santé, très peu de ressources nationales sont appliquées à tout type de recherche sur la santé dans la sous-région, y compris la RPSS [^[[50]](#endnote-50)^]. Un sondage réalisé par l’OOAS a révélé que seul un tiers des pays d’Afrique de l’Ouest a mis en place des stratégies pour mettre en oeuvre la recommandation internationale pour l’allocation de 2 % du budget de ministères de la Santé et de 5 % des budgets pour des projets de recherche. La plupart des documents relatifs au budget de ministères de la Santé vérifiés n’avaient aucune ligne budgétaire pour appuyer des projets de recherche du mécanisme de recherche sur la santé au sein du ministère de la Santé [^[[51]](#endnote-51)^]. Plusieurs études ont documenté le fait que l’aide internationale multilatérale et bilatérale est la principale source de financement pour la RPSS par l’entremise de subventions de projets dans les PFR-PRI [^[[52]](#endnote-52),^^[[53]](#endnote-53),^^[[54]](#endnote-54)^]. Cependant, les établissements des PFR-PRI sont moins susceptibles de recevoir du financement de base que les établissements basés dans les PRE [^[[55]](#endnote-55)^]. Les ressources de donneurs externes sont une source potentielle de financement de la RPSS pour les États membres de la CEDEAO, mais la capacité d’élaborer des propositions solides pour se battre afin d’obtenir ces fonds est malheureusement également faible en Afrique de l’Ouest, ce qui limite les possibilités de puiser dans les occasions de concours internationaux de financement pour la RPSS [^[[56]](#endnote-56)^].

En deuxième lieu, la RPSS est propre au contexte et nécessite des acteurs locaux possédant une compréhension et une appréciation de leurs propres défis en matière de systèmes de santé afin de stimuler les processus de production pour les systèmes et les politiques de santé et en bout de ligne d’amélioration des résultats sur la santé. Cela peut seulement être le cas à condition que les acteurs locaux aient les compétences requises. La capacité fait référence à la faculté des personnes, des établissements et des sociétés à exercer des fonctions, à résoudre des problèmes, et à établir et à réussir des objectifs de manière durable [^[[57]](#endnote-57)^]. Plus particulièrement, la capacité en RPSS nécessite l’expertise et les ressources au niveau des chercheurs, des projets et des établissements afin de produire de nouvelles connaissances et applications pour l’amélioration de la réponse sociale envers les problèmes de santé [^[[58]](#endnote-58)^]. Malheureusement, plusieurs études ont mis l’accent sur la faible capacité, voire inexistante, en matière d’activités de RPSS dans les PFR-PRI [^[[59]](#endnote-59),^^[[60]](#endnote-60)^]. Le manque de capacité pour produire de la RPSS en Afrique de l’Ouest est clairement étayé dans cet examen sur la portée. La majorité des pays produisent des quantités négligeables de publications de RPSS. Les quelques pays qui produisent un bon nombre de publications doivent cela à des auteurs qui ne sont pas ouest-africains ou à des auteurs ouest-africains qui sont rattachés à des établissements situés à l’extérieur de la région.

En troisième lieu, hormis la faible capacité des personnes et des établissements, le contexte plus large dans lequel les chercheurs locaux interviennent donne un effet de dissuasion pour la recherche en santé en général, y compris la RPSS. Une étude menée par l’OOAS a également révélé que seuls 50 % des pays d’Afrique occidentale ont des directions au sein de leur ministère de la Santé pour surveiller la recherche en santé, de même que des documents stratégiques qui présentent les priorités en matière de recherche sur la santé qui comprennent des questions liées aux systèmes de santé [^[[61]](#endnote-61)^]. Seuls cinq pays étaient dotés de comités d’éthiques composés de membres formés en éthique de la recherche. De plus, les représentants du gouvernement en santé manquaient de capacités suffisantes pour appuyer l’application des résultats de recherche aux politiques et aux pratiques, ce qui limite donc l’utilité des données probantes produites. Cela a freiné encore plus l’enthousiasme envers la production de données probantes supplémentaires.

À l’évidence, la capacité en RPSS en Afrique de l’Ouest doit être renforcée. Il n’existe pas beaucoup de données sur l’initiative la plus efficace concernant le renforcement des capacités; cependant, diverses formes de collaborations dans les pays, entre les pays, intrarégionales et internationales ont aidé à renforcer la capacité en recherche et ont augmenté la productivité en recherche dans plusieurs PFR-PRI, y compris certains des pays les plus prospères en Afrique de l’Ouest, et la sous-région entière peut tirer des enseignements de ces collaborations. Par exemple, la Medical Education Partnership Initiative au Nigéria (MEPIN), un consortium de six universités nigériennes travaillant en collaboration avec deux universités américaines, ont développé la capacité de recherche de plus de 1 600 facultés, étudiants de cycle supérieur et médecins résidents entre 2011 et 2013 par l’entremise d’un programme de formation des formateurs. Les partenaires américains forment les personnes-ressources dans les pays, environ six dans chaque université membre qui sont dans neuf cours différents, qui à leur tour, reproduisent les ateliers dans leurs différents établissements dans le cadre d’un programme régulier de développement de carrière, en utilisant le même matériel de formation. Cette capacité améliorée a non seulement entraîné un nombre accru de publications dans les revues à comité de lecture, mais elle a également augmenté les réponses aux demandes et aux octrois de subventions locales et internationales[^[[62]](#endnote-62)^]. De plus, une collaboration entre la Thaïlande et l’Afrique du Sud avec un appui du Royaume-Uni a renforcé modérément la capacité institutionnelle en Afrique du Sud, mais a renforcé de façon importante les capacités de recherche individuelle dans ce pays. Les activités de renforcement des capacités mises en oeuvre lors de cette collaboration ont compris : formation postuniversitaire, élaboration en commun de propositions, publication et diffusion de résultats de recherche, détachement de personnel, mentorat et échanges [^[[63]](#endnote-63)^].

Dans la Communauté de développement d’Afrique australe (SADC), une collaboration Sud-Sud entre le Centre d’excellence pour la recherche et la formation biomédicales de la région et le Laboratoire de recherche Blair du Zimbabwe avec l’appui du laboratoire danois Bilharziasis a aidé à développer les capacités de recherche au Zimbabwe et dans d’autres établissements africains grâce à de la formation au niveau du doctorat et à des projets de recherche conjoints [^[[64]](#endnote-64)^]. En dernier lieu, la capacité de recherche entourant les déterminants sociaux de la santé (DSS) était renforcée dans plusieurs PFR-PRI (Brésil, Colombie, Mexique, Kenya, Afrique du Sud et Tanzanie) grâce à des réseaux collaboratifs triangulaires Sud-Nord-Sud avec l’Espagne, la Suisse, le Royaume-Uni et l’Allemagne [^[[65]](#endnote-65)^].

Plusieurs études ont également révélé un lien étroit entre la coopération internationale en recherche et la productivité scientifique accrue [^[[66]](#endnote-66),^^[[67]](#endnote-67),^^[[68]](#endnote-68)^]. Elles affirment que les complexités impliquées dans la recherche nécessitent des connaissances plus spécialisées qu’aucun individu ou pays à lui seul n’est censé avoir. Par conséquent, la collaboration permet aux personnes de jouer leurs meilleures cartes qui contribuent à leurs compétences les plus solides et à leurs connaissances les plus vastes, tout en comptant sur d’autres pour contribuer à d’autres compétences et connaissances [^[[69]](#endnote-69)^]. À grande échelle, la collaboration permettra aux établissements et aux pays de mobiliser et d’utiliser leurs capacités différenciées pour améliorer le processus de production de connaissances en vue d’accroître la productivité.

**Limites de l’étude**

La littérature grise dans des cadres faibles en ressources peut être indispensable pour déterminer les résultats d’activités de recherche et d’efforts de production de connaissances. L’examen de la portée était malheureusement limité aux publications à comité de lecture, ce qui signifie qu’une quantité assez importante de données probantes ont été exclues pour présenter une vue d’ensemble de la situation. De plus, les publications en portugais, la troisième langue officielle en Afrique de l’Ouest, ne pouvaient être recueillies en raison du manque de connaissances linguistiques. La Guinée-Bissau est lusophone, mais nous avons trouvé une publication en langue anglaise provenant de ce pays. Aucune publication n’a été trouvée de Cabo Verde. Étant donné les limites linguistiques, nous ne pouvons conclure qu’il n’y avait aucune publication de RPSS provenant de Cabo Verde ou que la seule publication que nous avons trouvée en anglais de la Guinée-Bissau est la seule possible. Le domaine de la RPSS est très large et même si nous avons essayé d’utiliser un vaste éventail de termes de recherche, nous sommes peut-être passés à côté de certains articles. Par conséquent, la valeur de cette étude réside dans sa nature comparative au lieu de l’être dans les nombres absolus. Finalement, l’étude a couvert une période de 25 ans et les rattachements des auteurs à des établissements peuvent avoir changé au cours de cette période.

**Conclusions**

La première analyse situationnelle de la RPSS en Afrique de l’Ouest a examiné les tendances générales dans les publications et a cherché à déterminer les personnes et les établissements impliqués dans la RPSS. Les résultats ont montré une croissance très lente, mais stable, des publications de RPSS depuis 2008 avec une répartition inégale de productions parmi les pays et les établissements. Les chercheurs nigériens et ghanéens ont produit plus de 50 % du nombre total de publications de RPSS dans la région. Il semble que davantage de chercheurs et d’établissements qui ne sont pas ouest-africains dirigent les programmes de RPSS en Afrique de l’Ouest francophone, et la plupart des francophones ont publié en anglais, ce qui en a fait la langue privilégiée pour les publications scientifiques. La recherche et les publications de RPSS dans la CEDEAO ont augmenté régulièrement au fil du temps. Elles sont cependant assez dominées par des chercheurs principaux rattachés à des établissements du Nord, surtout dans les pays francophones et les plus petits pays anglophones. Bien que les chercheurs principaux aient généralement recours à des collaborateurs de la CEDEAO, il est important que la capacité de diriger de la recherche appliquée telle que la RPSS soit renforcée dans la CEDEAO.

De toute évidence, il est impératif d’atteindre la masse critique de producteurs et d’utilisateurs de RPSS dans la sous-région. Ces résultats donnent une idée juste quant à la capacité future de formation et de mentorat sur la RPSS qui pourrait éventuellement exister en Afrique occidentale. Comme pour de nombreux PFR-PRI, le manque de ressources (financières et humaines) a été ciblé comme étant l’un des facteurs qui limite la production de RPSS en Afrique occidentale. Par conséquent, pour une région pauvre en ressources, les collaborations institutionnelles entre les pays qui mettent l’accent sur la mise en place de programmes collaboratifs de recherche parmi les chercheurs et les utilisateurs dans la sous-région sont essentielles. La validation de cette collaboration avec d’autres partenaires du Nord pourrait aider davantage à relever les défis cernés. Heureusement, il existe quelques activités embryonnaires de recherche collaborative au sein de la sous-région qui pourraient être explorées et développées davantage en une collaboration Sud-Sud plus durable.

**Liste des abréviations utilisées**

ONG : Organisation non gouvernementale, SAC : systèmes adaptatifs complexes, OMD : Objectifs du Millénaire pour le développement, PFR-PRI : pays à faible revenu et pays à revenu intermédiaire, RPSS : recherche sur les politiques et les systèmes de santé, OMS : Organisation mondiale de la Santé, MEPIN : Medical Education Partnership Initiative au Nigéria, SADC : Communauté de développement d’Afrique australe, DSS : déterminants sociaux de la santé, UE : Union européenne, UK DFID : Département du Développement international du Royaume-Uni, OOAS : Organisation ouest-africaine de la santé, CEDEAO : Communauté économique des États de l’Afrique de l’Ouest.

**Contribution des auteures**

I.A.A, A.L. et S.D. ont conçu l’article, S.D. a compilé les données. S.D., A.K. et I.A.A. ont analysé les données. S.D. a dirigé la rédaction de l’article et I.A.A. et A.K. ont révisé d’un oeil critique les différentes itérations du manuscrit et approuvé la version définitive.

**Approbation de l’éthique et consentement à participer**

Sans objet

**Intérêts conflictuels**

Les auteures déclarent n’avoir aucun intérêt opposé.

**Financement**

Ce travail a reçu le soutien financier du Centre de recherches pour le développement international (CRDI) dans le cadre du projet de renforcement, dans les pays d’Afrique de l’Ouest, des capacités de recherche sur les systèmes de santé (107694 -001).

**Références**

1. Kebede, D., W. E. Soumbey-Alley, E. Asamoah-Odei, P. S Lusamba-Dikassa et L. G.Sambo (2010). « Progress on the health-related MDGs in the African Region ». *Observatoire africain de la Santé*, *10*, 10-17. [↑](#endnote-ref-1)
2. Organisation mondiale de la Santé. (2000). « Rapport sur la santé dans le monde, 2000 – Pour un système de santé plus performant ». Organisation mondiale de la Santé. [↑](#endnote-ref-2)
3. Organisation mondiale de la Santé. (2003). « Rapport sur la santé dans le monde, 2003 – façonner l’avenir ». Organisation mondiale de la Santé. [↑](#endnote-ref-3)
4. Swanson, R. C., A. Cattaneo, E. Bradley, S. Chunharas, R. Atun, K. M. Abbas et A. Best (2012). « Rethinking health systems strengthening: key systems thinking tools and strategies for transformational change ». *Health Policy and Planning*, *27*(suppl 4), iv54-iv61. [↑](#endnote-ref-4)
5. Campbell, D. M., S. Redman, L. Jorm, M. Cooke, A. B. Zwi et L. Rychetnik (2009). « Increasing the use of evidence in health policy: practice and views of policy makers and researchers ». *Australia and New Zealand Health Policy*,6(1), 21. [↑](#endnote-ref-5)
6. Dobrow, M. J., V. Goel et R. E. G. Upshur (2004). *«*Evidence-based health policy: context and utilisation ». *Social science & medicine*, 58(1), 207-217. [↑](#endnote-ref-6)
7. Hanney, S. R., M. A. Gonzalez-Block, M. J. Buxton et M. Kogan (2003). *«*The utilisation of health research in policy-making: concepts, examples and methods of assessment ». *Health research policy and systems*, 1(1), 2. [↑](#endnote-ref-7)
8. Innvær, S., G. Vist, M. Trommald et A. Oxman (2002). « Health policy-makers’ perceptions of their use of evidence: a systematic review ». *Journal of health services research & policy*, *7*(4), 239-244. [↑](#endnote-ref-8)
9. Bennett, S., A. Ghaffar, A. Mills, M. Yesudian et J. Mandelbaum-Schmidt (2007). « What is health policy and systems research and why does it matter? ». *Briefing note*, (1), 1-8. [↑](#endnote-ref-9)
10. Alliance pour la recherche sur les politiques et les systèmes de santé (2011). « What is Health Policy and Systems Research (HPSR)? Overview ». Genève, Organisation mondiale de la Santé, <http://www.who.int/alliance-hpsr/about/hpsr/en/>, consulté le 9 mai 2016. [↑](#endnote-ref-10)
11. Remme, J. H., T. Adam, F. Becerra-Posada, C. D’Arcangues, M. Devlin, C. Gardner et M. T. Mbizvo (2010). « Defining research to improve health systems ». *PLoS Med*, *7*(11), e1001000. [↑](#endnote-ref-11)
12. Sommet ministériel sur la recherche en santé : la Déclaration de Mexico sur la recherche en santé. Forum mondial pour la recherche en santé (2004). Genève : Forum mondial

    pour la recherche en santé. [↑](#endnote-ref-12)
13. « World Report on Knowledge for Better Health: Strengthening Health Systems ». Organisation mondiale de la Santé (2004). Genève : Organisation mondiale de la Santé. [↑](#endnote-ref-13)
14. « Informed choices for attaining the Millennium Development Goals: Towards an international cooperative agenda for health-systems research ». Task Force on Health Systems Research (2004). Lancet 364: 1756. [↑](#endnote-ref-14)
15. Résolution WHA58.34 de l’Assemblée mondiale de la Santé, Sommet ministériel sur la recherche en santé. Organisation mondiale de la Santé (2005). Genève : Organisation mondiale de la Santé. [↑](#endnote-ref-15)
16. « Report from the Ministerial Summit on Health Research: Identify challenges, inform actions, correct inequities ». Organisation mondiale de la Santé (2005). Genève : Organisation mondiale de la Santé. [↑](#endnote-ref-16)
17. « Bamako Call to Action ». Forum ministériel mondial sur la recherche pour la santé (2008). Genève : Forum mondial pour la recherche en santé. [↑](#endnote-ref-17)
18. Executive Board, 124th session: « WHO’s role and responsibilities in health research: Bamako Global Ministerial Forum on Research for Health ». Organisation mondiale de la Santé (2009). Genève : Organisation mondiale de la Santé. [↑](#endnote-ref-18)
19. « Scaling up research and learning for health systems: Now is the time: Report of a High Level Task Force », présenté et appuyé au Forum ministériel mondial sur la recherche pour la santé en 2008, Bamako, Mali. Organisation mondiale de la Santé (2009). Genève : Organisation mondiale de la Santé. [↑](#endnote-ref-19)
20. Résolution WHA63.21 de l’Assemblée mondiale de la santé, « WHO’s Role and Responsibilities in Health Research ». Organisation mondiale de la Santé (2010). Genève : Organisation mondiale de la Santé. [↑](#endnote-ref-20)
21. Résolution WHA63/22 de l’Assemblée mondiale de la santé, « WHO’s role and responsibilities in health research: draft WHO strategy on research for health ». Organisation mondiale de la Santé (2010). Genève : Organisation mondiale de la Santé. [↑](#endnote-ref-21)
22. « Le rôle de l’UE dans la santé mondiale : Communication de la Commission au Conseil, au Parlement européen, au Comité économique et social européen et au Comité des régions ». Commission européenne (2010). Commission européenne. [↑](#endnote-ref-22)
23. « DFID Research Strategy 2008–2013: Working Paper Series: Better Health ». Département du Développement international du Royaume-Uni (2008). Londres, Royaume-Uni : DFID. [↑](#endnote-ref-23)
24. « Global Action for Health System Strengthening ». Task Force on Global Action for Health System Strengthening (2009). Policy Recommendations to the G8. Centre japonais d’échanges internationaux. [↑](#endnote-ref-24)
25. Bennett, S., T. Adam, C. Zarowsky, V. Tangcharoensathien, K. Ranson, T. Evans et Comité consultatif scientifique et technique régional de l’Alliance (2008). « From Mexico to Mali: progress in health policy and systems research ». *The Lancet*, *372*(9649), 1571-1578. [↑](#endnote-ref-25)
26. Yao, Q., K. Chen, L. Yao, P. H. Lyu, T. A. Yang, F. Luo et Z. Y. Liu (2014). « Scientometric trends and knowledge maps of global health systems research ». *Health Res Policy Syst*, *12*(1), 26. [↑](#endnote-ref-26)
27. Op. cit. : xxv [↑](#endnote-ref-27)
28. El-Jardali, F., D. Jamal, N. Ataya, M. Jaafar, S. Raouf, C. Matta et C. Smith (2011). « Health policy and systems research in twelve Eastern Mediterranean countries: a stocktaking of production and gaps (2000–2008) ». *Health Res Policy Syst*, *9*(39), 10-1186. [↑](#endnote-ref-28)
29. Op. cit. : xxvi [↑](#endnote-ref-29)
30. Op. cit. :xix [↑](#endnote-ref-30)
31. Sundewall, J., R. C. Swanson, A. Betigeri, D. Sanders, T. E. Collins, G. Shakarishvili et R. Brugha (2011). « Health-systems strengthening: current and future activities ». *The Lancet*, *377*(9773), 1222-1223. [↑](#endnote-ref-31)
32. Banque mondiale (n.d.) « Country and lending groups », <http://data.worldbank.org/about/country-and-lending-groups>, consulté le 31 mai 2016. [↑](#endnote-ref-32)
33. Op. cit. : xxviii. [↑](#endnote-ref-33)
34. Sawyerr, A. (2004). « African universities and the challenge of research capacity development ». *Journal of Higher Education in Africa/Revue de l’enseignement supérieur en Afrique*, 213-242 [↑](#endnote-ref-34)
35. Sombié, I., J. Aidam, B. Konaté, T. D. Somé et S. S. Kambou (2013). « The state of the research for health environment in the ministries of health of the Economic Community of the West African States (ECOWAS) ». *Health Res Policy Syst*, *11*, 35. [↑](#endnote-ref-35)
36. Nwaka, S., T. B. Ilunga, J. S. Da Silva, E. R. Verde, D. Hackley, R. De Vré et R. G. Ridley (2010). « Developing ANDI: a novel approach to health product R&D in Africa ». *PLoS Med*, *7*(6), e1000293. [↑](#endnote-ref-36)
37. Op. cit. : xxxv [↑](#endnote-ref-37)
38. Arksey, H. et L. O’Malley (2005). « Scoping studies: towards a methodological framework ». *International journal of social research methodology*, *8*(1), 19-32. [↑](#endnote-ref-38)
39. Levac, D., H. Colquhoun, H. et K. K. O’Brien (2010). « Scoping studies: advancing the methodology ». *Implement Sci*, *5*(1), 1-9. [↑](#endnote-ref-39)
40. Davis, K., N. Drey et D. Gould (2009). « What are scoping studies? A review of the nursing literature ». *International journal of nursing studies*, *46*(10), 1386-1400. [↑](#endnote-ref-40)
41. [↑](#endnote-ref-41)
42. Op. cit. : xv

    Op. cit. : xvii

    [↑](#endnote-ref-42)
43. [↑](#endnote-ref-43)
44. Op. cit. : xvi [↑](#endnote-ref-44)
45. Op. cit. : xxi [↑](#endnote-ref-45)
46. Op. cit. : xxiii [↑](#endnote-ref-46)
47. Op. cit. : xxii [↑](#endnote-ref-47)
48. Mills, A. (2004). « Strengthening health systems: the role and promise of policy and systems research ». Organisation mondiale de la Santé (OMS). Alliance pour la recherche sur les politiques et systèmes de santé. [↑](#endnote-ref-48)
49. Kirigia, J. M. et C. Wambebe (2006). « Status of national health research systems in ten countries of the WHO African Region ». *BMC Health Services Research*, *6*(1), 1. [↑](#endnote-ref-49)
50. Op. cit. : xxv [↑](#endnote-ref-50)
51. Op. cit. : xxxv [↑](#endnote-ref-51)
52. Adam, T., S. Ahmad, M. Bigdeli, A. Ghaffar et J. A. Røttingen (2011). « Trends in health policy and systems research over the past decade: still too little capacity in low-income countries ». *PLoS One*, *6*(11), e27263. [↑](#endnote-ref-52)
53. Op. cit. : xx [↑](#endnote-ref-53)
54. Mbondji, P. E., D. Kebede, C. Zielinski, W. Kouvividila, I. Sanou et P. S. Lusamba-Dikassa (2014). « Overview of national health research systems in sub-Saharan Africa: results of a questionnaire-based survey ». *Journal of the Royal Society of Medicine*, *107*(1 suppl), 46-54. [↑](#endnote-ref-54)
55. Op. cit. : xxv [↑](#endnote-ref-55)
56. Idem [↑](#endnote-ref-56)
57. PNUD. « Capacity development practice note », Programme des Nations Unies pour le développement, New York. [http://www.undp.org/content/dam/undp/library/capacity-development/french/PN%20Capacity%20Development_FR_final.pdf,](http://www.undp.org/content/dam/undp/library/capacity-development/french/PN%20Capacity%20Development_FR_final.pdf) consulté le 11 juin 2016. [↑](#endnote-ref-57)
58. Block, M. A. G. et A. Mills (2003). « Assessing capacity for health policy and systems research in low and middle income countries* ». *Health Research Policy and Systems*, *1*(1), 1. [↑](#endnote-ref-58)
59. Op. cit. : liii [↑](#endnote-ref-59)
60. Op. cit. : lviii [↑](#endnote-ref-60)
61. Op. cit. : xxxv [↑](#endnote-ref-61)
62. Olaleye, D. O., G. N. Odaibo, P. Carney, O. Agbaji, A. S. Sagay, H. Muktar et S. Akanmu (2014). « Enhancement of health research capacity in Nigeria through North–South and in-country partnerships ». *Academic Medicine*, *89*(8), S93-S97. [↑](#endnote-ref-62)
63. Mayhew, S. H., J. Doherty et S. Pitayarangsarit (2008). « Developing health systems research capacities through north-south partnership: an evaluation of collaboration with South Africa and Thailand ». *Health Research Policy and Systems*, *6*(1), 8. [↑](#endnote-ref-63)
64. Chandiwana, S. et N. Ornbjerg (2003). « Review of North-South and South-South cooperation and conditions necessary to sustain research capability in developing countries ». *Journal of Health, Population and Nutrition*, 288-297. [↑](#endnote-ref-64)
65. Cash-Gibson, L., G. Guerra et V. N. Salgado-de-Snyder (2015). « SDH-NET: a South–North-South collaboration to build sustainable research capacities on social determinants of health in low-and middle-income countries ». *Health Research Policy and Systems*, *13*(1), 45. [↑](#endnote-ref-65)
66. de Solla Price, D. J. et D. Beaver (1966). « Collaboration in an invisible college ». *American psychologist*, *21*(11), 1011. [↑](#endnote-ref-66)
67. Godin, B. et Y. Gingras (2000). « Impact of collaborative research on academic science ». *Science and Public Policy*, *27*(1), 65-73. [↑](#endnote-ref-67)
68. Zuckerman, H. (1967). « Nobel laureates in science: Patterns of productivity, collaboration, and authorship ». *American Sociological Review*, 391-403. [↑](#endnote-ref-68)
69. Lee, S. et B. Bozeman (2005). « The impact of research collaboration on scientific productivity ». *Social studies of science*, *35*(5), 673-702. [↑](#endnote-ref-69)
