# Supplementary material for: People and research: improved health systems for West Africans, by West Africans - report on special supplement
Source: BMC Proc. 2019 Feb 7;13(Suppl 1):1. doi: 10.1186/s12919-019-0162-0 (PMC6366023; doi:10.1186/s12919-019-0162-0)
Supplement: Supplementary file 10 — L’expérience ouest-africaine de constitution de comités de pilotage pour améliorer la collaboration entre chercheurs et décideurs et accroître l’utilisation des résultats de la recherche en santé, Keita, N., Lokossou, V., Berthe, A., Sombie, I., Johnson, E., Busia, K. [file 12919_2019_162_MOESM10_ESM.docx]

| ***L’expérience ouest-africaine de constitution de comités de pilotage pour améliorer la collaboration entre chercheurs et décideurs et accroître l’utilisation des résultats de la recherche en santé*** |
| --- |

**Namoudou Keita^1^, Virgil Lokossou^1^, Abdramane Berthe^1^, Issiaka Sombie^1^, Ermel Johnson^1^ et Kofi Busia^1^**

* Correspondance : [vlokossou@wahooas.org](mailto:vlokossou@wahooas.org)

**^1^**Organisation Ouest Africaine de la Santé (OOAS), 01 BP 153 Bobo-Dioulasso 01, Burkina Faso

La liste complète des auteurs est disponible à la fin de l’article.

# Résumé

**Introduction :** Consciente des avantages que présentent les comités de pilotage (CP) de projets pour influencer la conception de politiques de santé à partir de données probantes, l’Organisation Ouest Africaine de la Santé (OOAS) a encouragé et soutenu la création de tels comités dans le cadre de quatre projets de recherche menés dans quatre pays (Burkina Faso, Nigéria, Sénégal et Sierra Leone). La présente étude a été réalisée pour décrire le processus de constitution de ces comités. Les résultats présentés ici visent à aider d’autres acteurs à entreprendre un tel processus.

**Méthodes :** Il s’agit d’une étude qualitative transversale sur les quatre projets de l’initiative. Outre une revue de la littérature et des documents de projet, les données ont été collectées auprès de 14 membres de CP, des équipes de recherche, de l’OOAS et du Centre de recherches pour le développement international (CRDI) à l’aide d’un guide d’entretien. La sélection des enquêtés visait la saturation des données. La technique d’analyse thématique par simple catégorisation a été utilisée.

**Résultats :** Pour constituer les CP, une équipe de recherche a travaillé avec les autorités sanitaires de chaque pays pour cerner les membres potentiels, organisé des réunions avec ces personnes et obtenir l’aval des autorités en vue d’officialiser les CP. Les CP avaient pour mission d’apporter une assistance technique aux chercheurs durant l’étape de la mise en oeuvre, ainsi que de faciliter le transfert et l’utilisation des résultats. L’approche « agir en apprenant » utilisée par chaque équipe de recherche et le rôle de catalyseur joué par l’OOAS auprès du ministère de la Santé de chaque pays ont permis aux CP de gérer leurs difficultés contextuelles et de fonctionner efficacement.

**Conclusion :** La participation de partenaires techniques et financiers a motivé les chercheurs et les ministères de la Santé qui, à leur tour, ont incité d’autres acteurs à siéger bénévolement aux CP. L’adoption de l’approche « agir en apprenant » a permis l’élaboration de stratégies adaptées aux divers contextes afin de créer, de faciliter et de faire fonctionner chaque CP, ainsi que d’en gérer les difficultés. La reproduction d’une telle expérience nécessitera une grande maîtrise du contexte local et la participation de partenaires solides.

**Mots clés :** Pilotage; Transfert de l’information; Comité d’évaluation et de diffusion d’innovations technologiques; Collaboration interprofessionnelle; Appropriation des connaissances; Afrique de l’Ouest.

# Contexte

La littérature scientifique a mis en évidence le rôle joué par les comités de pilotage (CP) [1-5], les comités de suivi et les comités consultatifs dans la recherche en santé [6-13] et dans les soins de santé [14, 15]. Un CP est un groupe d’acteurs (des leaders parmi les parties prenantes d’un projet) qui se réunit régulièrement pour déterminer, évaluer et orienter la mise en oeuvre d’un projet’, et suggérer des stratégies pour favoriser l’atteinte des objectifs dudit projet. Dans un programme de recherche, les CP peuvent jouer un rôle important dans la conception de la recherche, le transfert des résultats de recherche aux utilisateurs potentiels (communauté, praticiens et décideurs) et l’appropriation/utilisation des résultats par les utilisateurs. Lemire et al. [16] ont décrit les approches, les étapes et les déterminants du processus de transfert des connaissances. Le transfert et l’utilisation des connaissances scientifiques ne peuvent se faire sans interaction directe ou indirecte entre les chercheurs, les acteurs/bénéficiaires et les décideurs. Des collaborations étroites et continues facilitent ou garantissent le succès du transfert des connaissances ou la production et l’utilisation des constatations. Pour y parvenir, le regroupement des parties prenantes au sein d’un CP fonctionnel favorise à son tour l’instauration d’une collaboration dynamique. Les CP sont devenus des indicateurs de la forte participation à la recherche d’acteurs n’étant pas des chercheurs, ce qui représente une bonne stratégie pour établir des partenariats multi-acteurs, multisectoriels et multidimensionnels. De tels partenariats permettent de prendre en compte à la fois les besoins, les aspirations et les ressources des différentes parties [13]. Grâce à la littérature scientifique [5], ces différentes parties et leurs partenaires techniques et financiers ont découvert les avantages de ces comités. Cependant, ils méconnaissent ou maîtrisent mal le mécanisme qui mène à leur création, puisque cette même littérature scientifique contient peu d’information sur la constitution de ces comités. Selon Uneke et al. [5], un comité consultatif sur les politiques en santé peut servir d’excellente plate-forme pour faciliter l’interaction entre les décideurs et les chercheurs. Les auteurs ont montré que la mise en place d’un comité consultatif sur les politiques en santé peut stimuler les efforts du ministère de la Santé en vue d’appliquer des stratégies fondées sur des données probantes à l’amélioration des services. Cependant, ces auteurs, comme bien d’autres s’étant prononcés sur les CP, ont omis de décrire le mécanisme de constitution de ces types de comités. Le présent article aide à combler le manque de documentation scientifique du processus de constitution d’un CP dans le cadre d’un projet de recherche en santé.

En Afrique de l’Ouest, consciente des avantages de ces comités, l’Organisation Ouest Africaine de la Santé (OOAS) a encouragé et soutenu leur création dans le cadre de « l’Initiative ouest-africaine de renforcement des capacités au moyen de la recherche sur les systèmes de santé ». Cette initiative visait à renforcer les capacités des chercheurs, des acteurs et des décideurs dans la conduite de recherches sur les systèmes de santé, et dans le transfert et l’utilisation des résultats de ces recherches. Pour concevoir ce projet, l’OOAS est parti du constat qu’en Afrique de l’Ouest, plusieurs indicateurs de santé sont faibles. Les nombreux résultats de recherche en santé qui existent sont peu utilisés par les acteurs et les décideurs [17-19] en raison d’un accès limité; de difficultés de compréhension; du rejet des résultats ou de doutes quant à leur légitimité; de résultats contraires aux manières de penser, d’agir ou d’être des acteurs non chercheurs et des chercheurs; de la non-participation d’acteurs non chercheurs au processus de production des résultats, etc. [5, 17-20]. Les différents acteurs de la recherche sur les systèmes de santé (chercheurs, praticiens, décideurs) se méconnaissent et collaborent peu, par profil ou entre profils, par pays ou entre pays. Les bonnes pratiques de recherche et d’intervention en santé sont peu visibles et peu partagées. Il existe aussi des lacunes sur le plan des politiques et des pratiques fondées sur des données scientifiques [5, 17-21], en particulier en ce qui concerne la rétroaction. Cette initiative se veut donc la contribution de l’OOAS à la stimulation, à la promotion et au renforcement de la collaboration entre chercheurs, acteurs et décideurs par le biais de la constitution de comités de pilotage (CP) de projets de recherche en santé, en vue d’accroître l’utilisation des résultats et d’améliorer l’équité et la gouvernance dans les systèmes de santé. L’OOAS a été soutenue financièrement par le Centre de recherches pour le développement international (CRDI).

À ce stade de l’initiative, les équipes de recherche analysent les résultats de leurs recherches. Les CP utiliseront ces résultats pour influencer la prise de décisions sur les politiques et les programmes de santé. Il est donc difficile d’établir l’efficacité de ces CP. Un article spécifique analysera l’efficacité des CP un an après la fin de l’initiative. En revanche, une étude sur le processus de constitution des CP s’avère possible à l’étape de la mise en oeuvre de l’initiative. Cet article vise donc à décrire et à analyser le processus de constitution de CP dans le cadre de quatre projets de recherche de l’Initiative ouest-africaine de renforcement des capacités au moyen de la recherche sur les systèmes de santé, afin d’aider d’autres acteurs souhaitant utiliser un tel outil de collaboration.

# Méthodes

Il s’est agi d’une étude qualitative, descriptive et analytique, réalisée entre février et avril 2016 et portant sur les CP ou comités de suivi de quatre projets de « l’Initiative ouest-africaine de renforcement des capacités au moyen de la recherche sur les systèmes de santé ». Cette initiative a été mise en oeuvre par des consortiums d’organisations de recherche ou d’intervention au Burkina Faso, au Nigéria, au Sénégal et en Sierra Leone. Les consortiums étaient composés de l’équipe de recherche et des organisations de provenance des membres des CP. Les sujets suivants sont abordés par ces consortiums : conception d’un processus d’évaluation de la performance du système de santé de district (Burkina Faso); renforcement du système de santé grâce à un meilleur accès équitable aux soins de santé primaires (Nigéria); financement, équité et gouvernance dans le système de santé (Sénégal); et obstacles empêchant les femmes enceintes d’accéder gratuitement aux établissements de santé (Sierra Leone).

Pour mener cette étude, nous avons d’abord réalisé une revue de la littérature en explorant différentes bases de données (principalement PubMed et Cairn) à l’aide de différents mots clés (comité consultatif; comité de personnel professionnel; composition des comités; pilotage; transfert d’information; comité d’évaluation et de diffusion d’innovations technologiques; collaboration interprofessionnelle; appropriation des connaissances). Nous avons aussi exploré différents documents relatifs aux CP de ces quatre projets. Cette revue nous a permis de peaufiner notre guide d’entretien.

La population ciblée pour la collecte des données qualitatives était composée de membres des CP (appelé « comité de suivi » au Burkina Faso), de chercheurs des équipes de recherche, ainsi que d’employés de l’OOAS et du CRDI impliqués dans cette initiative. Les enquêtés ont été sélectionnés de façon raisonnée et opportuniste au sein de cette population. Nous avons profité de l’atelier de revue par les pairs (rencontre des différents consortiums de recherche et d’intervention des quatre pays) tenu à Dakar, au Sénégal, en février 2016 pour collecter des données auprès des participants. Au moins deux personnes (un chercheur et un membre du CP) ont été interrogées par pays. Il s’agissait le plus souvent du chercheur principal et/ou de son représentant et/ou d’un chercheur, et du président du CP ou de son représentant et/ou d’un membre du CP. Les entretiens ont été réalisés avec tous les enquêtés d’un pays jusqu’à la saturation des données. La saturation était atteinte avec un enquêté lorsque nous estimions avoir obtenu l’ensemble des informations nécessaires pour atteindre l’objectif de l’étude et que la poursuite de l’entretien n’aurait pas permis de recueillir de nouvelles informations stratégiques. Toutefois, au cours de l’analyse, des échanges de courriels ont tout de même eu lieu pour obtenir d’autres informations stratégiques auprès des enquêtés.

Les questions ont été soumises à chaque enquêté par le même chercheur/enquêteur. Ce chercheur/enquêteur était un consultant indépendant n’étant pas employé par l’OOAS ou le CRDI, ni membre d’une équipe de recherche d’un des quatre pays. Cette personne était toutefois au fait de cette initiative ouest-africaine. Le guide d’entretien était conçu autour des points suivants : présentation de l’enquêté, processus de création du CP, mode de constitution du CP, composition du CP, fonctionnement du CP, ressources et mission du CP, forces et faiblesses du processus, difficultés rencontrées, etc. Les entretiens ont été enregistrés et transcrits en français. Malgré l’anonymat garanti, les enquêtés étaient conscients que le rapport et l’article qui découleraient de l’étude seraient accessibles à l’ensemble des acteurs de l’initiative. Il est possible que cette situation ait poussé certains enquêtés à tenir des discours « politiquement corrects » pour ne léser aucun partenaire ou partie prenante.

Cette étude est caractérisée par une approche exhaustive [22], qui vise à comprendre les enquêtés et le phénomène étudié. Cette approche a accordé une grande importance aux déclarations, motivations (ce qui détermine un acte, une décision) et rationalités des enquêtés. Elle a tenu compte du contexte dans lequel se réalisent les quatre projets. L’étude a aussi utilisé l’approche systématique [22], laquelle privilégie le principe de la causalité circulaire et de la vérité plurielle. Depuis sa conception jusqu’à sa valorisation, cette étude a respecté les principes de l’éthique de la recherche scientifique en santé. Le principe du relativisme de l’éthique [22] de la recherche qualitative a été appliqué tout au long de cette étude. Enfin, cette étude a utilisé la théorisation ancrée dans les données [23]. Aucune hypothèse de départ n’a été formulée. Toutefois, au cours de la revue de la littérature, les chercheurs se sont particulièrement intéressés aux résultats ou modèles explicatifs d’autres auteurs [1-4, 7, 9, 12, 13].

Les données de cette étude ont été dépouillées manuellement au fur à mesure qu’elles étaient collectées. La technique d’analyse thématique par simple catégorisation a été utilisée [22]. Les catégories retenues étaient entre autres : création du CP, occasions surgies pendant la création, difficultés rencontrées, composition du CP, fonctionnement du CP, ressources et mission du CP. Chaque résultat majeur a été interprété (pour lui donner un sens dans son contexte), consolidé ou infirmé par les écrits d’autres auteurs.

**Résultats**

Au total, 14 personnes dont 4 membres de CP, 7 chercheurs et 3 membres d’une organisation sous-régionale (OOAS) ou internationale (CRDI) ont participé à cette étude. Tous les participants avaient participé à l’initiative depuis son lancement.

## Origine de l’idée de constituer des CP

Selon certains enquêtés, l’idée de constituer des CP a été implicitement évoquée dans l’appel à notes conceptuelles lancé par le CRDI et l’OOAS en août 2012. Cet appel invitait des équipes multipartites de chercheurs, de décideurs et de praticiens à présenter des notes conceptuelles sur deux thèmes admissibles, à savoir l’accès équitable aux systèmes de santé, et la gouvernance et les structures de gouvernance des systèmes de santé. Par ailleurs, l’objectif particulier 2 de cet appel précisait que l’initiative visait à resserrer les liens entre les groupes multipartites de chercheurs, de praticiens et de décideurs chargés de s’attaquer à des problèmes particuliers qu’ils avaient préalablement cernés. Parmi les critères d’admissibilité à cet appel, la demande devait être présentée au nom d’un consortium multipartite composé de chercheurs, de décideurs et de praticiens aptes à définir un problème ayant trait au renforcement des systèmes de santé et à utiliser les résultats de la recherche pour commencer à s’attaquer au problème.

Enfin, l’appel précisait que les équipes candidates devaient être composées à la fois de chercheurs et de partenaires, et que la qualité de cette composition représentait 20 % des critères de sélection des candidatures. Ces éléments ont mené à l’idée de constituer un CP dans la plupart des consortiums ayant répondu à cet appel : « Depuis le lancement du projet, nous [ministère de la Santé et de l’Action sociale du Sénégal] avons été sollicités pour apporter l’appui politique du ministère, afin de montrer que ce projet, de par sa conception, pouvait être intéressant pour le ministère de la Santé.

Nous avons donc produit une lettre d’appui à la proposition. » (membre 1 du CP du Sénégal).

Ainsi, les premières organisations partenaires pouvant ou devant participer aux CP (partenaires potentiels) ont été identifiées par les chercheurs pour constituer leur consortium et répondre à l’appel à notes conceptuelles. Une soixantaine de consortiums ouest-africains ont proposé des notes conceptuelles en réponse à cet appel. À l’issue d’un processus d’examen, sept propositions ont été présélectionnées selon des critères techniques rigoureux, à savoir : pertinence et retombées potentielles du projet de recherche (40 %); pertinence et mérite scientifiques potentiel (40 %) et composition des équipes et partenaires (20 %).

Afin de permettre aux consortiums de mieux préparer leurs soumissions finales aux fins d’examen, un atelier de création de protocoles a été donné à Bobo-Dioulasso, au siège de l’OOAS, en octobre 2012. Au cours de cet atelier, les consortiums présélectionnés ont interagi avec des spécialistes du comité consultatif régional constitué par l’OOAS et le CRDI. Cette fois-ci, l’idée de constituer des CP a été explicitement suggérée aux consortiums : « Depuis le début du processus, à l’occasion d’une réunion régionale de dialogue organisée par le CRDI en novembre 2011 à Dakar, lorsque l’OOAS a été retenue par l’ensemble des participants à ce dialogue en tant qu’institution supranationale devant soumettre une proposition, la proposition principale de l’OOAS a été de mettre en place des CP fondés sur les équipes.

Cette idée a été bien accueillie par le CRDI. Lors de la rencontre avec les sept équipes présélectionnées à Bobo-Dioulasso, l’OOAS a présenté l’idée de CP. Il a été demandé aux équipes, au-delà des questions de recherche, de la conception de la recherche et de l’approche méthodologique, entre autres, de prendre en compte la constitution du CP et donc d’être prêtes à mettre en place le CP dès leur retour et de l’associer à la finalisation des questions de recherche examinées à Bobo-Dioulasso. »
(entretien avec un responsable de l’OOAS)

Cette information a été confirmée par tous les enquêtés qui, à l’unanimité, ont déclaré que l’idée de constituer des CP réunissant des chercheurs, des praticiens de la santé et des décideurs venait implicitement et explicitement de l’OOAS. En stimulant et en soutenant la création de ces CP, l’OOAS a présumé que la réunion de chercheurs, d’acteurs/intervenants et de décideurs dans un cadre fonctionnel de rencontre régulière (CP) aurait plusieurs conséquences. Elle leur permettrait de co-définir un thème de recherche à haute utilité pratique basé sur les besoins et aspirations des acteurs et des décideurs et tenant compte des ressources, compétences et capacités des chercheurs. Elle favoriserait la co-production de résultats probants et la communication de ces résultats. Elle autoriserait l’appropriation de ces résultats par toutes les parties qui pourraient les utiliser ou les convertir en action au profit d’une communauté ou de la population générale.

Tous les chercheurs interrogés ont reconnu que l’idée de constituer ces CP avait été explicitement exprimée lors de cette rencontre. Selon un chercheur, l’idée de constituer ces CP est apparue comme étant « *naturelle* » dans le cadre de cette initiative, car tout (l’appel à notes conceptuelles, les recommandations des spécialistes du comité consultatif régional, les expériences des chercheurs) laissait croire que ces CP étaient indispensables : « l’idée de mettre en place un CP découle de plusieurs préoccupations.

D’abord, dans l’appel à propositions, il était clairement indiqué que les candidats devaient montrer clairement comment ils comptaient assurer une certaine appropriation et utilisation de leurs résultats de recherche… Mais au-delà de tout cela, c’était aussi une préoccupation récurrente. En général, au niveau de notre ministère [Burkina Faso], c’est assez systématique quand on veut mener ce genre d’activités. Les acteurs du ministère participent pleinement dès le départ. En général, nous mettons en place un CP ou un comité de suivi. » (membre de l’équipe de recherche du Burkina Faso)

Après la rencontre avec les spécialistes, quatre consortiums ont été sélectionnés. Ces consortiums ont participé à un atelier de développement de protocoles de recherche à Dakar, en mai 2013, et ont de nouveau rencontré le ministère de la Santé de leur pays respectif, une organisation qui devait participer au consortium pour que celui-ci ait une chance d’être sélectionné.

## Constitution des CP

Dans chaque pays, la constitution du CP a véritablement commencé après la sélection définitive du consortium. Lorsque les chercheurs, à savoir les leaders de ces consortiums, ont eu la certitude d’avoir été sélectionnés, ils ont commencé par informer d’autres partenaires potentiels de leur consortium. Chaque chercheur principal a établi une liste de membres potentiels du CP et rencontré le ministère de la Santé de son pays pour discuter de la constitution du CP. En général, outre les échanges par courriel ou par téléphone, deux ou trois réunions informelles ont été nécessaires pour obtenir la liste quasi définitive des membres du CP et des organisations partenaires. En fonction de la zone de collecte des données et de la thématique abordée, les chercheurs ont cerné les organisations les plus concernées par leur recherche et ses résultats. Cette liste a été modifiée par le ministère de la Santé pour obtenir la liste quasi définitive. Dans chaque organisation retenue, le premier responsable a désigné son représentant. Le choix était laissé à la discrétion de ce responsable. Dans la plupart des quatre pays, un document du ministère de la Santé ou de l’autorité sanitaire locale a officialisé la création du CP. Chaque CP était rattaché au ministère de la Santé par l’entremise de l’organisation chargée de la recherche dudit ministère; cette organisation assurait la présidence du CP.

Dès la constitution des CP, une première réunion officielle a été organisée pour permettre aux membres de se connaître davantage et pour modifier le protocole de recherche. Au cours de cette réunion, le CP a été lancé officiellement au moyen d’une déclaration faite devant les autorités sanitaires.

## Facteurs ayant facilité la constitution des CP

Selon les enquêtés, certains faits, gestes ou situations ont facilité la création des CP. À l’unanimité, les enquêtés ont cité l’engagement, la détermination ou le soutien de l’OOAS qui, en tant qu’organisation supranationale, a directement demandé au ministère de la Santé de chaque pays de bien vouloir s’impliquer à toutes les étapes (création du CP, conception, mise en oeuvre, valorisation, transfert et utilisation des résultats) du projet dans leur pays. Un responsable de l’OOAS a confirmé ces renseignements : « Pour encourager la mise en place des CP, le directeur général de l’OOAS a envoyé une lettre à chaque ministère de la Santé concerné.

Dans cette lettre, il était demandé au ministre de la Santé de bien vouloir mettre ses services compétents à la disposition de l’équipe de recherche. L’OOAS a présenté ces équipes et leur thème de recherche, et a demandé au ministère de s’engager à faciliter toute la recherche et l’utilisation des résultats. L’OOAS s’est aussi déplacée dans chaque pays pour appuyer les équipes dans la mise en place du CP. » (responsable de l’OOAS)

Dans chaque pays, les acteurs ont reconnu que l’engagement du ministère de la Santé à l’échelle nationale (Burkina Faso et Sénégal) ou locale (Nigéria et Sierra Leone) et les expériences du ministère dans la constitution de CP de projets similaires ont aussi facilité la création du CP : « Le ministère de la Santé et de l’Action sociale a l’habitude de mettre en place des CP.

En général, quand il veut mettre en place un CP, il essaye de prendre en compte l’ensemble des acteurs dès le début et de les associer autant que possible aux activités. Cela inclut généralement tout le monde : le ministère des Finances, l’Assemblée, le conseil économique, le secteur de l’environnement, les écoles, l’université, la société… Le ministère tente vraiment de ratisser large. Et le ministère est habitué à cela. » (membre 2 du CP du Sénégal)

Selon les enquêtés, outre ces engagements (supranational et national), dans chaque pays, le principal chercheur s’est investi sur plusieurs plans (appels téléphoniques ou appels Skype, envoi de courriels, réunions informelles).

Outre ces investissements multiformes, les expériences de collaboration entre les chercheurs principaux et le ministère de la Santé, leur carnet d’adresses ou leurs bonnes relations avec le ministère de la Santé ont beaucoup facilité la création du CP. Enfin, les perceptions positives des équipes, de la thématique de recherche et de la structure du CP ont fourni un incitatif non financier ainsi qu’un appui aux membres potentiels du CP, lesquels ont accepté de siéger bénévolement au CP.

## Taille et composition des CP

La taille des CP (nombre de membres par CP y compris au moins deux chercheurs) variait d’un pays à l’autre. Le CP comptait 10 membres au Burkina Faso, 18 en Sierra Leone, 20 au Sénégal et 23 au Nigéria. Selon les enquêtés, aucun quota hommes/femmes n’a été fixé par pays pour constituer les CP, mais dans l’ensemble des pays, au moins un cinquième des membres était des femmes. Les acteurs ont davantage opté pour la représentativité par institution ou service que par sexe. Chaque institution/service concerné était libre de choisir en son sein la personne appropriée pour siéger au CP. Dans chaque pays, le document officiel de constitution du CP précisait qu’en fonction des activités, les membres du CP auraient la possibilité de s’adjoindre d’autres membres ou organisations. De la constitution des CP jusqu’au moment de l’enquête, le nombre de membres par CP n’a pas changé. Toutefois, du fait de la mobilité professionnelle dans les postes ou institutions, les personnes siégeant aux CP ont souvent changé. Dans de tels cas, les personnes étaient systématiquement remplacées, sans que cela n’affecte le fonctionnement du CP. Ce fut notamment le cas au Sénégal lors du décès du point focal de l’OOAS, et en Sierra Leone lors de la mutation du médecin-chef de l’hôpital du district de Bombali.

Quant à la composition des CP, chaque pays a constitué des CP multisectoriels. Cette composition composite était conforme à l’une des recommandations principales de l’OOAS : « l’OOAS a insisté sur la nécessité de mettre en place un CP et de le rendre le plus hétérogène possible, et que les membres soient des acteurs concernés par l’étude et qu’ils puissent éventuellement commencer à utiliser les résultats intermédiaires avant la fin du projet de recherche. » (entretien avec un responsable de l’OOAS)

Dans les quatre pays, les membres provenaient des secteurs suivants : parlement (représentants locaux), communication et information (journalistes), santé (responsables au plan administratif ou opérationnel), transport, enseignement et éducation, défense et sécurité (police, gendarmerie), et société civile (association de protection et de promotion de la santé). Les CP de la Sierra Leone, du Nigéria et du Sénégal, composés de plus d’une dizaine de personnes, étaient plus hétérogènes que celui du Burkina Faso (10 membres), lequel était construit essentiellement, voire exclusivement de membres du secteur de la santé. Le fait que cette étude s’intéresse uniquement au processus de constitution des CP ne nous permet pas de connaître, à ce stade, l’incidence de la composition de chaque CP.

## Rôles/missions des CP

D’une façon globale, les CP s’étaient donné des rôles ou missions dans trois domaines. D’abord, dans le domaine de la facilitation de la recherche, les CP avaient pour mission de s’occuper de la validation et de la modification/l’ajustement des projets de recherche, de l’accompagnement des chercheurs dans la mise en oeuvre de leur recherche, et de la validation externe des résultats de recherche. Les CP avaient aussi pour mission de faciliter le transfert des résultats de la recherche aux utilisateurs potentiels. Enfin, les CP avaient pour rôle de faciliter l’appropriation et l’utilisation des résultats de la recherche.

## Fonctionnement des CP

Pour assurer le fonctionnement des CP, un arrêté ministériel (Burkina Faso et Sénégal) ou un mandat officiel (Nigéria, Sierra Leone) a officialisé leur création. L’OOAS a déclaré n’avoir pas trop insisté sur l’officialisation des CP, car toute « l’Initiative ouest-africaine de renforcement des capacités au moyen de la recherche sur les systèmes de santé » est basée sur la stratégie « agir en apprenant ». Il s’agissait pour les pays de constituer leur CP en suivant un processus moins spécifique et non préétabli, et de tirer des leçons de ce processus. Une trop grande officialisation réduirait donc l’apprentissage : « C’est vraiment une initiative qui est basée sur l’apprentissage par la pratique.

Chaque pays devait constituer son CP en fonction de son contexte. L’OOAS n’a pas voulu officialiser ni imposer un format standard. Par ailleurs, une officialisation trop administrative pourrait repousser les chercheurs. Il fallait plutôt encourager les chercheurs à travailler, à s’adresser à toutes les parties prenantes pour constituer les CP. » (entretien avec un responsable de l’OOAS)

Les CP fonctionnaient à l’aide des ressources financières détenues par les équipes de recherche financées par le CRDI. Les ressources matérielles provenaient des équipes de recherche ou des organisations des présidents des CP. Les rencontres des CP étaient dirigées par leur président. Dans tous les CP, les décisions ont été prises de façon consensuelle. Jamais les membres ne sont passés au vote pour prendre une décision. Aucun quorum n’a été fixé pour valider les réunions des CP. Dans la pratique, les membres ont le plus souvent été présents aux différentes rencontres. Les CP ont fonctionné sur la base du bénévolat : seuls le transport ou le carburant étaient remboursés aux membres pour leur permettre d’assister aux rencontres. Cela a été accepté par les membres qui, pour la plupart, étaient habitués à cette façon de faire avec d’autres partenaires. Les promoteurs de ces CP (l’OOAS, les chercheurs et le ministère de la Santé) ont justifié ce choix par leur souci commun d’assurer la pérennisation/systématisation des CP au moindre coût possible dans un contexte de rareté des ressources financières. Il n’y avait aucun lien formel ou direct entre les quatre CP. Cependant les rencontres annuelles des consortiums de l’initiative ont fourni des occasions de rencontres entre les équipes de recherche, les présidents des CP et les spécialistes/experts de l’OOAS et du CRDI. Ces rencontres ont permis le partage des expériences et la consolidation de la collaboration entre les acteurs d’un même pays et de plusieurs pays. L’OOAS a assuré le suivi et la coordination régionale de l’initiative.

Chaque CP a planifié deux rencontres par an. En trois ans, le nombre de rencontres effectives des CP a varié entre trois au Sénégal, quatre en Sierra Leone et cinq au Burkina Faso et au Nigéria. À l’occasion d’une réunion, les chercheurs ont assumé le rôle du président du CP, lui soumettant le projet d’ordre du jour. Après modification, le président a convoqué officiellement les autres membres du CP.

## Appréciation des retombées des CP

Les enquêtés ont évalué positivement les CP du point de vue de la composition, de l’ancrage institutionnel et du fonctionnement. Dans chaque pays, l’ancrage du CP au ministère de la Santé est perçu comme une force qui lui a permis de jouer pleinement son rôle dans les trois domaines (la facilitation de la recherche, le transfert des résultats de la recherche et l’utilisation de ces résultats). Chaque partie prenante (chercheurs, acteurs/intervenants et décideurs) se réjouissait de participer à un CP devant permettre la co-production et la co-utilisation de résultats de recherche. La constitution de ces CP dans les différents pays a créé des attentes implicites chez les parties prenantes. Au Nigéria, par exemple, le ministère fédéral de la Santé a présenté une requête officielle à l’OOAS afin de mieux comprendre le programme et d’y participer davantage. Un lien formel a été établi entre le ministère de la Santé de l’État de Delta (qui dirige le CP) et le ministère fédéral de la Santé du Nigéria. Ces liens devraient permettre une meilleure co-production d’énoncés de politique, et faciliter leur appropriation et leur utilisation. Un des indicateurs de l’adhésion des autorités sanitaires nationales au CP relève du fait d’avoir nommé/désigné par un document officiel les membres du CP, et accepté qu’une organisation spécialisée dirige le CP. Par contre, les enquêtés ont déploré le manque d’autonomie financière des CP, qui dépendaient étroitement des chercheurs, sans lesquels les CP ne pouvaient se réunir. En Sierra Leone, le fonctionnement du CP a longtemps été perturbé par l’épidémie d’Ebola (de mai 2014 à novembre 2015). Quelques enquêtés ont trouvé dommage le fait que les compétences des membres des CP n’aient pas été renforcées dans les domaines de la facilitation et du fonctionnement, de l’appui à la recherche, et du transfert et de l’utilisation des résultats de recherche. Cependant, selon les enquêtés, les différentes rencontres régionales et les visites d’appui de l’équipe de l’OOAS et d’experts techniques dans les pays ont constitué des occasions d’échanger des idées sur les approches possibles de facilitation, d’appui à la recherche, et de transfert et d’utilisation des résultats de recherche par les membres de CP présents.

## Rôles des acteurs dans la constitution et le fonctionnement des CP

Dans le cadre de cette initiative ouest-africaine, le CRDI a soutenu financièrement l’OOAS. Pour des raisons pratiques, le CRDI a aussi soutenu directement les équipes de recherche des pays aux fins de la conduite de leur recherche. Le CRDI a approuvé l’initiative de l’OOAS et sa stratégie de mise sur pied des CP par le biais du principe « agir en apprenant ». Le soutien financier du CRDI était une façon d’aider l’OOAS à explorer la valeur des CP et leur capacité à influencer les politiques et les pratiques en matière de santé en Afrique de l’Ouest. L’OOAS a joué un rôle technique et de gestion (interface sous-régionale). Du fait de son statut de structure supranationale, elle a encouragé les équipes de recherche à s’adresser à leur ministère de la Santé, et a invité les ministères à soutenir leurs chercheurs, et à faciliter la constitution, le fonctionnement et les réalisations du CP. L’OOAS a aussi suivi, à plus ou moins de distance, les activités des CP. Les ministères de la Santé avaient pour mission de faciliter la constitution et le fonctionnement des CP. Excepté le remboursement des frais de carburant et des frais de pause-déjeuner des membres des CP lors des rencontres (ces frais étaient pris en charge par les chercheurs grâce au financement du CRDI), les ministères ont pris en charge les réunions des CP en offrant des salles de réunion et en payant les charges associées (eau, électricité, frais d’entretien, équipement audio-visuel). Les chercheurs avaient pour rôle de constituer les CP en étroite collaboration avec le ministère de la Santé, de faciliter l’organisation des rencontres régulières des CP, de conduire la recherche en étroite collaboration avec les autres membres des CP, de faire régulièrement le point sur les progrès de leur recherche, et de communiquer régulièrement leurs résultats aux autres membres du CP. Les autres membres du CP avaient pour rôle de participer régulièrement aux rencontres du CP, d’orienter tout le processus de recherche afin qu’il aboutisse à la production de données à haute utilité pratique, de stimuler et faciliter le transfert de connaissances, de s’approprier ces connaissances et de les utiliser pour améliorer l’état de santé des populations ciblées.

## Difficultés rencontrées lors de la constitution ou du fonctionnement des CP

Les enquêtés du Nigéria ont déclaré qu’aucun événement majeur n’avait perturbé de façon significative la constitution de leur CP. Au Burkina Faso, l’équipe de chercheurs a mis en cause les formalités administratives et la mobilité du personnel comme des facteurs ayant ralenti le processus de création du CP.

Par ailleurs, étant donné qu’ils menaient leur recherche dans deux régions du Burkina Faso, les chercheurs burkinabés ont essayé de mettre en place un CP et deux comités régionaux. Ils se sont rendu compte après un an de flottement que ces comités régionaux fonctionneraient difficilement en raison des ressources financières limitées du projet. Cependant, l’équipe de recherche a utilisé des moyens de communication officiels et informels pour collaborer avec les responsables sanitaires des deux régions, afin de recueillir des données et de tenir les ateliers délibératifs prévus.

En Sierra Leone, les enquêtés ont reconnu avoir hésité pendant un certain temps sur l’emplacement géographique de leur CP. Pour en assurer l’efficacité et l’efficience, ils ont débattu de la question de savoir s’il fallait le positionner à Freetown ou à Makeni (capital du district de Bombali où s’est déroulé l’essentiel de la recherche). Au bout du compte, on a préféré installer le CP à Makeni, où sont basées l’équipe de recherche et les parties prenantes locales. L’équipe de recherche et l’OOAS devaient alors maintenir une communication régulière avec les responsables nationaux au ministère de la Santé, et cela été rendu possible par les fréquentes visites de l’OOAS en Sierra Leone et les rencontres officielles et informelles de l’équipe de recherche avec le ministère de la Santé. Une deuxième source d’hésitation a été le rôle ou la mission à donner au CP. Le CP devait-il réaliser la recherche ou appuyer les chercheurs dans la mise en oeuvre de la recherche ? Cette question découlait surtout de l’enthousiasme de certains membres du CP souhaitant participer directement à la collecte de données sur le terrain. Des clarifications à ce sujet ont été apportées tant par l’équipe de recherche que par l’OOAS lors des visites d’appui. Ces deux sources d’hésitation sont perçues par le consortium sierra-léonien comme des difficultés majeures ayant retardé la constitution de leur CP.

Au Sénégal, étant donné que la recherche a été menée par des enseignants chercheurs de l’Université Cheick Anta Diop, sous la gouverne du ministère de l’Enseignement supérieur, les acteurs ont eu des hésitations quant au soutien institutionnel du CP : fallait-il l’ancrer audit ministère ou à celui de la Santé et de l’Action sociale ? Face à ce dilemme, l’équipe de recherche sénégalaise a d’abord rattaché son CP au ministère de l’Enseignement supérieur. Apprenant vite de ses erreurs, elle l’a par la suite rattaché au ministère de la Santé et de l’Action sociale : « Les premiers gestes ont été posés par le ministère de l’Enseignement supérieur.

Cela n’a pas facilité la constitution de ce comité, car les gens pensaient que c’était un projet de l’université. » (membre 1 du CP du Sénégal)

Une correspondance officielle de l’OOAS adressée au ministère de la Santé du Sénégal avec copie à l’équipe de recherche a poussé cette équipe à collaborer essentiellement avec le ministère de la Santé. De plus, il faut noter qu’au-delà des rencontres officielles, des échanges réguliers par téléphone ou par courriel ont eu lieu entre l’équipe de recherche et certaines personnes-ressources du ministère de la Santé, notamment afin d’obtenir certains documents d’évaluation ou études financières déjà disponibles au ministère.

# Analyse Origine de l’idée de constituer les CP

Il est évident que dans les quatre pays (Burkina Faso, Nigéria, Sénégal et Sierra Leone) participant à cette initiative ouest-africaine, l’idée de constituer les CP a été implicitement et explicitement suggérée par l’OOAS. Cette organisation a donc stimulé et soutenu le fonctionnement des CP. Divers facteurs expliquent cette participation des partenaires techniques et financiers à la stimulation et au soutien du fonctionnement des CP en Afrique de l’Ouest. Dans ce contexte, entre autres, les chercheurs, les décideurs et les praticiens manquent souvent d’une culture professionnelle propice à la création de cadres de concertation quasi permanents pour codécider et coagir. Cependant, il arrive souvent également qu’il manque (objectivement ou subjectivement) de ressources financières pour organiser un minimum de rencontres productives. La participation d’un partenaire technique et financier a l’avantage d’apporter ces ressources, mais peut avoir l’inconvénient de construire des CP non pérennes qui ne fonctionnent que pour satisfaire ce partenaire.

La littérature scientifique [1, 5, 8, 13] montre que différents acteurs (la communauté, les autorités, les chercheurs ou les partenaires techniques et financiers) peuvent collectivement, ou en concertation avec d’autres, être à l’origine de la constitution d’un comité. L’appropriation, l’autonomie et la pérennisation d’un comité sont plus aisées si l’idée ou le besoin de le constituer et les ressources financières émanent de la communauté, des bénéficiaires ou des autorités. Elles sont plus complexes lorsque ceux-ci proviennent de chercheurs ou de leurs partenaires techniques et financiers. Dans ce dernier cas, le comité est perçu comme la « chose des chercheurs » et est très souvent lié à l’existence du projet de recherche [1, 8, 13]. Les autres acteurs non chercheurs mettent plus de temps à se l’approprier et à mobiliser les ressources nécessaires à sa pérennisation. Dans le cadre de cette initiative, la nature bénévole des travaux [24, 25] et la réduction des coûts de fonctionnement des CP devraient faciliter leur appropriation et pérennisation par les ministères de la Santé concernés, voire faciliter l’exportation du modèle dans les 11 autres pays membres de la Communauté économique des États de l’Afrique de l’Ouest (CEDEAO). Une des particularités de ce modèle de CP n’est pas le CP en tant que tel, car à des degrés divers, chaque pays a vécu des expériences positives et négatives par rapport au CP. L’originalité réside plus dans la participation d’une organisation supranationale (OOAS) soutenue financièrement par une organisation internationale (CRDI) et le rôle joué par cette dernière.

## Constitution des CP

Cette étude montre que la disponibilité ou la garantie de disponibilité de ressources financières est un levier pour le processus de constitution du CP. En réalité, sans un minimum de ressources financières, ne serait-ce que pour assurer les pauses café ou déjeuner lors des rencontres des membres de CP, il est difficile de pouvoir réunir des bénévoles pour former les comités. Pour constituer et rendre fonctionnel un comité, un minimum de ressources financières est donc requis. D’ailleurs, au sein d’un comité, le détenteur des ressources financières est aussi aux commandes des enjeux stratégiques. Dans cette expérience ouest-africaine, l’OOAS et le CRDI – en tant que partenaires techniques et financiers du consortium de chaque pays – avaient un certain pouvoir (de mobilisation, d’orientation) sur les chercheurs, qui en avaient aussi sur le reste des acteurs du consortium. C’est ainsi que les chercheurs, étant les détenteurs des ressources financières, ont été les architectes et les leaders de la constitution et du fonctionnement des quatre comités. Sans occuper le poste de président des CP dans chaque pays, les chercheurs se sont retrouvés les présidents invisibles des CP du fait qu’ils détenaient les ressources financières. Au sein des CP, rien de stratégique ne pouvait se décider sans leur consentement. Les présidents des CP fixaient la date, l’heure et l’ordre du jour des rencontres à partir des suggestions des chercheurs. C’était donc les autres acteurs non chercheurs qui devaient suivre le programme des chercheurs et non l’inverse. Cette façon de faire a ses avantages et ses inconvénients. Ils ne seront convenablement documentés qu’après l’évaluation globale de l’initiative. Le rôle de leadership des chercheurs leur avait été octroyé par l’OOAS et le CRDI par l’entremise de l’appel à notes conceptuelles et aussi par le fait qu’ils finançaient les comités. L’octroi de ce rôle illustre la confiance accordée par l’OOAS et le CRDI aux chercheurs, ce qui a sans doute influé sur la qualité de leur engagement.

Par ailleurs, l’octroi du rôle de leadership aux chercheurs illustre aussi la vision de l’OOAS, à savoir que les CP devraient être établis par les chercheurs. Nous sommes donc dans une logique de recherche (d’abord) et d’action (après).

Ces CP ont aussi été mis en place grâce à l’engagement et à la détermination de l’OOAS. Cette organisation supranationale, du fait de son statut et de ses missions, exerce une autorité politique, technique et financière sur les ministères de la Santé (pris individuellement) des pays membres de la CEDEAO. Sa participation renforce la crédibilité des consortiums, qui défendent à la fois des causes nationales et régionales. La participation d’un partenaire fort comme l’OOAS facilite la création des comités dans la mesure où la mission de chaque organisation membre du consortium d’un pays se limite essentiellement à trouver des acteurs motivés et disponibles pour jouer le rôle qu’on attend d’eux. Dans les faits, cependant, les chercheurs ou acteurs ont souvent besoin d’un soutien multiforme (informationnel, émotionnel, matériel, technique ou financier) de la part de leur partenaire pour mettre sur pied, faire fonctionner et pérenniser leur CP ou comité consultatif [13]. Pour constituer les CP dans chaque pays, les chercheurs se sont fortement inspirés de leur expérience et de leur connaissance du terrain.

La double sélection (présélection à partir de notes conceptuelles et sélection selon un protocole) a permis à l’OOAS et au CRDI de retenir les meilleurs candidats. La littérature scientifique [7, 9, 12, 13] montre que pour constituer un comité de pilotage ou un comité consultatif, lorsqu’ils méconnaissent le terrain, les chercheurs réalisent une recherche qualitative rapide à l’aide de la méthode participative ou classique pour cerner les membres potentiels de leur comité. Dans le cadre de cette initiative ouest-africaine, l’expérience des équipes de recherche et la participation du ministère de la Santé de chaque pays ont rendu cette démarche superflue.

## Facteurs ayant facilité la constitution des CP

Outre la participation de l’OOAS déjà mentionnée, l’engagement du ministère de la Santé de chaque pays a également facilité la mise en place et le fonctionnement des comités. Les ministères devraient tirer avantage de cette initiative, et c’est ce qui explique leur engagement. Par ailleurs, l’expérience des ministères ou des organisations qui forment le comité a aussi facilité la mise en place de ce dernier.

En général, plus les organisations ou personnes méconnaissent une innovation, plus elles peuvent être réticentes à y participer. L’expérience acquise dans le cadre de projets similaires renforce la confiance nécessaire pour s’engager dans une nouvelle initiative. L’ancrage des CP aux ministères de la Santé (organisations publiques et quasi pérennes) a aussi permis de rendre fonctionnels ces CP, et a facilité leur appropriation et probablement leur pérennisation par les autorités responsables des politiques en santé.

Certains facteurs liés aux chercheurs principaux (expérience de collaboration avec des membres potentiels du comité, degré de pénétration au sein du ministère de la Santé) ont beaucoup facilité la création des CP. Nous insistons sur le fait que par l’entremise de leur processus de présélection et de sélection des consortiums, l’OOAS et le CRDI se sont donné une double chance de mettre sur pied des consortiums formés des chercheurs principaux les plus expérimentés et motivés à atteindre les objectifs de l’initiative. De plus, après avoir soumis deux propositions pour être retenus, les chercheurs principaux se sentaient « responsables » des succès et des échecs du projet de leur pays, d’où leur grande détermination. Enfin, l’enthousiasme et la motivation des membres potentiels devenus membres réels des CP ont aussi facilité la création et le fonctionnement des comités.

Les membres ont été inspirés par l’engagement des chercheurs et des autorités sanitaires, par la thématique abordée et par la conception des comités. Souvent, les subordonnés adhèrent passivement ou activement, avec plus ou moins de conviction, aux initiatives déjà soutenues par leur supérieur, parce qu’ils ne veulent pas détériorer la qualité de leur relation avec leur employeur. Dans le cas de cette expérience ouest-africaine, les membres des CP ont été suffisamment motivés pour accepter de participer à titre bénévole.

En résumé, un système d’incitatifs non financiers a permis de mobiliser tous les acteurs des pays, de même que l’OOAS, en vue de la constitution et de la facilitation des comités. De façon systématique, la motivation de chaque acteur/membre motive et renforce la motivation des autres acteurs. Autrement dit, la motivation du CRDI a motivé l’OOAS. Cela a renforcé la motivation du CRDI. La motivation des partenaires techniques et financiers (CRDI/OOAS) a motivé les chercheurs, les ministères de la Santé et les autres acteurs. La motivation de ceux-ci a renforcé à son tour la motivation des partenaires techniques et financiers. Ce système de motivation est un élément central qui a favorisé la création et le fonctionnement de chaque CP avec des membres bénévoles.

La stratégie consistant à « agir en apprenant », ou la souplesse du mode de constitution des CP, a donc aussi été bénéfique, ayant permis d’adopter une approche adaptée aux contextes, aux forces et aux obstacles de chaque pays.

## Appréciations des retombées

Selon les enquêtés, la force de ce processus de constitution des CP réside dans sa composition, son ancrage institutionnel et son mode de fonctionnement. Cela a déjà été souligné dans la littérature scientifique [1, 3, 4, 6, 8, 10, 13, 24, 26-28], de même que le fait que des facteurs interpersonnels, organisationnels, humains et financiers jouent sur le succès des CP, et que l’ancrage institutionnel des CP détermine leur poids ou leur capacité à influencer les politiques. Dans le cadre de cette initiative ouest-africaine, la taille des CP et le profil des membres variaient selon les pays. Tout en les encourageant à constituer un CP, conformément au principe du « agir en apprenant », l’OOAS a laissé aux pays la liberté de choisir la façon de faire. Il n’existe pas de règle établie quant à la taille ou à la composition d’un comité [18]. La création d’un CP s’apparente à une création artistique : rien n’est laissé au hasard, mais rien ne suit non plus une règle préétablie. Le mode de constitution des CP est donc systématique, c’est à dire circulaire (des allers-retours sont possibles dans le processus), et il peut être adapté au contexte sociopolitique et organisationnel, à la thématique explorée, ainsi qu’aux besoins et aux aspirations des membres potentiels des CP dans chaque pays. La démarche est également exhaustive en ce sens qu’elle tient compte des acteurs/membres potentiels et du contexte. La flexibilité/adaptabilité de la démarche a donc facilité la création et le fonctionnement des CP. C’est ainsi que la composition des CP a varié d’un pays à l’autre en fonction du contexte. Par exemple, pour mener une recherche sur les obstacles qui empêchent les femmes enceintes d’accéder gratuitement aux établissements de santé, la Sierra Leone a constitué un comité formé de responsables des secteurs du transport, de la défense et de la sécurité.

Comme les thématiques ou faits sociaux étudiés par les consortiums touchaient plusieurs secteurs, les pays ont constitué des CP hétérogènes et multisectoriels pour trouver des solutions multisectorielles et concertées. Certes, les quatre CP n’avaient aucun lien direct entre eux. Il y a eu cependant des échanges selon les affinités de chacun. Par ailleurs, le fait que les CP des quatre pays se soient donné à peu près les mêmes missions prouve que les consortiums des pays ont communiqué entre eux directement ou par l’intermédiaire de l’OOAS, qui a en quelque sorte supervisé l’ensemble des travaux de l’initiative.

Deux faits majeurs peuvent être considérés comme des faiblesses du processus, à savoir le manque d’autonomie financière des CP, qui dépendaient étroitement des chercheurs, et le manque de formation des membres des CP dans les domaines de la facilitation et du fonctionnement de CP, de l’appui à la recherche, et du transfert et de l’utilisation des résultats de recherche. Pour tester ce modèle et en tirer des leçons, l’OOAS a voulu confier le leadership des CP aux chercheurs. Les leçons tirées seront énoncées dans le prochain rapport sur les CP.

## Rôles des acteurs dans la constitution et le fonctionnement des CP

Bien que le CRDI se soit contenté d’apporter un appui financier à l’OOAS et aux équipes/consortiums de recherche, il est perçu par la plupart des acteurs/chercheurs de cette initiative comme étant le commanditaire de la recherche conçue dans chaque pays. Beaucoup de choses sont mises en oeuvre en tenant compte des délais et des indicateurs validés par le CRDI. Au sein de certaines équipes de recherche, ce projet se distingue d’autres projets par le fait qu’il est identifié comme étant un projet du CRDI. Parfois, les choses se passent comme si les chercheurs cherchaient à satisfaire d’abord le CRDI, ensuite l’OOAS et enfin le ministère de la Santé. Autrement dit, les chercheurs se sentaient plus redevables à l’endroit du partenaire financier et technique que des partenaires collaborateurs ou bénéficiaires. Cette situation ou ce sentiment de redevabilité des chercheurs vis-à-vis les partenaires financiers s’explique par le contexte de rareté des ressources destinées à la recherche. Le détenteur des ressources financières est systématiquement aux commandes des enjeux de la recherche.

Dans le cadre de cette initiative, le pouvoir de l’OOAS a été atténué par le fait qu’elle n’était pas la structure autorisant le versement des fonds destinés aux chercheurs/consortium de chaque pays. Le fait que ces derniers obtenaient directement leurs ressources financières du CRDI et devaient rendre compte de leur utilisation à cette organisation les a amenés à se sentir plus redevables envers le CRDI qu’envers l’OOAS.

Les ministères de la Santé des différents pays se sont contentés de jouer le rôle qui leur avait été octroyé par les partenaires techniques et financiers et les chercheurs. Aucun ministère n’a fourni de ressources financières pour mener à bien l’initiative au-delà des ressources octroyées par le CRDI.

## Difficultés rencontrées lors de la constitution des CP

Contrairement aux facteurs ayant facilité la création des comités (facteurs communs aux quatre pays), les facteurs ou situations qui ont rendu difficile la création des CP étaient propres à chaque pays. À l’exception du Nigéria, les pays ont signalé des difficultés liées notamment aux formalités administratives et à la mobilité du personnel (Burkina Faso), et aux hésitations concernant l’ancrage institutionnel ou la mission du CP (Sénégal et Sierra Leone). Les enjeux relatifs à l’appartenance à un comité de pilotage et au contrôle dudit comité expliquent ces difficultés.

Tout en étant membres d’un même consortium ou comité, les acteurs, rationnels et stratégiques [29], développent des techniques plus ou moins efficaces pour contrôler le comité et accroître leur pouvoir et leurs gains. Cela a mené à des hésitations, à des luttes de pouvoir et à la complexification des procédures. Dans ce genre de situations, chaque acteur tente officiellement de donner le meilleur de lui-même et aide le groupe à mettre sur pied le meilleur comité possible. En pratique toutefois, selon Cinq-Mars [3], diverses sources d’influence, dont le désir de renforcer sa discipline ou son institution, la défense de ses intérêts et la préférence pour un modèle théorique (qui contribue à faire de leur identité sectorielle et institutionnelle la pierre angulaire), déterminent la qualité de la collaboration.

# Conclusion

L’« Initiative ouest-africaine de renforcement des capacités au moyen de la recherche sur les systèmes de santé » conçue par l’OOAS a permis la création de CP hétérogènes au Burkina Faso, au Nigéria, au Sénégal et en Sierra Leone. Pour y parvenir, la participation de l’OOAS à titre de structure supranationale, le leadership des chercheurs, l’engagement des autorités sanitaires et des membres des comités, et un système d’incitatifs non financiers ont été nécessaires. La participation des partenaires techniques et financiers (OOAS) a motivé les chercheurs et les ministères de la Santé qui, à leur tour, ont motivé les autres acteurs à s’engager bénévolement dans les CP. L’adoption de l’approche « agir en apprenant » a permis de concevoir des stratégies adaptées à chaque contexte en vue de créer, de faciliter et de faire fonctionner les CP, ainsi que de gérer les difficultés associées. Des facteurs individuels (comme le leadership des chercheurs), collectifs (comme le système de motivation réciproque), intra-pays (engagement et motivation des ministères de la Santé) et supranationaux (soutien politique et technique de l’OOAS, soutien technique et financier du CRDI) ont fortement contribué au succès du processus de création des CP. Cette expérience montre que lorsque les partenaires techniques et financiers des autorités politico-sanitaires, des chercheurs et des acteurs non chercheurs se mobilisent pour stimuler et soutenir la création et le fonctionnement de CP, ces comités ont une grande chance de fonctionner. La reproduction d’une telle expérience nécessite une maîtrise du contexte local et l’implication de partenaires forts et motivés.

#### Abréviations

CEDEAO : Communauté économique des États de l’Afrique de l’Ouest; CRDI : Centre de recherches pour le développement international; CP : comité de pilotage; OOAS : Organisation Ouest Africaine de la Santé

#### Remerciements

Les auteurs remercient les chercheurs et les membres des comités de pilotage du Burkina Faso, du Nigéria, du Sénégal et de la Sierra Leone, qui ont accepté volontairement de participer à cette étude.

#### Financement

Cette étude a été réalisée dans le cadre de la subvention accordée par le CRDI à l’Unité de soins de santé primaires de l’OOAS. Les coûts de publication ont été assumés par le CRDI.

#### Disponibilité des données et des documents

Toutes les données peuvent être obtenues des auteurs.

#### Contribution des auteurs

Tous les auteurs ont contribué de façon égale au présent article. Tous les auteurs ont lu et approuvé la version définitive du texte.

#### Intérêts conflictuels

Les auteurs déclarent n’avoir pas d’intérêts conflictuels.

#### Consentement à la publication

Ne s’applique pas.

#### Approbation éthique et consentement à la participation

Cette étude peut être considérée comme la documentation d’une expérience de constitution de CP en Afrique de l’Ouest. Elle n’est donc pas astreinte à l’examen d’un comité d’éthique. L’étude n’a été soumise à aucun comité d’éthique. Cependant elle est restée éthique depuis sa conception jusqu’à sa valorisation; elle porte sur un sujet intéressant toutes les parties prenantes, et elle a une utilité scientifique et pratique pour les pays et les acteurs concernés. La participation à l’étude a été libre et volontaire, et l’anonymat et la confidentialité ont été garantis. En bref, les principes éthiques fondamentaux ont été respectés.

#### Détails à propos des auteurs

^1^Organisation Ouest Africaine de la Santé (OOAS), 01 BP 153 Bobo-Dioulasso 01, Burkina Faso. ^2^Muraz Centre, 01 BP 390 Bobo-Dioulasso 01, Burkina Faso

Date de la publication : 12 juillet 2017

#### Références

1. Collins J: Effective committees. J Am Coll Radiol 2012, 9(3):181-4.
2. Pingaud H, Gourc D. Démarche de pilotage d’un projet industriel pour l’analyse des risques. Revue Française de Gestion Industrielle. 2004, 23(2):1-10.
3. Cinq-Mars M, Fortin D. Les enjeux de la planification participative : pouvoir à la communauté ou hégémonie des groupes dominants ? Une étude de cas. Les Cahiers Internationaux de Psychologie Sociale. 2007, 2(74):29-47.
4. Milstead JA. Are You an Asset or a Liability in Your Committee ? Ohio Nurses Rev. 2015, 9(2):8-9.
5. Uneke CJ, Ndukwe CD, Ezeoha AA, Uro-Chukwu HC, Ezeonu CT. Implementation of a health policy advisory committee as a knowledge translation platform: the Nigeria experience. Int J Health Policy Manag. 2015, 4(3):161-8.
6. Reddy P, Buchanan D, Sifunda S, James S, Naidoo N. The role of community advisory boards in health research: Divergent views in the South African experience. Sahara J. 2010, 7(3):2-8.
7. Morin SF, Maiorana A, Koester KA, Sheon NM, Richards TA. Community consultation in HIV prevention research: a study of community advisory boards at 6 research sites. J Acquir Immune Defic Syndr. 2003, 33(4):513-20.
8. Morin SF, Morfit S, Maiorana A, Aramrattana A, Goicochea P, Mutsambi JM, Robbins JL, Richards TA. Building community partnerships: case studies of community advisory boards at research sites in Peru, Zimbabwe, and Thailand. Clin Trials. 2008, 5(2):147-56.
9. Shagi C, Vallely A, Kasindi S, Chiduo B, Desmond N, Soteli S, Kavit N, Vallely L, Lees S, Hayes R et al. A model for community representation and participation in HIV prevention trials among women who engage in transactional sex in Africa. AIDS Care. 2008, 20(9):1039-49.
10. Shubis K, Juma O, Sharifu R, Burgess B, Abdulla S. Challenges of establishing a Community Advisory Board (CAB) in a low-income, low-resource setting: experiences from Bagamoyo, Tanzania. Health Res Policy Syst. 2009, 7:16.
11. NIMH Multisite HIV/STD Prevention Trial for African American Couples Group. The role of Community Advisory Boards (CABs) in Project Eban. J Acquir Immune Defic Syndr. 2008, 49 Suppl 1:S68-74.
12. Vallely A, Shagi C, Kasindi S, Desmond N, Lees S, Chiduo B, Hayes R, Allen C, Ross D. The benefits of participatory methodologies to develop effective community dialogue in the context of a microbicide trial feasibility study in Mwanza, Tanzania. BMC Public Health. 2007, 7:133.
13. Berthé A, Traoré I, Somé J, Berthé-Sanou L, Salouka S, Rouamba J, Mayaud P, Nagot N, Méda N. L’expérience burkinabè de constitution d’un Comité Consultatif Communautaire (CCC) pour un meilleur accompagnement des projets de recherche sur le VIH. Revue de Santé Publique. 2013, 25(6):829-37.
14. Nathan S, Johnston L, Braithwaite J. The role of community representatives on health service committees: staff expectations vs. reality. Health Expect. 2011, 14(3):272-84.
15. Lidz CW, Simon LJ, Seligowski AV, Myers S, Gardner W, Candilis PJ, Arnold R, Appelbaum PS. The participation of community members on medical institutional review boards. J Empir Res Hum Res Ethics. 2012, 7(1):1-6.
16. Lemire N, Souffez K, Laurendeau M-C. Animer un processus de transfert des connaissances : Bilan des connaissances et outil d’animation. Québec: Institut national de santé publique du Québec. 2009.
17. Choi BC, Pang T, Lin V, Puska P, Sherman G, Goddard M, Ackland MJ, Sainsbury P, Stachenko S, Morrison H et al. Can scientists and policy makers work together ? J Epidemiol Community Health. 2005, 59(8):632-7.
18. Sauerborn R, Nitayarumphong S, Gerhardus A. Strategies to enhance the use of health systems research for health sector reform. Trop Med Int Health. 1999, 4(12):827-35.
19. Baumbusch JL, Kirkham SR, Khan KB, McDonald H, Semeniuk P, Tan E, Anderson JM. Pursuing common agendas: a collaborative model for knowledge translation between research and practice in clinical settings. Res Nurs Health. 2008, 31(2):130-40.
20. Albert MA, Fretheim A, Maiga D. Factors influencing the utilization of research findings by health policy-makers in a developing country: the selection of Mali’s essential medicines. Health Res Policy Syst. 2007, 5:2.
21. Hanney SR, Gonzalez-Block MA, Buxton MJ, Kogan M. The utilisation of health research in policy-making: concepts, examples and methods of assessment. Health Res Policy Syst. 2003, 1:2.
22. Mucchielli A. Dictionnaire des méthodes qualitatives en sciences humaines Paris: Armand Colin. 2009.
23. Luckerhoff J, Guillemette F. Méthodologie de la théorisation enracinée. Québec : Presse de l’Université du Québec. 2012.
24. Pellikka PA. Increasing member engagement. J Am Soc Echocardiogr. 2012, 25(10):A19-20.
25. Weissman NJ. The importance of volunteering. J Am Soc Echocardiogr. 2014 27(10):13A-4A.
26. Brongers K. [How do you have influence as a member on the policy of the KNMvD ?]. Tijdschr Diergeneeskd. 2013, 138(12):20-1.
27. Katz MC. Membership: it is not just analog anymore. Conn Med. 2013, 77(8):505-06.
28. Wijesinghe PR, Palihawadana P, Peiris TS. Participatory decision-making through the Advisory Committee on Communicable Diseases: the Sri Lankan experience. Vaccine. 2010, 28 Suppl 1:A96-103.
29. Crozier M, Friedberg E. L’acteur et le système: les contraintes de l’action collective. Paris: Editions du Seuil. 1992.
