# Supplementary material for: People and research: improved health systems for West Africans, by West Africans - report on special supplement
Source: BMC Proc. 2019 Feb 7;13(Suppl 1):1. doi: 10.1186/s12919-019-0162-0 (PMC6366023; doi:10.1186/s12919-019-0162-0)
Supplement: Supplementary file 11 — Amélioration des processus d’élaboration de politiques sur la santé des mères et des enfants au Nigéria: une évaluation des besoins des décideurs, des obstacles et des éléments facilitateurs de l’élaboration de politiques fondées sur des données probantes, Uneke, C., Sombie, I., Keita, N., Lokossou, V., Johnson, E., Ongolo-Zogo, P. [file 12919_2019_162_MOESM11_ESM.docx]

***Amélioration des processus d’élaboration de politiques sur la santé des mères et des enfants au Nigéria : une évaluation des besoins des décideurs, des obstacles et des éléments facilitateurs de l’élaboration de politiques fondées sur des données probantes***

**Chigozie J Uneke^1^**^§^**, Issiaka Sombie^2^, Namoudou Keita^2^, Virgil Lokossou^2^, Ermel Johnson^2^, Pierre Ongolo-Zogo^3^**

1. Knowledge Translation Platform, African Institute for Health Policy & Health Systems Studies, Ebonyi State University, PMB 053 Abakaliki Nigéria, courriel : unekecj@yahoo.com, Tél. : +234 08038928597
2. Organisation ouest-africaine de la santé, 175, avenue Ouezzin Coulibaly, 01 BP 153 Bobo-Dioulasso 01, Burkina Faso
3. Hôpital Central de Yaoundé, CDBPH Laurence VERGNE, 2^e^ étage, avenue Henry Dunant, Messa Yaoundé, Cameroun

^§^Auteur-ressource, [unekecj@yahoo.com](mailto:unekecj@yahoo.com)

**Résumé**

**Contexte :** Au Nigéria, l’intérêt pour le processus d’intégration des données probantes dans les politiques s’affirme chez les décideurs qui participent au programme de santé des mères, des nouveau-nés et des enfants (SMNE). Toutefois, il subsiste de nombreuses lacunes chez les décideurs en ce qui a trait à l’utilisation de données issues de la recherche dans l’élaboration de politiques. La présente étude vise à évaluer les perceptions des décideurs qui participent au programme de SMNE en ce qui a trait à leurs besoins, aux obstacles et aux éléments facilitateurs en regard de l’utilisation des données de recherche dans l’élaboration de politiques au Nigéria.

**Méthodologies**: La conception de l’étude repose sur une évaluation transversale des perceptions qui a été entreprise lors d’un événement de mobilisation nationale des parties prenantes en SMNE organisé à Abuja, au Nigéria. Un questionnaire conçu pour évaluer les perceptions des participants a été administré en personne. On a aussi tenu des consultations de groupe axées sur les besoins des décideurs en ce qui concerne le processus d’intégration des données probantes dans les politiques afin d’améliorer l’utilisation de données probantes dans l’élaboration de politiques.

**Résultats :** Au total, 40 participants ont rempli le questionnaire et participé aux consultations de groupe. D’après les répondants, voici un aperçu des principaux obstacles cernés en lien avec l’utilisation de données probantes dans l’élaboration de politiques en matière de SMNE : l’insuffisance des capacités des organismes à mener des recherches en lien avec les politiques; des affectations budgétaires tout aussi insuffisantes pour mener des recherches en lien avec les politiques; l’indifférence des décideurs à l’égard des données probantes émanant de la recherche; une piètre diffusion des données probantes émanant de la recherche aux décideurs; et l’absence d’un forum d’interaction entre chercheurs et décideurs. Les principaux éléments facilitateurs de l’utilisation des données probantes issues de la recherche dans l’élaboration de politiques en matière de SMNE tels qu’ils sont perçus par les répondants comprennent notamment : renforcement des capacités des décideurs concernant l’utilisation des données de la recherche dans la formulation de politiques; diffusion appropriée des résultats de la recherche aux décideurs pertinents; participation des décideurs au processus de conception et d’exécution de la recherche et orientation des recherches en fonction des besoins des décideurs. Les principaux moyens cernés pour inciter les décideurs à utiliser les données probantes dans l’élaboration de politiques comprennent notamment : améliorer les compétences des décideurs en matière de technologies de l’information et de la communication (TIC), ainsi que dans l’utilisation, l’analyse et la communication des données et les activités de sensibilisation.

**Conclusion :** Pour accroître l’utilisation des données de la recherche dans l’élaboration de politiques au Nigéria, il faut mettre en place des mécanismes qui faciliteront le mouvement des données probantes jusqu’aux politiques, et se pencher sur les besoins indiqués par les décideurs. Il est aussi impératif d’améliorer les initiatives organisationnelles qui facilitent l’utilisation des données de la recherche dans l’élaboration de politiques.

**Contexte**

Depuis les dix dernières années, on constate à l’échelle mondiale un intérêt sans précédent pour la promotion de l’utilisation de données issues de la recherche pour éclairer l’élaboration de politiques dans le secteur de la santé. Cet intérêt remonte à la Résolution de l’Assemblée mondiale de la santé en 2005, qui encourageait fortement les États membres à exploiter plus efficacement la recherche sur la santé pour atteindre les Objectifs du millénaire pour le développement (OMD) des Nations Unies, et plus précisément dans les pays à faible revenu et les pays à revenu intermédiaire (PFR-PRI) [1].

L’utilisation de données de la recherche dans l’élaboration de politiques n’est plus réservée aux pays à revenu élevé, car les politiques fondées sur les données probantes prennent de plus en plus d’importance chez les décideurs des PFR-PRI [2-5]. Dans un précédent rapport, le directeur du Tanzanian Council for Science and Technology aurait déclaré, « si vous êtes pauvre, vous avez bien plus besoin de données probantes avant d’investir que si vous êtes riche » [6]. Des réunions internationales tenues à Mexico en 2004 et plus tard, à Bamako, au Mali, en 2008 ont également insisté sur l’importance de promouvoir la réalisation et l’utilisation de recherches essentielles sur les systèmes de santé, de s’assurer de la confiance du public dans la recherche et de combler l’écart entre le savoir et l’action dans les pays en développement [7, 8].

Même si on reconnaît dans le monde entier à quel point il est nécessaire de faire appel aux données de la recherche pour éclairer les politiques en matière de santé, plusieurs rapports prouvent sans équivoque que les politiques en matière de santé, dans la majorité des PFR-PRI ne sont pas fondées sur des données issues de la recherche [9, 10]. Pour Holmes et collaborateurs [11], l’écart entre les données probantes issues de la recherche et celles qui sont appliquées dans les soins de santé se creuse trop pour que l’on puisse l’ignorer. Oxman et collaborateurs [12] ont fait valoir qu’un processus décisionnel mal renseigné compte parmi les raisons qui expliquent pourquoi les services échouent parfois à atteindre ceux qui en ont le plus besoin, pourquoi les indicateurs de santé accusent un recul, et aussi pourquoi bon nombre de PFR-PRI sont incapables d’atteindre les OMD en matière de santé.

Le Nigéria compte parmi les PFR-PRI qui ont été incapables d’atteindre les OMD en matière de santé. Avec une population actuelle dépassant les 160 millions, l’Organisation mondiale de la Santé (OMS) a classé en 2000 les systèmes de santé du pays au 187^e^rang sur les 191 États membres [13]. Depuis lors, les incidences en matière de santé et plus particulièrement celles qui sont liées à la SMNE sont demeurées sous-optimales. Les rapports disponibles indiquent que le Nigéria affiche plus de 10 % de tous les décès maternels et d’enfants de moins de cinq ans – soit plus d’un million de décès de nouveau-nés, de nourrissons et d’enfants et plus de 50 000 décès maternels chaque année [14-16].

On a néanmoins enregistré quelques améliorations au cours de la dernière décennie au chapitre des incidences de la SMNE au Nigéria. Le taux national de mortalité maternelle (TMM) serait passé de 800 décès/100 000 naissances vivantes en 2005 [16, 17] à 576 décès/100 000 naissances vivantes en 2013 [18]. De plus, le taux de mortalité des enfants de moins de cinq ans au Nigéria est passé de 201 décès pour 1 000 naissances vivantes en 2003 [19, 20] à 117 décès pour 1 000 naissances vivantes en 2013 [21]. Cette amélioration des incidences en matière de SMNE au Nigéria peut être attribuée en partie à la reconnaissance croissante de l’importance de l’élaboration de politiques fondées sur des données probantes par les décideurs [22-24], et à la mise en oeuvre de diverses politiques relatives à la SMNE, dont certaines sont clairement fondées sur des données de recherche [25-28]. D’autres facteurs comme l’amélioration des systèmes de santé, le leadership/la gouvernance dans le secteur de la santé, la hausse du financement dans le domaine de la santé, ont contribué à la réduction du taux national de mortalité maternelle au cours de la dernière décennie [18].

Le processus consistant à utiliser les données de la recherche dans les politiques et les pratiques, autrement dit à réduire l’écart entre la théorie et la pratique, n’est pas une mince affaire, et au Nigéria on reconnaît que le processus est complexe [22, 29]. Cela s’explique du fait que le processus d’intégration des données probantes dans les politiques se caractérise par de nombreux obstacles et éléments facilitateurs qui sont propres à chaque pays et à chaque contexte. Parmi les obstacles souvent cités, on note l’absence de recherches pertinentes pour les politiques, l’absence de soutien politique, une faible structure administrative pour l’élaboration de politiques, le manque de décideurs formés à l’accès et à l’utilisation de données probantes, ainsi que la faible demande de la part des décideurs pour des données scientifiques [30-32]. Parmi les principaux éléments facilitateurs, on note l’amélioration du financement des travaux de recherche pertinents pour l’élaboration de politiques, un accès facile aux résultats de recherche pertinents pour l’élaboration de politiques, la communication et le réseautage entre décideurs et chercheurs, la diffusion à grande échelle de la recherche et l’amélioration des compétences des décideurs en ce qui concerne l’élaboration de politiques fondées sur des données probantes [30, 32]. Plusieurs rapports précédents ont clairement montré que pour réussir la mise en oeuvre de stratégies destinées à combler l’écart entre la théorie et la pratique, il est très important de déterminer les obstacles et les éléments facilitateurs de l’utilisation de la recherche dans l’élaboration de politiques dans chaque cadre différent [33-35].

On constate un urgent besoin de conception et d’exécution de stratégies et de programmes d’intervention particuliers qui s’attaqueront aux obstacles à l’utilisation des données probantes dans l’élaboration de politiques dans le contexte nigérian. D’après l’OMS [36], pour assurer la mise en oeuvre d’un mécanisme de données probantes-réaction qui soit ciblé et efficace, une évaluation approfondie des besoins est requise, et une telle évaluation devrait tenir compte des besoins en matière de données probantes et des priorités relatives des parties prenantes, à l’intérieur des pays et entre les pays, ainsi qu’une évaluation systématique des lacunes dans les données probantes au sujet de la santé des mères et des enfants dans la région.

Même si l’intérêt pour l’élaboration de politiques fondées sur les données probantes et la conscience de son existence s’affirment au Nigéria, les points de rencontre – comme des événements destinés à engager les décideurs à s’intéresser aux enjeux entourant l’interface entre la recherche et les politiques en lien avec la SMNE – sont pour ainsi dire inexistants. Il est pertinent de mentionner que les données probantes pour l’élaboration de politiques peuvent provenir, en plus de la recherche, de connaissances et de renseignements, d’idées et d’intérêts, ainsi que du contexte plus vaste de la politique et de l’économie [37]. Bowen et ZI [38], ont indiqué que les données probantes englobent la recherche, et sont susceptibles d’inclure les opinions et les points de vue d’individus ou de groupes, les résultats de processus de consultation, de même que les documents et les rapports publiés. Toutefois, les données probantes issues de la recherche scientifique ont constamment été présentées comme faisant partie des catégories les plus fiables de données dans l’élaboration et la mise en oeuvre de politiques en matière de santé capables de produire de meilleures incidences dans le domaine [39, 40]. La présente étude a donc mis l’accent sur l’utilisation de données de recherche dans l’élaboration de politiques. La présente étude vise à évaluer les perceptions des décideurs du programme de SMNE en regard de leurs besoins, des obstacles et des éléments facilitateurs de l’utilisation des données de recherche dans le cadre de l’élaboration de politiques au Nigéria. L’étude s’insère dans l’effort visant à promouvoir le processus d’intégration des données probantes dans les politiques et les pratiques pour améliorer les incidences en matière de santé des mères et des enfants au Nigéria.

**Méthodologies**

**Méthodologie et participants**

La méthodologie suivie est une évaluation transversale des perceptions des décideurs et autres parties prenantes clés en regard de leurs besoins, des obstacles et des éléments facilitateurs de l’utilisation des données de recherche dans le cadre de l’élaboration de politiques au Nigéria. L’étude a été menée auprès de 40 participants lors d’un événement national d’une journée destiné à mobiliser les parties prenantes en SMNE réunies sous les auspices de l’Organisation ouest-africaine de la santé (OOAS) et du Ministère fédéral de la santé (MFDS) du Nigéria, en octobre 2015, à Abuja, au Nigéria. Les participants étaient des cadres supérieurs de divers organismes qui participent au processus d’élaboration de politiques et comprenaient notamment : le ministère fédéral de la Santé à Abuja et les ministères, services et organismes connexes; les ministères de la Santé des États; des partenaires du développement; des organisations de la société civile; et des organisations non gouvernementales ainsi que des universités et des instituts de recherche. Le but de la réunion était de promouvoir l’utilisation de données de recherche dans l’élaboration de politiques et dans les pratiques en matière de SMNE. Nous n’avons pas été subventionnés pour effectuer une recherche officielle, étant donné que l’aide financière obtenue de l’OOAS visait à établir un dialogue national entre les parties prenantes. Nous avons par conséquent conçu l’événement pour déterminer s’il était possible de se procurer des renseignements susceptibles d’être considérés comme des données probantes de qualité pour renseigner de futurs efforts de renforcement des capacités et de quelle manière se les procurer. Il s’agit donc d’un exemple d’application d’une stratégie d’apprentissage empirique et d’utilisation de processus existants afin de cerner de manière plus systématique les priorités et les enjeux.

**Outil et technique de collecte des données**

***Questionnaire d’autoévaluation :*** Dans la présente étude nous avons utilisé un questionnaire d’autoévaluation fourni en annexe. Le questionnaire a été remis pendant la réunion aux 40 participants qui l’ont tous rempli et retourné. Lors de l’élaboration du questionnaire, nous nous sommes inspirés de l’outil d’autoévaluation produit par la Fondation canadienne de la recherche sur les services de santé (FCRSS) [41]. Nous avons consulté l’outil d’autoévaluation de la FCRSS en raison de rapports disponibles qui indiquaient son utilité pour évaluer la capacité individuelle et organisationnelle d’utiliser des données de recherche dans la conception et la prestation de services [24-28]. Le questionnaire a été conçu à titre d’instrument non limitatif axé sur les éléments suivants au sein de l’organisme de chaque participant; i) mécanismes, processus, outils, stratégies et plateformes existants en vue de l’utilisation de données de recherche dans l’élaboration de politiques; ii) surveillance existante, mécanismes d’évaluation et d’évaluation du rendement pour l’utilisation de données de recherche dans l’élaboration de politiques; iii) facteurs qui limitent l’utilisation de données de recherche dans l’élaboration de politiques; iv) stratégies possibles pour s’attaquer aux facteurs qui limitent l’utilisation de données de recherche dans l’élaboration de politiques.

***Consultations de groupe et présentations de groupe :*** Nous avons tenu des consultations de groupe au cours desquelles les participants étaient regroupés en fonction du type d’organisme auquel ils appartenaient. La consultation de groupe était axée sur les besoins des décideurs dans le cadre du processus d’intégration des données probantes dans les politiques afin d’améliorer l’utilisation de données dans le cadre du processus décisionnel; elle a été d’une durée de 70 minutes.

Au total, cinq consultations de groupes organisationnels ont été tenues, comme suit :

Groupe 1 : Participants du MFDS.

Groupe 2 : Participants des ministères, services et organismes.

Groupe 3 : Participants des ministères de la Santé des États.

Groupe 4 : Participants des partenaires du développement, des ONG et des OSC.

Groupe 5 : Participants des associations professionnelles et des instituts de recherche.

Chaque groupe avait entre 7 et 12 participants, et chacun était sous la direction d’un participant qui occupait un poste de cadre supérieur dans son organisme et qui avait été sélectionné par les autres participants du groupe en fonction de son expérience antérieure dans la direction de telles discussions. Nous avons fourni au leader de chaque groupe des lignes directrices pour faciliter la consultation. Les consultations de groupe n’ont pas été enregistrées, mais chaque groupe a désigné un participant chargé de prendre des notes sur les enjeux clés. Huit sujets clés ayant trait à la capacité d’utiliser des données probantes dans l’élaboration de politiques ont été abordés dans chaque groupe, et les commentaires et les résolutions ont été documentés. Les sujets ont été classés en deux catégories : capacité individuelle et capacité organisationnelle. On a demandé à chaque groupe d’articuler et de résumer les enjeux clés déterminés et ayant fait l’objet de la discussion sous la forme d’une liste énumérative et de courtes phrases. Un représentant de chaque groupe a présenté un exposé sur les résultats des consultations de groupe pendant la séance plénière. Voici la liste des questions qui ont été utilisées :

***a) Capacité individuelle***

1. Aptitudes : Pour renforcer vos aptitudes à l’utilisation de données de recherche, quelles sont les interventions qui revêtent de l’importance pour vous ?
2. Compétences : De quel genre de compétences avez-vous besoin pour mieux utiliser les données et les résultats de recherche ?
3. Sources des données probantes : Quelles sources de données probantes ou de résultats de recherche aimeriez-vous consulter pour améliorer votre utilisation des données et des résultats de recherche ?
4. Formats des données : Dans quels formats souhaiteriez-vous recevoir les données et les résultats de recherche pour en faciliter l’utilisation ?

***b) Capacité organisationnelle***

1. Milieu institutionnel : À votre avis, quel genre d’améliorations (lois, règlements, organisation du service, soutien, motivation, etc.) serait-il important d’apporter dans votre milieu de travail pour vous aider à mieux utiliser les données et les résultats de recherche ?
2. Plateformes ou mécanismes :
3. À votre avis, quel genre de plateformes ou de mécanismes serait-il important de mettre en place ou de renforcer dans votre milieu de travail pour vous faciliter l’accès aux données et aux résultats de recherche ?

b) À votre avis, quel genre de plateformes ou de mécanismes serait-il important de mettre en place ou de renforcer dans votre milieu de travail pour faciliter l’utilisation courante de données et de résultats de recherche ?

1. Occasions d’utiliser les données probantes : Quelles sont les activités dans le cadre de votre emploi du temps régulier qui constituent des occasions d’entreprendre l’utilisation permanente de données et de résultats de recherche ?
2. Besoins en matière de soutien : Quel genre de soutien souhaiteriez-vous obtenir lorsque vous décidez d’utiliser des données et des résultats de recherche ?

Un autre cycle de délibérations générales a été entrepris, et les principaux commentaires ont été articulés ensemble. La plénière a été d’une durée de 45 minutes.

**Analyse des réponses des participants**

Les réponses écrites aux questionnaires et les notes prises lors des consultations de groupe ont été analysées au moyen de l’approche phénoménologique de Giorgi [42], qui a été développée ultérieurement par Albert et collaborateurs [43]. L’analyse a été menée comme suit : i) passer en revue toute l’information textuelle; ii) repérer tous les commentaires jugés significatifs; iii) extraire les unités significatives, iv) classer et résumer les abstractions; v) retourner au texte extrait afin d’assurer une bonne correspondance.

**Résultats**

***Renseignements personnels et attributs relatifs au titre officiel***

Parmi les 92 participants, 71 (77,2 %) appartenaient à des organismes qui participent à des processus d’élaboration de politiques au Nigéria (par exemple ministère fédéral de la Santé, ministères, services et organismes, ministères de la Santé des États, partenaires du développement, ONG). Parmi les 71 participants de ces organismes, au total, 40 (56,3 %) ont signé le formulaire de consentement éclairé, ont rempli le questionnaire des décideurs et ont participé aux consultations de groupe. Le profil des 40 participants est présenté au tableau 1. Au total, 24 (60,0 %) répondants étaient des femmes, et jusqu’à 64 % des répondants étaient âgés de plus de 44 ans. Le ministère fédéral de la Santé et les ministères, services et organismes connexes regroupaient la plus grande proportion de représentants (45 %). La majorité des répondants (59 %) étaient soit directeurs soit présidents de leur organisme. La majorité des répondants affichaient leur présent titre soit depuis de trois à cinq ans (45 %) ou depuis moins de trois ans (37,5 %). Au total, 59 % des répondants ont déclaré qu’ils exerçaient une influence directe sur les processus d’élaboration de politiques. Les résultats du questionnaire et des consultations de groupe ont été combinés. Notre décision de combiner les résultats a été renseignée par le fait que la majorité des réponses des participants étaient très comparables. Cette situation laissait entendre qu’en ce qui a trait au processus d’intégration des données probantes dans les politiques, les contraintes individuelles et organisationnelles du point de vue de la capacité étaient semblables, sans égard à l’appartenance organisationnelle. À l’aide de l’approche phénoménologique de Giorgi [38], nous nous sommes concentrés davantage sur l’information textuelle et sur les commentaires qui nous paraissaient significatifs par rapport à la SMNE et au processus d’intégration des données probantes dans les politiques.

**Tableau 1. Profil des participants ayant rempli le questionnaire et pris part aux discussions de groupe lors de l’événement de mobilisation des parties prenantes**

| **Paramètre évalué** | **Incidences (%)** |
| --- | --- |
| **Genre** |  |
| Homme | 16 (40,0) |
| Femme | 20 (60,0) |
| Total | 40 |
| **Tranche d’âge** |  |
| 25 à 34 ans | 4 (11,1) |
| 35 à 44 ans | 9 (25,0) |
| >44 ans | 23 (63,9) |
| Total | 36 |
| **Type d’organisme** |  |
| MFDS/MSO | 8 (45,0) |
| MSDE (ministères de la Santé des États) | 10 (25,0) |
| ONG/OSC | 5 (12,5) |
| PD (partenaires du développement) | 3 (7,5) |
| Chercheurs | 4 (10,0) |
| Total | 40 |
| **Titre** |  |
| Directeur | 23 (59,0) |
| Gestionnaire/Chef de département | 5 (12,8) |
| Agent de programme/projet | 11 (28,2) |
| Total | 39 |
| **Durée de la nomination** |  |
| <3 ans | 15 (37,5) |
| De 3 à 5 ans | 18 (45,0) |
| De 6 à 10 ans | 5 (12,5) |
| >10 ans | 2 (5,0) |
| Total | 40 |
| **Influence sur le processus d’élaboration de politiques** |  |
| Directe | 23 (59,0) |
| Indirecte | 16 (41,0) |
| Total | 39 |

Voici un aperçu des principales conclusions, organisées en fonction du sujet.

**Capacité individuelle d’utilisation des données de la recherche**

***Aptitudes*:** Pour ce qui est du renforcement des aptitudes à l’utilisation des données probantes et des interventions qui sont importantes, les divers groupes ont insisté sur l’utilisation du renforcement des capacités, la création d’environnements porteurs à l’appui des données, et la fourniture de mesures d’encouragement ou de récompenses pour inciter les parties prenantes à promouvoir l’utilisation des données (tableau 2).

***Compétences requises :*** Les participants ont insisté sur leur besoin d’acquérir des compétences en matière de technologies de l’information et de la communication (TCI); des compétences en méthodologie de recherche et en rédaction scientifique; des compétences en collecte, utilisation et gestion de données; ainsi que des compétences en communication et en activités de sensibilisation (tableau 2).

***Sources de données :*** Les participants ont fait ressortir le besoin d’avoir accès à des publications, y compris à des revues à comité de lecture et à des articles divers, tant en version imprimée qu’électronique. Ils ont également mentionné le besoin d’établir des bases de données locales de recherches et de données probantes, ainsi que des forums de diffusion pour les résultats de recherche (tableau 2).

***Formes de données probantes :*** Les participants ont reconnu qu’ils avaient besoin de données probantes sous diverses formes : énoncés de politique, articles dans la presse, bulletins d’information, vidéos, histoires de réussite, témoignages, mise en commun des connaissances et mécanismes de rétroaction (tableau 2).

**Capacité organisationnelle d’utilisation des données de la recherche**

***Le milieu institutionnel devrait améliorer l’utilisation des données probantes :*** De manière générale, les participants s’entendaient pour dire qu’il était nécessaire de créer des comités ou des lignes directrices en matière d’éthique ou de réglementation de la recherche. Ils ont également souligné le besoin de pouvoir compter sur de la documentation appropriée concernant la recherche, notamment le recensement des données, une surveillance et une évaluation améliorées, l’établissement de bibliothèques bien gérées avec du personnel de liaison chargé de distribuer les recherches ou rapports, et l’adoption de lois et de réglementation concernant l’utilisation de la recherche ainsi qu’un engagement politique (tableau 3).

***Plateformes ou mécanismes pour faciliter l’utilisation de données probantes :*** Les participants ont indiqué le besoin de mettre en place des mécanismes de réseautage et de collaboration permanents entre chercheurs et décideurs. Ils ont également mentionné les besoins suivants : système d’information de gestion en matière de santé (SIGS)/base de données fonctionnelle, connectivité Internet et bibliothèque en ligne (tableau 3).

***Occasions d’utiliser les données probantes :*** Les principaux secteurs offrant des occasions d’utiliser les données de la recherche en vue d’apporter des changements de politique déterminés par les participants comprenaient notamment : rédaction de rapports, rédaction de propositions, planification de programmes, processus décisionnel, élaboration de politiques, mise en oeuvre d’activités, et surveillance et évaluation d’activités (tableau 3).

***Besoins en matière de soutien à l’utilisation de données probantes :*** Les participants ont indiqué les domaines suivants dans lesquels ils auraient besoin de soutien pour pouvoir utiliser les données de recherche : ressources financières pour réaliser les recherches, ressources humaines (notamment soutien technique, compétences en matière de statistique), technologies de l’information et communication, plan ou stratégie efficace de communication pour diffuser la recherche ou les données probantes, engagement politique, réseautage et collaboration, infrastructure matérielle qui créera un milieu de travail propice et des occasions de participer à des conférences (tableau 3).

***Initiatives organisationnelles pertinentes pour l’élaboration de politiques fondées sur des données probantes :*** Les réponses des parties prenantes concernant les initiatives organisationnelles (mécanismes, processus, outils, stratégies et plateformes) pertinentes pour l’élaboration de politiques fondées sur des données probantes sont présentées au tableau 4. Les réponses des parties prenantes ont montré que les principaux mécanismes visant à utiliser les données probantes font appel à la gestion des connaissances stratégiques, au fonctionnement du service de la planification, de la recherche et des statistiques, au conseil d’examen institutionnel et aux mécanismes externes de rétroaction des parties prenantes ou des examens. Les principaux processus relatifs à l’utilisation de données probantes comprenaient notamment : engagement des parties prenantes pertinentes, participation d’intervenants clés dans certains cas, détermination du problème, formation de l’intervention de recherche, diffusion des résultats et suivi régulier des indicateurs de programme. Les principaux outils liés à l’utilisation des données probantes comprenaient notamment : formulaires à utiliser lors de la collecte systématique de données, données de recherche, lignes directrices à l’intention des travailleurs; Service d’information de gestion en matière de santé (SIGS), énoncés de recherche et d’évaluation et fiches de rendement. Les principales stratégies concernant l’utilisation des données probantes comprenaient notamment : évaluation des résultats des données présentées; réunion avec les parties prenantes; renforcement des capacités; réunions de consultation et activités de sensibilisation fondées sur des données probantes. Les principales plateformes comprenaient notamment : division de la recherche du service de gestion des connaissances stratégiques; utilisation du comité des groupes de travail; forum des parties prenantes; plateforme d’apprentissage en ligne pour les aptitudes liées à la recherche; dépôts de données; service d’utilisation de la recherche et systèmes d’archives internes (passerelle des pratiques exemplaires et réunions de gestion ou du programme SharePoint) (tableau 4).

***Principaux obstacles à l’utilisation des données probantes :*** D’après les répondants, les principaux obstacles à l’utilisation des données probantes dans l’élaboration de politiques relatives à la SMNE comprenaient notamment : aucun moyen/mécanisme systématique d’utilisation de la recherche dans les interventions en SMNE; capacité inadéquate de l’organisme de mener des recherches susceptibles d’être utilisées; affectations budgétaires limitées ou insuffisantes pour effectuer de la recherche; aucune politique écrite demandant au personnel de fonder son travail sur des données probantes; les décideurs ne sont pas intéressés par les faits fondés sur des données probantes, et ce, même lorsque des résultats de recherche sont disponibles; la recherche est assujettie aux règles des bailleurs de fonds; les priorités de recherche sont déterminées par les bailleurs de fonds; piètre diffusion des résultats et des données de recherche documentés; aucun forum d’interaction entre chercheurs et décideurs (tableau 5).

***Principaux éléments facilitateurs de l’utilisation de données probantes :*** Les principaux éléments facilitateurs de l’utilisation de données de la recherche en vue de l’élaboration de politiques en matière de SMNE comprenaient notamment : renforcement des capacités concernant l’utilisation de la recherche dans la formulation de politiques; adoption de politiques visant à assurer des affectations budgétaires suffisantes pour la recherche; élaboration d’une politique pour assurer que toute nouvelle politique introduite sera accompagnée de données probantes; diffusion appropriée des résultats de la recherche aux parties prenantes pertinentes; utilisation d’énoncés de politique, participation des décideurs à la recherche; détermination par les décideurs des domaines de recherche en fonction des besoins; création d’un forum annuel pour la présentation et l’étude des travaux ou des résultats de recherche en vue d’une éventuelle adoption et intégration au processus d’élaboration de politiques (tableau 5).

**Tableau 2. Réponses des participants sur les besoins individuels concernant les capacités requises pour utiliser les données probantes dans les politiques pertinentes pour la santé des mères, des nouveau-nés et des enfants**

| ***Aptitudes*** | ***Compétences*** | ***Sources des données probantes*** | ***Formes des données probantes*** |
| --- | --- | --- | --- |
| i). Renforcement des capacités, par exemple formation, stages pour former des agents subalternes, fourniture du matériel nécessaire pour travailler  ii). Mesures d’encouragement comme des récompenses, des promotions  iii). Réseautage au moyen du partage de l’information  iv). Coordination intersectorielle et intrasectorielle  v). Création d’un environnement porteur qui favorise l’utilisation des données probantes en milieu de travail  vi). Capacité de produire des données probantes de haut niveau à partir d’ECR, de recherches sur les interventions et d’examens systématiques.  v). Capacité de déterminer les domaines pertinents ou les lacunes en matière de recherche pour les politiques | i). Compétences en matière de technologies de l’information et de la communication  ii). Collecte de renseignements, analyse et interprétation de données  iii). Compétences en communication, tant à l’oral qu’à l’écrit  iv). Rédaction et communication dans le domaine scientifique  v). Méthodologie de la recherche  vi). Compétences en matière de sensibilisation  vii). Capacité individuelle de reconnaître les types de données probantes, les sources de données probantes et le niveau de ces données  viii). Capacité de repérer les données de la recherche (extraire, synthétiser et présenter les données de la recherche)  ix). Compétences en matière d’examen critique de la documentation, compréhension de base de la statistique | i). Publications-revues, enquêtes, livres  ii). Versions papier et électroniques en bibliothèque  iii). Ressources électroniques ou en ligne pour les articles et les revues  iv). Forums de diffusion des résultats de la recherche  v). Élaboration de bases de données locales pour la recherche. | i). Versions papier – résumés et rapports textuels  ii). Bulletins d’information et vidéos  iii). Cas de réussites et témoignages  iv). Énoncés de politique/feuillets  V). Réunions scientifiques et publications des actes de conférences.  vi). Mise en commun des connaissances et mécanismes de rétroaction |

**Tableau 3. Réponses des participants sur les besoins organisationnels concernant les capacités requises pour utiliser les données probantes dans les politiques pertinentes pour la santé des mères, des nouveau-nés et des enfants**

| ***Contexte institutionnel*** | ***Plateformes ou mécanismes*** | ***Occasions d’utiliser les données probantes*** | ***Besoins en matière de soutien*** |
| --- | --- | --- | --- |
| i). Lignes directrices en matière d’éthique/réglementation  ii). Documentation adéquate de la recherche, qui comprend notamment le recensement des données  iii). Amélioration des processus de suivi et d’évaluation  iv). Organisation d’un forum de parties prenantes pour la diffusion de résultats de recherche  v). Amélioration du financement pour des travaux de recherche pertinents pour les politiques  vi). Volonté politique en vue de l’application de la législation nationale sur la santé, de la politique nationale sur la santé et de la politique nationale sur la recherche en matière de santé  vii). Création d’un centre de ressources ou d’une bibliothèque bien gérée avec personnel de liaison chargé de diffuser les recherches/rapports et les données probantes au personnel  viii). Adoption de lois/règlements sur l’utilisation des données de la recherche | i). Site Web pour le stockage des travaux de recherche et pour permettre leur consultation.  ii). Création d’une base de données nationale  iii). Nominations conjointes entre universitaires (institutions de recherche) et ministère de la Santé (par exemple congés sabbatiques/détachements)  iv). SGIS/base de données fonctionnels  v). Mécanismes redditionnels au niveau des États et national  vi). Fourniture de l’accès à une connectivité Internet et à une bibliothèque en ligne  vii). Disponibilité de plateformes pour permettre des interactions régulières entre décideurs et institutions de recherche  viii). Utilisation d’énoncés de politique, de feuillets d’information, etc. | i). Changement de politiques  ii). Rédaction de propositions et de rapports  ii). Suivi et évaluation des activités  iv). Utilisation des pratiques exemplaires et évaluation de leur utilisation pour fournir une rétroaction et soulever d’autres questions de recherche  v). Planification de programmes et mise en oeuvre d’activités  vi). Prise de décisions et élaboration de politiques | i). Plan/stratégie de communication efficace pour diffuser les recherches/données probantes (y compris aux organismes professionnels)  ii). Réseautage et collaboration  iii). Infrastructure matérielle, milieu de travail propice  iv). Financement et disponibilité d’outils et de ressources pour mettre en oeuvre les politiques fondées sur des données probantes  v). Ressources financières pour réaliser les recherches, etc.  vi). Ressources humaines, soutien technique, compétences en matière de statistique  vii). Technologies de l’information et de la communication  viii). Logistique, comme moyens de transport, etc.  ix). Occasions d’assister à des conférences et séminaires |

**Tableau 4.** **Sommaire des réponses des participants sur l’existence d’initiatives organisationnelles (mécanismes, processus, outils, stratégies et plateformes) pertinentes pour l’élaboration de politiques fondées sur des données probantes concernant la santé des mères, des nouveau-nés et des enfants**

| **a). Mécanismes** | **b). Processus** | **c). Outils** | **d). Stratégies** | **e). Plateformes** |
| --- | --- | --- | --- | --- |
| i)- Collecte de données en matière de SMNE  ii). Gestion des connaissances stratégiques  iii). Département de la planification, de la recherche et des statistiques  iv). Réunion des parties prenantes pour discuter des enjeux  v). Conseil d’examen institutionnel  vi). Mécanisme de rétroaction pour les parties prenantes externes ou les examens  vii). Conférences annuelles | i). Mobilisation des parties prenantes pertinentes  ii). Détermination des problèmes, formation d’intervention de recherche, diffusion des résultats  iii). Processus d’évaluation des propositions de recherche  iv). Définition des priorités de la recherche  v). Suivi régulier des indicateurs de programme | i). Formulaires à utiliser lors de la collecte de données  ii). Lignes directrices à l’intention des travailleurs  iii). Service d’information sur la gestion de la santé (SIGS)  iv). Divers outils de données adaptés pour le MFDS  v). Énoncés de recherche et d’évaluation  vi). Fiches de rendement;  vii). Revues d’associations | i). Évaluation des résultats des données soumises  ii). Formation et renforcement des capacités  iii). Réunion de consultation avec les parties prenantes  iv). Bibliothèque électronique pour accéder aux revues internationales  v). Diffusion interne de données probantes au programme  vi). Sensibilisation fondée sur des données probantes | i). Division de la recherche du Département de gestion des connaissances stratégiques  ii). Réunions de l’équipe de gestion  iii). Utilisation du comité des groupes de travail  iv). Réunions du comité technique principal pour la SMNE  v). Forum des parties prenantes  vi). Plateformes d’apprentissage électroniques et dépôts de données  vii). Département d’utilisation de la recherche  viii). Systèmes d’archives internes |

**Tableau 5. Sommaire des réponses des participants sur les obstacles et les éléments facilitateurs de l’utilisation des données de la recherche dans l’élaboration de politiques concernant la santé des mères, des nouveau-nés et des enfants.**

| Principaux obstacles à l’utilisation des données de la recherche | Principaux éléments facilitateurs de l’utilisation des données de la recherche |
| --- | --- |
| - Aucun moyen/mécanisme systématique en vue de l’utilisation de la recherche dans les interventions en matière de SMNE  - Capacité insuffisante de l’organisme d’effectuer des travaux de recherche susceptibles d’être utilisés  - Financement ou affectations budgétaires insuffisants pour effectuer de la recherche  - Certaines incidences de la recherche pourraient ne pas être d’une grande utilité dans les programmes étant donné qu’elles pourraient ne pas répondre aux questions précises que nous nous posons  - Aucune politique écrite ne demande au personnel de fonder leurs notes de service, propositions, etc., sur des données probantes  - Installations inadéquates en vue de la mise en oeuvre - Capacités insuffisantes des décideurs  - Corruption et ingérence politique  - Piètre volonté politique;  - Faiblesse des liens entre chercheurs et décideurs  - Absence de renforcement des capacités chez les décideurs quant à l’importance de la recherche  - Piètre diffusion des résultats et des données probantes de la recherche documentée  - Non-participation des décideurs au tout début de la recherche  - Non-communication des incidences de la recherche aux décideurs  - Pas de forum d’interaction entre chercheurs et décideurs  - La recherche est assujettie aux règles des bailleurs de fonds  - Les priorités de la recherche sont établies par les bailleurs de fonds | - Renforcement des capacités des décideurs concernant l’utilisation de la recherche dans la formulation de politiques  - Création d’une politique prévoyant des affectations budgétaires pour la recherche  - Création d’une politique rendant obligatoire l’utilisation des données probantes dans l’élaboration de politiques  - Affiliation avec un établissement universitaire dans le secteur de la production de données probantes, de leur interprétation et de leur utilisation  - Davantage de formation ou de réunions des parties prenantes auxquelles participent les décideurs  - Intégration des décideurs au moment de planifier et d’élaborer les projets de recherche  - Diffusion appropriée des résultats de la recherche aux parties prenantes pertinentes  - Une politique devrait être instituée pour assurer que chaque mise en application de projets du gouvernement soit fondée sur des données probantes  - Renforcement des départements de la Planification, de la recherche et des stratégies en matière de santé pour qu’ils puissent jouer un rôle efficace dans la promotion des travaux de recherche et leur utilisation  - Création d’un forum annuel en vue de la présentation et de l’étude de travaux ou de résultats de recherche en vue d’une adoption possible et intégration au processus d’élaboration de politiques  - Création d’un forum entre universitaires et décideurs  - Faire en sorte que les besoins des décideurs guident les projets de recherche |

**Discussion**

La présente étude a permis de cerner les besoins perçus par les décideurs et autres parties prenantes participant à l’élaboration de politiques en matière de SMNE au Nigéria de même que les obstacles et les éléments facilitateurs de l’élaboration de politiques fondées sur des données probantes. À notre connaissance, il s’agissait de la première fois que des décideurs nationaux, des chercheurs et autres parties prenantes en matière de SMNE se réunissaient au Nigéria pour étudier des enjeux entourant l’interface entre la recherche et les politiques ainsi que pour déterminer les secteurs où les besoins sont prioritaires à l’appui de l’utilisation des données de la recherche dans l’élaboration de politiques. Dans un rapport publié en 2009, Lavis et collaborateurs [44] ont indiqué constater un intérêt croissant pour la détermination des mécanismes qui améliorent la mise en commun interactive des connaissances, laquelle permet de réunir les données de la recherche avec les points de vue, les expériences et les connaissances tacites de ceux qui participeront aux décisions futures, ou qui seront touchés par ces décisions futures, concernant des enjeux hautement prioritaires. Ils ont cité deux rapports publiés par Lavis [45] et Lomas [46] lesquels indiquaient que l’intérêt pour la détermination de mécanismes interactifs de mise en commun des connaissances a été alimenté par la reconnaissance du besoin d’un système d’aide à la décision contextuel et adapté localement à l’intention des décideurs et autres parties prenantes pour améliorer le processus d’intégration des données probantes dans les politiques.

Les secteurs où les besoins sont vitaux en matière d’intégration des données probantes dans les politiques pour les décideurs en SMNE du Nigéria ont été repérés dans le cadre de la présente étude, et ces renseignements faciliteront l’élaboration de stratégies pour combler ces besoins. Les résultats d’études précédentes ont clairement montré que l’utilisation optimale des données de la recherche n’est pas possible sans prendre en considération les besoins, les préoccupations et le degré de réceptivité des utilisateurs potentiels de ces connaissances [47, 48]. Dans cette étude, les participants ont insisté sur l’utilisation du renforcement des capacités pour améliorer leurs compétences en ce qui a trait à l’élaboration de politiques fondées sur les données probantes, la création d’environnements porteurs à l’appui des données probantes, ainsi que l’adoption de mesures d’encouragement ou de récompenses pour inciter les parties prenantes à promouvoir l’utilisation des données probantes. Il est bien établi que les compétences dans l’utilisation des données probantes pourraient être améliorées au moyen de la formation et de programmes de perfectionnement à l’intention des décideurs et autres agents responsables des politiques. En effet, la majorité des décideurs ayant participé à cette étude ont indiqué le manque de formation comme leur principale contrainte en matière de capacité.

La formation en vue d’acquérir de nouvelles compétences axées sur les données probantes est très importante et éduquer les représentants de l’administration qui peuvent ensuite introduire de nouvelles approches à la prise de décision dans leur organisme constitue un important moyen d’entraîner un changement systémique [49]. On ne saurait trop insister sur l’importance du perfectionnement des capacités chez les décideurs et autres parties prenantes dans le secteur de la santé nigérian. Il s’agit d’un facteur majeur susceptible de stimuler l’intérêt pour le transfert et l’intégration des données de la recherche dans les politiques et les pratiques. Cela ne manquera pas d’influencer positivement la gouvernance et le leadership, les ressources (humaines, matérielles et financières), la communication et la qualité de la recherche [22, 23, 29, 50]. C’est déjà un fait bien établi que la formation axée sur des compétences pourrait aider les décideurs et leurs employés non seulement à déterminer les données de recherche qui sont pertinentes pour les politiques, mais aussi à repérer les qualités plus ou moins élevées des recherches sur le plan méthodologique [51-53].

Parmi les compétences les plus importantes dont les participants ont besoin pour améliorer l’utilisation de données de recherche dans l’élaboration de politiques, il convient de mentionner les compétences en TIC, en méthodologie de recherche, en rédaction scientifique et en analyse de données. Les résultats d’enquêtes précédentes ont montré qu’il existe un lien entre le bon rendement d’un intervenant dans le secteur de la santé (décideur ou fournisseur de services) et l’utilisation des TIC [54-56]. Dzenowagis [55] a fait remarquer que les TIC ont considérablement amélioré l’accès aux renseignements et à la recherche sur la santé, ce qui soutient les organismes de recherche en santé et facilite l’élaboration de politiques complètes et axées sur les données probantes. Plusieurs études réalisées au Nigéria ont révélé que le manque de capacités suffisantes en matière de TIC chez les décideurs constitue un frein majeur à l’intégration des données de la recherche au processus d’élaboration de politiques [57-59]. Peizer [60] a fortement recommandé d’investir beaucoup de temps et de ressources dans la formation visant à améliorer les compétences en matière de TIC des personnes qui contribuent à l’élaboration de politiques en santé. C’est parce que la compétence en matière de TIC améliorera la capacité de rédaction scientifique, d’analyse et de gestion de données ainsi que la détermination des données probantes de la recherche.

Les participants à cette étude ont mentionné que le processus d’intégration des données probantes dans les politiques pourrait être stimulé au Nigéria par l’établissement de mécanismes de réseautage et de collaboration permanents entre chercheurs et décideurs. Un tel partenariat entre chercheurs et décideurs a été décrit comme un élément crucial pour promouvoir l’utilisation de la recherche en santé dans l’élaboration de politiques dans d’autres contextes [61, 62]. Ce partenariat exige une attention et une considération plus soutenues dans les pays en développement, y compris au Nigéria, où son utilité potentielle n’a pas été entièrement évaluée. Hyder et collaborateurs [63] ont fait remarquer que le processus consistant à transposer les incidences de la recherche en politiques est essentiel et pourtant, ce processus demeure relativement peu étudié dans la majorité des pays en développement. Ils ont en outre indiqué que les mécanismes, tant formels qu’informels utilisés pour effectuer cette transposition, ainsi que les types de personnes en cause, plus particulièrement dans les entités comme les services chargés des politiques en matière de santé, méritent tous d’être examinés [63].

Dans une étude antérieure réalisée au Nigéria, des décideurs avaient déclaré que pour promouvoir le processus d’intégration des données probantes dans les politiques, il faudrait que les décideurs participent à la planification et à l’exécution des recherches en matière de santé, et que les chercheurs participent à la planification et à l’exécution des programmes de santé. De plus, le dialogue entre chercheurs et décideurs devrait être encouragé, plus particulièrement au moyen d’une tribune commune ou de réunions, et les méthodologies applicables à la recherche en santé devraient être simplifiées de manière à pouvoir être facilement comprises par les décideurs [21, 29].

Parmi les domaines très importants dans lesquels les participants ont indiqué avoir des besoins, il faut mentionner ceux du format et du mécanisme de communication et de diffusion des données de la recherche, lesquels devraient être adaptés de manière à répondre aux besoins des décideurs. On a mentionné l’utilisation possible d’énoncés de politique à titre d’exemple d’outils de mise en commun des connaissances susceptible d’encourager les décideurs à recevoir des données de recherche et à s’en servir. Dans une étude récente sur les efforts relatifs à l’emballage de l’information à l’appui de l’élaboration de politiques fondées sur des données probantes dans les PFR-PRI, Adam et coll. [2] ont fait remarquer que l’on reconnaît de plus en plus l’importance d’élaborer des documents concis et des outils pour communiquer divers types de renseignements aux décideurs. Selon eux, c’est grâce à cette reconnaissance que l’on assiste à une pléthore d’efforts en matière d’emballage de l’information, lesquels visent à appuyer les processus d’intégration des données probantes dans les politiques et les pratiques en se fondant sur les messages renseignés par les résultats de la recherche [5, 64, 65]. Lavis et collaborateurs [44] ont indiqué que les énoncés de politique comptent parmi les types les plus intéressants d’outils d’emballage de l’information et qu’ils constituent une nouvelle approche en vue de l’amélioration du processus d’élaboration de politiques en appuyant l’intégration des données probantes dans les politiques. Des rapports récents en provenance du Nigéria ont montré que l’utilisation d’énoncés de politique pour promouvoir l’élaboration de politiques fondées sur des données probantes est bien accueillie par les décideurs [24, 66], et que cela s’explique du fait que cela leur facilite à eux ainsi qu’aux autres parties prenantes l’exercice consistant à déterminer si et comment les données de la recherche qui sont disponibles correspondent à leurs propres croyances, valeurs, intérêts ou buts et stratégies politiques [44].

Il convient de mentionner que les participants ont insisté sur la nécessité que le gouvernement s’engage sur le plan politique et qu’il prévoie des ressources financières pour appuyer les processus d’intégration des données probantes dans les politiques, de même que l’infrastructure matérielle et les milieux de travail propices. Ces besoins sont étayés par plusieurs rapports récents, lesquels ont clairement montré l’existence d’un lien entre l’intérêt politique, le financement et l’infrastructure pour promouvoir l’intégration de la recherche dans les politiques [31, 67]. Selon Deans et Ademokun [67], mis à part la capacité d’utiliser la recherche pour élaborer des politiques fondées sur des données probantes, de nombreux autres facteurs, y compris la volonté politique et les contraintes de financement, freinent la possibilité que les politiques soient renseignées par des données probantes. Dans une étude qui étudiait les obstacles à l’utilisation de la recherche dans la formulation des politiques en Égypte, la disponibilité de fonds pour soutenir la mise en oeuvre des résultats (90,7 %) et les intérêts politiques (81,3 %) se sont classés comme les plus importants facteurs qui freinent le processus d’élaboration de politiques [30]. De plus, El-Jardali et collaborateurs [31] ont indiqué que 79,2 % des répondants de leur étude avaient signalé que le financement public limité pour le secteur de la santé et les valeurs des partis au pouvoir (53,9 %) exerçaient une forte influence sur le processus d’élaboration de politiques. Des données probantes issues d’études antérieures ont montré qu’en améliorant l’infrastructure de recherche, l’engagement politique et les ressources financières en regard du processus d’intégration des données probantes dans les politiques on pouvait créer un environnement porteur en vue de l’utilisation des données de la recherche dans l’élaboration de politiques et les pratiques [61, 69].

Les participants ont formulé une autre remarque digne de mention, à savoir que les chercheurs devraient passer leur congé sabbatique dans les ministères du gouvernement, les services et les organismes pour accroître les interactions entre décideurs et chercheurs. À l’inverse, les décideurs devraient être encouragés à passer un certain temps dans les institutions de recherche. Ce type de stratégie d’échange de personnel, ou de programme de détachement, possède le potentiel d’améliorer les interactions entre décideurs et chercheurs.

On a déjà montré que les détachements offrent de bonnes occasions d’améliorer le développement personnel et les méthodes de travail du personnel de première ligne grâce à des rencontres directes utiles au cours desquelles les personnes en détachement sont exposées à des concepts, des valeurs et des cultures nouvelles qui sont susceptibles de mettre à l’épreuve leur capacité de réussir dans un contexte différent [6]. Les détachements peuvent donc offrir un moyen positif de motiver les personnes et d’accroître la satisfaction au travail, tout en améliorant les pratiques exemplaires, les partenariats professionnels collaboratifs, les connaissances et les compétences [70]. Même si, en règle générale, le recours aux détachements est vu comme une stratégie de perfectionnement des compétences comportant des avantages mutuels pour l’organisme et pour l’individu, il existe toutefois des lacunes dans la documentation concernant les considérations liées à l’application pratique et aux facteurs essentiels à la réussite en lien avec l’utilisation d’un détachement à titre de stratégie globale pour promouvoir l’élaboration de politiques de santé fondées sur des données probantes et une stratégie de mise en œuvre [71]. Cela mérite que l’on s’y attarde davantage dans le cadre d’études futures.

Il est intéressant de mentionner que les répondants ont reconnu l’existence de quelques initiatives organisationnelles, notamment des mécanismes, des processus, des outils, des stratégies et des plateformes qui sont susceptibles d’améliorer l’utilisation des données de la recherche dans l’élaboration de politiques. Certains rapports antérieurs ont décrit l’existence d’initiatives organisationnelles comparables conçues pour promouvoir l’utilisation de données de recherche dans l’élaboration de politiques [32, 48, 72]. Un exemple important est celui de l’initiative Données probantes et systèmes de santé au Nigéria (NEHSI) qui a été créée dans le but de mettre sur pied un système de santé ouvert aux données probantes, en mettant l’accent sur les soins de santé primaires, en vue d’améliorer les incidences pour la santé des mères et des enfants [73-76]. Le MFDS et les 36 ministères de la Santé des États au Nigéria disposent de deux importantes unités appelées Service de la planification, de la recherche et des statistiques (DPRS) et Division du service d’information de gestion de la santé (HMIS). Les décideurs ayant participé à la présente étude ont admis que ces unités comptent parmi les principales initiatives conçues pour promouvoir l’élaboration de politiques fondées sur les données probantes parce que les unités en question participent à ce qui suit : i) détermination des problèmes et formation d’intervention de recherche; ii) diffusion des résultats et suivi régulier des indicateurs de programme; iii) dépôts de données et système d’archives internes; iv) gestion des connaissances stratégiques et utilisation de la recherche; v) suivi et évaluation de programme et évaluation du rendement.

Toutefois, il est pertinent de mentionner que l’existence de ces initiatives organisationnelles – que l’on peut voir comme des mesures d’encouragement ou des motivations à utiliser la recherche – ne garantissent pas leur engagement efficace ni leur utilisation par les décideurs. L’un des principaux facteurs qui peut être responsable de l’engagement inadéquat de ces initiatives organisationnelles pourrait être le manque de capacités de recherche de base et de compétences chez les décideurs. Il s’agit d’un important facteur qui a été décrit dans la présente étude comme un obstacle majeur à l’utilisation des données de la recherche dans l’élaboration de politiques, et qui semble être un dénominateur commun dans bien des pays à faible revenu et pays à revenu intermédiaire [9, 10, 22, 32]. Dans une étude réalisée antérieurement, on faisait remarquer que la demande pour les données de recherche n’est pas influencée seulement par les mesures d’encouragement ou les motivations des décideurs de se servir de la recherche mais, ce qui importe encore davantage, par leur capacité d’avoir accès à la recherche, de la comprendre et de l’utiliser [77].

En plus d’une capacité de recherche inadéquate, d’autres obstacles importants à l’utilisation des données de la recherche dans l’élaboration de politiques mentionnés par les participants à la présente étude correspondent à ceux qui sont indiqués dans plusieurs autres études ayant évalué les obstacles perçus par les décideurs à l’utilisation de données probantes dans l’élaboration de politiques [32, 62, 78-80]. À n’en pas douter, les obstacles indiqués dans la présente étude pourraient jouer un rôle important dans l’incapacité à élaborer une politique de santé efficace fondée sur des données probantes en lien avec la SMNE au Nigéria. Ils pourraient aussi être responsables du manque d’engagement adéquat et d’utilisation des nombreuses initiatives organisationnelles instituées pour promouvoir l’utilisation des données de recherche dans le processus d’élaboration de politiques au Nigéria. D’après Davis et Davis [81], ainsi qu’Ellen et collaborateurs [82], ces obstacles ne sont pas faciles à surmonter, mais les occasions d’apprentissage et leur évaluation sont des mécanismes susceptibles d’être utiles dans l’établissement d’occasions de s’y attaquer.

Fait intéressant, tous les principaux éléments facilitateurs de l’utilisation de données de recherche dans l’élaboration de politiques indiqués par les décideurs dans la présente étude concordent avec ce qui est signalé dans de nombreuses études antérieures [32, 62, 80]. Les décideurs ont insisté fortement sur la nécessité du renforcement des capacités concernant l’utilisation de la recherche dans la formulation de politiques, la diffusion appropriée des résultats de recherche aux parties prenantes pertinentes et la participation des décideurs à la recherche. Dans plusieurs études, les décideurs ont constamment suggéré que la mise en place de ces éléments facilitateurs pourrait améliorer l’intégration des données de recherche au processus d’élaboration de politiques [78-81].

**Limites de l’étude**

La présente étude comporte des limites. Premièrement, la méthode d’autoévaluation que nous avons choisie a la réputation de comporter un biais lié à l’estime de soi, pourrait ne pas être fiable, et est difficile à valider [83]. Faisant ressortir les faiblesses de cette technique, Deans et Ademokun [67] ont indiqué qu’être en mesure de comprendre et de reconnaître de façon critique ses propres lacunes en matière de compétences et de connaissances est un processus difficile qui exige une réflexion guidée. La deuxième limite est notre incapacité à employer l’analyse de contenu interactionnelle des incidences des consultations de groupe. Cela est préconisé dans des études futures. On reconnaît que ces limites peuvent avoir réduit la quantité et la profondeur des renseignements utiles qui auraient pu résulter de la présente étude; cependant, nous sommes d’avis que la qualité des données n’en a pas souffert. Les constatations peuvent servir de première étape fiable vers l’élaboration d’une stratégie d’intervention efficace pour améliorer le processus d’intégration des données probantes dans les politiques en matière de SMNE au Nigéria.

**Conclusion**

Les renseignements fournis par les répondants à cette étude ont révélé les secteurs où les besoins se font sentir, ainsi que les obstacles et les éléments facilitateurs concernant l’utilisation des données de la recherche dans l’élaboration de politiques au Nigéria. Ces renseignements sont très utiles dans le cadre de l’élaboration de stratégies d’intervention précises, tant au niveau individuel qu’organisationnel, qui faciliteront le processus d’intégration des données probantes dans les politiques. Pour améliorer le processus d’élaboration de politiques fondées sur des données probantes, il est important de perfectionner les initiatives organisationnelles qui promeuvent l’utilisation des données de la recherche, par exemple la commande de recherches par les organismes responsables de l’élaboration de politiques. Il importe aussi d’améliorer l’infrastructure de recherche, le financement, la formation des décideurs et l’établissement de plateformes durables pour favoriser les interactions entre décideurs et chercheurs. Les interventions de renforcement tant individuelles qu’institutionnelles pourraient promouvoir l’élaboration de politiques fondées sur des données probantes, et ce, même si les études qui évaluent l’incidence de ces dernières sur les processus liés aux politiques sont pour ainsi dire inexistantes. Des études futures pour évaluer l’incidence des interventions conçues pour s’attaquer aux obstacles associés à l’intégration des données probantes dans les processus d’élaboration de politiques sont préconisées.

**Abréviations**

**SMNE :** Santé des mères, des nouveau-nés et des enfants

**Contribution des auteurs**

Tous les auteurs ont participé à la conception et à l’élaboration de l’étude. CJU a rédigé le manuscrit, tous les autres auteurs ont apporté leur contribution à la version définitive du manuscrit.

**Remerciements**

Les auteurs souhaitent remercier le ministère fédéral de la Santé, à Abuja, au Nigéria ainsi que tous les décideurs politiques, chercheurs et autres parties prenantes en matière de SMNE au Nigéria qui ont participé à la présente étude.

**Considérations d’ordre éthique**

Les attestations de conformité à l’éthique pour la présente étude ont été délivrées par le Comité d’éthique de la recherche universitaire de l’Université de l’État d’Ebonyi, au Nigéria (l’institution de l’auteur principal). La délivrance des attestations reposait sur la garantie que la participation à la recherche était volontaire et fondée sur le consentement libre et éclairé; que l’anonymat des participants serait préservé et que tous les résultats demeureraient rigoureusement confidentiels et serviraient uniquement aux fins de la recherche. Toutes ces conditions ont été remplies. Tous les participants ont fourni leur consentement éclairé puisqu’ils ont rempli et signé le formulaire de consentement.

**Consentement à la publication**

Sans objet

**Disponibilité des données et des documents**

Les données seront fournies sur demande

**Intérêts conflictuels**

Les auteurs déclarent n’avoir pas d’intérêts conflictuels.

**Financement**

La présente étude est l’une des incidences du projet *Transformer en politiques les données probantes concernant la santé des mères, des nouveau-nés et des enfants en Afrique de l’Ouest* ​​entrepris par l’Organisation ouest-africaine de la santé (OOAS) avec l’aide financière du Centre de recherches pour le développement international (CRDI) du Canada (Référence : CRDI 107892_001).

**Références**

1. Assemblée mondiale de la Santé, *Fin de l’Assemblée mondiale de la Santé : adoption de résolutions cruciales pour la santé publique dans le monde,* cinquante-huitième session, Genève, Organisation mondiale de la Santé, 2005.

2. Adam, T., K.A. Moat, A. Ghaffar et J.N. Lavis, « Towards a better understanding of the nomenclature used in information-packaging efforts to support evidence-informed policymaking in low- and middle-income countries », *Implement Sci,* vol. 9, n^o^ 67, 2014.

3. Panisset, U., T.P. Koehlmoos, A.H. Alkhatib, T. Pantoja, P. Singh, J. Kengey-Kayondo, et B. McCutchen, « Implementation research evidence uptake and use for policy-making », *Health Res Policy Syst*, vol. 10, 2012, p. 20.

4. Koon, A.D., K.D. Rao, N.T. Tran et A. Ghaffar, « Embedding health policy and systems research into decision-making processes in low- and middle-income countries », *Health Res Policy Syst*, vol. 11, 2013, p. 30.

5. Rosenbaum, S.E., C. Glenton, C.S. Wiysonge, E. Abalos, L. Mignini, T. Young, F. Althabe, A. Ciapponi, S.G. Marti, Q. Meng, J. Wang, A.M. la Hoz Bradford, S.N. Kiwanuka, E. Rutebemberwa, G.W. Pariyo, S. Flottorp et A.D. Oxman, « Evidence summaries tailored to health policy-makers in low- and middle-income countries », *Bull World Health Organ*, vol. 89, n^o^ 1, 2011, p. 54-61.

6. A.D. Oxman, A. Bjørndal, F. Becerra-Posada, M. Gibson, M.A. Block, A. Haines, M. Hamid, C.H. Odom, H. Lei, B. Levin, M.W. Lipsey, J.H. Littell, H. Mshinda, P. Ongolo-Zogo, T. Pang, N. Sewankambo, F. Songane, H. Soydan, C. Torgerson, D. Weisburd, J. Whitworth et S. Wibulpolprasert, « A framework for mandatory impact evaluation to ensure well informed public policy decisions », *Lancet*, vol. 375, n^o^ 9712, 2010, p. 427-31.

7. Organisation mondiale de la Santé, *World Report on Knowledge for Better Health-Strengthening Health Systems,* Genève, Organisation mondiale de la Santé, 2004.

8. Alliance pour la recherche sur les politiques et les systèmes de santé, *Briefing Note Number 1: What is Health Policy and Systems Research and why does it matter?*, Genève, Organisation mondiale de la Santé, 2007.

9. González-Block, M.A. et A. Mills, « Assessing capacity for health policy and systems research in low and middle income countries », *Health Res Policy Syst*, vol. 1, n^o^ 1, 2003, p. 1.

10. Shroff, Z., B. Aulakh, L. Gilson, I.A. Agyepong, F. El-Jardali et A. Ghaffar, « Incorporating research evidence into decision-making processes: researcher and decision-maker perceptions from five low- and middle-income countries », *Health Res Policy Syst*, vol. 13, p. 70, 2015.

11. Holmes, B.J., M. Schellenberg, K. Schell et G. Scarrow, « How funding agencies can support research use in healthcare: an online province-wide survey to determine knowledge translation training needs », *Implement Sci*, vol. 9, 2014, p. 71.

12. Oxman, A.D., J.N. Lavis, S. Lewin et A. Fretheim, « SUPPORT Tools for evidence-informed health Policymaking (STP) 1: What is evidence-informed policymaking? », *Health Res Policy Syst.*, vol. 7 (suppl. 1), 2009, p. S1.

13. Organisation mondiale de la Santé, *Rapport sur la santé dans le monde 2000 : Pour un système de santé plus performant*, Genève, Organisation mondiale de la Santé, 2000.

14. United States Agency for International Development, *Working Toward the Goal of Reducing Maternal and Child Mortality: USAID Programming and Response*, Washington (D.C.), USAID, 2008.

15. Galandanci, H., C. Ejembi, Z. Iliyasu, B. Alagh et U. Umar, « Maternal health in Northern Nigeria— a far cry from ideal », *BJOG*, vol. 114, 2007, p. 448-52.

16. Federal Ministry of Health. *Integrated Maternal, Newborn and Child Health Strategy*, Abuja, Federal Ministry of Health, 2007.

17. Ebonyi State Mother and Child Care Initiative (MCCI) Nigeria, Documentation commissioned by The United Nations Population Fund (UNFPA), Final Report, Abuja, UNFPA, 2010.

18. National Population Commission of Nigeria et ICF International, *Nigeria Demographic and Health Survey 2013*, Abuja (Nigeria) et Rockville (Maryland), États-Unis, NPC et ICF International, 2014.

19. National Population Commission of Nigeria, *Nigeria Demographic and Health Survey 2008*, Abuja, National Population Commission et ICF Macro, 2009.

20. National Population Commission et ORC Macro, *Nigeria Demographic and Health Survey 2003*, Calverton (MD), National Population Commission (NPC) et ORC Macro, 2004.

21. Banque mondiale, Taux de mortalité infantile, moins de 5 ans (pour 1 000), Banque mondiale, 2015. Sur Internet : [https://donnees.banquemondiale.org/indicateur/SH.DYN.MORT](http://data.worldbank.org/indicator/SH.DYN.MORT).

22. Uneke, C.J., A.E. Ezeoha, C.D. Ndukwe, P.G. Oyibo, F. Onwe, E.B. Igbinedion et P.N. Chukwu, « Individual and organisational capacity for evidence use in policy making in Nigeria: an exploratory study of the perceptions of Nigeria health policy makers », *Evidence & Pol*, vol. 7, n^o^ 3, 2011, p. 251-276.

23. Uneke, C.J., A.E. Ezeoha, C.D. Ndukwe, P.G. Oyibo et F. Onwe, « Promotion of evidence-informed health policymaking in Nigeria: bridging the gap between researchers and policymakers », *Global Public Health*, vol. 7, n^o^ 7, 2012, p. 750-65.

24. Onwujekwe, O., N. Uguru, G. Russo, E. Etiaba, C. Mbachu, T. Mirzoev et B. Uzochukwu, « Role and use of evidence in policymaking: an analysis of case studies from the health sector in Nigeria », *Health Res Policy Syst*, vol. 13, p. 46, 2015.

25. Federal Ministry of Health, *Nigeria’s Call to Action to Save Newborn Lives*, Abuja, Federal Ministry of Health, 2015.

26. Banque mondiale, *Saving One Million Lives Initiative Nigeria*, Banque mondiale, 2015.

27. Federal Ministry of Health, *National Guidelines for the Prevention of Mother to Child Transmission of HIV (PMTCT)*, Abuja, Federal Ministry of Health, 2010.

28. Partnership for reviving routine immunization in northern Nigeria-maternal, newborn & child health (PRRINN-MNCH) initiative, *Kangaroo Training Guidelines for Low Birth Weight Babies*, Abuja, PRRINN-MNCH initiative, 2010.

29. Uneke, C.J., A. Ezeoha, C.D. Ndukwe, P.G. Oyibo et F. Onwe, « Development of Health Policy and Systems Research in Nigeria: Lessons for Developing Countries’ Evidence-Based Health Policy Making Process and Practice », *Healthc Pol*, vol. 6, n^o^ 1, 2010, p. 48-65.

30. Abou-Zeid, A., Y. Galal, M. Shawky et M. El-Rabbat, « Exploring Barriers to Research Utilization in Policy Formulation in Egypt: Researchers’ Perspectives », *J Am Sci*, vol. 8, n^o^ 12, 2012, p. 43-49.

31. [El-Jardali, F.](http://www.ncbi.nlm.nih.gov/pubmed/?term=El-Jardali%20F%5BAuthor%5D&cauthor=true&cauthor_uid=22799440), [J.N. Lavis](http://www.ncbi.nlm.nih.gov/pubmed/?term=Lavis%20JN%5BAuthor%5D&cauthor=true&cauthor_uid=22799440), [N. Ataya](http://www.ncbi.nlm.nih.gov/pubmed/?term=Ataya%20N%5BAuthor%5D&cauthor=true&cauthor_uid=22799440), [D. Jamal](http://www.ncbi.nlm.nih.gov/pubmed/?term=Jamal%20D%5BAuthor%5D&cauthor=true&cauthor_uid=22799440), [W. Ammar](http://www.ncbi.nlm.nih.gov/pubmed/?term=Ammar%20W%5BAuthor%5D&cauthor=true&cauthor_uid=22799440) et [S. Raouf](http://www.ncbi.nlm.nih.gov/pubmed/?term=Raouf%20S%5BAuthor%5D&cauthor=true&cauthor_uid=22799440), « Use of health systems evidence by policymakers in eastern Mediterranean countries: views, practices, and contextual influences », [BMC Health Serv Res](http://www.ncbi.nlm.nih.gov/pubmed/?term=Use+of+health+systems+evidence+by+policymakers+in+eastern+mediterranean+countries%3A+views%2C+practices%2C+and+contextual+influences), vol. 12, 2012, p. 200.

32. Oliver, K., S. Innvar, T. Lorenc, J. Woodman et J. Thomas, « A systematic review of barriers to and facilitators of the use of evidence by policymakers », *BMC Health Serv Res*, vol. 14, 2014, p. 2.

33. Graham, I.D., J. Logan, M.B. Harrison, S.E. Straus, J. Tetroe, W. Caswell et N. Robinson, « Lost in knowledge translation: time for a map? », *J Contin Educ Health Prof*, vol. 26, n^o^ 1, 2006, p. 13-24.

34. Graham, I.D. et J. Tetroe, « Some theoretical underpinnings of knowledge translation », *Acad Emerg Med*, vol. 14, n^o^ 11, 2007, p. 936-941.

35. Lavis, J.N, « Research, public policymaking, and knowledge translation processes: Canadian efforts to build bridges », *J Contin Educ Health Prof*, vol. 26, n^o^ 1, 2006, p. 37-45.

36. Organisation mondiale de la Santé, *Asia-Pacific Leadership and Policy Dialogue for Women’s and Children’s Health. Responding to Evidence Requests for Policies and Programmes*, Genève, Organisation mondiale de la Santé, 2012.

37. Mirzoev, T., A. Green, N. Gerein, S. Pearson, P. Bird, B.T.T. Ha, K. Ramani, X. Qian, [M. Mukhopadhyay et W. Soors,](http://pure.itg.be/en/persons/werner-soors(e4bd444c-1c2b-4e8f-8cbb-dc263944d707).html) [« Role of evidence in maternal health policy processes in Vietnam, India and China: findings from the HEPVIC project »](http://pure.itg.be/en/publications/role-of-evidence-in-maternal-health-policy-processes-in-vietnam-india-and-china-findings-from-the-hepvic-project(f80f54c3-bb10-4bbd-b15d-7d37345a68c9).html), *Evidence and Policy 2013,* vol. 9, n^o^ 4, p. 493-511.

38. S. Bowen et A.B. Zwi, « Pathways to “evidence-informed” policy and practice: a framework for action », *PLoS Med.*, vol. 2, n^o^ 7, 2005, p. e166.

39. Dobrow, M.J., V. Goel et R.E.G. Upshur, « Evidence-Based Health Policy: Context and Utilisation », *Soc Sci Med*, vol. 58, n^o^ 1, 2004, p. 207-17.

40. Hanney, S.R., M.A. Gonzalez-Block, M.J. Buxton et M. Kogan, « The Utilization of Health Research in Policy-Making: Concepts, Examples and Methods of Assessment », *Health Res Pol Systems*, vol. 1, 2003, p. 2-29.

41. Fondation canadienne de la recherche sur les services de santé (FCRSS), *Validating the Foundation’s Self-assessment tool: A summary*, Ottawa (Ontario), FCRSS, 2008. Sur Internet :

[https://www.cfhi-fcass.ca/Libraries/Documents/SAT-Outil-autoevaluation.sflb.ashx](http://www.cfhi-fcass.ca/Libraries/Documents/SAT-Self-Assessment-Tool.sflb.ashx).

42. Giorgi, A., « Sketch of a Psychological Phenomenological Method », *in* Giorgi, A. (dir.), *Phenomenology and Psychological Research: Essays*, Pittsburgh, Duquesne University Press, 1985, p. 8-22.

43. Albert, M.A., A. Fretheim et D. Maïga, « Factors Influencing the Utilization of Research Findings by Health Policy-Makers in a Developing Country: The Selection of Mali’s Essential Medicines », *Health Res Policy Syst*, 2007, vol. 5, p. 2.

44. Lavis, J.N., G. Permanand, A.D. Oxman, S. Lewin et A. Fretheim, « SUPPORT Tools for evidence-in-formed health policymaking (STP) 13: preparing and using policy briefs to support evidence-in-formed policymaking », *Health Res Policy Syst*, 2009, vol. 7, p. S13.

45. Lavis, J.N., « Moving forward on both systematic re-views and deliberative processes », *Health Policy*, vol. 1, 2006, p. 59-63.

46. Lomas, J. « Decision support: a new approach to making the best healthcare management and policy choices », *Healthc Q*, vol. 10, 2007, p. 16-18.

47. Hanney, S.R., M.A. Gonzalez-Block, M.J. Buxton et M. Kogan, « The utilization of health research in policy-making: concepts, examples and methods of assessment », *Health Res Policy Syst*, vol. 1, 2003, p. 2-29

48. Dagenais, C., « Knowledge transfer in community-based organizations: A needs assessment study », *Global Journal of Community Psychology Practice*, 2010, vol. 1, n^o^ 2, 13-30.

49. Green, A. et Bennett, S. (dir.), *Sound choices: enhancing capacity for evidence-informed health policy*, Genève, Organisation mondiale de la Santé, 2007.

50. Uneke, C.J., A.E. Ezeoha, C.D. Ndukwe, P.G. Oyibo et F. Onwe, « Enhancing Leadership and Governance Competencies to Strengthen Health Systems in Nigeria: Assessment of Organizational Human Resources Development » *Healthcare Policy*, 2012, vol. 7, n^o^ 3, p. 76-87.

51. Uneke, C.J., A.E. Ezeoha, H. Uro-Chukwu, C.T. Ezeonu, O. Ogbu, F. Onwe et C. Edoga, « Enhancing health policymakers’ information literacy knowledge and skill for policymaking on control of infectious diseases of poverty in Nigeria », *Online J Public Health Inform*, 2015, vol. 7, n^o^ 2, p. e221.

52. Uneke, C.J., A.E. Ezeoha, H. Uro-Chukwu, C.T. Ezeonu, O. Ogbu, F. Onwe et C. Edoga, « Improving Nigerian health policymakers’ capacity to access and utilize policy relevant evidence: outcome of information and communication technology training workshop », *Pan Afr Med J*, vol. 21, 2015, p. 212.

53. Uneke, C.J., A.E. Ezeoha, H. Uro-Chukwu, C.T. Ezeonu, O. Ogbu, F. Onwe et C. Edoga, Enhancing the capacity of policy-makers to develop evidenceinformed policy brief on infectious diseases of poverty in Nigeria, *Int J Health Policy Manag*, vol. 4, n^o^ 9, 2015, p. 599-610.

54. Chetley, A., *Improving Health, Connecting People: the Role of ICTs in the Health Sector of Developing Countries. A Framework Paper*, Information for Development Program (Infodev), Working paper No. 7., Royaume-Uni, Health Link Worldwide, 2006.

55. Dzenowagis, J., *Bridging the digital divide: linking health and ICT policy*, Ottawa, Centre de recherches pour le développement international, 2009.

56. Conseil économique et social des Nations Unies et Commission économique pour l’Afrique des Nations Unies, *Information and communication technology for health sector : the African Development Forum ‘99 post ADF summit*, comité de l’information pour le développement de la Commission économique pour l’Afrique des Nations Unies, (2^e^ réunion, 4-7 septembre 2001, Addis-Ababa, Éthiopie), Addis-Ababa, août 2001.

57. Alabi, G.A., *Case Study Effectiveness of Informatics Policy Instruments in Africa: Nigeria*, Addis-Ababa, Commission économique pour l’Afrique, 1994.

58. Akande, J.O., P.O. Jegede, « Andragogy and Computer Literacy: The Nigerian Perspective », *The African Symposium*, vol. 4, 2004, p. 2.

59. Uneke, C.J., A.E. Ezeoha, C.D. Ndukwe, P.G. Oyibo et F. Onwe, « Enhancing health policymakers’ capacity to use information and communication technology in Nigeria », *J Health Inform Dev Countr*, vol. 5, n^o^ 2, 2011, p. 228-246.

60. Peizer, J, *Bridging the digital divide: first you need the bridge*, 2000. Sur Internet : https://www.socialtext.net/m/page/ourmedia/bridging_the_digital_divide.

61. Campbell, D.M., S. Redman, L. Jorm, M. Cooke, A.B. Zwi et L. Rychetnik, « Increasing the use of evidence in health policy: practice and views of policy makers and researchers », *Aust New Zealand Health Policy*, vol. 6, 2009, p. 21.

62. Innvær, S., G. Vist, M. Trommald et A. Oxman, « Health policy-makers perceptions of their use of evidence: a systematic review », *J Health Serv Res Policy*, vol. 7, 2002, p. 239-244.

63. Grignon, J.S., J.H. Ledikwe, D. Makati, R. Nyangah, B.W. Sento et B. Semo, « Maximizing the benefit of health workforce secondment in Botswana: an approach for strengthening health systems in resource-limited settings », *Risk Manag Healthc Policy*, vol. 7, 2014, p. 91-98.

64. Lavis, J.N., « How can we support the use of systematic reviews in policymaking? » *PloS Med*, vol. 6, n^o^ 11, 2009, p. e1000141.

65. Lavis, J.N., J. Lomas, M. Harnid et N.K. Sewankambo, Assessing country-level efforts to link research to action, *Bull World Health Organ*, vol. 84, 2006, p. 620-628.

66. Uneke, C.J., A.E. Ezeoha, H. Uro-Chukwu, C.T. Ezeonu, O. Ogbu, F. Onwe et C. Edoga, « Promoting Evidence to Policy Link on the Control of Infectious Diseases of Poverty in Nigeria: Outcome of A Multi-Stakeholders Policy Dialogue », *Health Promot Perspect*, vol. 5, n^o^ 2, 2015, p. 104-115.

67. Deans, F. et A. Ademokun, *Investigating capacity to use evidence*, Royaume-Uni, International Network for the Availability of Scientific Publications (INASP), 2004.

68. Holmes, B.J., M. Schellenberg, K. Schell et G. Scarrow, « How funding agencies can support research use in healthcare: an online province-wide survey to determine knowledge translation training needs », *Implement Sci*, vol. 9, 2014, p. 71.

69. Santesso, N. et P. Tugwell, « Knowledge translation in developing countries », *J Contin Educ Health Prof*, vol. 26, 2006, p. 87-96.

70. MHW UK, *Understanding Secondments in MHW*, Royaume-Uni, Health & Safety Dominion House, 2013.

71. Grignon, J.S., J.H. Ledikwe, D. Makati, R. Nyangah, B.W. Sento et B. Semo, « Maximizing the benefit of health workforce secondment in Botswana: an approach for strengthening health systems in resource-limited settings », *Risk Manag Healthc Policy*, vol. 7, 2014, p. 91-98.

72. Jewell, C.J., et L.A. Bero, « ”Developing good taste in evidence”: facilitators of and hindrances to evidence-informed health policymaking in state government », *Milbank Q*, vol. 86, n^o^ 2, 2008, p. 177-208

73. *Données probantes et systèmes de santé au Nigéria (NEHSI). Le point sur le projet*. Sur Internet : https://www.idrc.ca/sites/default/files/sp/Documents%20EN/NEHSI-Update-English.pdf. Consulté le 21 mai 2016. Publié en 2007.

74. Vidéo du CIET sur le bilan social de l’initiative NEHSI (*CIET Methods Documentary*), 2013. Sur Internet : <https://www.youtube.com/watch?v=0CyYa8eJNww>.

75. *NEHSI docudrama on childhood illnesses, Bauchi State, Nigeria*, 2012. Sur Internet : [https://www.youtube.com/watch?v=ahHpwqhndg8](file:///\\hqvm42\multitranspro\TRADUCTI\.%202012.%20https:\www.youtube.com\watch%3fv=ahHpwqhndg8).

76. Andersson, N., « Proof of impact and pipeline planning: directions and challenges for social audit in the health sector », *BMC Health Serv Res.*, vol. 11 (suppl. 2), 2011, p. S16.

77. Newman, K., C. Fisher et L. Shaxson, « Stimulating Demand for Research Evidence: What role for capacity building? », *IDS Bull*, vol. 43, n^o^ 5, 2012, p. 17-24.

78. El-Jardali, F., J.N. Lavis, N. Ataya, D. Jamal, W. Ammar et S. Raouf, « Use of health systems evidence by policymakers in eastern Mediterranean countries: views, practices, and contextual influences », *BMC* *Health Serv Res*, vol. 12, 2012, p. 200.

79. Corluka, A., A.A. Hyder, E. Segura, P. Winch et RKD McLean, « Survey of Argentine Health Researchers on the Use of Evidence in Policymaking », *PLoS ONE*, vol. 10, n^o^ 4, 2015, p. e0125711.

80. Orton, L., F. Lloyd-Williams, D. Taylor-Robinson, M. O’Flaherty et S. Capewell, « The use of research evidence in public health decision making processes: systematic review », *PLoS ONE*, vol. 6, 2011, p. e21704.

81. D. Davis et N. Davis, « Educational interventions », *in* Straus, S., J. Tetroe et I.D. Graham (dir.), *Knowledge Translation in Healthcare: Moving from Evidence to Practice*, Chichester, Wiley-Blackwell, 2009, p. 113-122.

82. Ellen, M.E., G. Léon, G. Bouchard, J.N. Lavis, M. Ouimet et J.M. Grimshaw, « What supports do health system organizations have in place to facilitate evidence-informed decision-making? A qualitative study », *Implement Sci*, vol. 8, 2013, p. 84.

83. Haahr, J.H., H. Shapiro et S. Sørensen, *Defining a Strategy for the Direct Assessment of Skills*, Danish Technological Institute, 2004.
